# DABCO-Catalyzed Synthesis of Thiazolidine-2-thiones: System Development and Mechanistic Insights

**DOI:** 10.1021/acs.joc.5c00020

**Published:** 2025-03-19

**Authors:** Savvas G. Chalkidis, Sungil Hong, Anthi-Markella Tsiadi, Evangelia Fika, Nikolaos Tsoureas, Giannis Mpourmpakis, Georgios C. Vougioukalakis

**Affiliations:** † Laboratory of Organic Chemistry, Department of Chemistry, National and Kapodistrian University of Athens, Panepistimiopolis, Athens 15784, Greece; ‡ Department of Chemical and Petroleum Engineering, University of Pittsburgh, Pittsburgh, Pennsylvania 15261, United States; § School of Chemical Engineering, National Technical University of Athens (NTUA), Athens GR-15780, Greece; 4 Laboratory of Inorganic Chemistry, Department of Chemistry, National and Kapodistrian University of Athens, Athens 15784, Greece

## Abstract

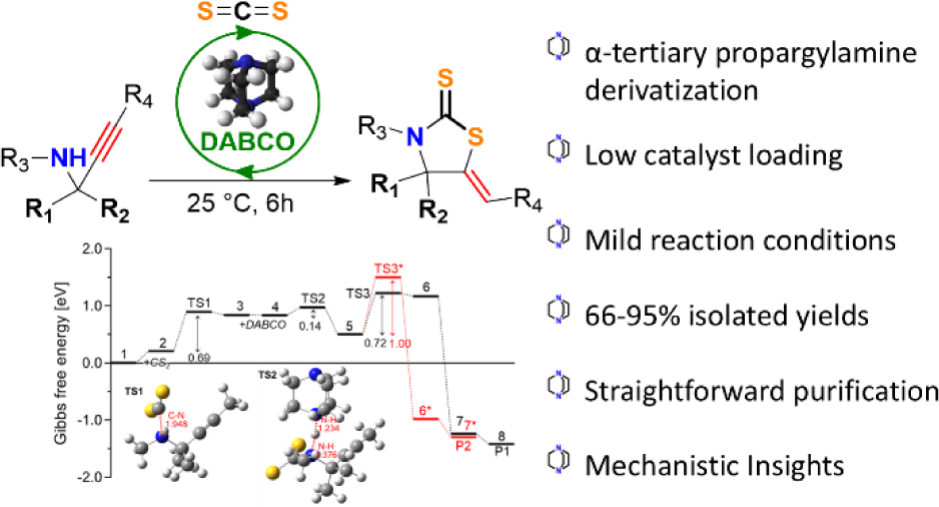

A base-catalyzed protocol is reported for the construction of 1,3-thiazolidine-2-thione scaffolds bearing quaternary carbon centers from carbon disulfide and α-tertiary propargylamines. The reaction proceeds using low catalyst loading, under ambient temperatures, and in the absence of solvent. Various α-tertiary propargylamines have been employed, affording a series of previously unreported thiazolidine-2-thione compounds and avoiding purification via column chromatography in certain cases. We also describe a one-pot strategy for the synthesis of the same products through a KA^2^ coupling–CS_2_ incorporation approach. The reaction mechanism and substituent-dependent catalytic behavior were studied through a combination of detailed experimental and computational studies.

## Introduction

The unique structure of propargylamines, encompassing a nucleophilic amine adjacent to an electrophilic alkyne moiety, renders them versatile building blocks for the preparation of numerous valuable organic structures and compounds of medicinal interest.^1−6^ Among the plethora of synthetic strategies aimed at their derivatization, significant emphasis has been placed on their reactivity with heteroallenes, targeting heterocycles featuring an exocyclic carbon-heteroatom bond.^6^ Extensive research has been devoted to the reactivity of propargylamines with carbon dioxide for the construction of substituted oxazolidinones and oxazolones.^7^ This transformation was developed almost 40 years ago, when it was demonstrated that propargylamines undergo cyclization with carbon dioxide in the presence of metal catalysts or under basic conditions.^8−11^ Since then, numerous transition metal-catalyzed methodologies have been reported, utilizing silver,^12−16^ gold,^17−20^ copper,^21−23^ palladium,^24,25^ or cobalt.^26,27^ Metal-free,^28−32^ heterogeneous,^33−38^ and one-pot A^3^/KA^2^ coupling–CO_2_ trapping^39−44^ synthetic protocols have also been developed. Additionally, considerable effort has been devoted to the utilization of alternative heteroallenes, such as carbonyl sulfide (COS),^45^ isocyanates,^46−52^ isothiocyanates,^48,53−58^ carbodiimides,^50,59−63^ and ketenimines,^64−67^ in tandem with propargylamines, providing immediate access to a diverse array of heterocyclic compounds. The *in situ* generation of heteroallenes, particularly carbonyl sulfide and carbonyl selenide, by employing elemental sulfur or selenium in conjunction with carbon monoxide or carbon monoxide surrogates, toward the synthesis of 1,3-thiazolidine-2-ones and 1,3-selenazolidine-2-ones, has also been exploited.^68−70^

**Figure 1:**
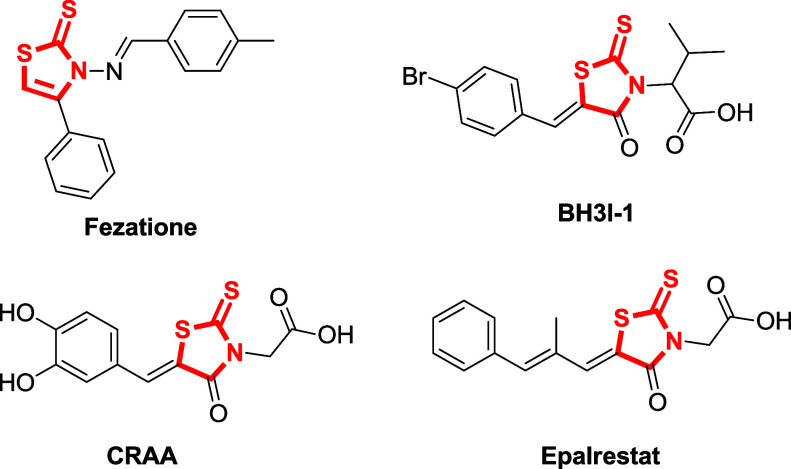
Examples of biologically active compounds featuring the thiazolidine/thiazole-2-thiones scaffold.

However, carbon disulfide, a valuable building block for the synthesis of sulfur-containing compounds,^71−74^ has received considerably less attention as a reaction partner of propargylamines toward the preparation of thiazolidine-2-thiones, despite the fact that this motif and the closely related thiazole-2-thione and dithiocarbamate moieties appear in numerous biologically relevant scaffolds with diverse pharmacological activities.^75−82^ Selected examples, among others, include Fezatione,^83^ an antifungal agent; BH3I–1,^84^ an apoptosis regulator agent used in cancer chemotherapy; CRAA,^85^ a privileged scaffold that binds to NAD(P)(H)-binding protein drug targets;^86^ and Epalrestat,^87^ an aldose reductase inhibitor (Figure 1).

Early reports on this transformation were published by Weedon,^88^ Teach,^89^ and coworkers, who demonstrated that simple propargylamines readily cyclize with carbon disulfide to prepare 1,3-thiazolidine-2-thiones. More recently, Van der Eycken and coworkers developed a sequential, one-pot protocol for the preparation of 1,3-thiazolidine-2-thiones from carbon disulfide and propargylamines derived from the A^3^ coupling reaction, resulting in a scaffold featuring one carbon substituent at position 4 of the five-membered ring.^90^ The A^3^ reaction in this transformation is catalyzed by CuBr, while the cyclization of the corresponding propargylamine with carbon disulfide occurs in the absence of a catalyst or other activators. The synthesis of fused heterocyclic systems, through the derivatization of propargylanilines and propargylamides with carbon disulfide, has also been described.^91,92^

Based on the scarcity of synthetic methodologies toward thiazolidine-2-thiones featuring two carbon substituents at position 4 of the heterocyclic ring, and our continued interest in the synthesis and derivatization of propargylic compounds,^58,93−101^ we report herein a straightforward, DABCO-catalyzed protocol for the synthesis of thiazolidine-2-thiones from carbon disulfide and polysubstituted α-tertiary propargylamines derived from the KA^2^ coupling. The mechanism of this approach was also comprehensively investigated, both experimentally and computationally.

## Results and Discussion

**Figure 2:**
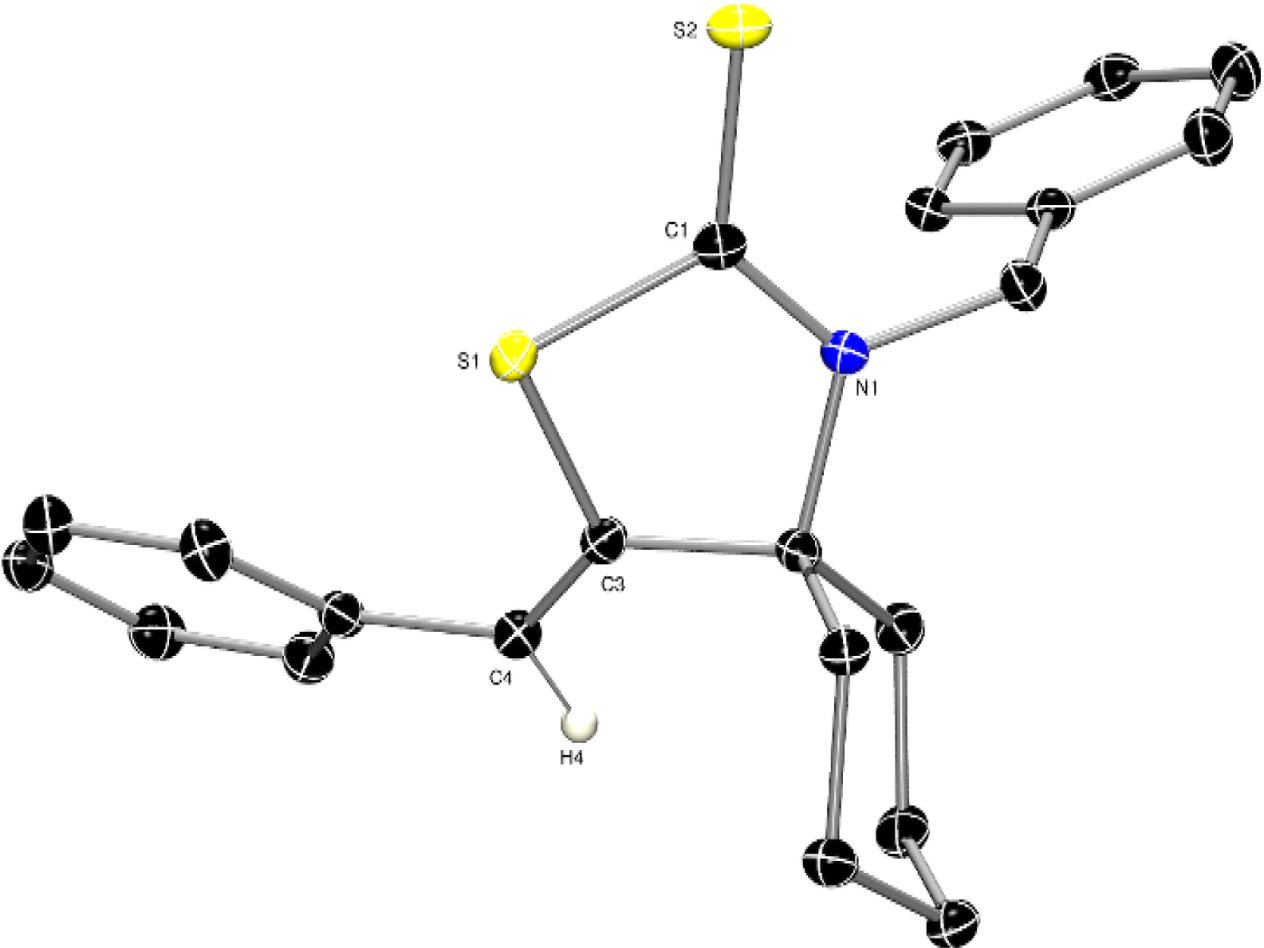
ORTEP diagram of the molecular structure of **2a** displaying 50% ADP‘s. H atoms (except H4) have been omitted for clarity (CCDC = 2388912). Selected bond lengths (Å) and angles (°): C1–S1:1.7554(12), S1–C3:1.7720(11), C1–S2:1.6646(11), N1–C1:1.3308(14), C3–C4:1.3364(16), C4–H4:0.9500; S1–C1–S2:120.81(6), C4–C3–S1:124.25(9), C3–S1–C1:91.81(5), N1–C1–S2:128.01(9), N1–C1–S1:111.18(8), C3–C4–H4:115.87(11).

We began our study by heating the α-tertiary propargylamine **1a** in toluene at 100 °C in an oil bath in the presence of 5 equiv of carbon disulfide, but no product was observed after 24 h of stirring (Table 1, entry 1). Taking into consideration that Van der Eycken and coworkers have found that α-secondary propargylamines undergo spontaneous cyclization with carbon disulfide under similar conditions,^90^ our observation highlights the considerably lower reactivity of α-tertiary propargylamines. These highly substituted species are recognized as challenging substrates in other related studies as well.^21,102−106^ Nevertheless, the incorporation of 1 equiv of DBU into the system led to the desired product (**2a**) in 84% yield (calculated by ^1^H NMR) (entry 2). Besides **2a**, we also detected the formation of thiazolidine-2-one **3** in 16% yield, most probably formed through oxidation by atmospheric O_2_, or by hydrolysis of the thiocarbonyl moiety due to traces of water found in DBU. Thus, α-tertiary propargylamines require the use of an activator to react with carbon disulfide. Considering that the base most likely mediates the proton transfer from the nitrogen atom of the starting propargylamine to the alkene group of the final thiazolidine-2-thione, regenerating itself in the process, we explored the possibility of conducting the reaction under catalytic amounts of base. When 30 mol % of DBU was used, the desired product **2a** formed in 17% yield, along with 3% of the byproduct **3** (entry 3). We then conducted the reaction at higher concentrations in the absence of solvent, keeping the catalytic loading of DBU at 30 mol %, obtaining product **2a** in 91% yield and byproduct **3** in 6% yield (entry 4). Reducing the reaction time from 24 to 6 h leads to the desired product **2a** in 90% yield and **3** in 4% yield (entry 5). When DBU was replaced by DABCO, we observed complete conversion of propargylamine **1a** after 2 h; equally important, the formation of the thiazolidine-2-one **3** was fully suppressed (entry 6). On the other hand, triethylamine proved to be an unsuitable catalyst for this transformation, as only unreacted propargylamine was detected in this case (entry 7). The use of DBN in 30 mol % led to **2a** in 61% yield (entry 8), while inorganic bases Cs_2_CO_3_ and KOH did not lead to product formation (entries 9 and 10). Complete conversion of the starting material was also observed when 15 mol % of DABCO was used, after 2 h of stirring at 100 °C (entry 11). By conducting the reaction at room temperature, the desired product was obtained in 75% yield after 2 h (entry 12). By increasing the reaction time to 6 h, at room temperature, we achieved complete conversion of the starting material and the isolation of the desired product **2a** in 95% yield (entry 13). It is worth noting that in the case of complete conversion after 6 h of stirring (based on TLC analysis), the isolation of the thiazolidine-2-thione can be achieved by a simple filtration through a silica gel plug using dichloromethane, after removing the residual carbon disulfide under reduced pressure, thus eliminating the necessity for column chromatography. In cases of incomplete conversion, increasing the reaction time to 16 h allows for the complete consumption of the starting propargylamine, thereby enabling the purification of the desired product using silica plug filtration. The *Z*-conformation of the C=C bond (i.e., C3–C4) in **2a** was unambiguously determined by single-crystal X-ray crystallography (Figure 2).

**Table 1:**
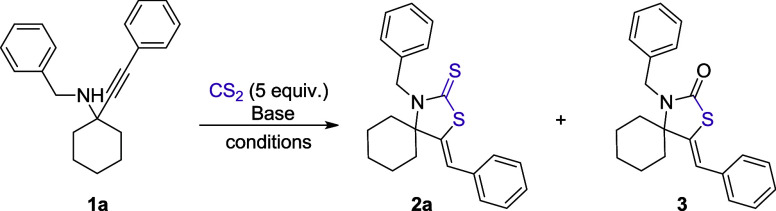
Probing and Optimizing the Reaction Conditions^a^

Entry	Base	Solvent	Temp.	Time	Yield **2a** [%]^b^	Yield **3** [%]^b^
1	-	Toluene (0.6 M)	100 °C	24 h	-	-
2	DBU (1 equiv)	Toluene (0.6 M)	100 °C	24 h	84	16
3	DBU (30 mol %)	Toluene (0.6 M)	100 °C	24 h	17	3
4	DBU (30 mol %)	-	100 °C	24 h	91	6
5	DBU (30 mol %)	-	100 °C	6 h	90	4
6	DABCO (30 mol %)	-	100 °C	2 h	100	-
7	Et_3_N (30 mol %)	-	100 °C	2 h	-	-
8	DBN (30 mol %)	-	100 °C	2 h	61	-
9	Cs_2_CO_3_ (30 mol %)	-	100 °C	2 h	-	-
10	KOH (30 mol %)	-	100 °C	2 h	-	-
11	DABCO (15 mol %)	-	100 °C	2 h	100	-
12	DABCO (15 mol %)	-	25 °C	2 h	75	-
**13**	**DABCO (15**mol %)	**-**	**25 °C**	**6 h**	**100 (95)**	**-**

a All reactions were carried out at 0.3 mmol scale.

b Yields calculated by ^1^H NMR, isolated yield after silica plug filtration in parentheses.

**Scheme 1:**
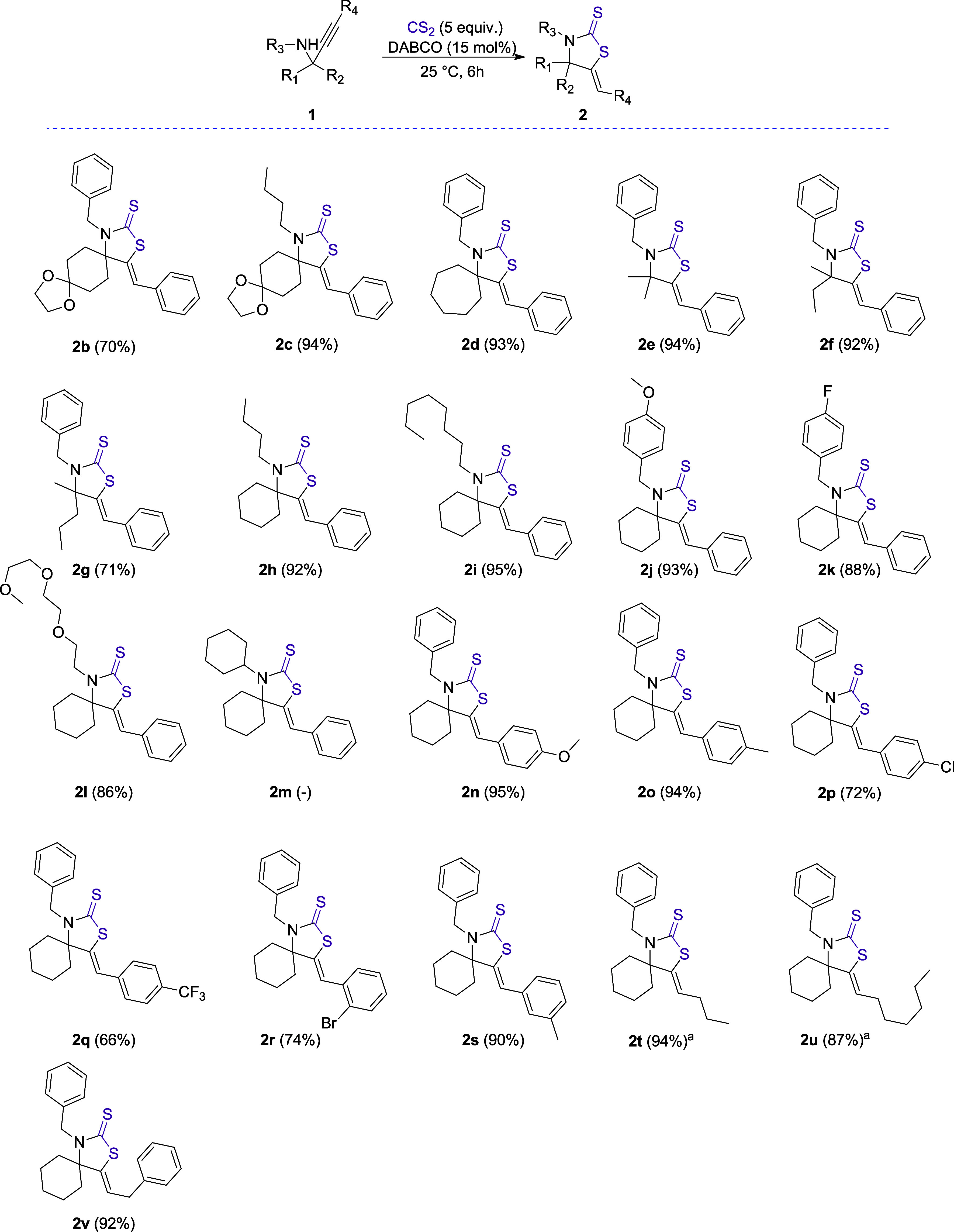
Substrate Scope Studies All reactions were carried out on a 0.3 mmol scale (isolated yields). DBU (15 mol%) was used.

With the optimized conditions in hand, we continued our investigation by employing a series of different α-tertiary propargylamines (Scheme 1). Propargylamines bearing cyclic acetal moieties led to compounds **2b** and **2c** in 70% and 94% isolated yields, respectively. Using a propargylamine with an expanded 7-membered carbocyclic ring, we obtained thiazolidine-2-thione **2d** in 93% yield. The utilization of propargylamines with noncyclic carbon substituents led to compounds **2e–2g** in very good to excellent yields as well (71–94%). The slightly reduced product yield obtained with a propargylamine bearing methyl and propyl groups (**2g**) is most probably due to the increased steric hindrance at the α-position of the nitrogen atom. Propargylamines featuring *N*-butyl, *N*-octyl, *N*-(4-methoxybenzyl), and *N*-(4-fluorobenzyl) groups resulted in compounds **2h**–**2k** in excellent yields (88–95%). Furthermore, we employed a propargylamine featuring a methoxyoligoethylene glycol group, leading to thiazolidine-2-thione **2l** in 86% yield. On the other hand, the utilization of a propargylamine bearing a bulkier *N*-cyclohexyl group did not lead to the desired product **2m**. Continuing our substrate scope studies, we utilized a series of propargylamines bearing various alkyne units. Propargylamines with electron-donating methoxy or methyl groups on the *para* position of the phenylacetylene moiety led to products **2n** and **2o** in excellent yields (95% and 94%), whereas the presence of electron-withdrawing chloro or trifluoromethyl groups resulted in a slight reduction in yields of 72% and 66% for products **2p** and **2q,** respectively. These results indicate a correlation between the electron density of the phenylacetylene moiety and the reactivity of the corresponding propargylamine toward cyclization (*vide infra*). Next, we examined the reactivity of propargylamines bearing substituents at the *ortho* or *meta* positions of the alkynyl phenyl ring. Product **2r**, starting from a propargylamine having a bromide substituent at the *ortho* position, was obtained in 74% yield, while product **2s**, originating from the utilization of a propargylamine carrying a *m*-tolylethynyl group, was isolated in 90% yield. Finally, we employed propargylamines bearing sp^3^-carbon atom-substituted alkyne moieties. Along these lines, thiazolidine-2-thiones featuring 3- or 6-carbon atom chains on the olefin moiety were prepared from the corresponding propargylamines using DBU as the catalyst, given that DABCO proved to result in very low yields for these particular substrates (<10%). Products **2t** and **2u** were isolated in 94% and 87% yields, respectively. On the other hand, compound **2v**, bearing a benzyl group attached to the olefin, was prepared in 92% yield starting from the corresponding propargylamine, using DABCO as the catalyst. The different reactivity between propargylamines bearing aliphatic carbon chains on the alkyne moiety (**1t** and **1u**) and those bearing aromatic or benzyl groups toward DABCO and DBU was elucidated using DFT studies (*vide infra*).

**Scheme 2:**
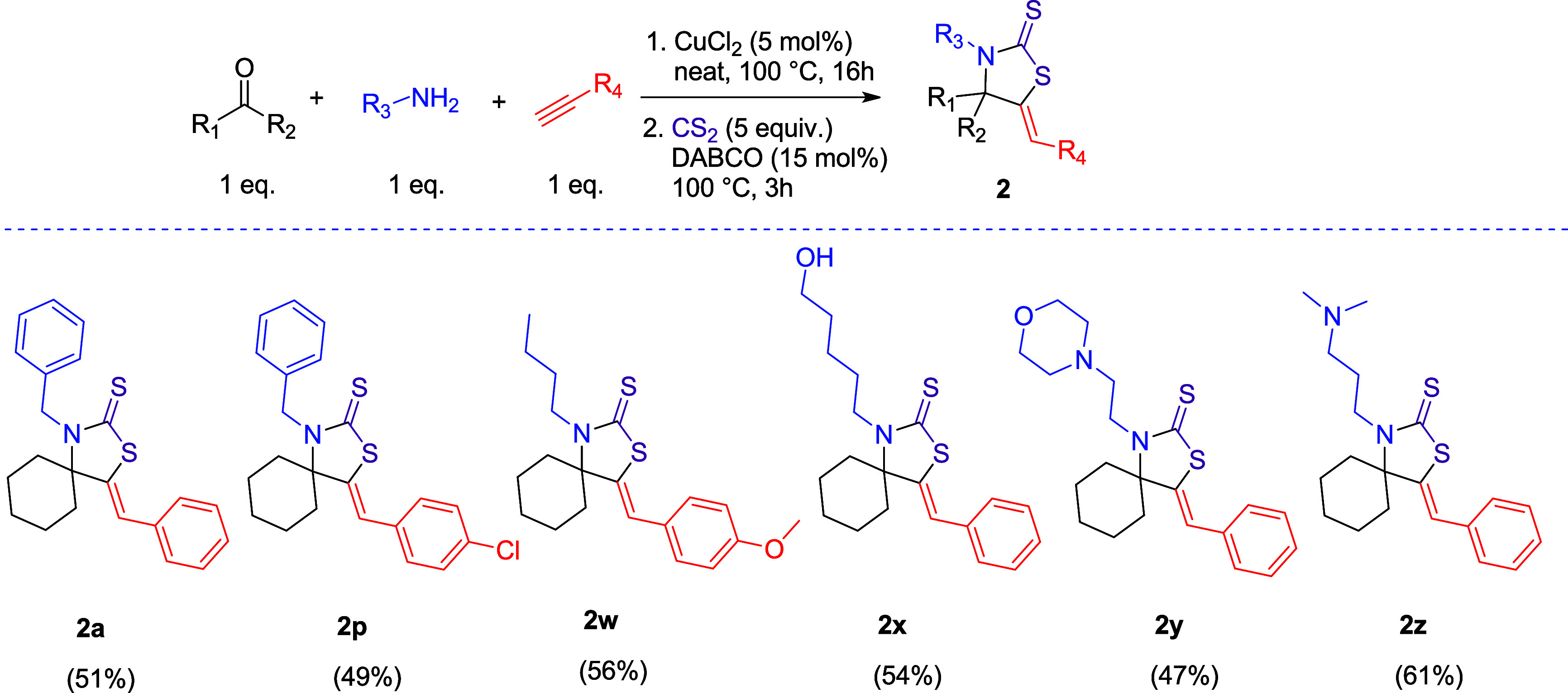
Two-Step, One-Pot Synthesis of Thiazolidine-2-Thiones All reactions were carried out at 1 mmol scale.

To explore additional synthetic strategies for the preparation of the desired compounds, we prepared a series of thiazolidine-2-thione products using a sequential, one-pot KA^2^ reaction–CS_2_ trapping methodology starting from commercially available building blocks (Scheme 2). In this regard, the α-tertiary propargylamine is prepared *in situ* by a Cu(II)-catalyzed KA^2^ coupling reaction in the first step, using the conditions developed by Larsen’s group.^107^ Subsequently, 5 equiv of carbon disulfide and 15 mol % of DABCO are added, leading to the desired product while circumventing the intermediate propargylamine isolation. This approach offers rapid access to thiazolidine-2-thione scaffolds but results in lower overall yields (83% vs 51%, two-step vs one-pot, for **2a**) and more challenging purification procedures due to the presence of byproducts. Products **2a**, **2p**, **2w**, **2x**, **2y,** and **2z** were synthesized by the one-pot approach in moderate to good yields (49–61%).

**Figure 3:**
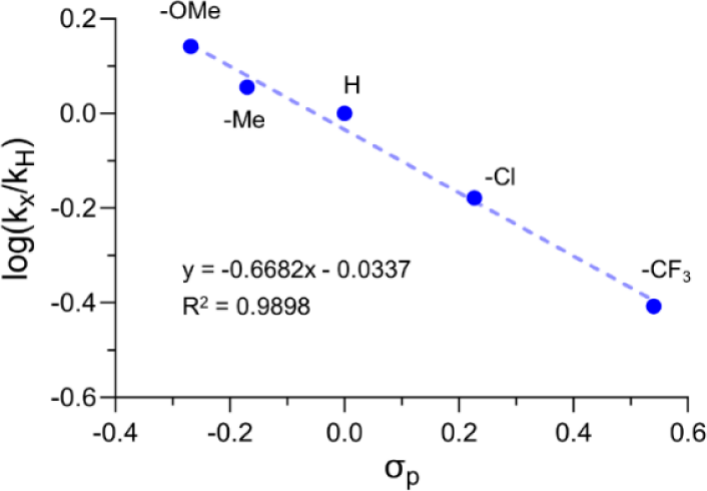
Hammett plot determined by competition experiments with *para*-substituted propargylamines.

Next, we focused on investigating the mechanism of the reaction. We considered that the transformation could proceed through the formation of a dithiocarbamate anion, generated from the base-assisted nucleophilic attack of the propargylamine on carbon disulfide, followed by an intramolecular 5-*exo*-*dig* cyclization step (attack of the dithiocarbamate anion and proton transfer from the protonated base to the triple bond), yielding the thiazolidine-2-thione product. To confirm our hypothesis, a Hammett study was initially carried out. Competition experiments were performed under the optimized reaction conditions using equimolar amounts of propargylamine **1a** and propargylamines bearing different *para* substituents (OMe, Me, Cl, CF_3_) on the phenyl ring attached to the alkyne. The relative ratios of the products were determined by ^1^H NMR (see Figure S1). The study revealed a very good linear correlation (*R*^2^ = 0.99) between the log(*k*_*X*_*/k*_*H*_*)* and Hammett σ_p_ constants, with a negative slope of −0.67 (Figure 3). The negative ρ-value (−0.67) measured confirms that electron-donating groups on the alkynyl phenyl group increase the rate of the reaction. This result implies that the first step is rate-determining (nucleophilic attack of the propargylamine nitrogen atom on carbon disulfide), during which a loss of electron density takes place at the reaction site. The small ρ-value can be attributed to the large distance (7 carbon atoms) between the nitrogen atom and each *para* substituent.

**Figure 4:**
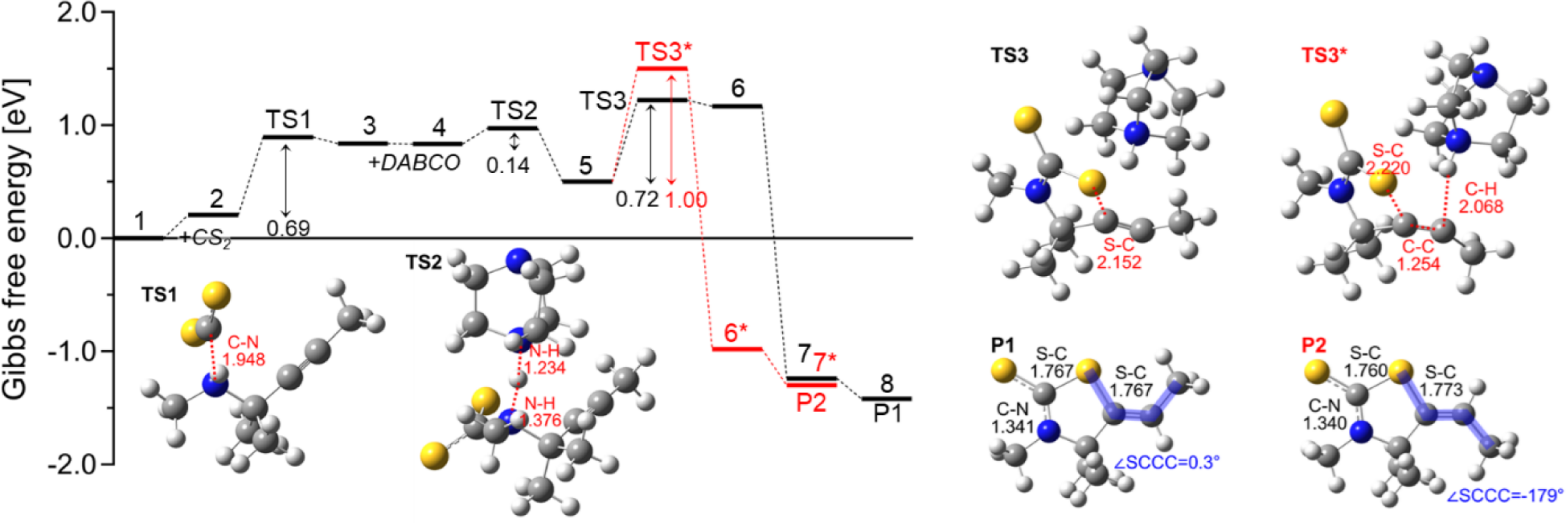
Gibbs free energy profiles of DABCO-catalyzed reactions between α-tertiary propargylamine and carbon disulfide (CS_2_) to *Z*- and *E*-isomer of thiazolidine-2-thiones (P1 and P2, respectively) determined using DFT calculations at 25 °C and 1 atm. Methyl groups (−CH_3_) are used as substituents for computational simplicity. Optimized molecular structures of transition states (TS) are shown with the interatomic distances associated with the imaginary modes. P1 and P2 are also shown with selected interatomic distances and dihedral angles. Atoms are color-coded based on the atom type (white = H, gray = C, blue = N, and yellow = S).

We carried out Density Functional Theory (DFT) calculations to further test our hypothesis regarding the reaction mechanism. We performed initial calculations of DABCO-catalyzed thiazolidine-2- thione formation (Figure 4) with methyl groups (−CH_3_) being used in place of all substituents (R1–R4 in α-tertiary propargylamine **1**) to reduce computational time before investigating the substrates used in our experiments. The first step is the nucleophilic attack of propargylamine on carbon disulfide, which involves a considerable activation energy (TS1, ΔG^‡^ = 0.69 eV). Next, DABCO is introduced, abstracting a proton from N with a small barrier (TS2, ΔG^‡^ = 0.14 eV). Thereafter, the reaction can proceed via two different pathways. The first is a stepwise mechanism that involves C–S bond formation (TS3) followed by a barrierless proton transfer from DABCO-H^+^ to the C backbone, leading to the *Z*-conformation of the C–C double bond (P1). The second is a concerted mechanism, where C–S bond formation and proton transfer occur simultaneously (TS3*), leading to the *E*-isomer of the product (P2). The stereoselectivity of P2, determined based on the activation energies of TS3 vs TS3* (i.e., the Curtin-Hammett principle^108,109^) is found to be 1.98 × 10^−5^, which agrees with the absence of P2 in experiments. We also considered a reaction mechanism initiated with CS_2_ addition to DABCO followed by CS_2_ transfer to propargylamine, but it was not competitive with the overall reaction mechanism (see Section S6.1).

**Figure 5:**
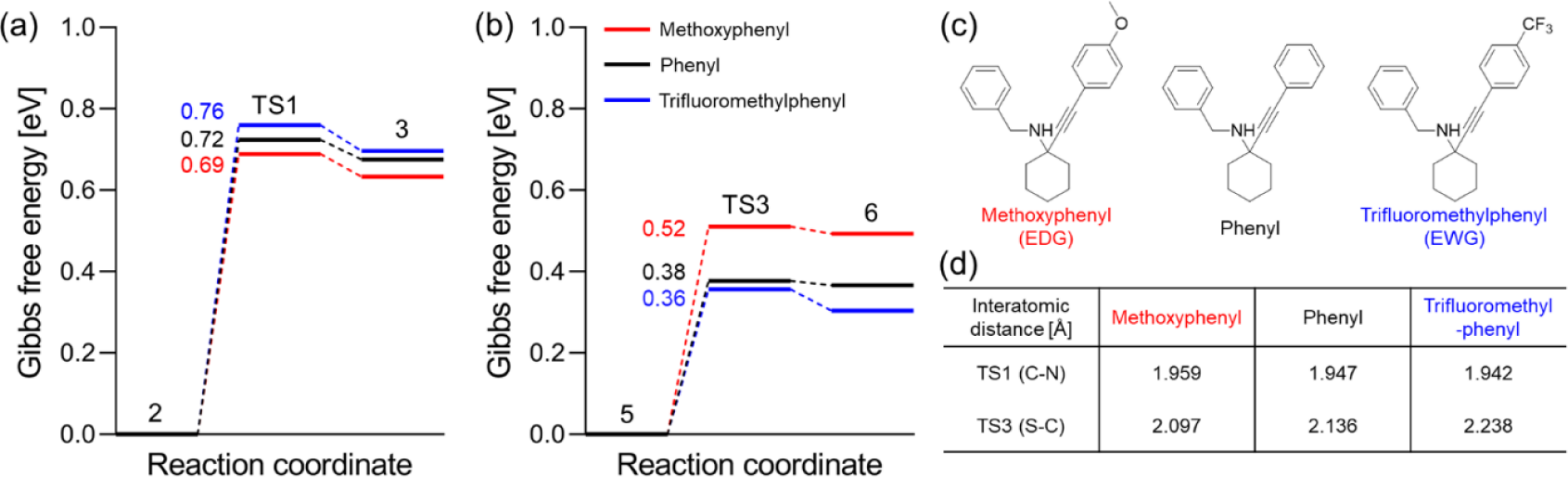
Gibbs free energy profiles of (a) TS1 and (b) TS3 (presented in Figure 4) of DABCO-catalyzed α-tertiary propargylamine and CS_2_ to thiazolidine-2-thiones bearing methoxyphenyl (red), phenyl (black), and trifluoromethylphenyl (blue) substituents. (c) Structures of the reactant species. (d) Characteristic interatomic distances of the TSs with each substituent.

The reaction pathway of DABCO-catalyzed thiazolidine-2-thiones (P1) formation in Figure 4 is characterized by two TSs with comparable activation energies (TS1, 0.69 eV vs. TS3, 0.72 eV). To identify the rate-determining step, the TS search was repeated after the methyl groups were replaced with the actual substituents used in experiments. More specifically, the α-tertiary propargylamine **1a** and its derivatives with methoxy and trifluoromethyl groups on the *para* position of the phenylacetylene moiety were considered (Figure 5c). Note that the reaction products are **2a**, **2n**, and **2q**, respectively. Figure 5a,b shows that the activation energies of TS1 (≥0.69 eV) are consistently higher than those of TS3 (≤0.52 eV), which indicates that TS1 (nucleophilic attack of the propargylamine nitrogen atom to CS_2_) is the actual rate-limiting step. As expected, the presence of the electron-donating methoxy group decreases the activation energy, whereas the electron-withdrawing trifluoromethyl group leads to a higher barrier (Figure 5a), in agreement with our experimental observations (Figure 3). It should be noted that although the activation energy of TS3 shows trends opposite to those of TS1 with respect to the substituents (Figure 5b), since this cyclization step involves electron donation from the negatively charged sulfur atom to the alkynyl carbon, TS1 is still higher in energy and remains rate-determining. The electronic effects of the *para* substituents are reflected in the characteristic distances of the TSs (Figure 5d) and the HOMO–LUMO energy gap of state 2 (reactant of TS1; Section S6.2). Overall, the DFT results are in excellent agreement with the experimentally derived Hammett plot.

**Figure 6:**
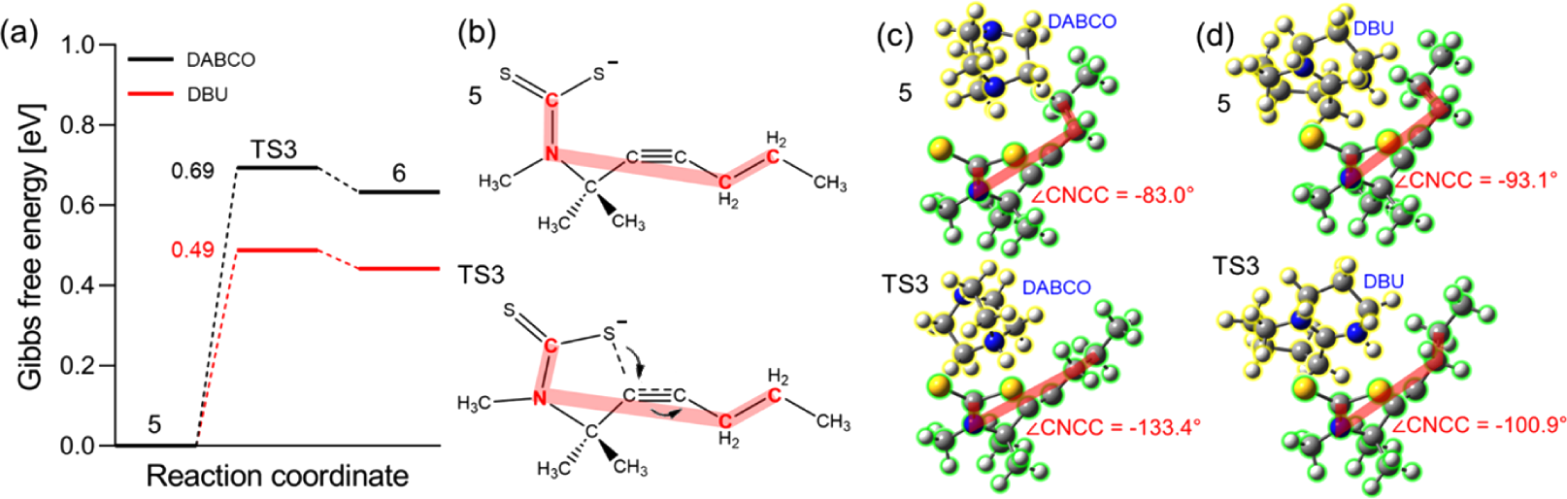
(a) Gibbs free energy profiles of TS3 on the reaction mechanisms of DABCO (black) and DBU (red) catalysis of α-tertiary propargylamine and CS_2_ to thiazolidine-2-thiones bearing a propyl group at the terminal position of the alkyne moiety. Methyl groups are used in place of all of the other substituents for computational simplicity. (b) Dithiocarbamate anion structures in state 5 and TS3 with a characteristic dihedral angle marked in red. Optimized molecular structures of states 5 and TS3 in the presence of (c) DABCO and (d) DBU, where the protonated catalysts and dithiocarbamate anions are highlighted in yellow and green, respectively.

To elucidate the significantly lower yield of the DABCO-catalyzed synthesis of thiazolidine-2-thiones featuring alkyl chains on the triple bond (**2t** and **2u**), compared to that of DBU-catalyzed, we performed additional DFT calculations. A propyl group was added at the terminal position of the alkyne moiety (see R4 in α-tertiary propargylamine **1** in Scheme 1) and methyl groups were used in place of the remaining substituents (R1–R3). The experimentally observed reactivity difference is likely associated with the second most kinetically important step, TS3 (cyclization via C–S bond formation), since the rate-determining TS1 proceeds in the absence of any catalyst (Figure 4). Although the catalysts do not directly participate in the C–S bond formation step, their Lewis conjugates (i.e., DABCO-H^+^ or DBU-H^+^) are involved in state 5, which strongly interact with a dithiocarbamate anion (ΔG of removal of a protonated catalyst from state 5 is >3.6 eV) and, therefore, may influence the energy barrier leading to TS3. We calculated the activation energy of TS3 to be significantly higher in the presence of DABCO compared to DBU (0.69 vs 0.49 eV; Figure 6a). This is primarily due to the steric hindrance created by the bulky DABCO molecule, which destabilizes the TS relative to the initial state (state 5), as demonstrated by the significant distortion of a propyl end group relative to a CS_2_-propargylamine nitrogen moiety (Figure 6c and Section S6.3). In contrast, the planar DBU catalyst does not give rise to significant steric effects (Figure 6d). It should be noted that such destabilization of DABCO on TS3 is not prominent on substrates bearing aromatic rings on the alkynyl group, as evidenced by the high yield of **2v**. Furthermore, the π-orbitals of the benzene moieties stabilize the bulky DABCO catalyst (ΔG of interactions between dithiocarbamate anions and protonated catalysts are −4.03 and −3.68 eV for DABCO and DBU, respectively), which compensates for the steric effects and therefore leads to the nearly identical activation energies of TS3 in DABCO- and DBU-catalysis (Figure S6a). This emphasizes the importance of intermolecular interactions between catalysts and the substituents of α-tertiary propargylamines in the synthesis of thiazolidine-2-thiones.

**Scheme 3:**
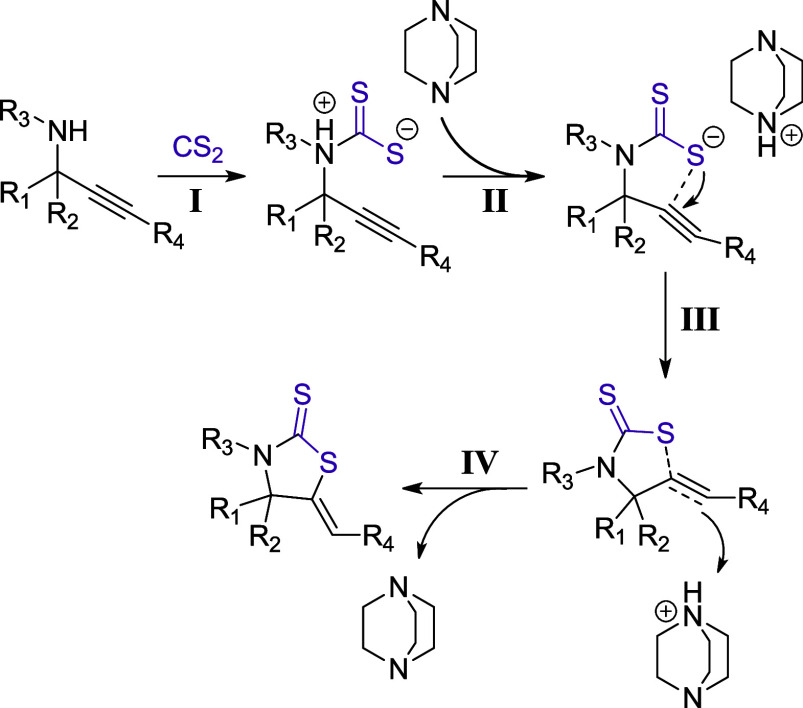
Proposed Mechanism for the DABCO-Catalyzed Synthesis of Thiazolidine-2-Thiones

Based on our results and the related literature,^45,58,69,90^ a proposed mechanism for the DABCO-catalyzed synthesis of thiazolidine-2-thiones is presented in Scheme 3. In the rate-determining step **I**, a nucleophilic attack of the propargylamine on carbon disulfide takes place, leading to a zwitterion. During Step **II**, DABCO abstracts the proton from the propargylamine nitrogen, yielding a dithiocarbamate anion and DABCO-H^+^ as a counterion. The negatively charged sulfur atom attacks the π-system of the triple bond (*5-exo-dig* cyclization, Step **III**) and during Step **IV**, a proton transfer from DABCO-H^+^ to the π-system occurs, yielding the final 1,3-thiazolidine-2-thione product and regenerating the DABCO catalyst.

## Conclusion

We have herein established a DABCO-catalyzed protocol for the synthesis of 4,4-disubstituted-1,3-thiazolidine-2-thiones, starting from various α-tertiary propargylamines and carbon disulfide. Our methodology works efficiently under mild reaction conditions, using low catalyst loading at room temperature, in the absence of solvent, furnishing the desired products in good to excellent yields (66–95%) starting from a variety of propargylamine substrates and, in certain cases, eliminating the need for column chromatography. A two-step, one-pot synthetic approach was also developed, leading to thiazolidine-2-thiones in moderate to good yields (49–56%). Our system thus offers a new method for the derivatization of α-tertiary propargylamines, enabling the preparation of 5-alkenyl-thiazolidine-2-thione derivatives bearing quaternary carbon centers. The detailed reaction mechanism was elucidated through a combination of Hammett experimental kinetic studies and DFT calculations. We revealed the first step of the transformation (i.e., the nucleophilic attack of the propargylamine on CS_2_) to be rate-determining. Electron-donating groups attached to the propargylamine alkyne moiety were found to increase the reaction rate, with the observed reactivity trends between theory and experiments being in excellent agreement. Furthermore, computational studies shed light on the *E*/*Z* stereoselectivity and revealed significant substitution and steric hindrance effects between the catalytic species and the triple bond substituents of the propargylamines on kinetically important elementary steps, rationalizing our experimental observations.

## Experimental Section

### General Procedure for the Synthesis of Thiazolidine-2-thiones from Propargylamines

Propargylamine (0.3 mmol, 1 equiv), DABCO (5 mg, 0.045 mmol, 15 mol %), and CS_2_ (114 mg, 90 μL, 1.5 mmol, 5 equiv) were added to a 4 mL vial under air. The reaction was stirred at 25 °C for 6 h. Then, the reaction mixture was diluted with CH_2_Cl_2_ and concentrated under reduced pressure. The crude product was isolated by filtration through a silica gel plug or by column chromatography, depending on propargylamine conversion.

### General Procedure for the One-Pot Synthesis of Thiazolidine-2-thiones

Ketone (1 mmol, 1 equiv), amine (1 mmol, 1 equiv), alkyne (1 mmol, 1 equiv), and anhydrous CuCl_2_ (6.7 mg, 0.05 mmol, 5 mol %) were added to a flame-dried Schlenk tube. The reaction was stirred at 100 °C for 16 h in an oil bath. DABCO (16.8 mg, 0.15 mmol, 15 mol %) and CS_2_ (380 mg, 300 μL, 5 mmol, 5 equiv) were added to the reaction mixture, and the reaction was stirred for 3 h. The volatiles were removed under reduced pressure, and the product was purified by column chromatography.

### Density Functional Theory (DFT) Calculations

Density functional theory (DFT) calculations were performed using the Gaussian 09 software package.^110^ The M06-2X functional, coupled with a split-valence triple-ζ basis set with diffuse functions, 6-311+G(d,p) was used since it has been shown to accurately describe main-group chemistry.^111^ Vibrational frequency calculations were performed to confirm that the stationary points are either minima or saddle points on the potential energy surfaces. Intrinsic Reaction Coordinate (IRC) calculations^112^ further confirmed that the optimized transition state (TS) structures connect the corresponding reactants and products. All of the reaction energies were described in terms of Gibbs free energy, determined at 25 °C and 1 atm.

## Data Availability

The data underlying this study are available in the published article and its online Supporting Information.

## Supporting Information Available

Experimental procedures, spectroscopic data, copies of NMR spectra, additional DFT calculations, Cartesian coordinates, and crystallographic data for **2a** (PDF)
