# Supplementary material for: DABCO-Catalyzed Synthesis of Thiazolidine-2-thiones: System Development and Mechanistic Insights
Source: J Org Chem. 2025 Mar 19;90(12):4302–12. doi: 10.1021/acs.joc.5c00020 (PMC11959531; doi:10.1021/acs.joc.5c00020)
Supplement: Supplementary file 1 — jo5c00020_si_001.pdf [file jo5c00020_si_001.pdf]

Electronic Supplementary Information

DABCO-Catalyzed Synthesis of Thiazolidine-2-thiones: System Development and Mechanistic Insights

Savvas G. Chalkidis,[a] Sungil Hong,[b] Anthi-Markella Tsiadi,[a] Evangelia Fika,[a] Nikolaos Tsoureas,[a] Giannis Mpourmpakis[b,c,]* and Georgios C. Vougioukalakis[a]*

[a] Laboratory of Organic Chemistry, Department of Chemistry, National and Kapodistrian University of Athens, Panepistimiopolis, 15784 Athens, Greece
E-mail: vougiouk@chem.uoa.gr

[b] Department of Chemical and Petroleum Engineering, University of Pittsburgh, Pittsburgh, PA 15261, USA
E-mail: gmpourmp@pitt.edu

[c] School of Chemical Engineering, National Technical University of Athens (NTUA), Athens, GR-15780, Greece.

Contents

1. General S2
2. General procedure for the synthesis of new propargylamines (A) S3
3. General procedure for the synthesis of thiazolidine-2-thiones starting from propargylamines (B) S7
4. General procedure for the one-pot synthesis of thiazolidine-2-thiones (C) S7
5. Competition Experiments S18
6. DFT Studies S19
7. Single Crystal X-ray Crystallography S23
8. NMR Spectra S25
9. Optimized molecular structures with DFT S65
10. References S113

1. General

All reactants and reagents were obtained from commercial suppliers and used as received. The course of the reactions was monitored *via* thin-layer chromatography (TLC), using aluminum sheets (0.2 mm) coated with silica gel 60 with a fluorescence indicator (silica gel 60 F254). The developed TLC plates were analysed by UV lamp (254 nm and 365 nm) or by potassium permanganate solution for visualization. Purification of the products was carried out by column chromatography or silica plug, using silica gel 60 (230−400 mesh). Nuclear magnetic resonance (NMR) spectra were obtained on a Bruker Avance 400 MHz (^1H) or a Varian Mercury 200 MHz (^1H) NMR spectrometers, using $CDCl_3$ as solvent. The spectra were referenced using either the solvent's residual proton signal (^1H) or the solvent's peak ($^{13}C\{^1H\}$). Data are reported as follows: chemical shift, multiplicity (s = singlet, d = doublet, t = triplet, q = quartet, quint = quintet, h = sextet, dq = doublet of quartets, ddd = doublet of doublet of doublets, td = triplet or quartets, qt = quartet of triplets and m = multiplet), coupling constant (Hz), and integration. HRMS spectra were recorded in a QTOF maxis impact (Bruker) spectrometer using positive electron spray ionization (ESI^+).

Propargylamines **1a, 1b 1e, 1h-1k, 1m and 1t-1u** were prepared according to literature procedures.[1–7]

2. General procedure for the synthesis of new propargylamines (A)

The synthesis of propargylamines was carried out according to the following literature procedures.[1,8] In a flame dried Schlenk tube equipped with a magnetic stirring bar, anhydrous $CuCl_2$ (20 mg, 0.15 mmol, 0.05 equiv.), ketone (3 mmol, 1 equiv.), alkyne (3 mmol, 1 equiv.) and amine (3 mmol, 1 equiv.) were added under an argon atmosphere. When linear ketones were used, $Ti(OEt)_4$ (342 mg, 314 µL 1.5 mmol, 0.5 equiv.) was added. The reaction was stirred at 100 °C in an oil bath for 16h. The products were purified by column chromatography (Petroleum Ether 40-60 - PE/EtOAc).

N-butyl-8-(phenylethynyl)-1,4-dioxaspiro[4.5]decan-8-amine (1c)

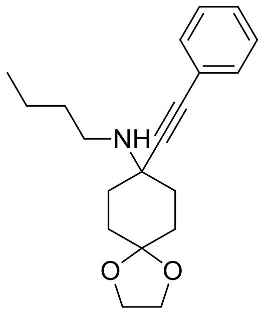

Prepared according to the general procedure **A**. **1c** was obtained as a yellow oil after purification with column chromatography (PE/EtOAc = 7/3) (677 mg, 72%). ^{1}H NMR (400 MHz, $CDCl_3$) δ 7.43 – 7.35 (m, 2H), 7.30 – 7.23 (m, 3H), 3.93 (s, 4H), 2.77 (t, J = 7.0 Hz, 2H), 2.09 – 1.84 (m, 4H), 1.82 – 1.72 (m, 4H), 1.57 – 1.44 (m, 2H), 1.42 – 1.29 (m, 2H), 0.92 (t, J = 7.2 Hz, 3H). ^{13}C{^{1}H} NMR (101 MHz, $CDCl_3$) δ 131.8, 128.3, 127.9, 123.6, 108.7, 92.8, 84.1, 64.4, 64.3, 54.0, 43.5, 35.6, 32.9, 31.6, 20.7, 14.1. HRMS (ESI-TOF): calcd for $C_{20}H_{28}NO_2$ [M + H$^+$] 314.2115, found 314.2120.

N-benzyl-1-(phenylethynyl)cycloheptanamine (1d)

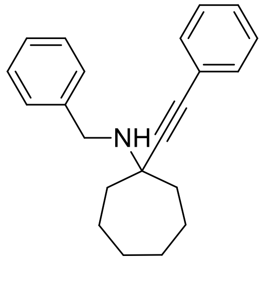

Prepared according to the general procedure **A**. **1d** was obtained as a yellow oil after purification with column chromatography (PE/EtOAc = 9/1) (419 mg, 46%). ^{1}H NMR (400 MHz, $CDCl_3$) δ 7.59 – 7.44 (m, 2H), 7.40 (d, J = 7.6 Hz, 2H), 7.35 – 7.28 (m, 5H), 7.28 – 7.20 (m, 1H), 3.96 (s, 2H), 2.08 – 1.99 (m, 2H), 1.84 – 1.55 (m, 10H). ^{13}C{^{1}H} NMR (101 MHz, $CDCl_3$) δ 141.2, 131.8, 128.6, 128.5, 128.4, 127.9, 127.0, 123.8, 94.8, 83.9, 58.5, 48.8, 40.9, 28.4, 22.8. HRMS (ESI-TOF): calcd for $C_{22}H_{25}N$ [M + H$^+$] 304.2060, found 304.2057.

N-benzyl-3-methyl-1-phenylpent-1-yn-3-amine (1f)

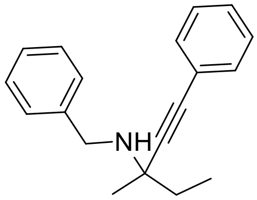

Prepared according to the general procedure **A**. **1f** was obtained as a yellow oil after purification with column chromatography (PE/EtOAc = 9/1) (498 mg, 63%). ^{1}H NMR (400 MHz, CDCl$_3$) δ 7.53 – 7.43 (m, 2H), 7.42 – 7.38 (m, 2H), 7.36 – 7.30 (m, 5H), 7.28 – 7.22 (m, 1H), 3.99 (d, *J* = 12.0 Hz, 1H), 3.94 (d, *J* = 12.0 Hz, 1H), 1.86 – 1.67 (m, *J* = 7.0 Hz, 2H), 1.45 (s, 3H), 1.10 (t, *J* = 7.5 Hz, 3H). ^{13}C{^{1}H} NMR (101 MHz, CDCl$_3$) δ 141.1, 131.8, 128.6, 128.4, 128.0, 127.1, 123.7, 93.9, 83.7, 54.8, 48.9, 34.9, 26.6, 9.1. HRMS (ESI-TOF): calcd for $C_{19}H_{22}N$ [M + H$^+$] 264.1747, found 264.1749.

N-benzyl-3-methyl-1-phenylhex-1-yn-3-amine (1g)

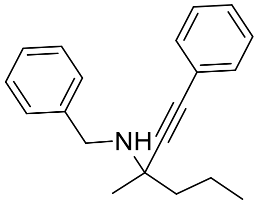

Prepared according to the general procedure **A**. **1g** was obtained as a yellow oil after purification with column chromatography (PE/EtOAc = 9/1) (483 mg, 58%). ^{1}H NMR (400 MHz, CDCl$_3$) δ 7.52 – 7.42 (m, 2H), 7.39 (d, *J* = 7.5 Hz, 2H), 7.35 – 7.28 (m, 5H), 7.28 – 7.21 (m, 1H), 3.98 (d, *J* = 11.9 Hz, 1H), 3.92 (d, *J* = 12.2 Hz, 1H), 1.80 – 1.66 (m, 2H), 1.62 – 1.50 (m, 2H), 1.46 (s, 3H), 0.98 (t, *J* = 6.9 Hz, 3H). ^{13}C{^{1}H} NMR (101 MHz, CDCl$_3$) δ 141.0, 131.8, 128.6, 128.6, 128.4, 128.0, 127.1, 123.7, 94.1, 83.6, 54.4, 48.9, 44.6, 27.2, 18.0, 14.6. HRMS (ESI-TOF): calcd for $C_{20}H_{24}N$ [M + H$^+$] 278.1903, found 278.1913.

N-(2-(2-(2-methoxyethoxy)ethoxy)ethyl)-1-(phenylethynyl)cyclohexanamine (1l)

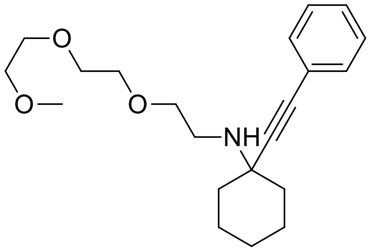

Prepared according to the general procedure **A**. **1l** was obtained as a yellow oil after purification with column chromatography (PE/EtOAc = 1/1, EtOAc 100%) (528 mg, 51%). ^{1}H NMR (400 MHz, CDCl$_3$) δ 7.43 – 7.37 (m, 2H), 7.31 – 7.24 (m, 3H), 3.67 – 3.60 (m, 8H), 3.55 – 3.49 (m, 2H), 3.35 (s, 3H), 2.99 (t, *J* = 5.4 Hz, 2H), 1.94 (d, *J* = 13.2 Hz, 2H), 1.75 – 1.58 (m, 5H), 1.52 – 1.36 (m, 2H), 1.32 – 1.15 (m, 1H). ^{13}C{^{1}H} NMR (101 MHz, CDCl$_3$) δ 131.7, 128.3, 127.8, 123.8, 93.3, 84.8, 72.0, 71.1, 70.7, 70.6, 70.2, 59.1, 54.9, 42.7, 38.3, 26.0, 23.2. HRMS (ESI-TOF): calcd for $C_{21}H_{32}NO_3$ [M + H$^+$] 346.2377, found 346.2381.

N-benzyl-1-((4-methoxyphenyl)ethynyl)cyclohexanamine (1n)

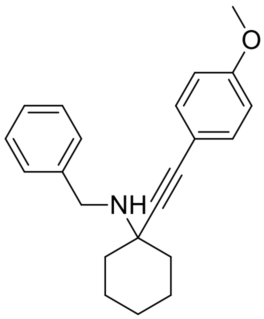

Prepared according to the general procedure **A**. **1n** was obtained as a yellow oil after purification with column chromatography (PE/EtOAc = 9/1) (728 mg, 76%). ^{1}H NMR (400 MHz, $CDCl_3$) δ 7.48 – 7.38 (m, 4H), 7.36 – 7.30 (m, 2H), 7.26 (tt, *J* = 6.3, 1.4 Hz, 1H), 6.92 – 6.80 (m, 2H), 3.99 (s, 2H), 3.82 (s, 3H), 1.99 (d, *J* = 12.0 Hz, 2H), 1.75 – 1.63 (m, 5H), 1.60 – 1.48 (m, 2H), 1.38 – 1.20 (m, 1H). ^{13}C{^{1}H} NMR (101 MHz, $CDCl_3$) δ 159.3, 141.3, 133.1, 128.6, 128.5, 127.0, 116.0, 114.0, 92.1, 84.6, 55.4, 55.4, 48.2, 38.4, 26.1, 23.2. HRMS (ESI-TOF): calcd for $C_{22}H_{26}NO$ [M + H^{+}] 320.2009, found 320.2021.

N-benzyl-1-(p-tolylethynyl)cyclohexanamine (1o)

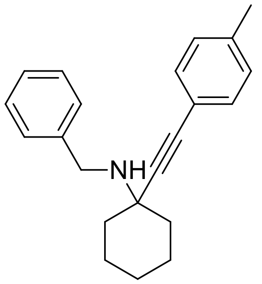

Prepared according to the general procedure **A**. **1o** was obtained as a yellow solid after purification with column chromatography (PE/EtOAc = 9/1) (646 mg, 73%). ^{1}H NMR (400 MHz, $CDCl_3$) δ 7.45 – 7.30 (m, 6H), 7.26 (t, *J* = 7.2 Hz, 1H), 7.14 (d, *J* = 7.7 Hz, 2H), 4.00 (s, 2H), 2.37 (s, 3H), 2.00 (d, *J* = 12.0 Hz, 2H), 1.77 – 1.67 (m, 5H), 1.60 – 1.49 (m, 2H), 1.33 – 1.26 (m, 1H). ^{13}C{^{1}H} NMR (101 MHz, $CDCl_3$) δ 141.3, 137.9, 131.7, 129.1, 128.6, 128.5, 127.0, 120.7, 92.9, 84.9, 55.5, 48.2, 38.4, 26.1, 23.2, 21.6. HRMS (ESI-TOF): calcd for $C_{22}H_{26}N$ [M + H^{+}] 304.2060, found 304.2065.

N-benzyl-1-((4-chlorophenyl)ethynyl)cyclohexanamine (1p)

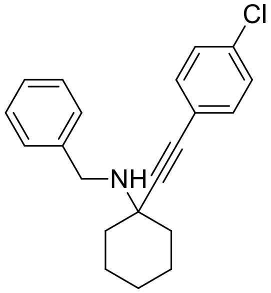

Prepared according to the general procedure **A**. **1p** was obtained as a yellow solid after purification with column chromatography (PE/EtOAc = 9/1) (651 mg, 67%). ^{1}H NMR (400 MHz, $CDCl_3$) δ 7.45 – 7.36 (m, 4H), 7.35 – 7.15 (m, 5H), 3.96 (s, 2H), 1.97 (d, *J* = 12.5 Hz, 2H), 1.73 – 1.52 (m, 7H), 1.35 – 1.24 (m, 1H).

$^{13}C\{^1H\}$ NMR (101 MHz, $CDCl_3$) δ 141.1, 133.9, 133.0, 128.7, 128.6, 127.0, 122.3, 94.8, 83.7, 55.5, 48.2, 38.2, 26.0, 23.1. HRMS (ESI-TOF): calcd for $C_{21}H_{23}ClN$ [M + H$^+$] 324.1514, found 324.1511.

N-benzyl-1-((4-(trifluoromethyl)phenyl)ethynyl)cyclohexanamine (1q)

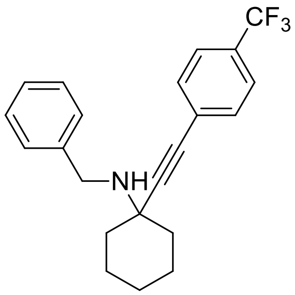

Prepared according to the general procedure **A**. **1q** was obtained as a yellow solid after purification with column chromatography (PE/EtOAc = 9/1) (729 mg, 68%). ^1H NMR (400 MHz, $CDCl_3$) δ 7.59 – 7.51 (m, 4H), 7.40 (d, *J* = 7.6 Hz, 2H), 7.33 (t, *J* = 7.4 Hz, 2H), 7.26 (m, 1H), 3.98 (s, 2H), 2.00 (d, *J* = 12.1 Hz, 2H), 1.84 – 1.51 (m, 7H), 1.36 – 1.23 (m, 1H). $^{13}C\{^1H\}$ NMR (101 MHz, $CDCl_3$) δ 140.9, 132.0, 129.7 (q, *J* = 32.8 Hz), 128.6, 128.6, 127.6, 127.1, 125.3 (q, *J* = 3.9 Hz), 124.1 (q, *J* = 272.7 Hz), 96.5, 83.7, 55.6, 48.2, 38.2, 26.0, 23.1. ^{19}F NMR (376 MHz, $CDCl_3$) δ -62.7. HRMS (ESI-TOF): calcd for $C_{22}H_{23}F_3N$ [M + H$^+$] 358.1777, found 358.1782.

N-benzyl-1-((2-bromophenyl)ethynyl)cyclohexanamine (1r)

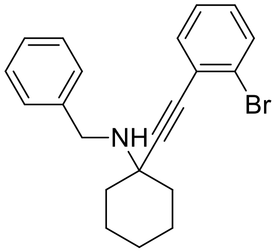

Prepared according to the general procedure **A**. **1r** was obtained as a yellow oil after purification with column chromatography (PE/EtOAc = 9/1) (585 mg, 53%). ^1H NMR (400 MHz, $CDCl_3$) δ 7.60 (d, *J* = 8.0 Hz, 1H), 7.49 (dd, *J* = 7.7, 1.7 Hz, 1H), 7.42 (d, *J* = 7.5 Hz, 2H), 7.33 (t, *J* = 7.5 Hz, 2H), 7.29 – 7.22 (m, 2H), 7.16 (td, *J* = 7.7, 1.7 Hz, 1H), 4.04 (s, 2H), 2.03 (d, *J* = 12.1 Hz, 2H), 1.83 – 1.65 (m, 5H), 1.56 (td, *J* = 11.8, 5.0 Hz, 2H), 1.34 – 1.24 (m, 1H). $^{13}C\{^1H\}$ NMR (101 MHz, $CDCl_3$) δ 141.2, 133.5, 132.5, 129.1, 128.7, 128.6, 127.1, 127.0, 125.9, 125.7, 98.8, 83.5, 55.8, 48.3, 38.2, 26.1, 23.1. HRMS (ESI-TOF): calcd for $C_{21}H_{23}BrN$ [M + H$^+$] 368.1008, found 368.1001.

N-benzyl-1-(m-tolylethynyl)cyclohexanamine (1s)

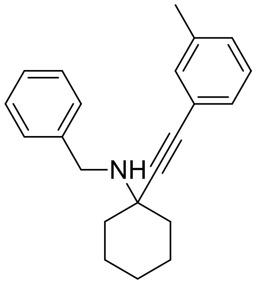

Prepared according to the general procedure **A**. **1s** was obtained as a yellow solid after purification with column chromatography (PE/EtOAc = 9/1) (638 mg, 70%). ^{1}H NMR (400 MHz, $CDCl_3$) δ 7.40 (d, *J* = 7.5 Hz, 2H), 7.27 (m, 6H), 7.12 (d, *J* = 7.6 Hz, 1H), 3.99 (s, 2H), 2.34 (s, 3H), 1.98 (d, *J* = 12.3 Hz, 2H), 1.69 (m, 5H), 1.54 (m, 2H), 1.34 – 1.24 (m, 1H). ^{13}C{^{1}H} NMR (101 MHz, $CDCl_3$) δ 141.2, 138.1, 132.4, 128.9, 128.8, 128.7, 128.6, 128.3, 127.0, 123.6, 93.2, 85.1, 55.5, 48.2, 38.4, 26.1, 23.2, 21.3. HRMS (ESI-TOF): calcd for $C_{22}H_{26}N$ [M + H^{+}] 304.2060, found 304.2066.

N-benzyl-1-(3-phenylprop-1-yn-1-yl)cyclohexanamine (1v)

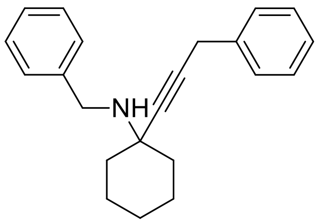

Prepared according to the general procedure **A**. **1v** was obtained as a yellow oil after purification with column chromatography (PE/EtOAc = 9/1) (309 mg, 34%). ^{1}H NMR (400 MHz, $CDCl_3$) δ 7.43 (d, *J* = 7.6 Hz, 2H), 7.40 – 7.31 (m, 6H), 7.30 – 7.22 (m, 2H), 3.95 (s, 2H), 3.74 (s, 2H), 1.94 (d, *J* = 11.6 Hz, 2H), 1.81 – 1.60 (m, 5H), 1.58 – 1.46 (m, 2H), 1.35 – 1.21 (m, 1H). ^{13}C{^{1}H} NMR (101 MHz, $CDCl_3$) δ 141.3, 137.6, 128.6, 128.6, 128.5, 127.9, 126.9, 126.6, 86.5, 81.8, 55.1, 48.1, 38.5, 26.1, 25.3, 23.1. HRMS (ESI-TOF): calcd for $C_{22}H_{26}N$ [M + H^{+}] 304.2060, found 304.2058.

3. General procedure for the synthesis of thiazolidine-2-thiones starting from propargylamines (B)

Propargylamine (0.3 mmol, 1 equiv.), DABCO (5 mg, 0.045 mmol, 15 mol%), and CS_2 (114 mg, 90 µL, 1.5 mmol, 5 equiv.) were added in a 4 mL vial under air. The reaction was stirred at 25 °C for 6h. Then, the reaction mixture was diluted with CH_2Cl_2 and concentrated under reduced pressure. The crude product was isolated by filtration through a silica gel plug with CH_2Cl_2 or by column chromatography (for compounds **2b**, **2g**, **2l**, **2p**, **2q** and **2r**), based on propargylamine conversion. DBU was used for the preparations of compounds **2t** and **2u**.

4. General procedure for the one-pot synthesis of thiazolidine-2-thiones (C)

Ketone (1 mmol, 1 equiv.), amine (1 mmol, 1 equiv.), alkyne (1 mmol, 1 equiv.), and anhydrous $CuCl_2$ (6.7 mg, 0.05 mmol, 5 mol%) were added in a flame dried Schlenk tube. The reaction was stirred at 100 °C for 16h in an oil bath. DABCO (16.8 mg, 0.15 mmol, 15 mol%) and CS_2 (380 mg, 300 µL, 5 mmol, 5 equiv.) were added to the reaction mixture and the reaction was stirred for 3h. The volatiles were removed under reduced pressure and the product was purified by column chromatography.

(*Z*)-1-benzyl-4-benzylidene-3-thia-1-azaspiro[4.5]decane-2-thione (2a)

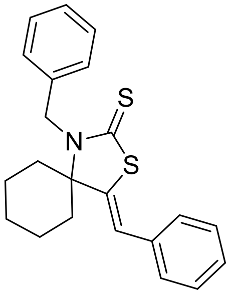

Prepared according to the general procedure **B** and **C**. **2a** was obtained as a beige solid (**B**: 104 mg, 95%, **C**: 186 mg, 51% after purification with column chromatography PE/CH_2Cl_2 = 7/3). ^{1}H NMR (200 MHz, $CDCl_3$) δ 7.48 – 7.17 (m, 10H), 6.91 (s, 1H), 5.17 (s, 2H), 2.10 (d, *J* = 11.1 Hz, 2H), 1.92 – 1.64 (m, 7H), 1.38 – 1.11 (m, 1H). ^{13}C{^{1}H} NMR (50 MHz, $CDCl_3$) δ 193.4, 139.3, 136.3, 135.6, 128.6, 128.6, 128.5, 127.8, 127.2, 126.3, 122.3, 78.2, 48.5, 33.4, 24.4, 22.3. HRMS (ESI-TOF): calcd for $C_{22}H_{24}NS_2$ [M + H$^+$] 366.1345, found 366.1339.

(*Z*)-1-benzyl-4-benzylidene-5.8-dioxaspiro-3-thia-1-azaspiro[4.5]decane-2-thione (2b)

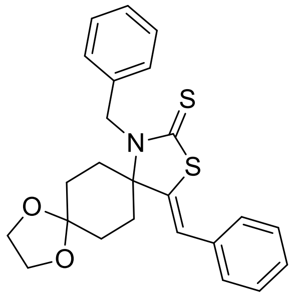

Prepared according to the general procedure **B**. **2b** was obtained as a white solid after purification with column chromatography PE/CH_2Cl_2 = 1/2 (89 mg, 70%). ^{1}H NMR (400 MHz, $CDCl_3$) δ 7.44 – 7.36 (m, 2H), 7.35 – 7.29 (m, 5H), 7.28 – 7.22 (m, 3H), 6.90 (s, 1H), 5.17 (s, 2H), 3.97 (s, 4H), 2.26 (td, *J* = 13.8, 5.0 Hz, 2H), 2.16 – 2.08 (m, 2H), 2.01 (td, *J* = 13.8, 4.7 Hz, 2H), 1.85 – 1.76 (m, 2H). ^{13}C{^{1}H} NMR (101 MHz, $CDCl_3$) δ 193.7, 139.1, 136.4, 135.5, 128.8, 128.7, 128.6, 128.00, 127.3, 126.4, 121.2, 107.0, 77.1, 64.7, 64.6, 48.7, 31.4, 31.2. HRMS (ESI-TOF): calcd for $C_{24}H_{26}NO_2S_2$ [M + H$^+$] 424.1399, found 424.1424.

(*Z*)-4-benzylidene-1-butyl-5.8-dioxaspiro-3-thia-1-azaspiro[4.5]decane-2-thione (2c)

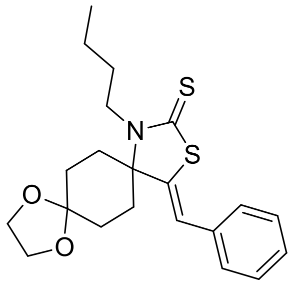

Prepared according to the general procedure **B**. **2c** was obtained as a yellow oil (110 mg, 94%). ^{1}H NMR (400 MHz, $CDCl_3$) δ 7.36 (t, *J* = 7.5 Hz, 2H), 7.27 (d, *J* = 7.9 Hz, 3H), 6.87 (s, 1H), 4.00 (s, 4H), 3.76 – 3.68 (m, 2H), 2.26 (td, *J* = 13.7, 5.0 Hz, 2H), 2.20 – 2.11 (m, 2H), 2.06 (td, *J* = 13.7, 4.6 Hz, 2H), 1.87 (dt, *J* = 14.0, 3.2 Hz, 2H), 1.76 – 1.63 (m, 2H), 1.41 (h, *J* = 7.4 Hz, 2H), 0.97 (t, *J* = 7.3 Hz, 3H). ^{13}C{^{1}H} NMR (101 MHz, $CDCl_3$) δ 191.4, 139.6, 135.7, 128.7, 128.5, 127.9, 120.6, 107.1, 77.0, 64.7, 64.6, 46.2, 31.3, 31.1, 30.5, 20.4, 13.8. HRMS (ESI-TOF): calcd for $C_{21}H_{28}NO_2S_2$ [M + H$^+$] 390.1556, found 390.1553.

(*Z*)-1-benzyl-4-benzylidene-3-thia-1-azaspiro[4.6]undecane-2-thione (2d)

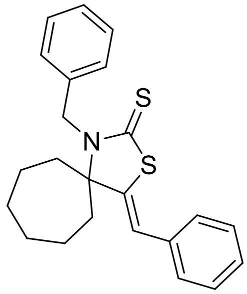

Prepared according to the general procedure **B**. **2d** was obtained as a white solid (106 mg, 93%). ^{1}H NMR (400 MHz, $CDCl_3$) δ 7.42 – 7.24 (m, 10H), 6.59 (s, 1H), 5.24 (s, 2H), 2.20 – 2.00 (m, 4H), 1.72 – 1.51 (m, 8H). ^{13}C{^{1}H} NMR (101 MHz, $CDCl_3$) δ 192.3, 139.3, 136.4, 135.4, 128.7, 128.6, 128.5, 127.7, 127.4, 126.8, 120.2, 83.1, 48.8, 37.8, 31.5, 23.6. HRMS (ESI-TOF): calcd for $C_{23}H_{26}NS_2$ [M + H$^+$] 380.1501, found 380.1506.

(*Z*)-3-benzyl-5-benzylidene-4,4-dimethylthiazolidine-2-thione (2e)

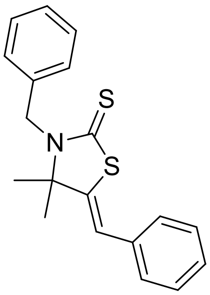

Prepared according to the general procedure **B**. **2e** was obtained as a brown solid (92 mg, 94%). ^{1}H NMR (200 MHz, $CDCl_3$) δ 7.49 – 7.21 (m, 10H), 6.43 (s, 1H), 5.17 (s, 2H), 1.55 (s, 6H). ^{13}C{^{1}H} NMR (50 MHz, $CDCl_3$) δ 192.4, 139.7, 136.6, 135.6, 128.9, 128.8, 128.3, 127.6, 127.6, 127.0, 118.2, 77.2, 48.7, 28.5. HRMS (ESI-TOF): calcd for $C_{19}H_{20}NS_2$ [M + H$^+$] 326.1032, found 326.1032.

(*Z*)-3-benzyl-5-benzylidene-4-ethyl-4-methylthiazolidine-2-thione (2f)

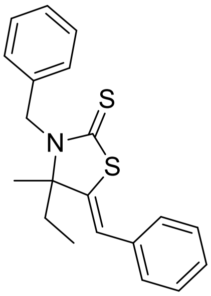

Prepared according to the general procedure **B**. **2e** was obtained as a white solid (94 mg, 92%). ^{1}H NMR (400 MHz, $CDCl_3$) δ 7.45 – 7.37 (m, 4H), 7.35 – 7.30 (m, 4H), 7.29 – 7.23 (m, 2H), 6.34 (s, 1H), 5.60 (d, *J* = 15.7 Hz, 1H), 4.65 (d, *J* = 15.7 Hz, 1H), δ 2.12 (dq, *J* = 14.5, 7.3 Hz, 1H), 1.77 (dq, *J* = 14.7, 7.3 Hz, 1H), 1.45 (s, 3H), 0.84 (t, *J* = 7.3 Hz, 3H). ^{13}C{^{1}H} NMR (50 MHz, $CDCl_3$) δ 193.3, 137.9, 136.5, 135.6, 128.8, 128.6, 128.1, 127.6, 127.5, 127.3, 117.9, 81.2, 48.6, 35.1, 28.7, 7.9. HRMS (ESI-TOF): calcd for $C_{20}H_{22}NS_2$ [M + H$^+$] 340.1188, found 340.1189.

(*Z*)-3-benzyl-5-benzylidene-4-methyl-4-propylthiazolidine-2-thione (2g)

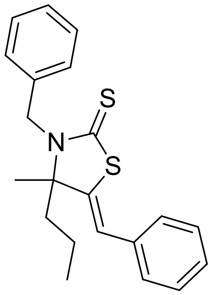

Prepared according to the general procedure **B**. **2g** was obtained as a white solid after purification with column chromatography PE/CH_2Cl_2 = 7/3 (75 mg, 71%). ^{1}H NMR (400 MHz, $CDCl_3$) δ 7.46 – 7.36 (m, 4H), 7.32 (td, *J* = 7.3, 6.6, 1.5 Hz, 4H), 7.29 – 7.23 (m, 2H), 6.34 (s, 1H), 5.46 (d, *J* = 15.6 Hz, 1H), 4.80 (d, *J* = 15.7 Hz, 1H), 1.98 (ddd, *J* = 14.3, 12.2, 4.0 Hz, 1H), 1.68 (ddd, *J* = 14.4, 12.2, 4.7 Hz, 1H), 1.47 (s, 3H), 1.46 – 1.36 (m, 1H), 1.16 – 1.03 (m, 1H), 0.74 (t, *J* = 7.3 Hz, 3H). ^{13}C{^{1}H} NMR (50 MHz, $CDCl_3$) δ 193.1, 138.3, 136.6, 135.7, 128.8, 128.7, 128.6, 128.2, 127.6, 127.5, 127.5, 117.8, 80.7, 48.7, 44.5, 28.9, 16.8, 13.8. HRMS (ESI-TOF): calcd for $C_{21}H_{24}NS_2$ [M + H$^+$] 354.1345, found 354.1348.

(*Z*)-4-benzylidene-1-butyl-3-thia-1-azaspiro[4.5]decane-2-thione (2h)

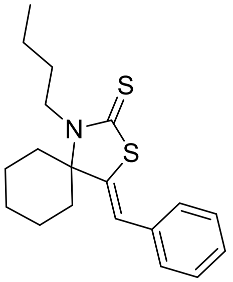

Prepared according to the general procedure **B**. **2h** was obtained as a yellow oil (91 mg, 92%). ^{1}H NMR (400 MHz, $CDCl_3$) δ 7.37 (m, 2H), 7.31 – 7.25 (m, 3H), 6.89 (s, 1H), 3.76 – 3.67 (m, 2H), 2.18 – 2.11 (m, 2H), 1.90 – 1.76 (m, 7H), 1.76 – 1.63 (m, 2H), 1.40 (h, *J* = 7.4 Hz, 2H), 1.35 – 1.24 (m, 1H), 0.97 (t, *J* = 7.4 Hz, 3H). ^{13}C{^{1}H} NMR (101 MHz, $CDCl_3$) δ 191.3, 140.1, 135.9, 128.7, 128.5, 127.7, 121.7, 78.2, 46.2, 33.5, 30.5, 24.5, 22.3, 20.4, 13.9. HRMS (ESI-TOF): calcd for $C_{19}H_{26}NS_2$ [M + H$^+$] 332.1501, found 332.1508.

(*Z*)-4-benzylidene-1-octyl-3-thia-1-azaspiro[4.5]decane-2-thione (2i)

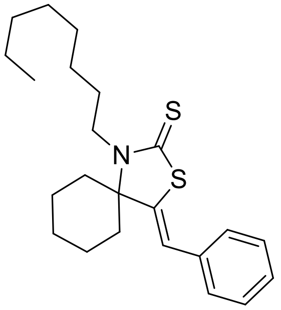

Prepared according to the general procedure **B**. **2i** was obtained as a yellow oil (110 mg, 95%). ^{1}H NMR (400 MHz, $CDCl_3$) δ 7.37 (t, *J* = 7.7 Hz, 2H), 7.29 (d, *J* = 7.5 Hz, 3H), 6.89 (s, 1H), 3.75 – 3.66 (m, 2H), 2.14 (m, 2H), 1.83 (m, 7H), 1.71 (m, 2H), 1.33 (m, 11H), 0.89 (t, *J* = 6.6 Hz, 3H). ^{13}C{^{1}H} NMR (101 MHz, $CDCl_3$) δ 191.2, 140.1, 135.9, 128.7, 128.5, 127.7, 121.6, 78.2, 46.4, 33.5, 31.9, 29.3, 29.2, 28.4, 27.2, 24.5, 22.7, 22.2, 14.2. HRMS (ESI-TOF): calcd for $C_{23}H_{34}NS_2$ [M + H^+] 388.2127, found 388.2134.

(*Z*)-4-benzylidene-1-(4-methoxybenzyl)-3-thia-1-azaspiro[4.5]decane-2-thione (2j)

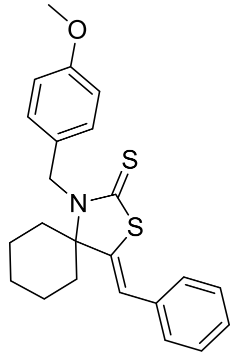

Prepared according to the general procedure **B**. **2j** was obtained as a off-white solid (110 mg, 93%). ^{1}H NMR (200 MHz, $CDCl_3$) δ 7.48 – 7.25 (m, 5H), 7.20 (d, *J* = 8.9 Hz, 2H), 6.92 (s, 1H), 6.85 (d, *J* = 8.8 Hz, 2H), 5.12 (s, 2H), 3.78 (s, 3H), 2.09 (d, *J* = 11.0 Hz, 2H), 1.94 – 1.66 (m, 7H), 1.48 – 1.04 (m, 1H). ^{13}C{^{1}H} NMR (50 MHz, $CDCl_3$) δ 193.3, 158.7, 139.4, 135.6, 128.6, 128.5, 128.3, 127.8, 127.6, 122.3, 114.0, 78.3, 55.3, 48.1, 33.4, 24.4, 22.3. HRMS (ESI-TOF): calcd for $C_{23}H_{26}NOS_2$ [M + H^+] 396.1450, found 396.1452.

(*Z*)-4-benzylidene-1-(4-fluorobenzyl)-3-thia-1-azaspiro[4.5]decane-2-thione (2k)

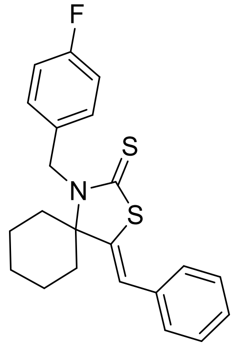

Prepared according to the general procedure **B**. **2k** was obtained as a beige solid (101 mg, 88%). ^{1}H NMR (400 MHz, $CDCl_3$) δ 7.40 (t, *J* = 7.6 Hz, 2H), 7.34 – 7.27 (m, 3H), 7.23 (dd, *J* = 8.4, 5.3 Hz, 2H), 7.00 (t, *J* = 8.6 Hz, 2H), 6.92 (s, 1H), 5.13 (s, 2H), 2.09 (d, *J* = 11.4 Hz, 2H), 1.89 – 1.69 (m, 7H), 1.39 – 1.15 (m, 1H). ^{13}C{^{1}H} NMR (101 MHz, $CDCl_3$) δ 193.9, 162.1 (d, *J* = 245.7 Hz), 139.4, 135.7, 132.3 (d, *J* = 3.1 Hz), 128.8, 128.6, 128.2 (d, *J* = 8.0 Hz), 128.0, 122.5, 115.6 (d, *J* = 21.5 Hz), 78.3, 48.0, 33.6, 24.5, 22.4. ^{19}F NMR (376 MHz, $CDCl_3$) δ -115.3. HRMS (ESI-TOF): calcd for $C_{22}H_{23}FNS_2$ [M + H^+] 384.1250, found 384.1249.

(*Z*)-4-benzylidene-1-(2-(2-(2-methoxyethoxy)ethoxy)ethyl)-3-thia-1-azaspiro[4.5]decane-2-thione (2l)

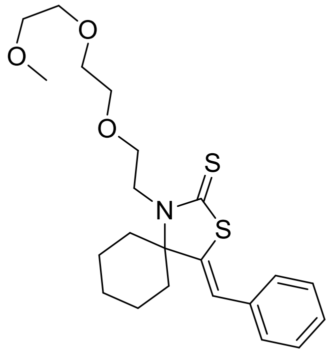

Prepared according to the general procedure **B**. **2l** was obtained as a yellow oil after purification with column chromatography PE/EtOAc = 8/2 (109 mg, 86%). ^{1}H NMR (400 MHz, $CDCl_3$) δ 7.37 (t, *J* = 7.6 Hz, 2H), 7.28 (d, *J* = 7.6 Hz, 3H), 6.91 (s, 1H), 3.98 (t, *J* = 6.6 Hz, 2H), 3.76 (t, *J* = 6.6 Hz, 2H), 3.69 – 3.61 (m, 6H), 3.55 (t, *J* = 4.1 Hz, 2H), 3.37 (s, 3H), 2.13 (d, *J* = 13.5 Hz, 2H), 1.95 – 1.72 (m, 7H), 1.32 – 1.26 (m, 1H). ^{13}C{^{1}H} NMR (126 MHz, $CDCl_3$) δ 192.8, 139.8, 135.9, 128.7, 128.5, 127.8, 122.0, 78.2, 72.0, 70.7, 70.66, 70.64, 67.6, 59.2, 45.6, 33.3, 24.4, 22.3. HRMS (ESI-TOF): calcd for $C_{22}H_{32}NO_3S_2$ [M + H$^+$] 422.1818, found 422.1828.

(*Z*)-1-benzyl-4-(4-methoxybenzylidene)-3-thia-1-azaspiro[4.5]decane-2-thione (2n)

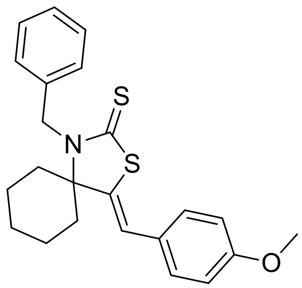

Prepared according to the general procedure **B**. **2n** was obtained as a beige solid (112 mg, 95%). ^{1}H NMR (200 MHz, $CDCl_3$) δ 7.39 – 7.14 (m, 7H), 6.92 (d, *J* = 8.8 Hz, 2H), 6.85 (s, 1H), 5.16 (s, 2H), 3.84 (s, 3H), 2.09 (d, *J* = 10.4 Hz, 2H), 1.76 (d, *J* = 4.7 Hz, 7H), 1.41 – 1.11 (m, 1H). ^{13}C{^{1}H} NMR (50 MHz, $CDCl_3$) δ 193.7, 159.1, 136.9, 136.4, 129.9, 128.6, 128.3, 127.2, 126.4, 122.0, 114.1, 78.2, 55.4, 48.60, 33.5, 24.5, 22.4. HRMS (ESI-TOF): calcd for $C_{23}H_{26}NOS_2$ [M + H$^+$] 396.1450, found 396.1455.

(*Z*)-1-benzyl-4-(4-methylbenzylidene)-3-thia-1-azaspiro[4.5]decane-2-thione (2o)

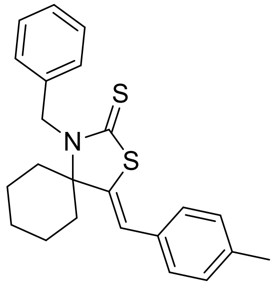

Prepared according to the general procedure **B**. **2o** was obtained as a yellow solid (107 mg, 94%). ^{1}H NMR (400 MHz, $CDCl_3$) δ 7.35 – 7.27 (m, 2H), 7.25 – 7.19 (m, 7H), 6.87 (s, 1H), 5.17 (s, 2H), 2.37 (s, 3H), 2.09 (d, *J* = 12.4 Hz, 2H), 1.88 – 1.69 (m, 7H), 1.33 – 1.19 (m, 1H). ^{13}C{^{1}H} NMR (50 MHz, $CDCl_3$) δ 193.6, 138.2, 137.7, 136.4, 132.8, 129.3, 128.6, 128.4, 127.2, 126.3, 122.2, 78.2, 48.5, 33.4, 24.4, 22.3, 21.3. HRMS (ESI-TOF): calcd for $C_{23}H_{26}NS_2$ [M + H$^+$] 380.1501, found 380.1506.

(*Z*)-1-benzyl-4-(4-chlorobenzylidene)-3-thia-1-azaspiro[4.5]decane-2-thione (2p)

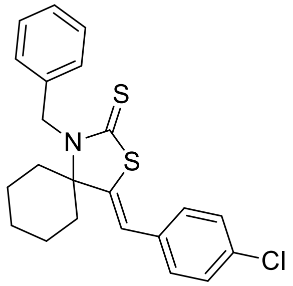

Prepared according to the general procedure **B** and **C**. **2p** was obtained as a white solid after purification with column chromatography (PE/CH_2Cl_2 = 7/3) (**B**: 86 mg, 72%, **C**: 196 mg, 49%). ^{1}H NMR (400 MHz, $CDCl_3$) δ 7.40 – 7.28 (m, 4H), 7.27 – 7.22 (m, 5H), 6.84 (s, 1H), 5.16 (s, 2H), 2.08 (d, *J* = 13.1 Hz, 2H), 1.88 – 1.64 (m, 7H), 1.30 – 1.22 (m, 1H). ^{13}C{^{1}H} NMR (50 MHz, $CDCl_3$) δ 192.8, 140.3, 136.2, 134.1, 133.4, 129.8, 128.8, 128.6, 127.3, 126.3, 120.9, 78.3, 48.6, 33.4, 24.3, 22.2. HRMS (ESI-TOF): calcd for $C_{22}H_{23}ClNS_2$ [M + H$^+$] 400.0955, found 400.0983.

(*Z*)-1-benzyl-4-(4-(trifluoromethyl)benzylidene)-3-thia-1-azaspiro[4.5]decane-2-thione (2q)

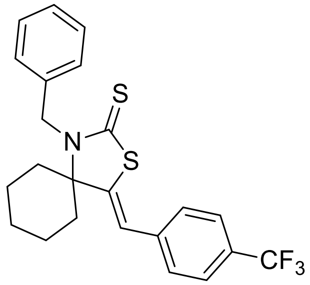

Prepared according to the general procedure **B**. **2q** was obtained as a white solid after purification with column chromatography (PE/CH_2Cl_2 = 7/3) (85 mg, 66%). ^{1}H NMR (400 MHz, $CDCl_3$) δ 7.65 (d, *J* = 8.0 Hz, 2H), 7.42 (d, *J* = 8.0 Hz, 2H), 7.32 (t, *J* = 7.4 Hz, 2H), 7.28 – 7.21 (m, 3H), 6.92 (s, 1H), 5.17 (s, 2H), 2.10 (d, *J* = 13.3 Hz, 2H), 1.92 – 1.66 (m, 7H), 1.34 – 1.22 (m, 1H). ^{13}C{^{1}H} NMR (101 MHz, $CDCl_3$) δ 192.7, 142.8, 139.3, 136.3, 129.5 (q, *J* = 32.6 Hz), 128.8, 128.8, 127.5, 126.5, 125.6 (q, *J* = 3.8 Hz), 124.0 (q, *J* = 272.5 Hz), 120.6, 78.5, 48.8, 33.6, 24.4, 22.4. ^{19}F NMR (376 MHz, $CDCl_3$) δ -62.6. HRMS (ESI-TOF): calcd for $C_{23}H_{23}F_3NS_2$ [M + H$^+$] 434.1219, found 434.1209.

(*Z*)-1-benzyl-4-(2-bromobenzylidene)-3-thia-1-azaspiro[4.5]decane-2-thione (2r)

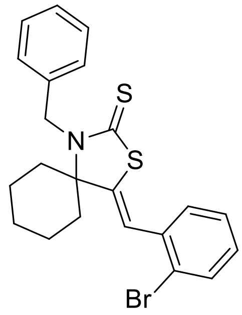

Prepared according to the general procedure **B**. **2r** was obtained as a yellow oil after purification with column chromatography (PE/CH_2Cl_2 = 7/3) (99 mg, 74%). ^{1}H NMR (400 MHz, $CDCl_3$) δ 7.61 (d, *J* = 8.0 Hz, 1H), 7.44 (d, *J* = 7.6 Hz, 1H), 7.36 – 7.30 (m, 3H), 7.26 (m, 3H), 7.16 (t, *J* = 7.6 Hz, 1H), 7.03 (s, 1H), 5.16 (s, 2H), 2.11 (d, *J* = 4.5 Hz, 2H), 1.90 – 1.76 (m, 7H), 1.33 – 1.21 (m, 1H). ^{13}C{^{1}H} NMR (101 MHz, $CDCl_3$) δ 193.1, 142.3, 136.4, 136.0, 133.0, 129.5, 129.3, 128.8, 127.7, 127.4, 126.5, 124.3, 121.4, 78.3, 48.8, 33.7, 24.4, 22.1. HRMS (ESI-TOF): calcd for $C_{22}H_{23}BrNS_2$ [M + H$^+$] 444.0450, found 444.0461.

(*Z*)-1-benzyl-4-(3-methylbenzylidene)-3-thia-1-azaspiro[4.5]decane-2-thione (2s)

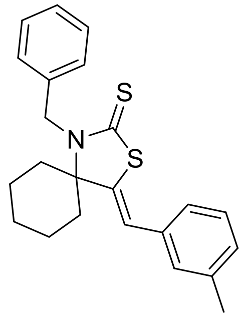

Prepared according to the general procedure **B**. **2s** was obtained as a yellow oil (103 mg, 90%). ^{1}H NMR (400 MHz, $CDCl_3$) δ 7.37 – 7.22 (m, 6H), 7.19 – 7.10 (m, 3H), 6.92 (s, 1H), 5.20 (s, 2H), 2.41 (s, 3H), 2.11 (d, *J* = 12.7 Hz, 2H), 1.91 – 1.68 (m, 7H), 1.36 – 1.22 (m, 1H). ^{13}C{^{1}H} NMR (101 MHz, $CDCl_3$) δ 193.6, 139.1, 138.3, 136.4, 135.6, 129.2, 128.6, 128.5, 127.2, 126.4, 125.6, 122.4, 78.2, 48.5, 33.5, 24.4, 22.3, 21.5. HRMS (ESI-TOF): calcd for $C_{23}H_{26}NS_2$ [M + H$^+$] 380.1501, found 380.1505.

(*Z*)-1-benzyl-4-butylidene-3-thia-1-azaspiro[4.5]decane-2-thione (2t)

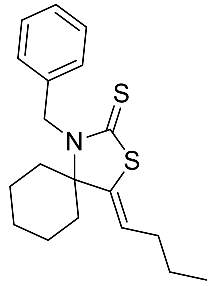

Prepared according to the general procedure **B**. **2t** was obtained as a beige solid (93 mg, 94%). ^{1}H NMR (400 MHz, $CDCl_3$) δ 7.35 – 7.27 (m, 2H), 7.26 – 7.20 (m, 3H), 5.84 (t, *J* = 7.1 Hz, 1H), 5.11 (s, 2H), 2.05 (q, *J* = 7.2 Hz, 2H), 1.93 (d, *J* = 13.3 Hz, 2H), 1.78 – 1.54 (m, 7H), 1.49 (h, *J* = 7.3 Hz, 2H), 1.28 – 1.11 (m, 1H), 0.95 (t, *J* = 7.3 Hz, 3H). ^{13}C{^{1}H} NMR (101 MHz, $CDCl_3$) δ 193.6, 138.3, 136.6, 128.6, 127.1, 126.4, 123.1, 76.6, 48.6, 33.6, 33.3, 24.4, 22.3, 22.1, 13.7. HRMS (ESI-TOF): calcd for $C_{19}H_{26}NS_2$ [M + H$^+$] 332.1501, found 332.1499.

(*Z*)-1-benzyl-4-heptylidene-3-thia-1-azaspiro[4.5]decane-2-thione (2u)

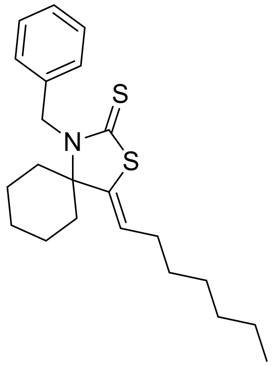

Prepared according to the general procedure **B**. **2u** was obtained as a beige solid (98 mg, 87%). ^{1}H NMR (400 MHz, $CDCl_3$) δ 7.34 – 7.28 (m, 2H), 7.27 – 7.20 (m, 3H), 5.84 (t, *J* = 7.1 Hz, 1H), 5.11 (s, 2H), 2.06 (q, *J* = 7.3 Hz, 2H), 1.93 (d, *J* = 13.5 Hz, 2H), 1.78 – 1.52 (m, 6H), 1.44 (quint, *J* = 7.0 Hz, 2H), 1.38 – 1.24 (m, 7H), 1.23 – 1.11 (m, 1H), 0.90 (t, *J* = 7.2 Hz, 3H). ^{13}C{^{1}H} NMR (101 MHz, $CDCl_3$) δ 193.8, 138.1, 136.7, 128.7, 127.2, 126.5, 123.4, 76.7, 48.7, 33.4, 31.8, 31.7, 29.0, 28.9, 24.5, 22.7, 22.2, 14.2. HRMS (ESI-TOF): calcd for $C_{22}H_{32}$NS2 [M + H$^+$] 374.1971, found 374.1974.

(*Z*)-1-benzyl-4-(2-phenylethylidene)-3-thia-1-azaspiro[4.5]decane-2-thione (2v)

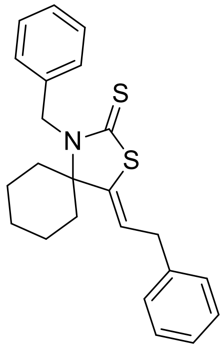

Prepared according to the general procedure **B**. **2v** was obtained as a beige solid (105 mg, 92%). ^{1}H NMR (400 MHz, $CDCl_3$) δ 7.32 (t, *J* = 7.4 Hz, 4H), 7.26 – 7.17 (m, 6H), 6.04 (t, *J* = 7.1 Hz, 1H), 5.13 (s, 2H), 3.42 (d, *J* = 7.1 Hz, 2H), 1.98 (d, *J* = 13.2 Hz, 2H), 1.77 – 1.50 (m, 7H), 1.17 (qt, *J* = 13.7, 4.3 Hz, 1H). ^{13}C{^{1}H} NMR (101 MHz, $CDCl_3$) δ 193.3, 139.7, 139.1, 136.6, 128.9, 128.7, 128.5, 127.3, 126.7, 126.5, 121.7, 76.9, 48.8, 37.8, 33.5, 24.4, 22.2. HRMS (ESI-TOF): calcd for $C_{23}H_{25}NNaS_2$ [M + Na$^+$] 402.1321, found 402.1323.

(*Z*)-1-butyl-4-(4-methoxybenzylidene)-3-thia-1-azaspiro[4.5]decane-2-thione (2w)

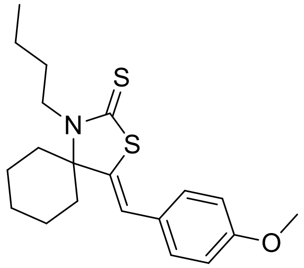

Prepared according to the general procedure **C**. **2w** was obtained as a yellow oil after purification with column chromatography (PE/CH_2Cl_2 = 7/3) (203 mg, 56%). ^{1}H NMR (400 MHz, $CDCl_3$) δ 7.22 (d, *J* = 8.7 Hz, 2H), 6.90 (d, *J* = 8.8 Hz, 2H), 6.83 (s, 1H), 3.83 (s, 3H), 3.76 – 3.67 (m, 2H), 2.14 (d, *J* = 7.8 Hz, 2H), 1.90 – 1.76 (m, 7H), 1.75 – 1.63 (m, 2H), 1.40 (h, *J* = 7.5 Hz, 2H), 1.33 – 1.27 (m, 1H), 0.97 (t, *J* = 7.3 Hz, 3H). ^{13}C{^{1}H} NMR (101 MHz, $CDCl_3$) δ 191.3, 159.0, 137.5, 129.8, 128.5, 121.3, 114.0, 78.1, 55.4, 46.0, 33.4, 30.4, 24.4, 22.2, 20.4, 13.8. HRMS (ESI-TOF): calcd for $C_{20}H_{28}NOS_2$ [M + H$^+$] 362.1607, found 362.1610.

(*Z*)-1-(5-hydroxypentyl)-4-(4-methoxybenzylidene)-3-thia-1-azaspiro[4.5]decane-2-thione (2x)

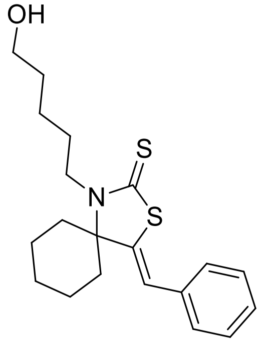

Prepared according to the general procedure **C**. **2x** was obtained as an orange oil after purification with column chromatography (PE/EtOAc = 1/1) (195 mg, 54%) after purification with column chromatography (EtOAc/MeOH = 9/1). ^{1}H NMR (400 MHz, $CDCl_3$) δ 7.37 (t, *J* = 7.6 Hz, 2H), 7.28 (d, *J* = 7.7 Hz, 3H), 6.89 (s, 1H), 3.75 – 3.69 (m, 2H), 3.67 (t, *J* = 6.4 Hz, 2H), 2.15 (d, *J* = 7.0 Hz, 2H), 1.91 – 1.70 (m, 9H), 1.67 – 1.57 (m, 2H), 1.51 – 1.43 (m, 2H), 1.36 – 1.24 (m, 1H). ^{13}C{^{1}H} NMR (101 MHz, $CDCl_3$) δ 191.4, 139.9, 135.9, 128.7, 128.5, 127.8, 121.8, 78.2, 62.7, 46.2, 33.4, 32.2, 28.1, 24.5, 23.4, 22.2. HRMS (ESI-TOF): calcd for $C_{20}H_{28}NOS_2$ [M + H$^+$] 362.1607, found 362.1607.

(Z)-4-benzylidene-1-(2-morpholinoethyl)-3-thia-1-azaspiro[4.5]decane-2-thione (2y)

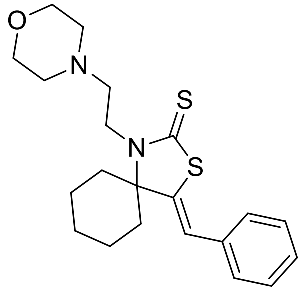

Prepared according to the general procedure **C**. **2y** was obtained as a beige solid (182 mg, 47%) after purification with column chromatography (EtOAc). ^{1}H NMR (400 MHz, $CDCl_3$) δ 7.41 – 7.33 (t, *J* = 7.1, Hz, 2H), 7.28 (d, *J* = 7.2 Hz, 3H), 6.90 (s, 1H), 3.94 (t, *J* = 7.6 Hz, 2H), 3.73 (t, *J* = 4.6 Hz, 4H), 2.66 (t, *J*

= 8.0 Hz, 2H), 2.60 (t, *J* = 4.7 Hz, 4H), 2.15 (d, *J* = 12.7 Hz, 2H), 1.91 – 1.79 (m, 7H), 1.35 – 1.23 (m, 1H). $^{13}C\{^1H\}$ NMR (101 MHz, $CDCl_3$) δ 192.4, 139.7, 135.8, 128.7, 128.5, 127.9, 122.1, 78.1, 67.0, 55.7, 54.1, 43.4, 33.4, 24.4, 22.3. HRMS (ESI-TOF): calcd for $C_{21}H_{29}N_2OS_2$ [M + H^+] 389.1716, found 389.1717.

(Z)-4-benzylidene-1-(3-(dimethylamino)propyl)-3-thia-1-azaspiro[4.5]decane-2-thione (2z)

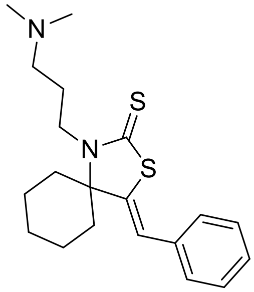

Prepared according to the general procedure **C**. **2z** was obtained as a brown solid (220 mg, 61%) after purification with column chromatography (CH_2Cl_2/MeOH = 9.5/0.5). ^1H NMR (400 MHz, $CDCl_3$) δ 7.37 (t, *J* = 7.6 Hz, 2H), 7.28 (d, *J* = 7.9 Hz, 3H), 6.89 (s, 1H), 3.83 – 3.75 (m, 2H), 2.39 (t, *J* = 6.9 Hz, 2H), 2.26 (s, 6H), 2.15 (d, *J* = 12.2 Hz, 2H), 1.98 – 1.76 (m, 9H), 1.40 – 1.27 (m, 1H). $^{13}C\{^1H\}$ NMR (101 MHz, $CDCl_3$) δ 191.6, 140.1, 135.9, 128.7, 128.5, 127.8, 121.8, 78.3, 57.2, 45.6, 44.7, 33.4, 26.3, 24.4, 22.3. HRMS (ESI-TOF): calcd for $C_{20}H_{29}N_2S_2$ [M + H^+] 361.1767, found 361.1769.

(*Z*)-1-benzyl-4-benzylidene-3-thia-1-azaspiro[4.5]decan-2-one (3)

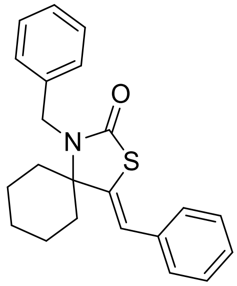

3 was obtained as a white solid after purification with column chromatography (PE/CH_2Cl_2 = 7/3) (11 mg, 10%). ^1H NMR (400 MHz, $CDCl_3$) δ 7.43 – 7.28 (m, 10H), 6.98 (s, 1H), 4.62 (s, 2H), 2.04 (d, *J* = 9.1 Hz, 2H), 1.83 – 1.70 (m, 7H), 1.32 – 1.18 (m, 1H). $^{13}C\{^1H\}$ NMR (101 MHz, $CDCl_3$) δ 169.2, 139.0, 138.1, 136.1, 128.7, 128.7, 128.6, 127.7, 127.3, 126.7, 123.3, 70.0, 45.0, 33.7, 24.7, 22.8. HRMS (ESI-TOF): calcd for $C_{22}H_{24}NOS$ [M + H^+] 350.1573, found 350.1579.

5. Competition Experiments

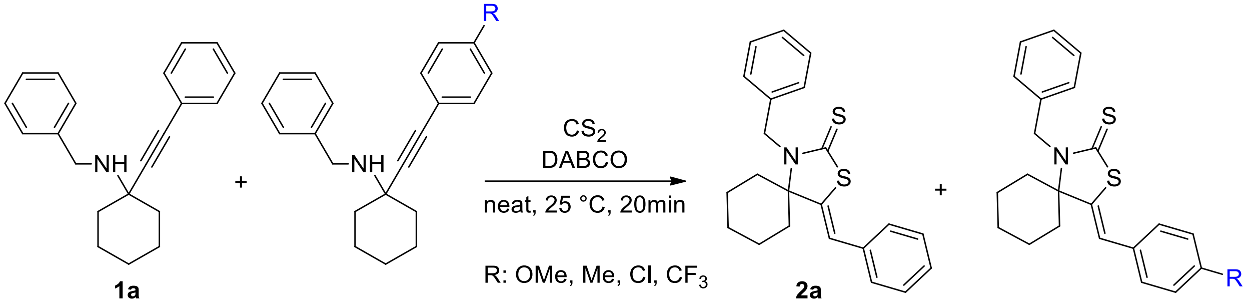

Scheme S1: Competition experiments between propargylamine **1a** and *p*-substituted propargylamines.

Propargylamine **1a** (43.4 mg, 0.15 mmol), *para*-substituted propargylamine (0.15 mmol), DABCO (5 mg, 0.045 mmol), and CS_2 (114 mg, 90 µL, 1.5 mmol) were added in a 4 mL vial under air. The reaction was stirred at 25 °C for 20 min. Then, the reaction mixture was diluted with CH_2Cl_2 and concentrated under reduced pressure. The relative ratios of the products were determined by ^{1}H-NMR of the crude mixtures. In the case of OMe vs H, the product mixture was separated from the crude reaction mixture due to peak overlap using column chromatography (PE/CH_2Cl_2 = 1/1). (Figure S1).

k_{OMe}/k_H = 1.39/1

k_{Me}/k_H = 1.14/1

k_{Cl}/k_H = 0.66/1

k_{CF3}/k_H = 0.39/1

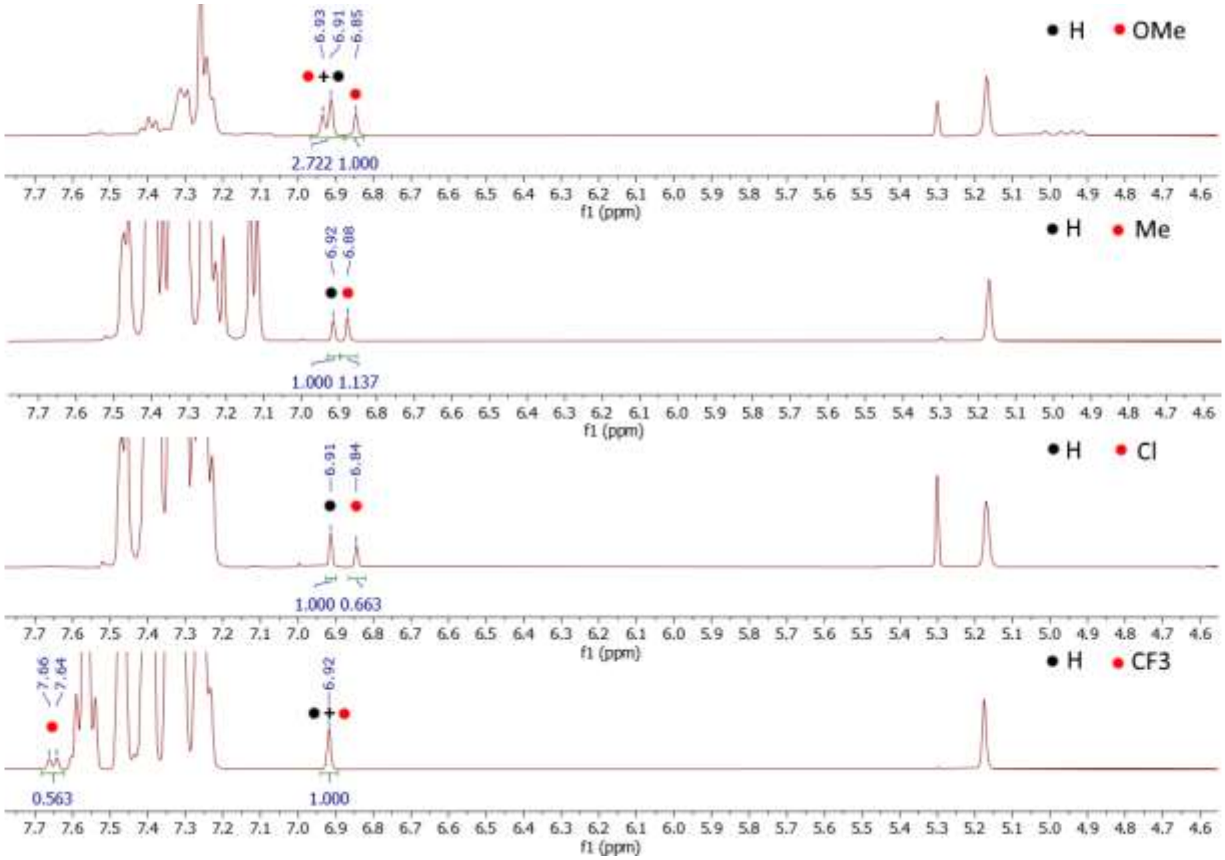

Figure S1: ^{1}H-NMR (400 MHz, $CDCl_3$) spectra of the reaction mixtures obtained from the competition experiments.

6. DFT Studies

6.1. Reaction mechanism

Figure S2 compares the Gibbs free energy profiles of two pathways, which differ in the CS_2 activation step: pathway 1 (black) starts with CS_2 addition to α-tertiary propargylamine, and pathway 2 (red) is initiated with CS_2 addition to DABCO followed by CS_2 transfer to propargylamine. Pathway 1 is identical to the stepwise mechanism presented in Figure 4 of the main text. In pathway 2, the first N-C bond formation between CS_2 and DABCO (TS4) is found to involve a lower barrier than that between CS_2 and propargylamine (TS1) (0.53 vs. 0.69 eV). We explored the possibility of a concerted CS_2 transfer from DABCO to propargylamine and found that it is not likely to occur due to the significantly high activation energy ($\Delta G^{\ddagger}$ = 1.10 eV). Therefore, the CS_2 transfer occurs in a stepwise fashion via N-C dissociation between CS_2 and DABCO (TS5) followed by CS_2 addition to propargylamine (TS6). Here, TS6 is essentially identical to TS1 except from the presence of DABCO in the molecular structure of TS6, leading to the similar activation energy (TS1, 0.69 eV vs. TS6, 0.63 eV). Overall, pathway 2 does not significantly contribute to the overall reaction kinetics.

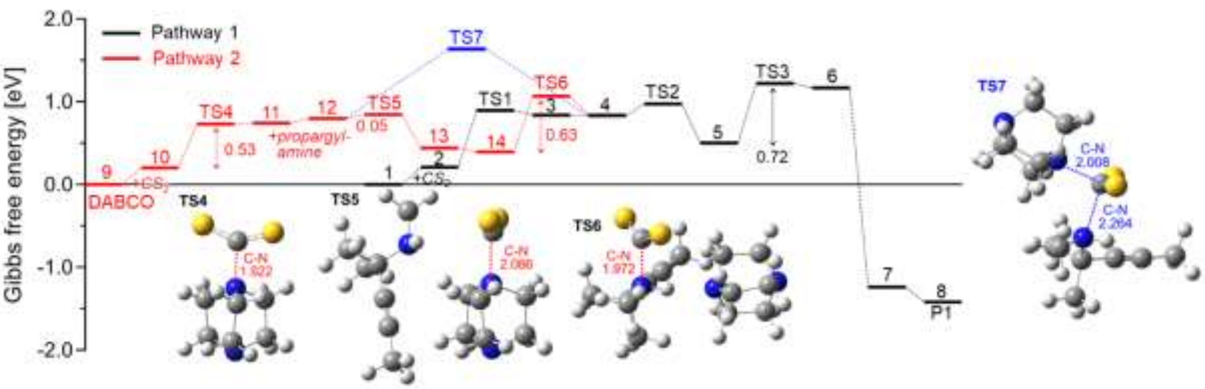

Figure S2: Gibbs free energy profiles of DABCO-catalyzed reactions between α-tertiary propargylamine and CS_2 to thiazolidine-2-thiones (P1), determined using DFT calculations at 25 °C and 1 atm. Methyl groups ($-CH_3$) are used as substituents. Black lines represent the reaction mechanism starting with CS_2 addition to propargylamine (pathway 1; stepwise mechanism of Figure 4 of the main text) and red lines represent the reaction mechanism starting with CS_2 addition to DABCO (pathway 2). Molecular structures of transition states (TS) in the second mechanism are shown with the interatomic distances associated with the imaginary modes. Atoms are color-coded based on the atom type (white = H, gray = C, blue = N, and yellow = S).

6.2. Electronic effects of *para*-substituents on TS1

Figure S3 shows the energy change of the highest occupied molecular orbital (HOMO) and lowest unoccupied molecular orbital (LUMO) of state 2 (reactant of TS1) by the presence of phenyl substituents on the *para* position of the phenylacetylene moiety of α-tertiary propargylamine **1a**. The LUMO energy remains at the same level, since it is a π* orbital in CS_2, which is independent of the presence of the substituents on tertiary propargylamine (Figure S4). On the other hand, the HOMO localizes on the tertiary propargylamine and therefore the substituents affect the molecular orbitals. The electron-donating methoxy group makes the HOMO mostly distributed in the phenylacetylene moiety whereas the electron-withdrawing trifluoromethyl group makes the HOMO further diffused around the propargylamine N. Hence, the presence of the methoxy and trifluoromethyl groups leads to the smaller and greater HOMO-LUMO energy gap, respectively. The electronic effects of the substituents on the HOMO-LUMO energy gap rationalize the change of the activation energy of TS1 shown in Figure 5a.

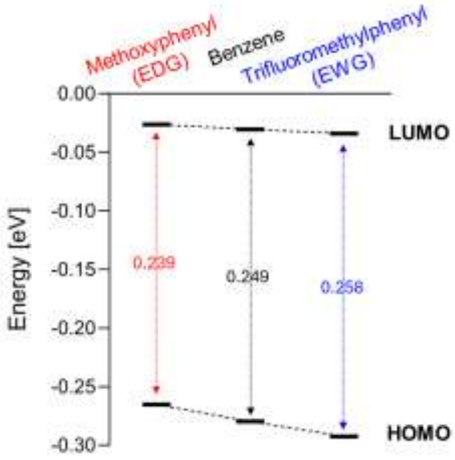

Figure S3: Highest occupied molecular orbital (HOMO) and lowest unoccupied molecular energy (LUMO) energies of the state 2 of tertiary propargylamine **1a** and their derivatives with methoxy and trifluoromethyl groups on the *para* position of the phenylacetylene moiety.

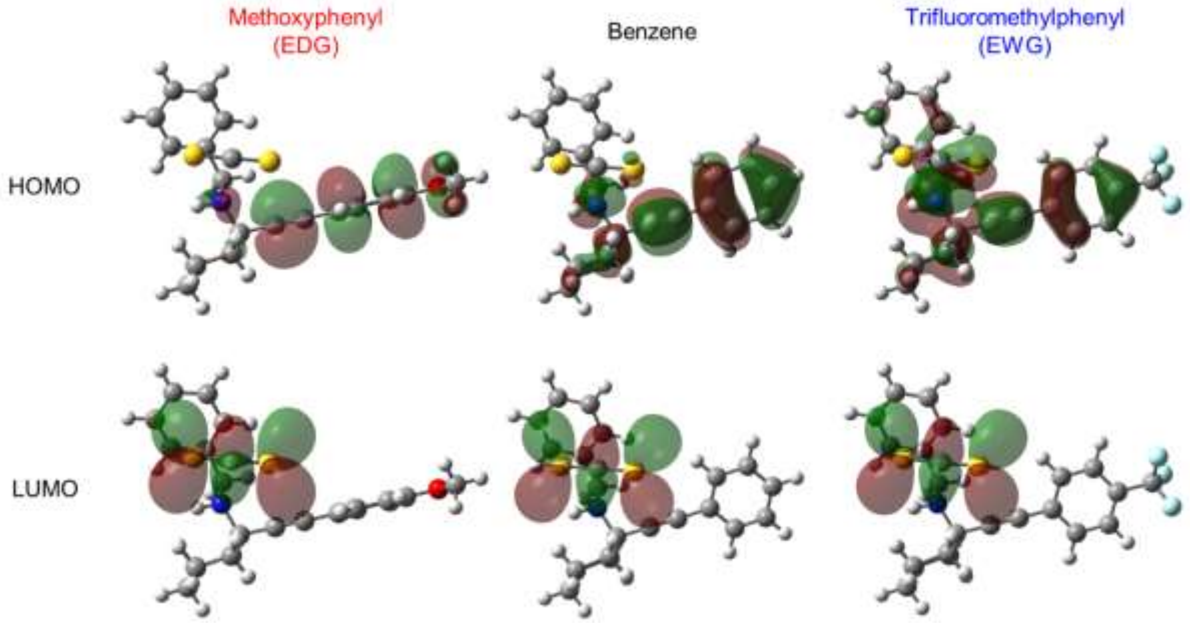

Figure S4: Molecular structures and visualized HOMO and LUMO of the species in Figure S3 (isovalue = 0.02).

6.3. Steric hindrance of DABCO on alkyl-bearing species

Figure S5 shows the change in two interatomic distances from state 5 to TS3: S^--H^+ and C-H^+, where S^- and C form a covalent bond upon TS3, and H^+ belongs to a protonated catalyst (DABCO or DBU). The increase in the S^--H^+ distance and the decrease in the C-H^+ distance indicate that TS3 involves a change in the relative position and orientation of the protonated catalyst in such a way that it moves away from S^- and closer to C relative to state 5. This change is attributed to the electron transfer from S^- to C upon the intramolecular cyclization. The bulky DABCO molecule gives rise to steric hindrance with the propyl

substituent in TS3, leading to a significant distortion of the molecular structure (Figure 6c). On the other hand, DBU has a planar structure, with much milder steric effects and structural distortion (Figure 6d) in TS3.

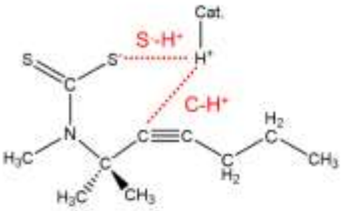

S^--H^+ distance [Å]	DABCO	DBU
State 5	1.974	2.603
TS3	2.429	2.846
Δ	+0.455	+0.243

C-H^+ distance [Å]	DABCO	DBU
Reactant	3.764	2.785
TS	2.138	2.424
Δ	-1.626	-0.361

Figure S5: S^--H^+ and C-H^+ interatomic distances in state 5 and TS3 of DABCO- and DBU-catalysis of propyl-bearing thiazolidine-2-thiones. "Cat." refers to DABCO or DBU.

To provide a better understanding of the interactions between a protonated catalyst and a substituent at the terminal position of the alkyne moiety of the dithiocarbamate anion, we repeated the calculations of Figure 6 of the main text by replacing the propyl groups with benzyl groups, as shown in Figure S6. As described above, TS3 involves changes in the relative position and orientation of the protonated catalyst species (Figure S7), and a bulkier DABCO catalyst provides more significant steric hindrance, as evidenced by the distortion of the benzyl moiety relative to a CS_2-propargylamine nitrogen moiety (Figure S6c-d). However, the benzene ring provides stabilization effects to DABCO, canceling out the steric hindrance in TS3, which leads to the similar activation energy with that of the DBU case (0.50 vs. 0.53 eV). This explains the considerable different yields of thiazolidine-2-thiones bearing sp^3-carbon atom substituted alkyne moieties with DABCO: alkane chains with a low yield (**2t** and **2u**; >10%) vs. a benzyl group with a high yield (**2v**; 92%).

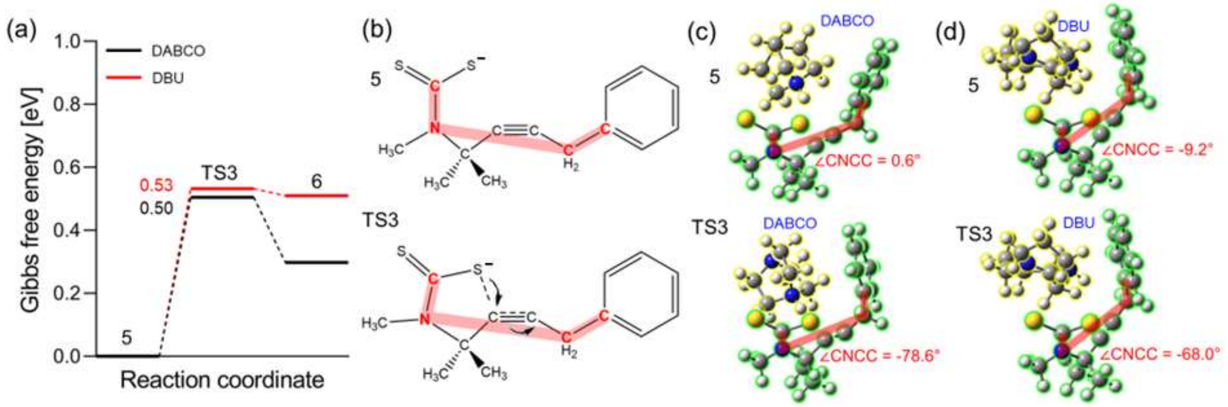

Figure S6: (a) Gibbs free energy profiles of TS3 on the reaction mechanisms of DABCO (black) and DBU (red) catalysis of α-tertiary propargylamine and CS_2 to thiazolidine-2-thiones bearing a benzyl group at the terminal position of the alkyne moiety. Methyl groups are used in place of all the other substituents for computational simplicity. (b) Dithiocarbamate anion structures in state 5 and TS3 with a characteristic dihedral angle marked in red. Optimized molecular structures of state 5 and TS3 in the presence of (c) DABCO and (d) DBU, where the protonated catalysts and dithiocarbamate anions are highlighted in yellow and green, respectively.

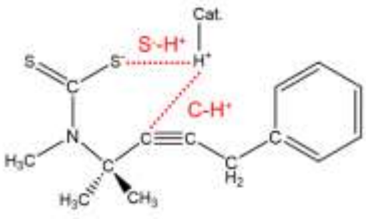

S⁻-H⁺ distance [Å]	DABCO	DBU
State 5	2.298	2.472
TS3	2.601	2.774
Δ	+0.303	+0.302

C-H⁺ distance [Å]	DABCO	DBU
Reactant	2.764	3.691
TS	2.224	3.216
Δ	-0.540	-0.475

Figure S7: S^--H^+ and C-H^+ interatomic distances in state 5 and TS3 of DABCO- and DBU-catalysis of benzyl-bearing thiazolidine-2-thiones. “Cat.” refers to DABCO or DBU.

7. Single Crystal X-ray Crystallography

Colorless crystals of compound **2a** were grown via vapor diffusion of *n*-pentane into a solution of **2a** in CH_2Cl_2. Single Crystal X-ray diffraction data for compound **2a** were collected using Mo/Kα at 100 K to a resolution of 0.75 Å, on a Bruker D8-Venture dual source SC-XRD equipped with IμS Diamond Cu/Kα and Mo/Kα X-ray sources, a 4-circle kappa goniometer and Photon-III area detector, using φ and ω scans to fill the Ewald sphere. Data collection strategy, acquisition, integration and scaling were handled by the APEX4 software and packages within. Non-H atoms were refined anisotropically, using weighted full-matrix least-squares on F^2 while H-atoms were added using the riding model with SHELXL default parameters. Structure solution (ShelXT, reference: Sheldrick, G.M. (2015). Acta Cryst. A71, 3-8), modelling and refinement (ShelXL, reference: Sheldrick, G.M. (2008). Acta Cryst. A64, 112-122.) were achieved *via* their implementation into the Olex2-1.5 (reference: Dolomanov, O.V., Bourhis, L.J., Gildea, R.J, Howard, J.A.K. & Puschmann, H.(2009), J. Appl. Cryst. 42, 339-341) software suite. Data collection, processing and final refinement details are given in the following table.

Compound	**2a**
Colour, habit	Colourless/Plate
Size/mm	0.37x0.29x0.045
Empirical formula	$C_{22}H_{23}NS_2$
FW	365.53
Crystal system	Monoclinic
Space group	$P2_1/c$
a/Å	9.1703(3)
b/Å	9.9994(3)
c/Å	20.3921(7)
a/°	90
b/°	94.173(2)
γ/°	90
V/Å^3	1864.95(10)
Z	4
μ/mm^{-1}	0.290

T/K	100
θ min/full/max (˚)	2.003/25.242/28.291
Completeness to θfull/max (%)	100%/99.9%
Reflections Total/ Independent	4624/4224
Parameters/restraints	226/0
R_{int}	0.0451
Final *R*1, *wR*2	0.0305, 0.0807
Goof	1.036
Largest peak, hole / e.Å^{-3}	0.4/-0.2
ρ_{calc}/g cm^{-3}	1.302
CCDC Reference	2388912

8. NMR Spectra

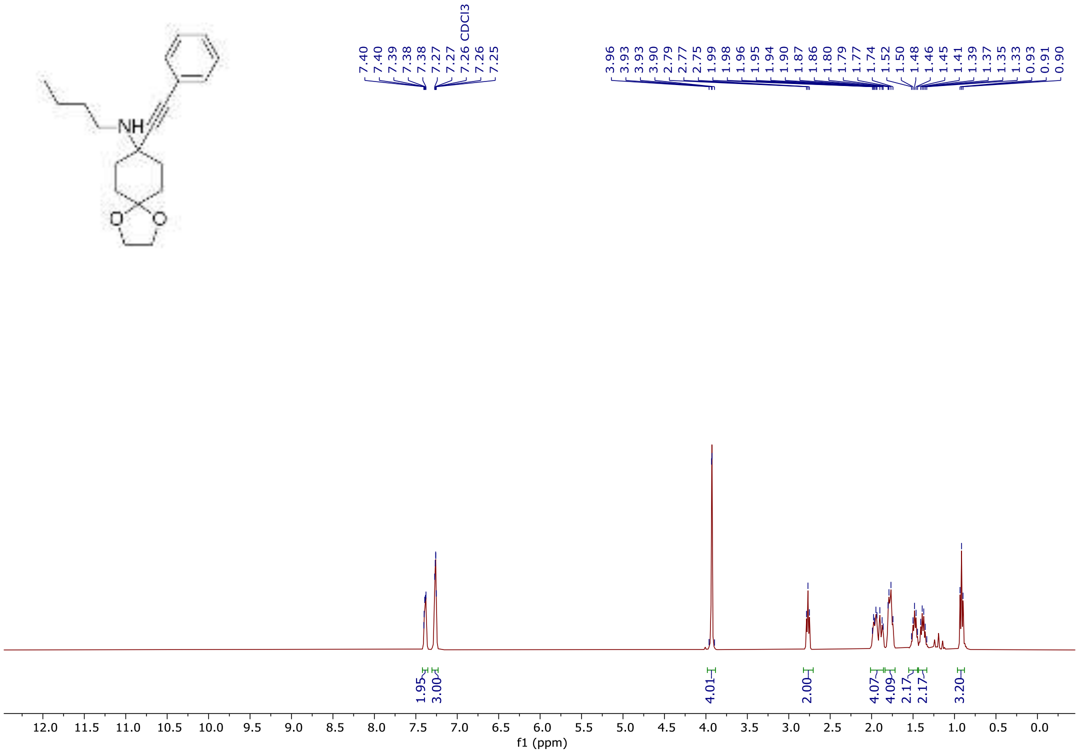

Figure S8: ^{1}H-NMR (400 MHz, $CDCl_3$) spectrum of compound **1c**.

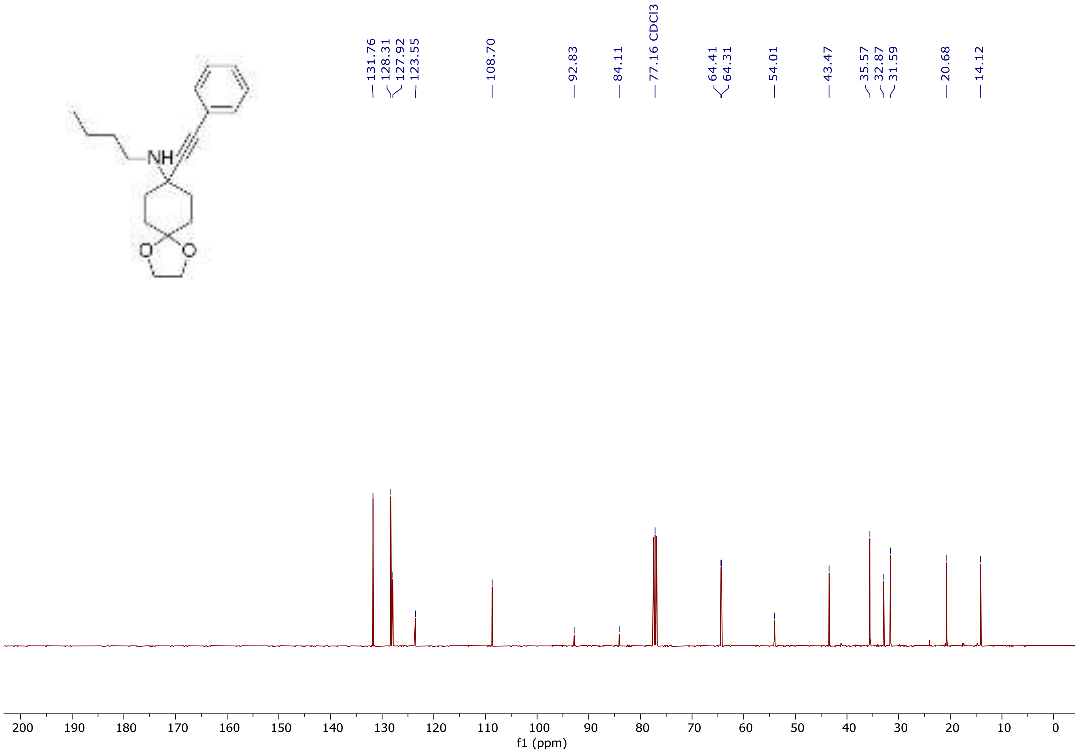

Figure S9: $^{13}C\{^{1}H\}$-NMR (101 MHz, $CDCl_3$) spectrum of compound **1c**.

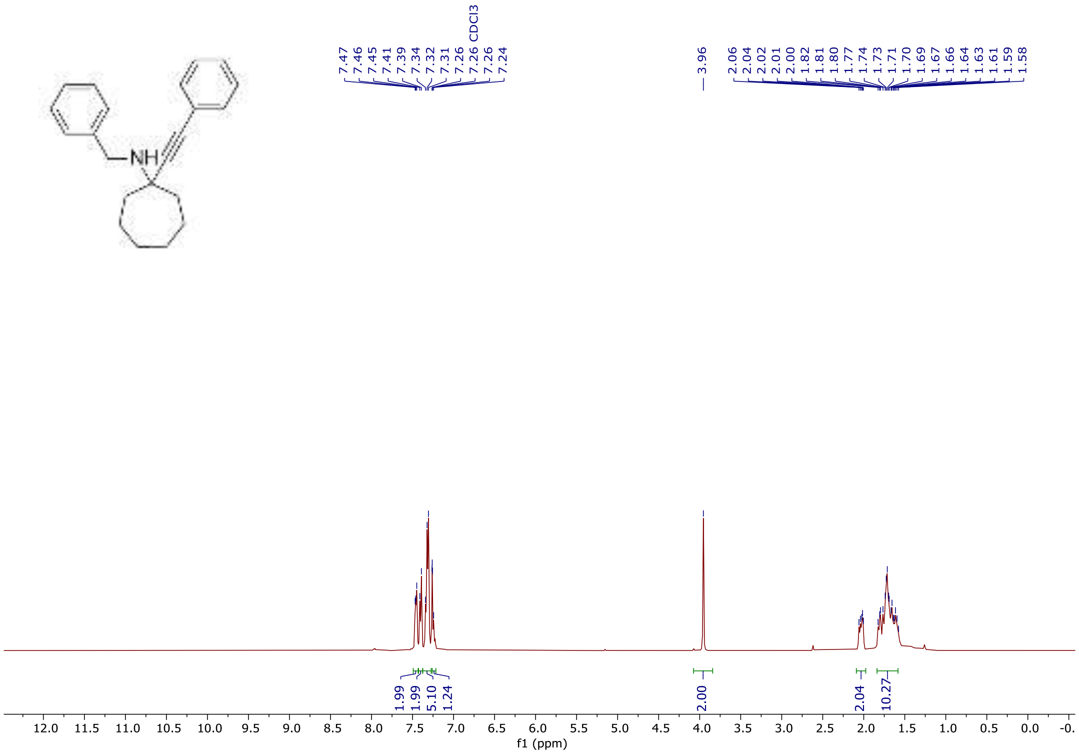

Figure S10: ^{1}H-NMR (400 MHz, $CDCl_3$) spectrum of compound **1d**.

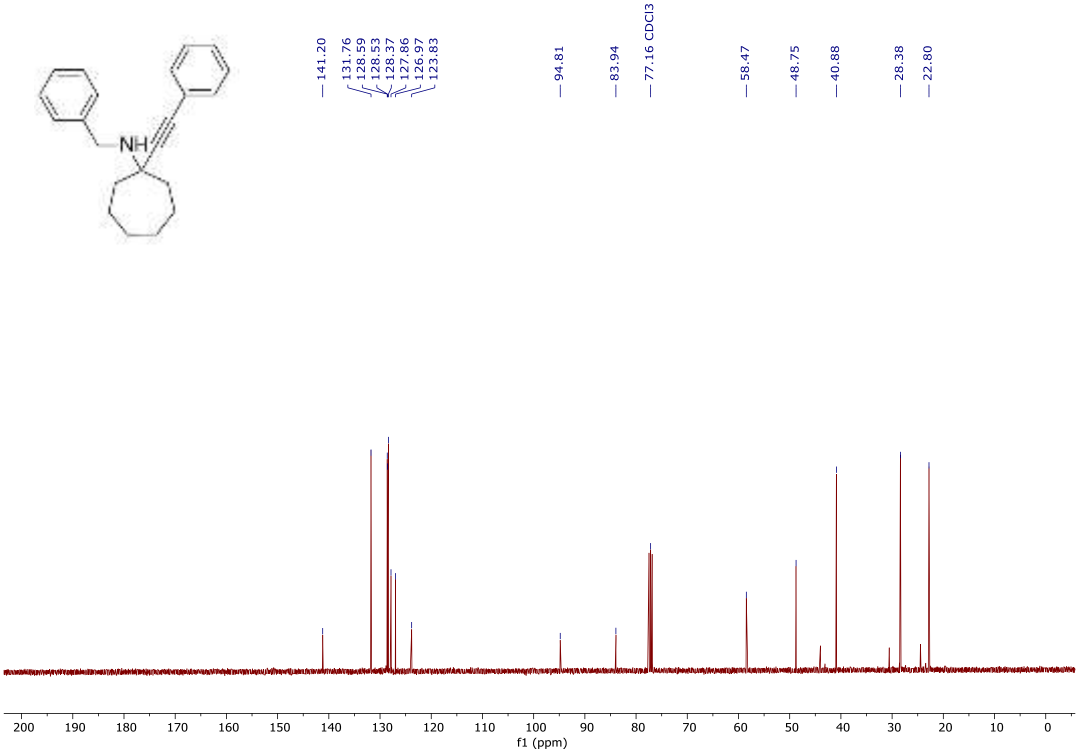

Figure S11: $^{13}C\{^1H\}$-NMR (101 MHz, $CDCl_3$) spectrum of compound **1d**.

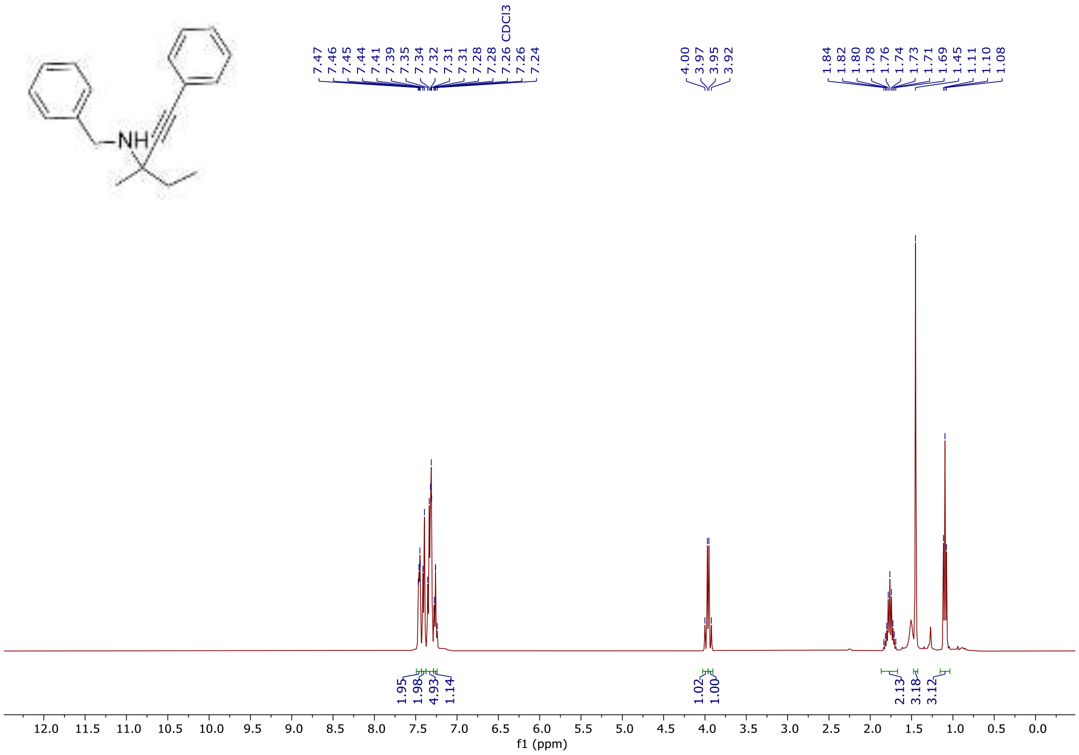

Figure S12: ^{1}H-NMR (400 MHz, $CDCl_3$) spectrum of compound **1f**.

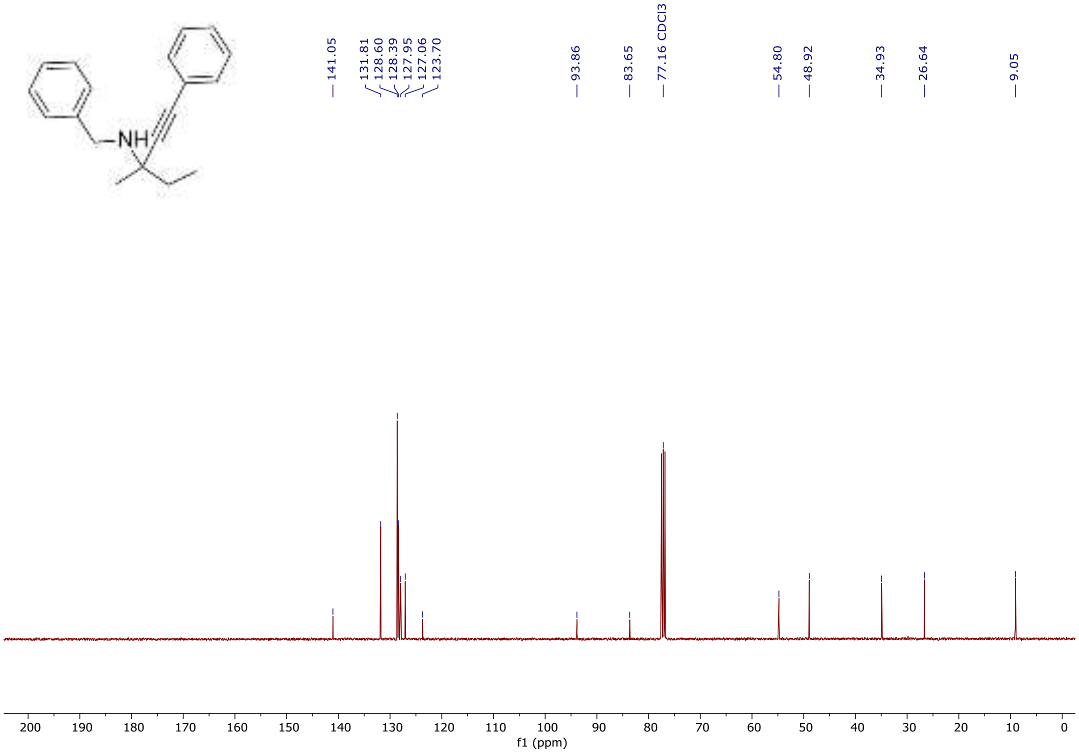

Figure S13: $^{13}C\{^{1}H\}$-NMR (101 MHz, $CDCl_3$) spectrum of compound **1f**.

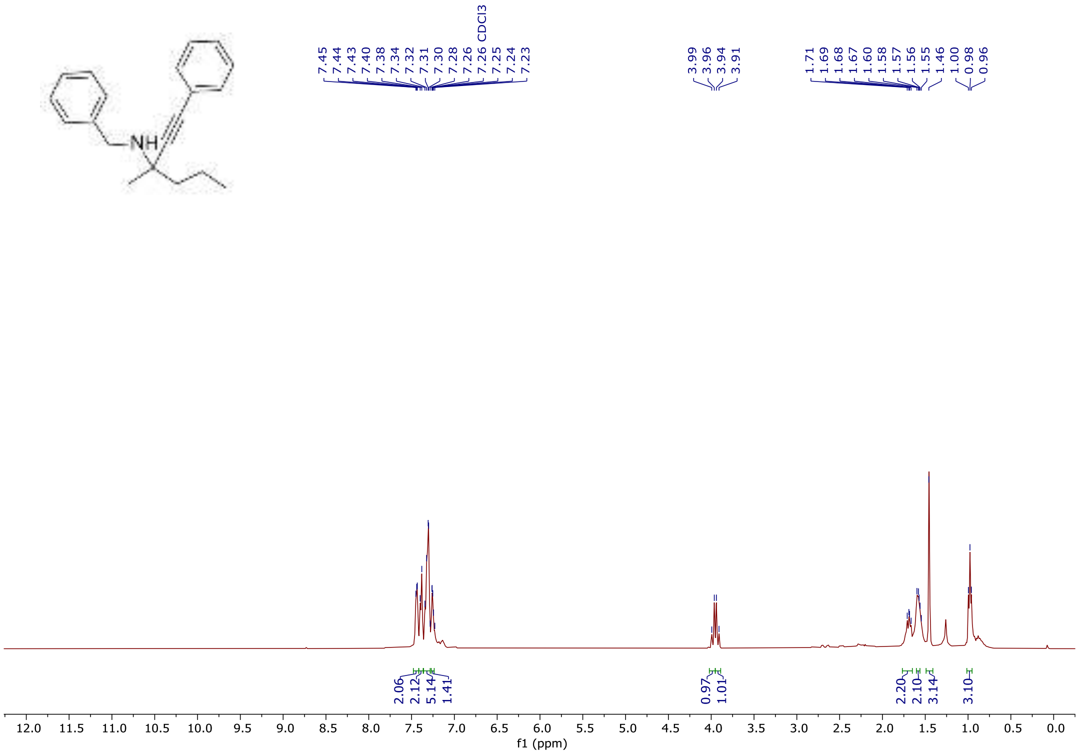

Figure S14: ^{1}H-NMR (400 MHz, $CDCl_3$) spectrum of compound **1g**.

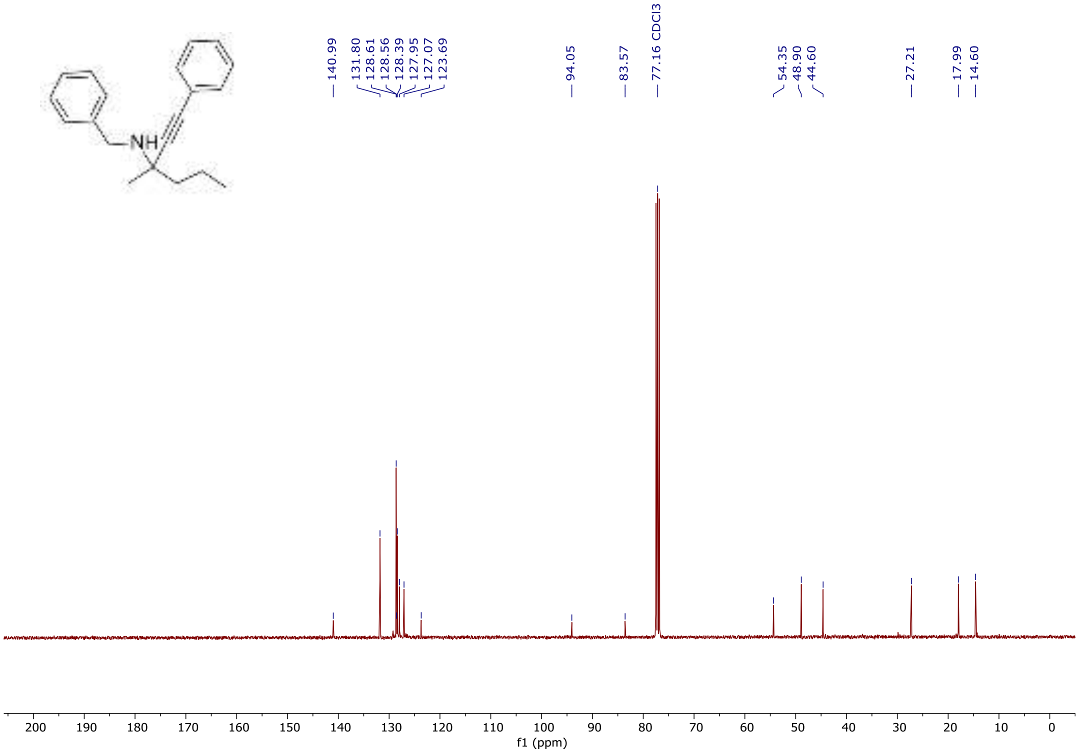

Figure S15: $^{13}C\{^{1}H\}$-NMR (101 MHz, $CDCl_3$) spectrum of compound **1g**.

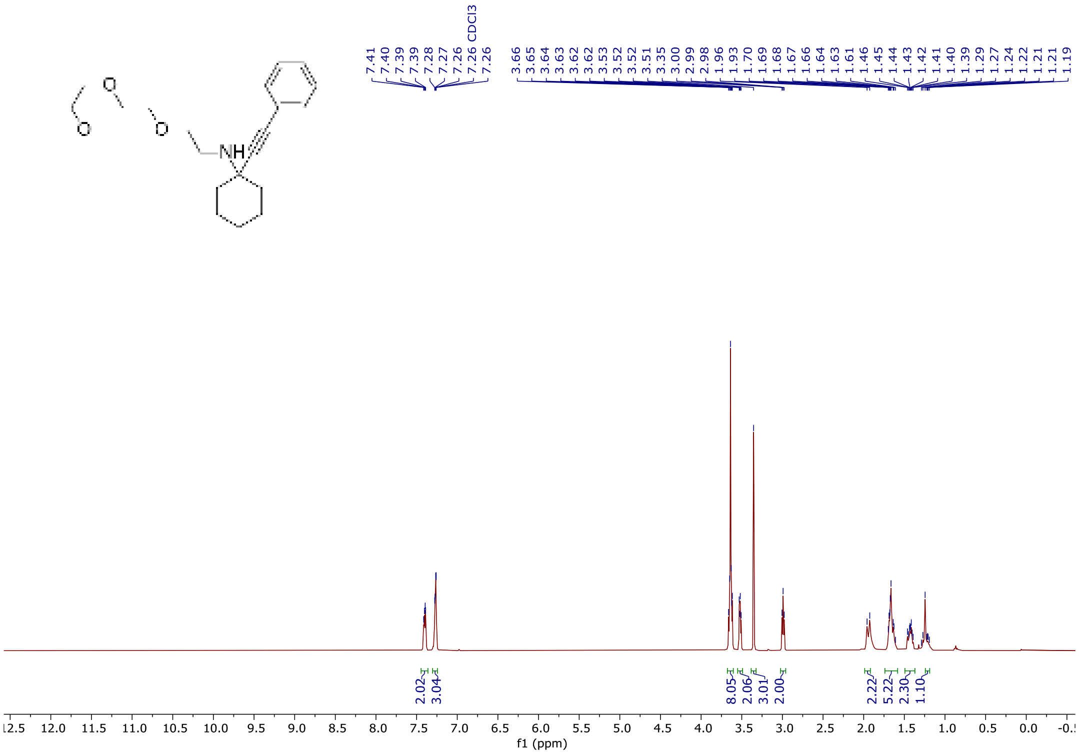

Figure S16: ^{1}H-NMR (400 MHz, $CDCl_3$) spectrum of compound **1l**.

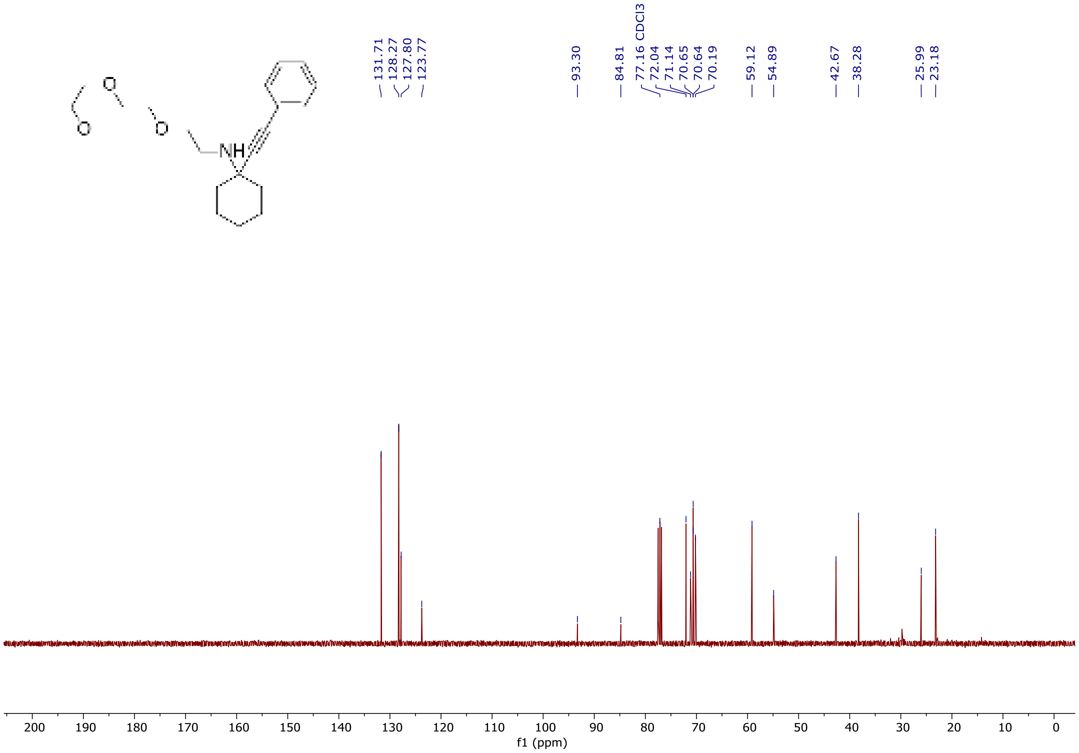

Figure S17: $^{13}C\{^{1}H\}$-NMR (101 MHz, $CDCl_3$) spectrum of compound **1l**.

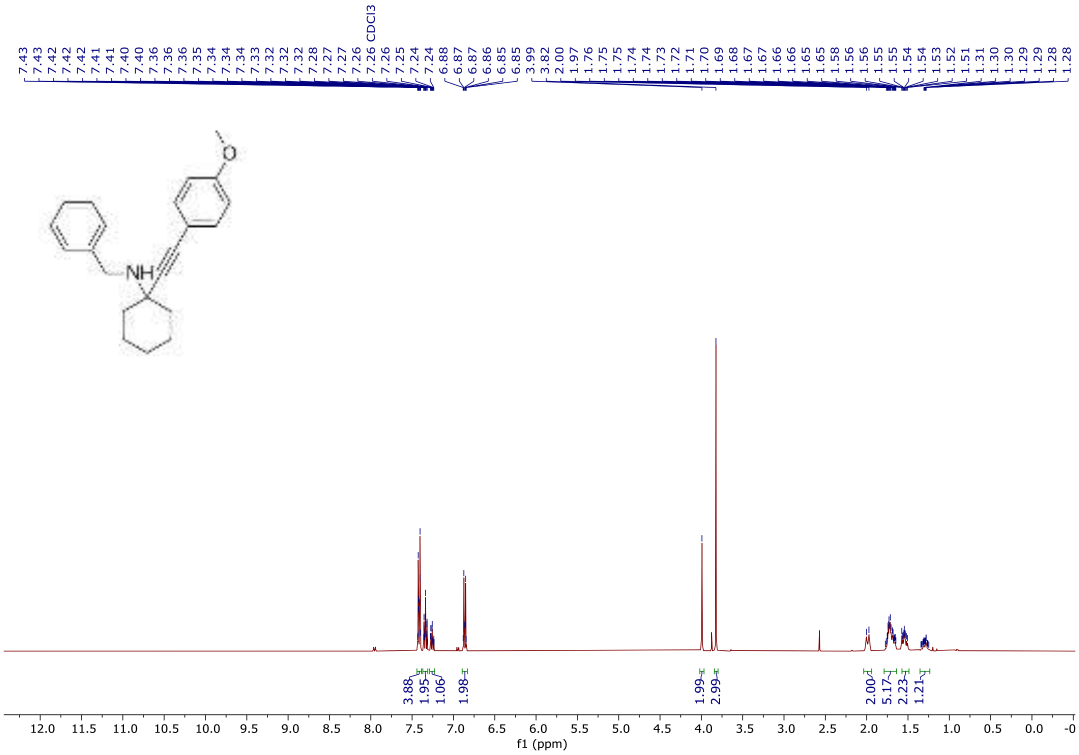

Figure S18: ^{1}H-NMR (400 MHz, $CDCl_3$) spectrum of compound **1n**.

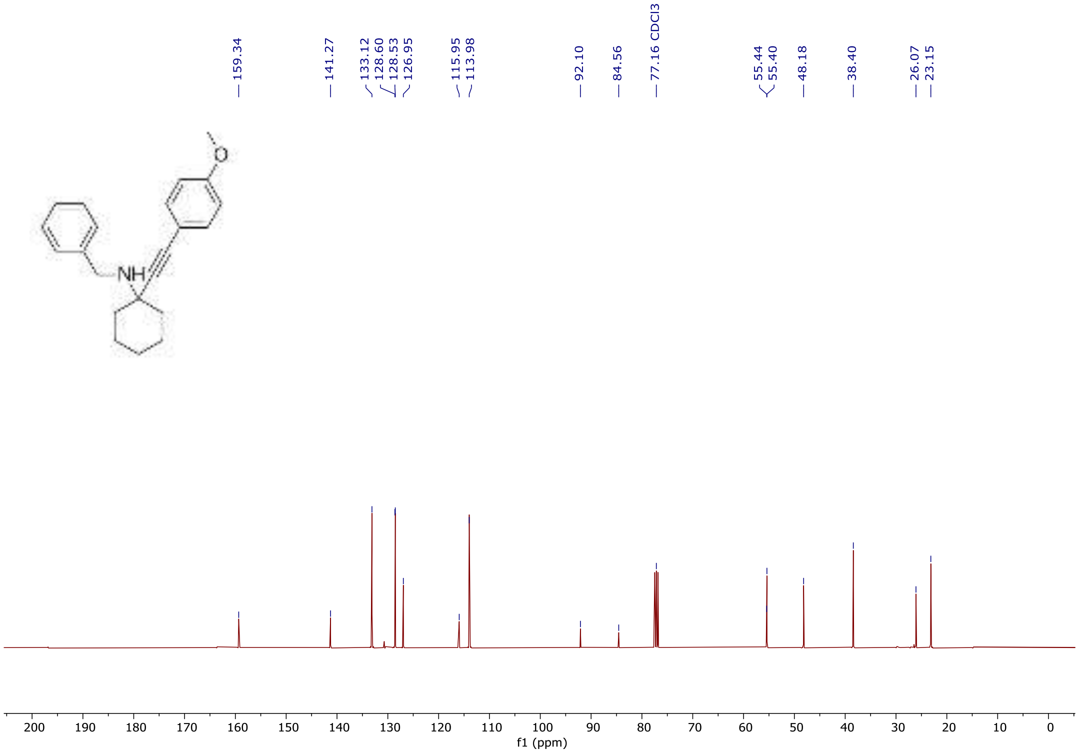

Figure S19: $^{13}C\{^{1}H\}$-NMR (101 MHz, $CDCl_3$) spectrum of compound **1n**.

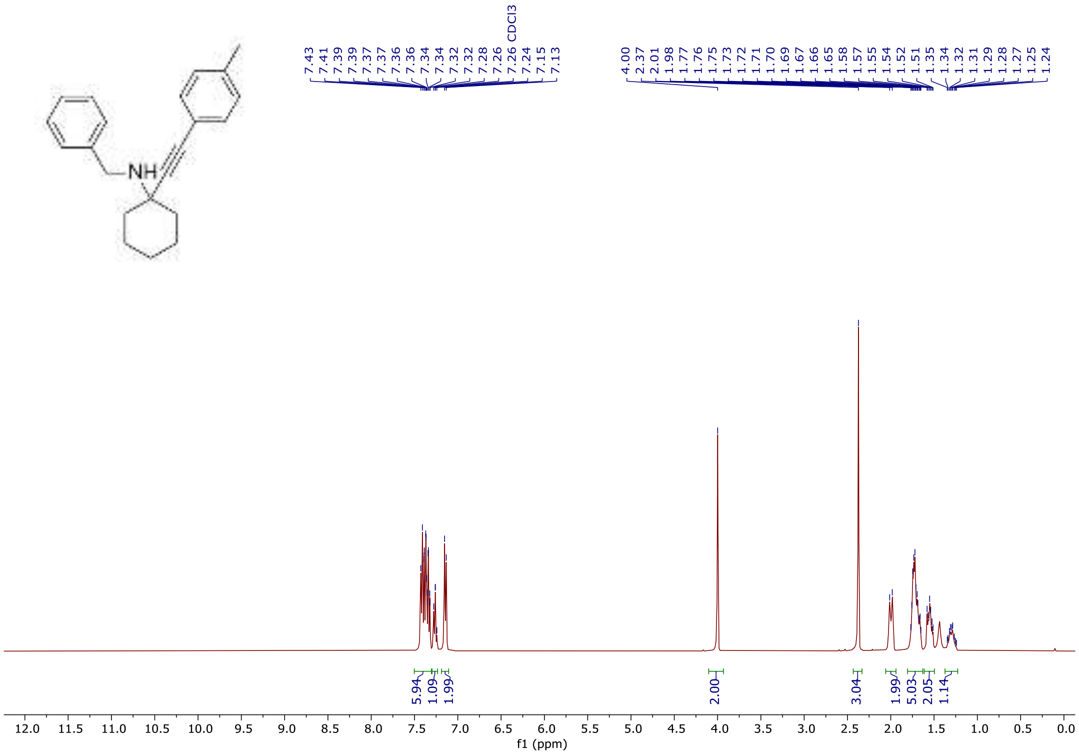

Figure S20: ^{1}H-NMR (400 MHz, $CDCl_3$) spectrum of compound **1o**.

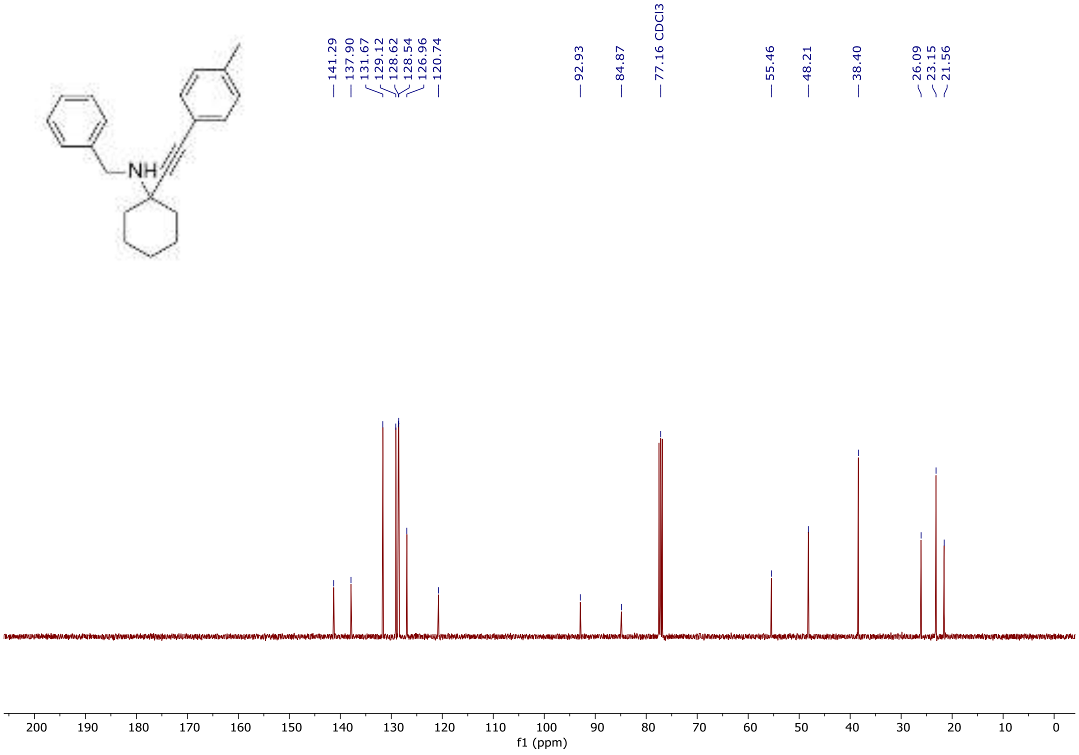

Figure S21: $^{13}C\{^{1}H\}$-NMR (101 MHz, $CDCl_3$) spectrum of compound **1o**.

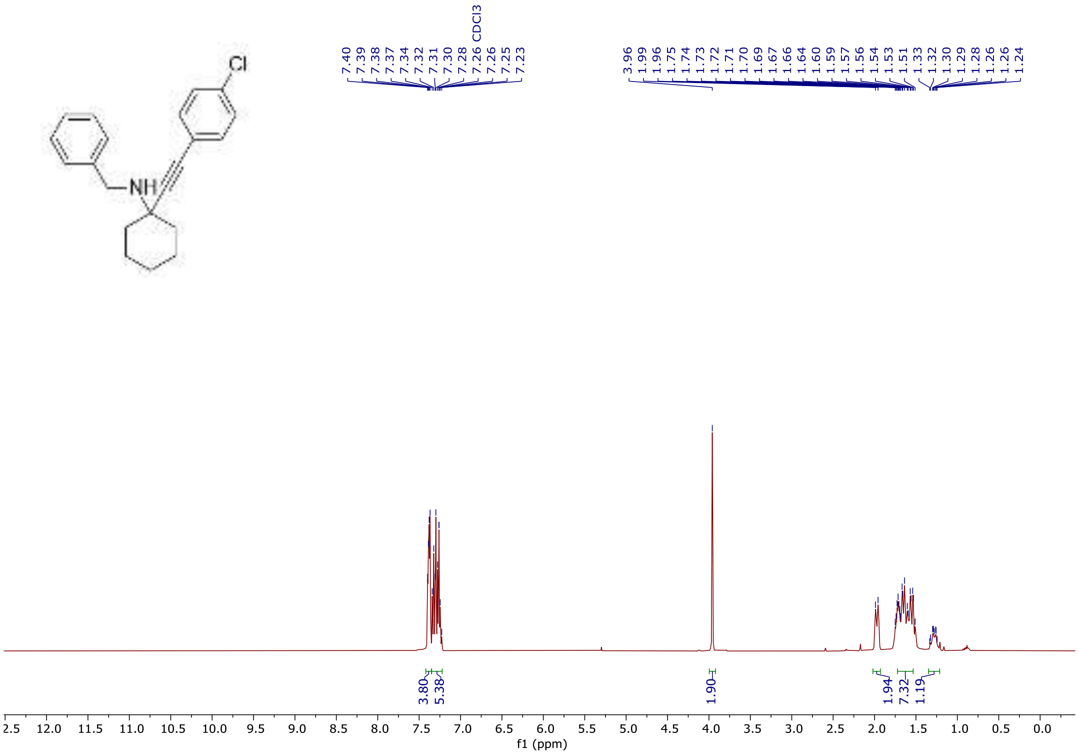

Figure S22: ^{1}H-NMR (400 MHz, $CDCl_3$) spectrum of compound **1p**.

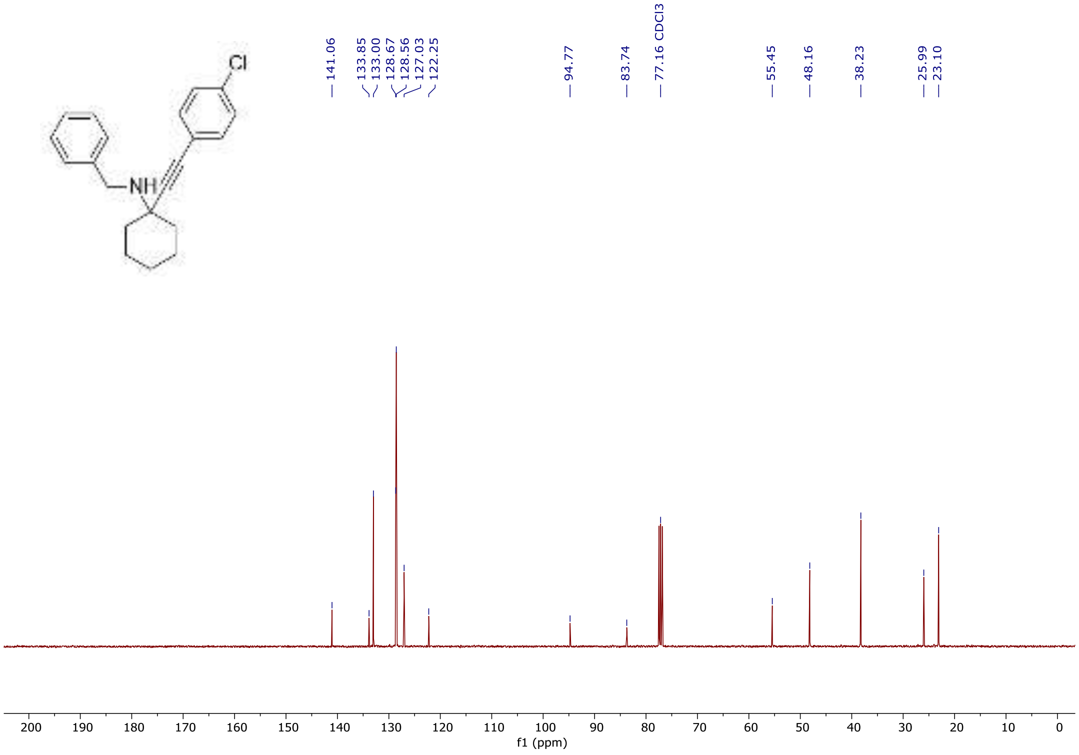

Figure S23: $^{13}C\{^{1}H\}$-NMR (101 MHz, $CDCl_3$) spectrum of compound **1p**.

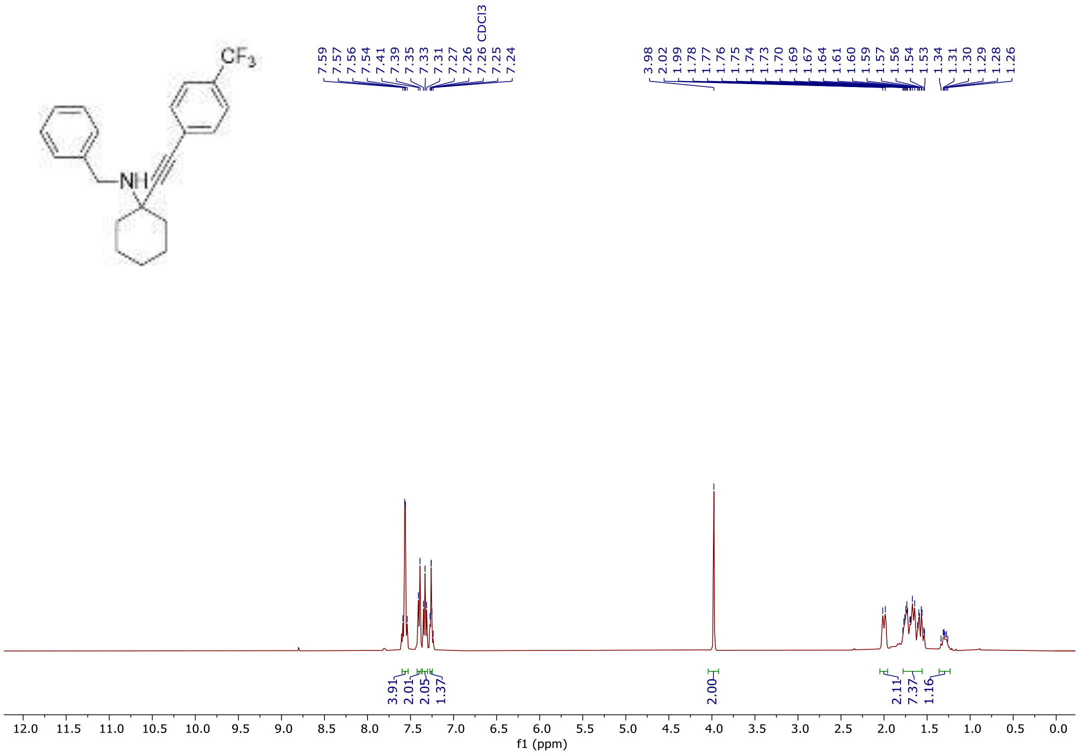

Figure S24: ^{1}H-NMR (400 MHz, $CDCl_3$) spectrum of compound **1q**.

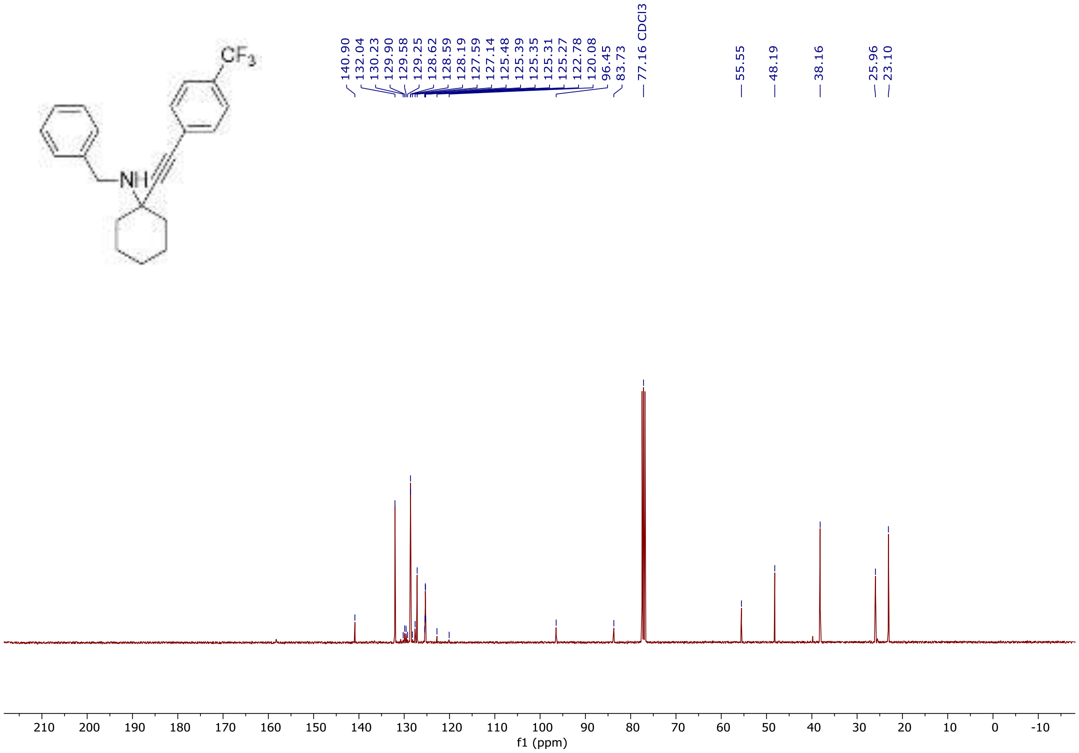

Figure S25: $^{13}C\{^{1}H\}$-NMR (101 MHz, $CDCl_3$) spectrum of compound **1q**.

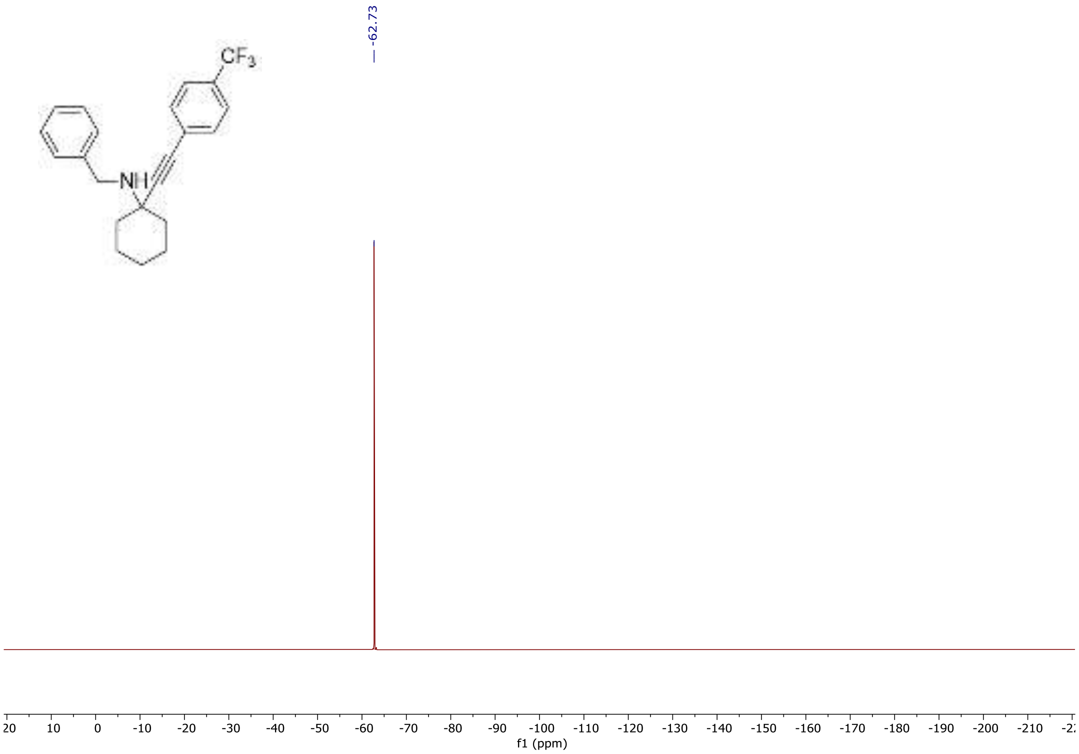

Figure S26: ^{19}F-NMR (376 MHz, $CDCl_3$) spectrum of compound **1q**.

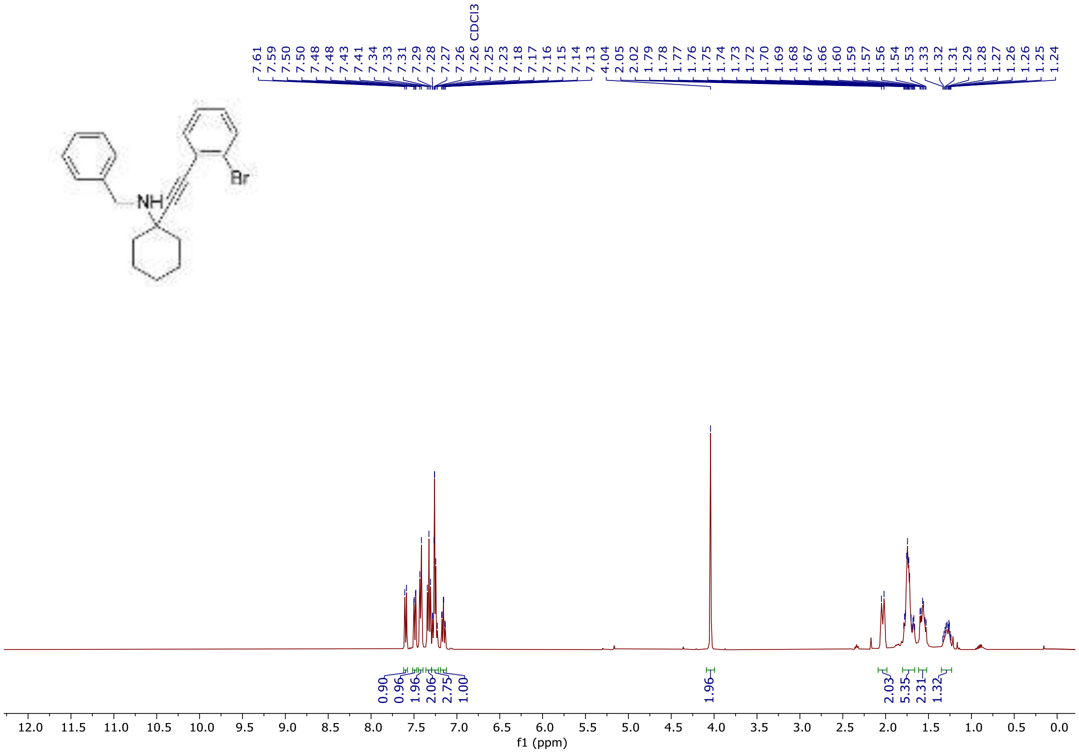

Figure S27: ^{1}H-NMR (400 MHz, $CDCl_3$) spectrum of compound **1r**.

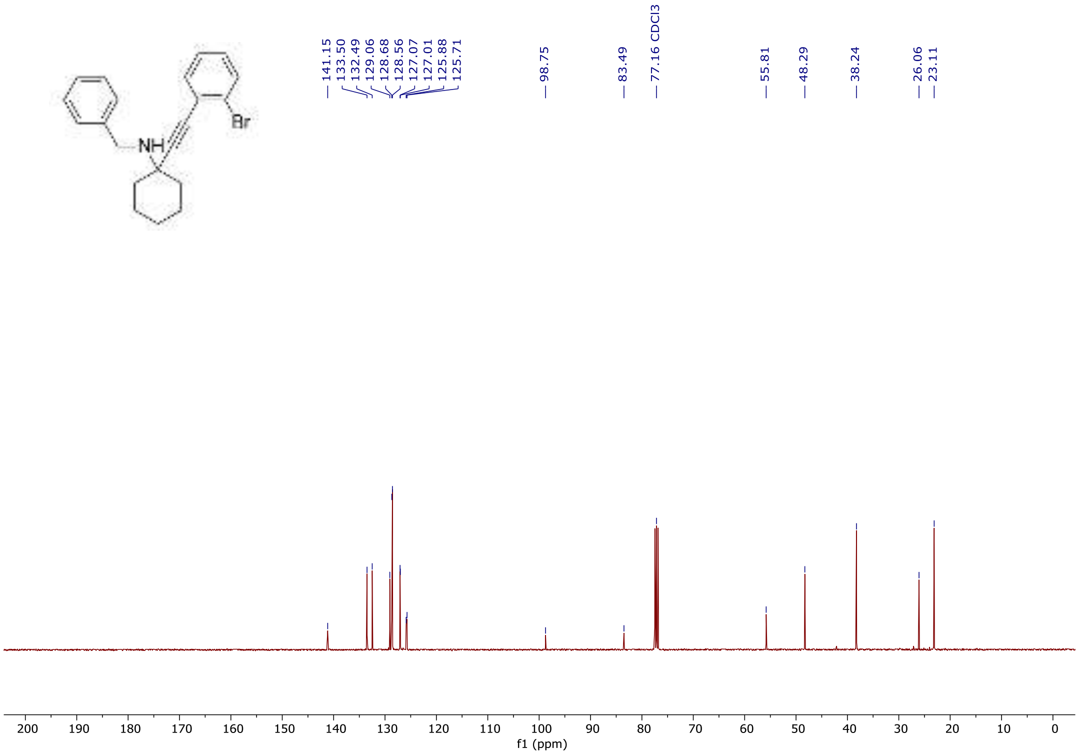

Figure S28: $^{13}C\{^{1}H\}$-NMR (101 MHz, $CDCl_3$) spectrum of compound **1r**.

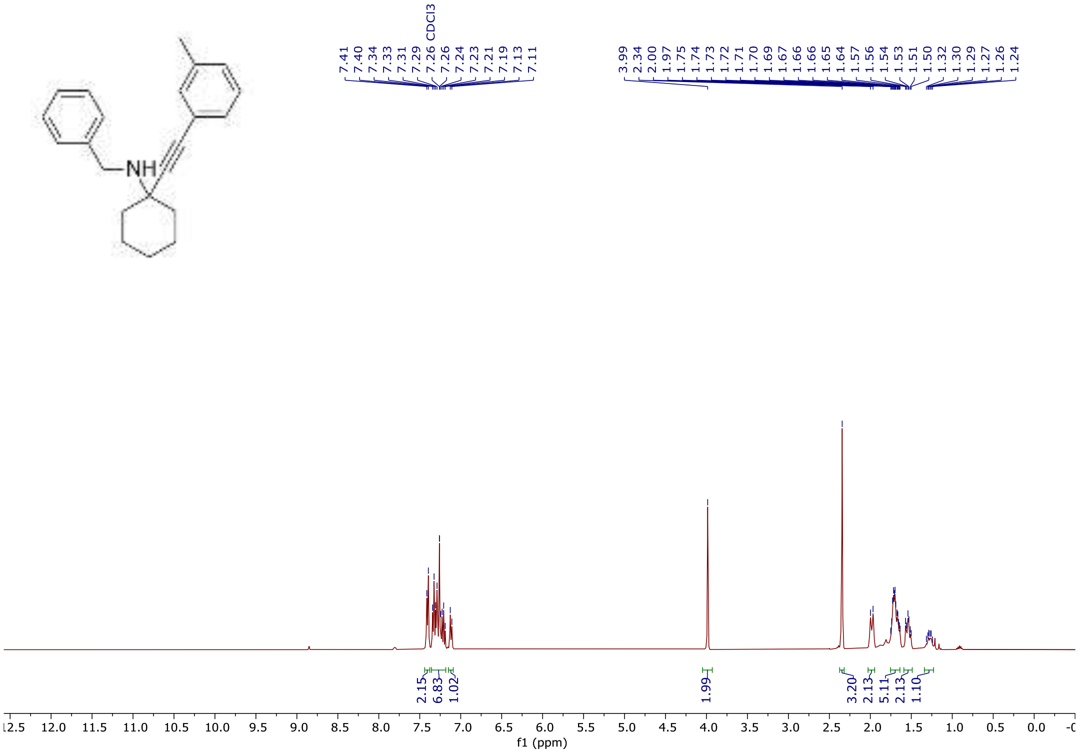

Figure S29: ^{1}H-NMR (400 MHz, $CDCl_3$) spectrum of compound **1s**.

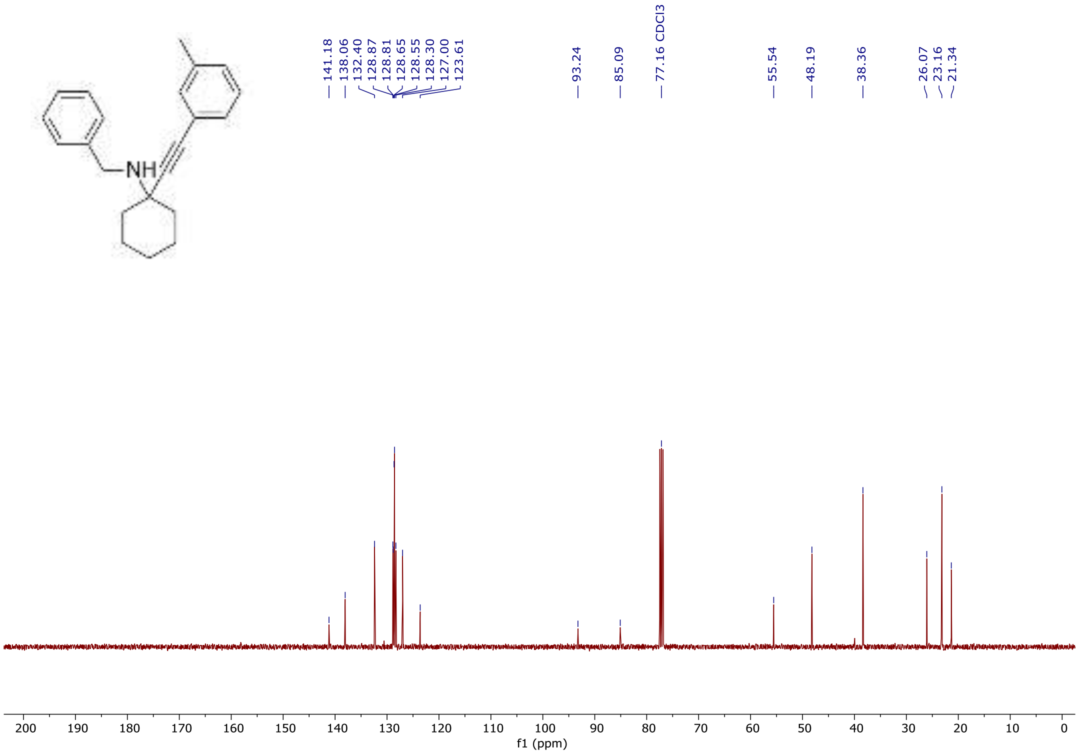

Figure S30: $^{13}C\{^{1}H\}$-NMR (101 MHz, $CDCl_3$) spectrum of compound **1s**.

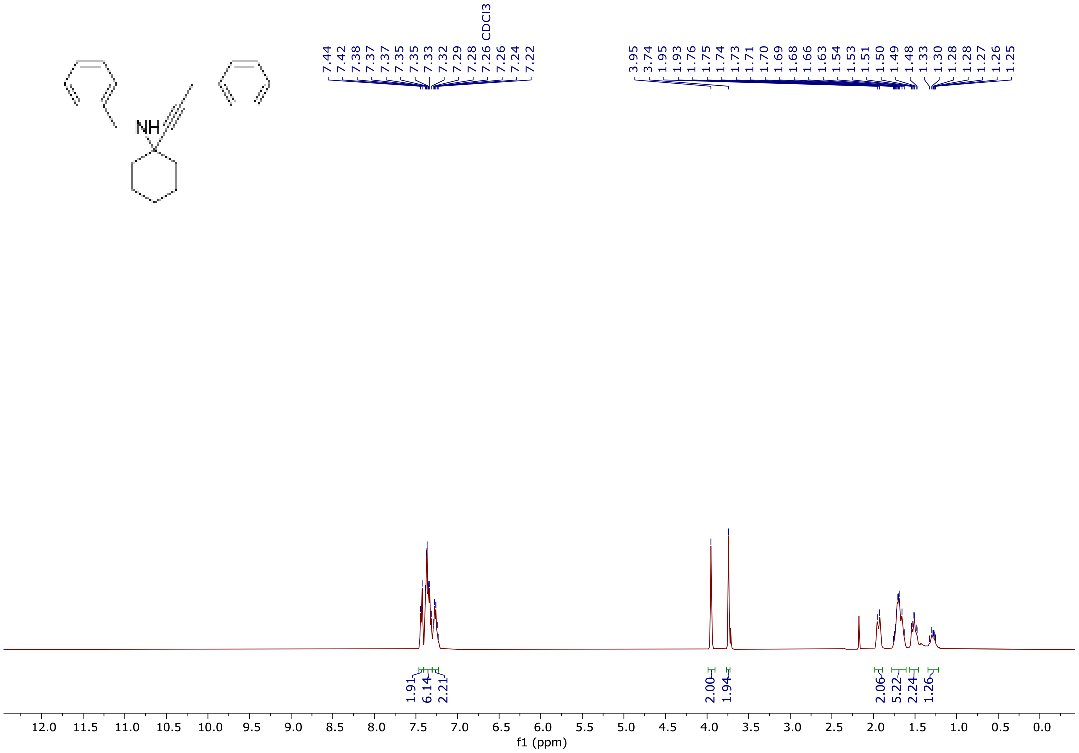

Figure S31: ^{1}H-NMR (400 MHz, $CDCl_3$) spectrum of compound **1v**.

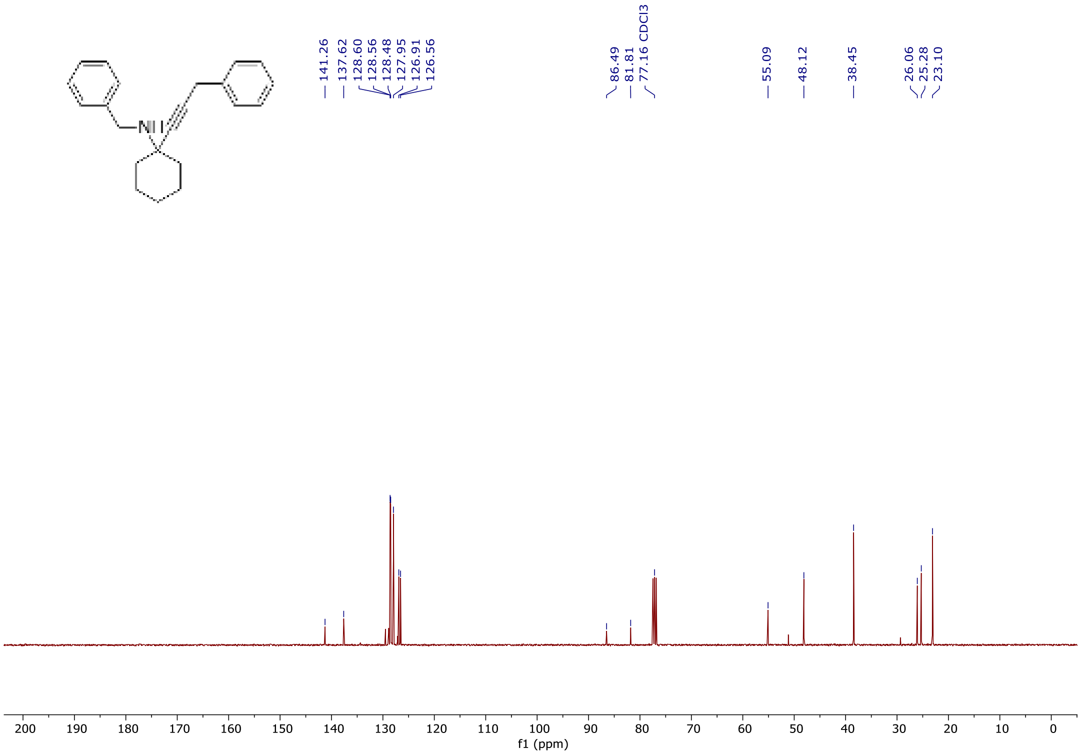

Figure S32: $^{13}C\{^{1}H\}$-NMR (101 MHz, $CDCl_3$) spectrum of compound **1v**.

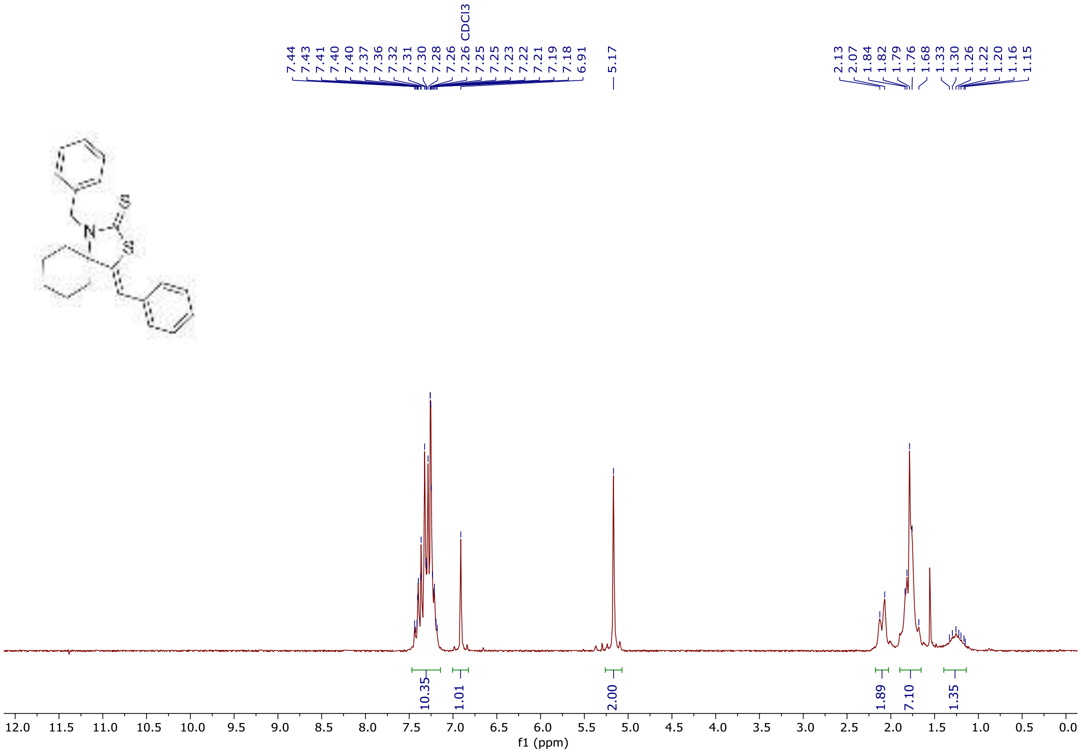

Figure S33: ^{1}H-NMR (200 MHz, $CDCl_3$) spectrum of compound **2a**.

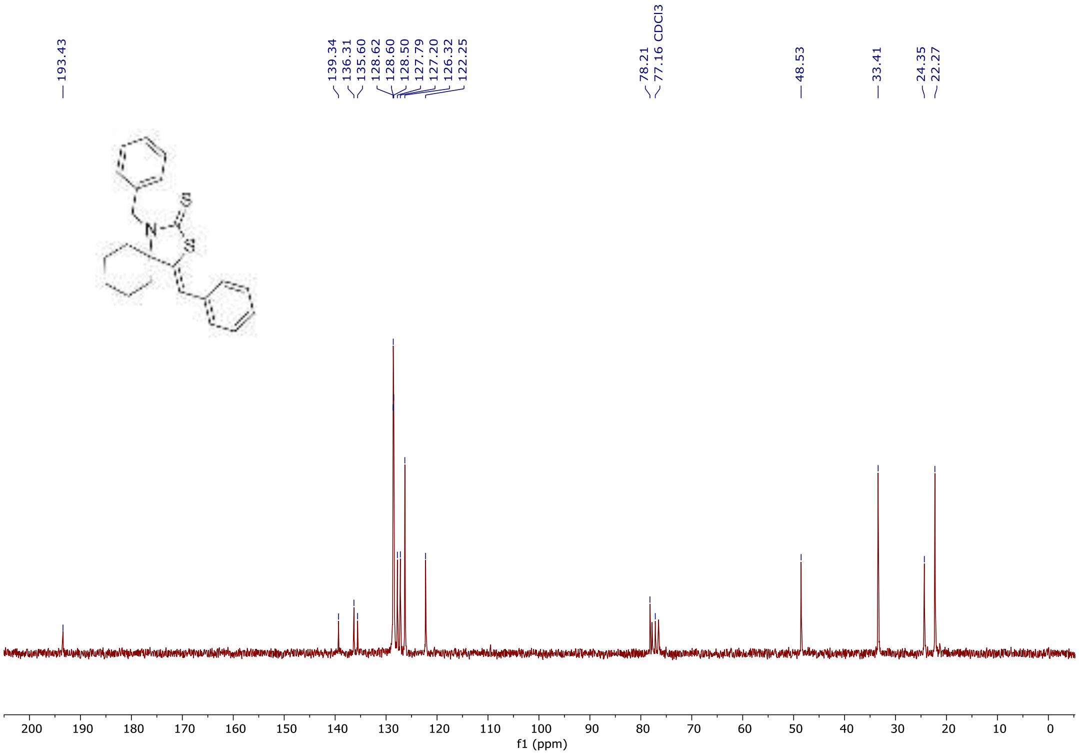

Figure S34: $^{13}C\{^{1}H\}$-NMR (50 MHz, $CDCl_3$) spectrum of compound **2a**.

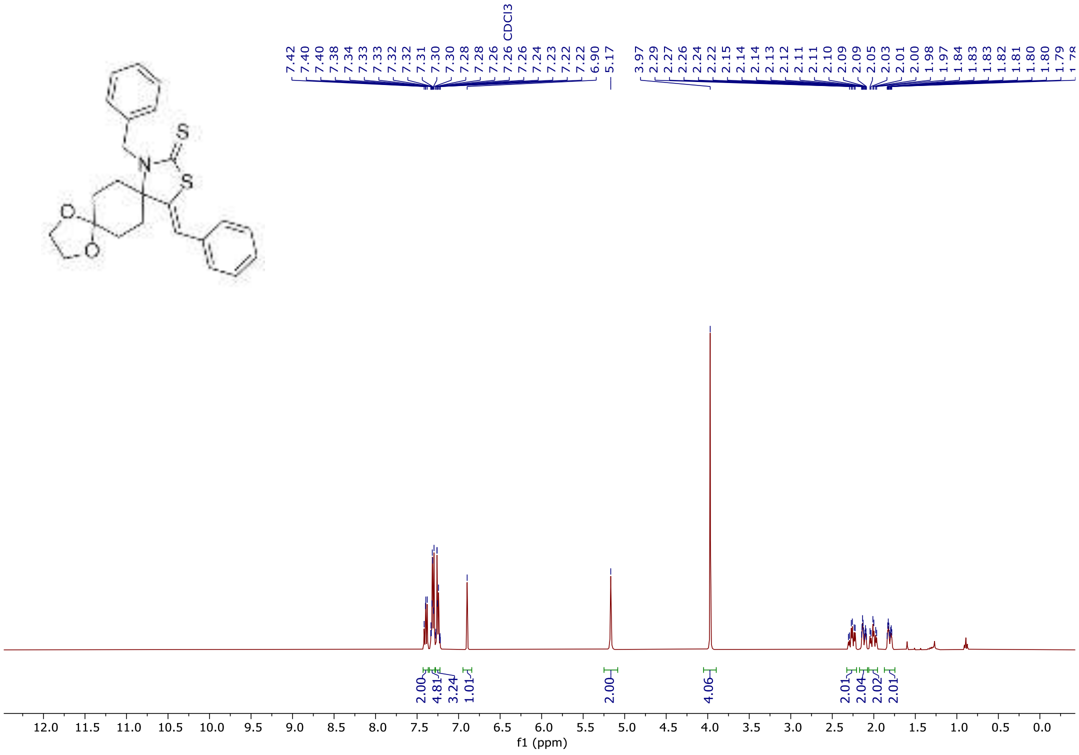

Figure S35: ^{1}H-NMR (400 MHz, $CDCl_3$) spectrum of compound **2b**.

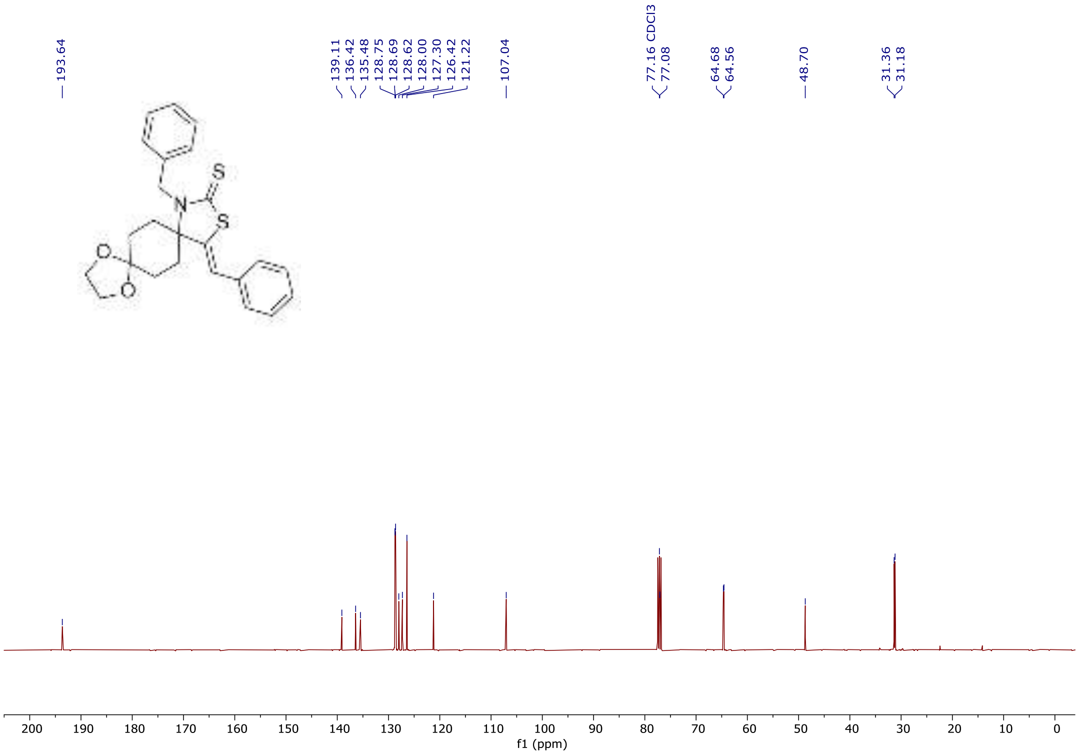

Figure S36: $^{13}C\{^{1}H\}$-NMR (101 MHz, $CDCl_3$) spectrum of compound **2b**.

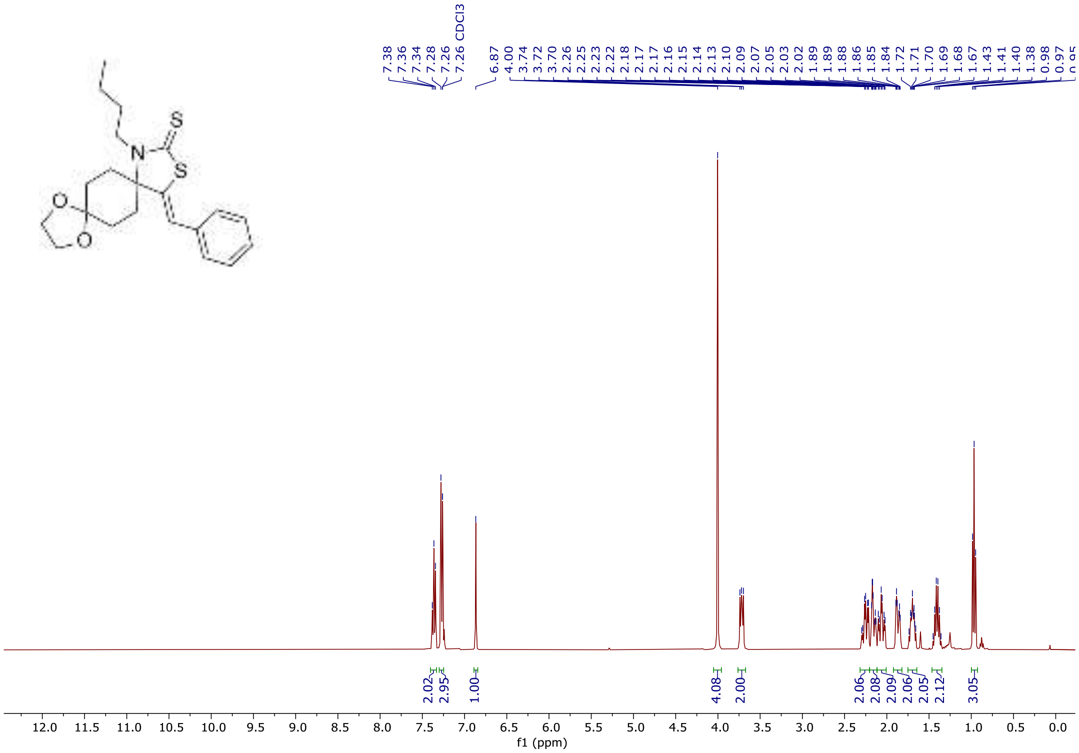

Figure S37: ^{1}H-NMR (400 MHz, $CDCl_3$) spectrum of compound **2c**.

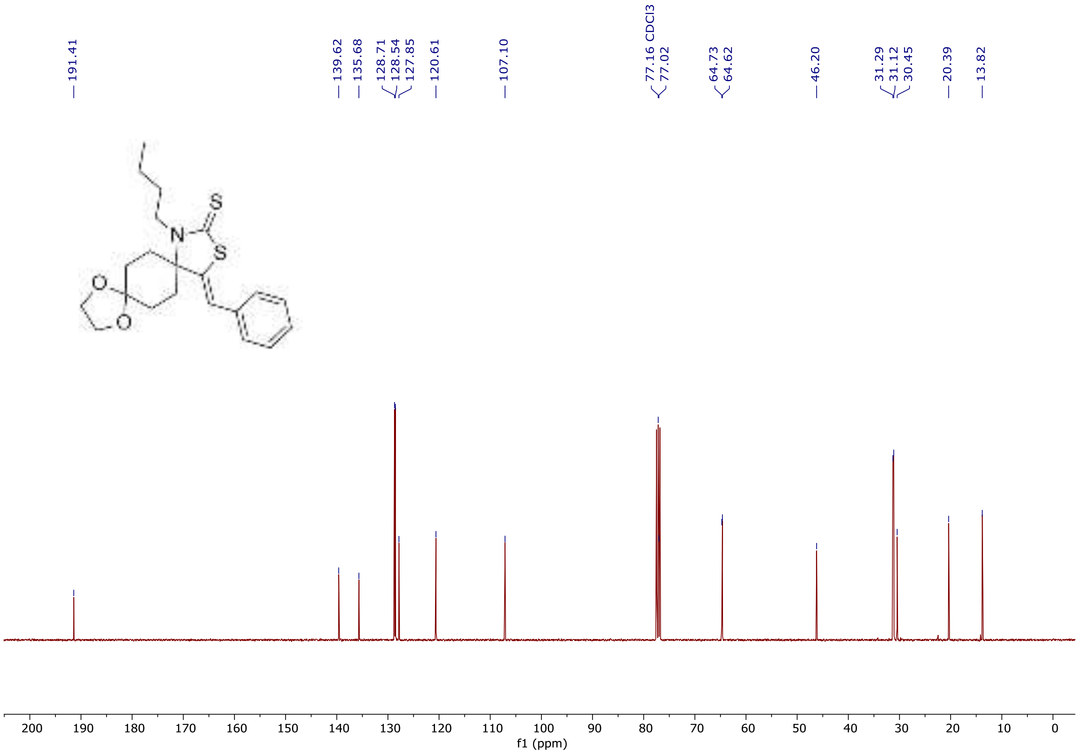

Figure S38: $^{13}C\{^{1}H\}$-NMR (101 MHz, $CDCl_3$) spectrum of compound **2c**.

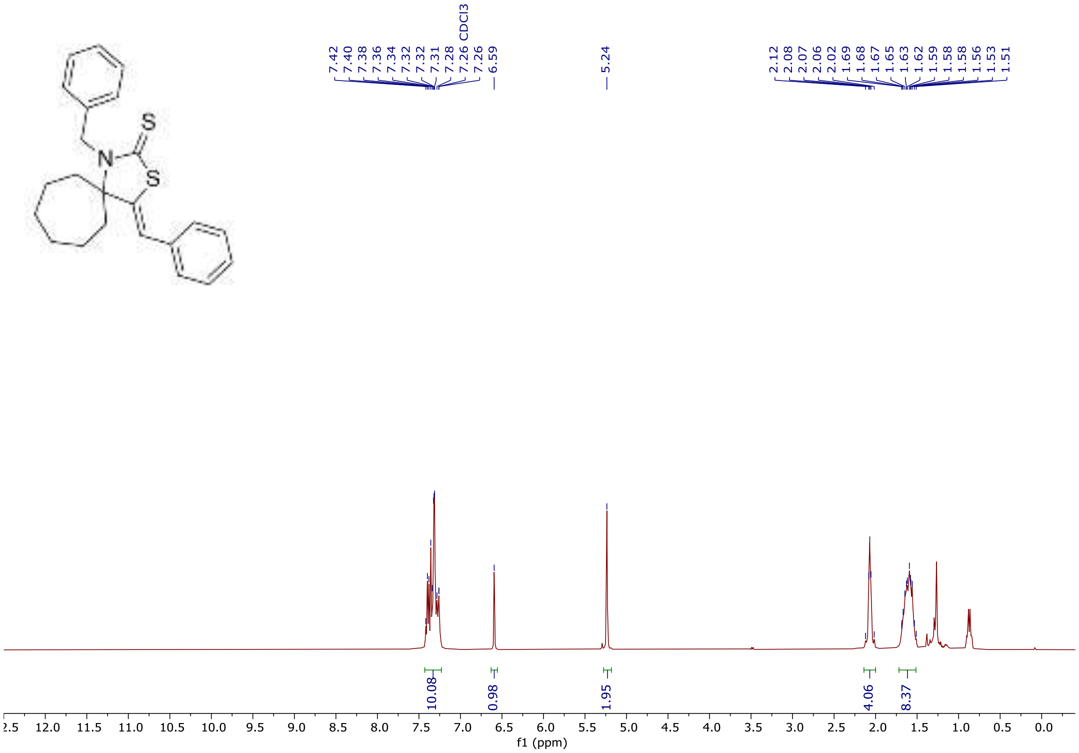

Figure S39: ^{1}H-NMR (400 MHz, $CDCl_3$) spectrum of compound **2d**.

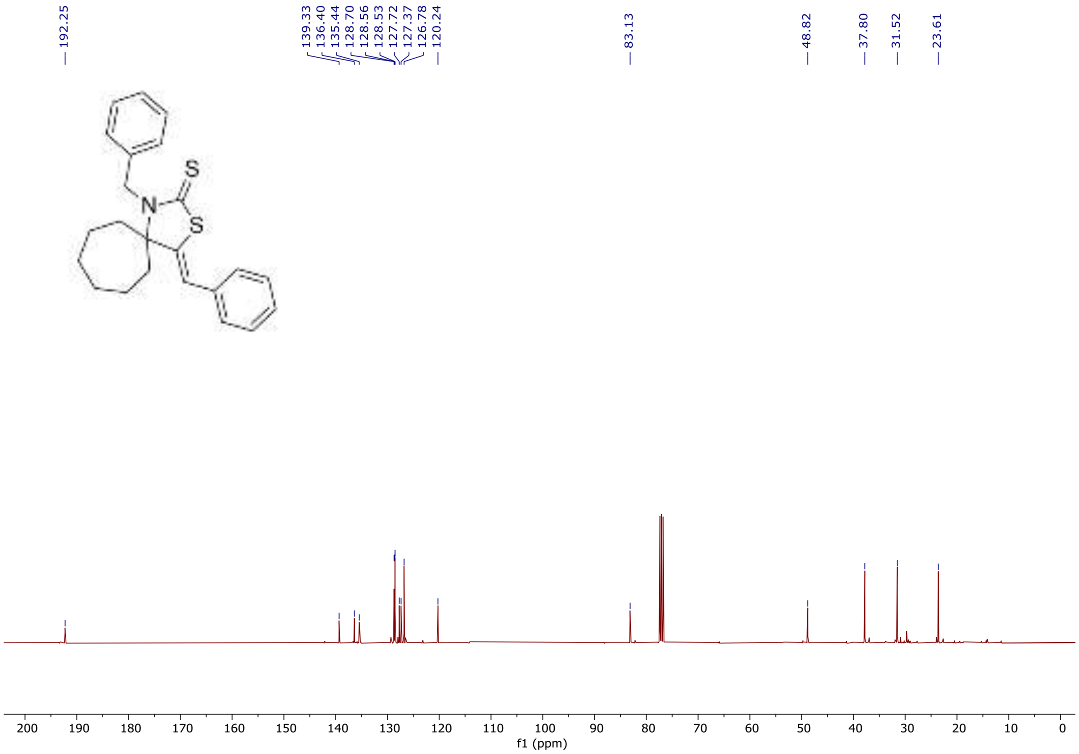

Figure S40: $^{13}C\{^{1}H\}$-NMR (101 MHz, $CDCl_3$) spectrum of compound **2d**.

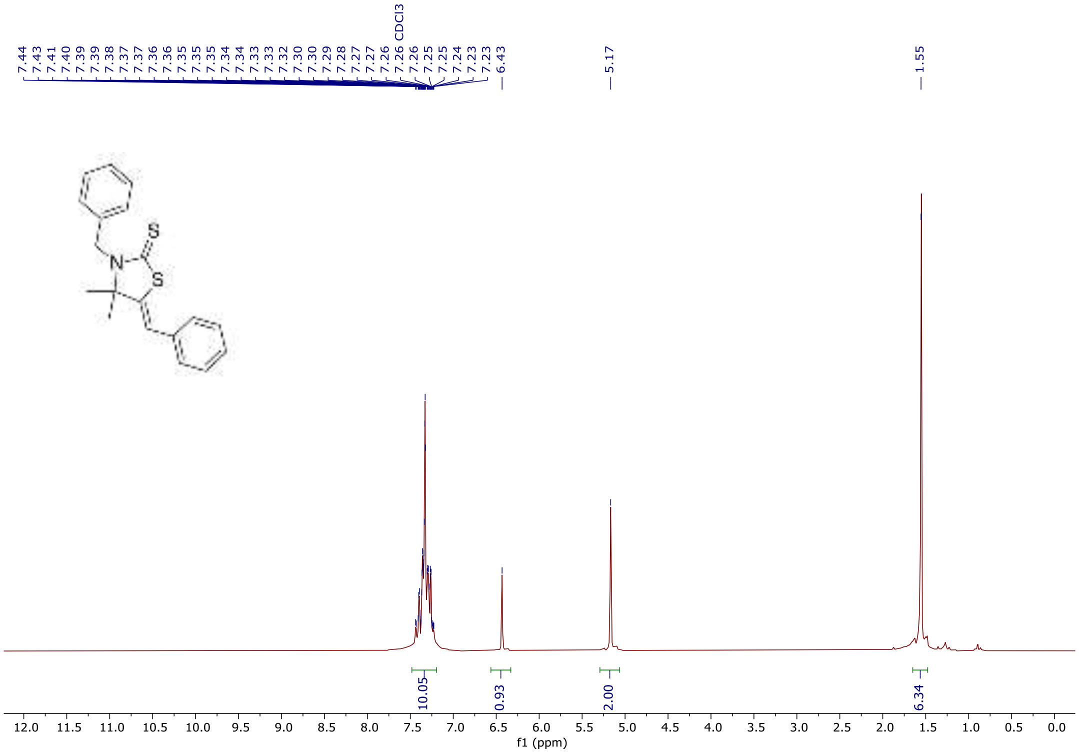

Figure S41: ^{1}H-NMR (200 MHz, $CDCl_3$) spectrum of compound **2e**.

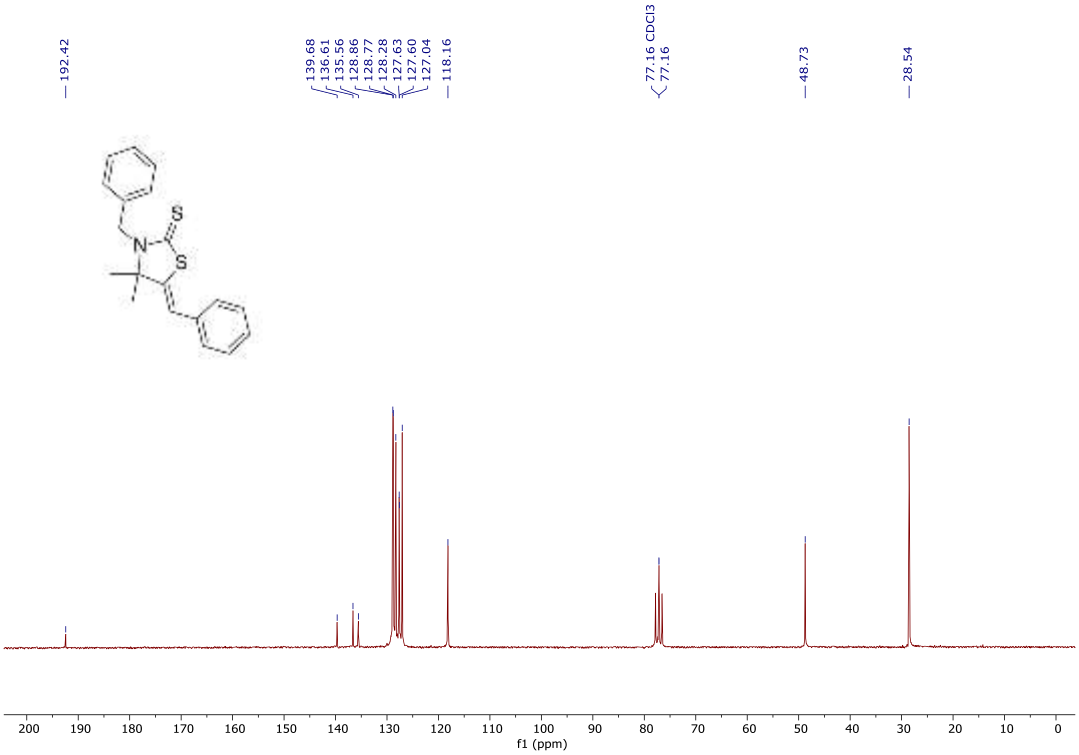

Figure S42: ^{13}C{1H}-NMR (50 MHz, $CDCl_3$) spectrum of compound **2e**.

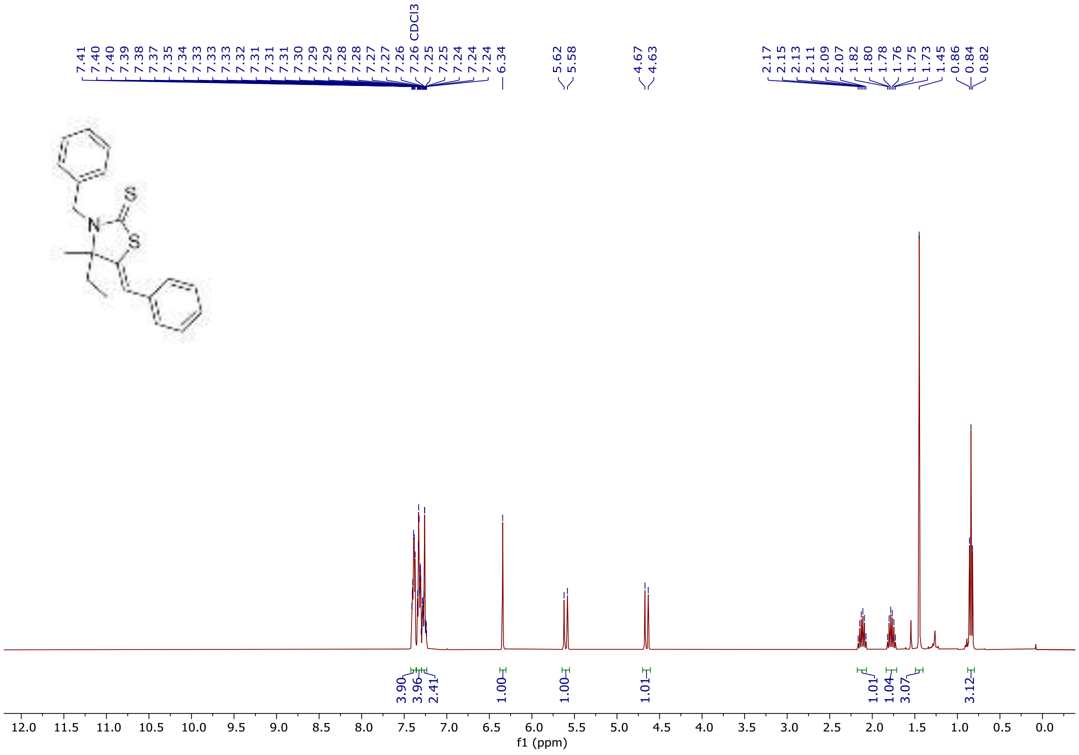

Figure S43: ^{1}H-NMR (400 MHz, $CDCl_3$) spectrum of compound **2f**.

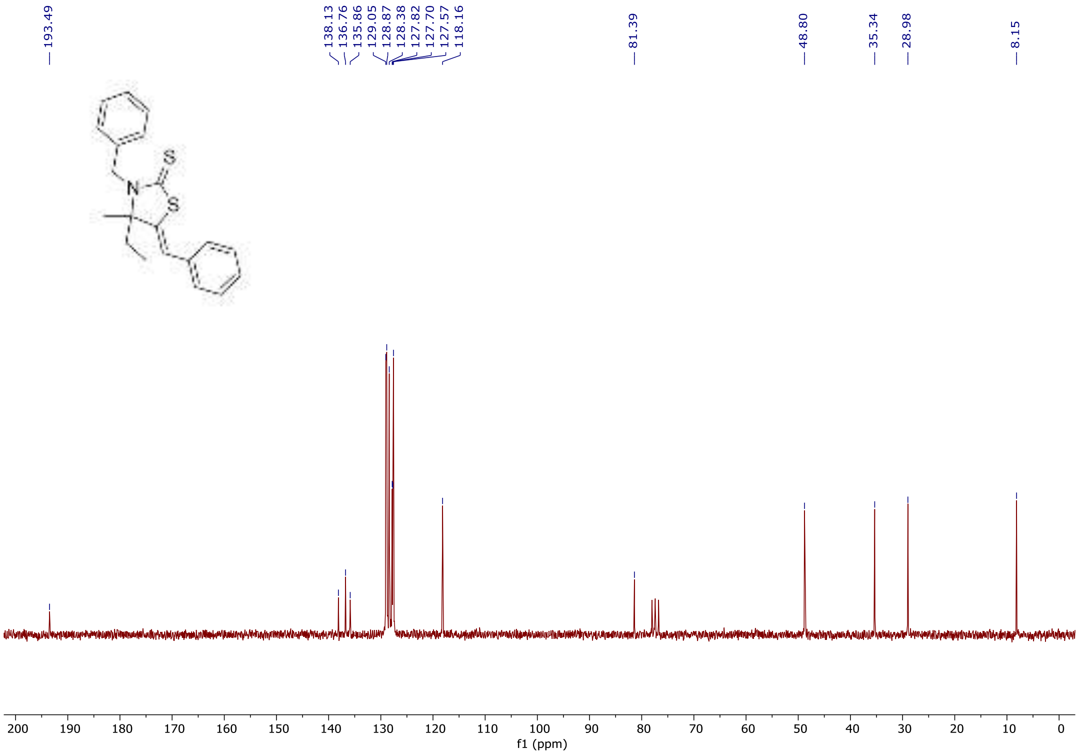

Figure S45: $^{13}C\{1H\}$-NMR (50 MHz, $CDCl_3$) spectrum of compound **2f**.

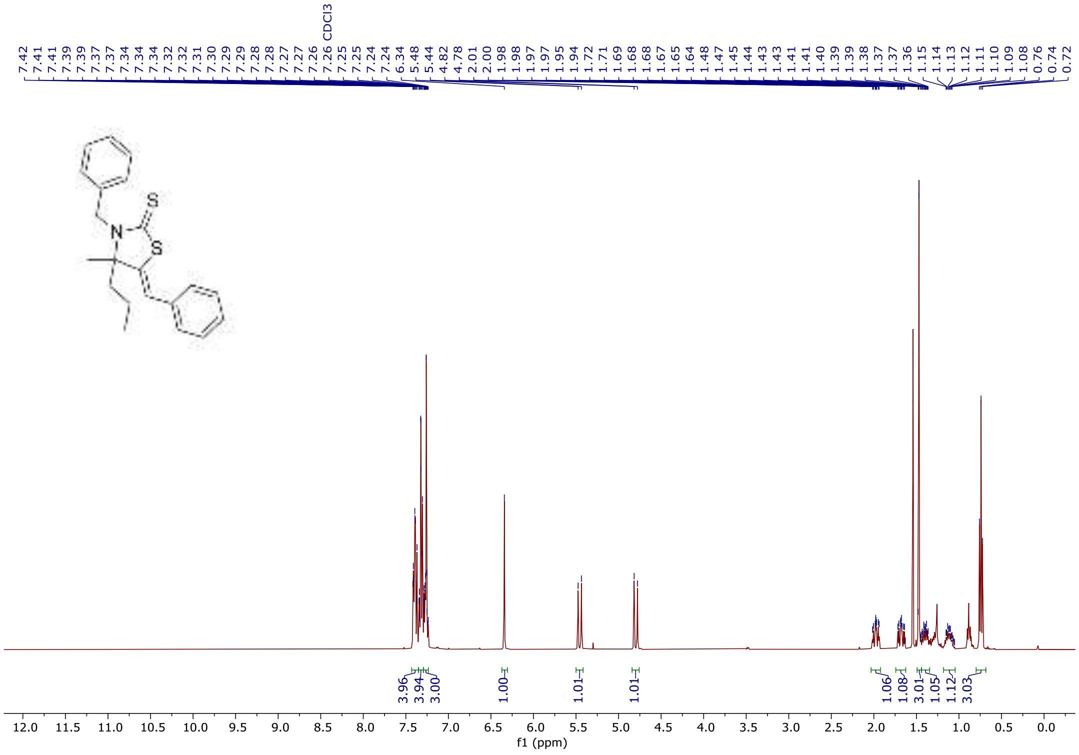

Figure S46: ^{1}H-NMR (400 MHz, $CDCl_3$) spectrum of compound **2g**.

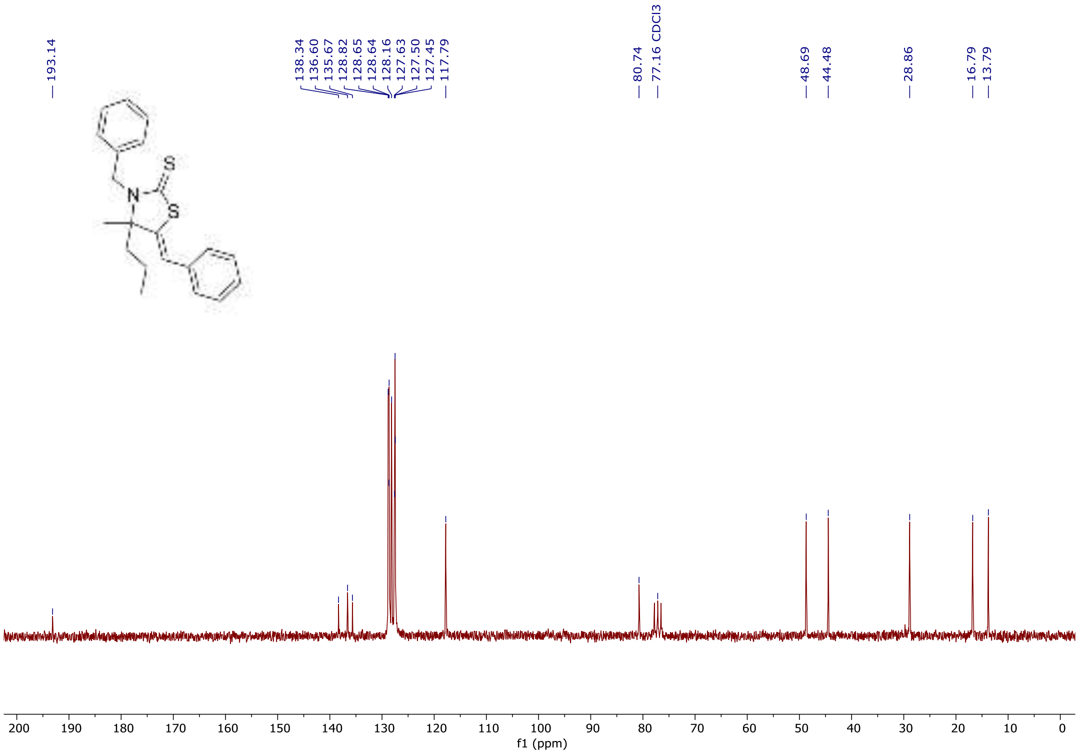

Figure S47: $^{13}C\{1H\}$-NMR (50 MHz, $CDCl_3$) spectrum of compound **2g**.

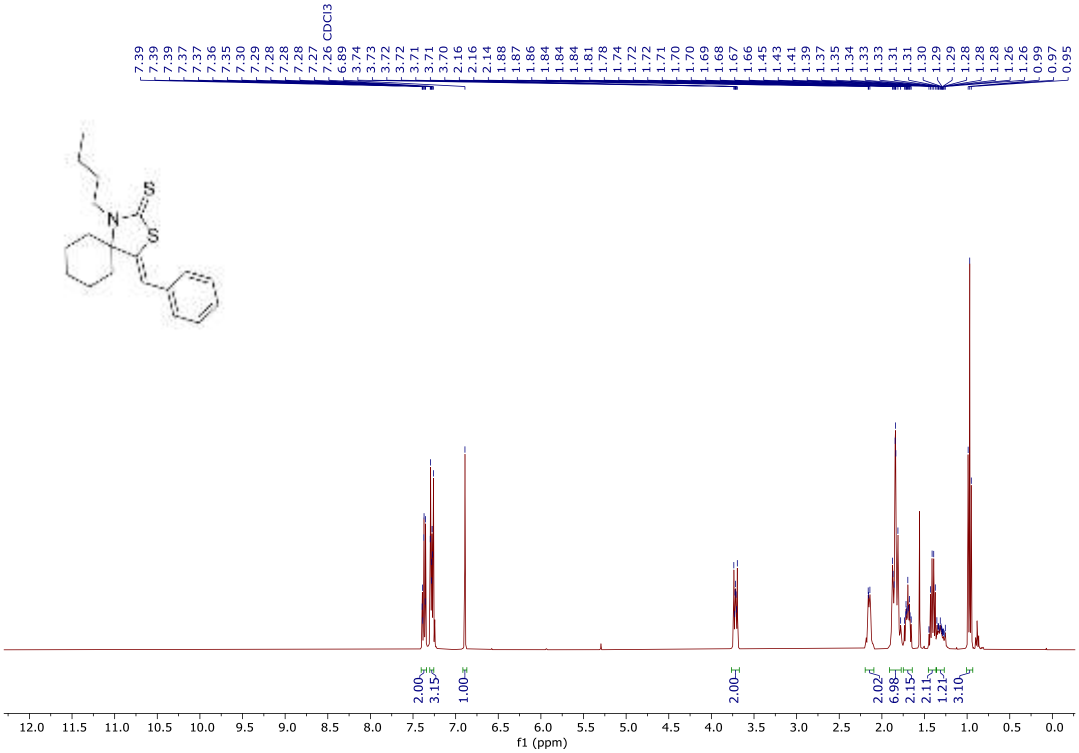

Figure S48: ^1H-NMR (400 MHz, $CDCl_3$) spectrum of compound **2h**.

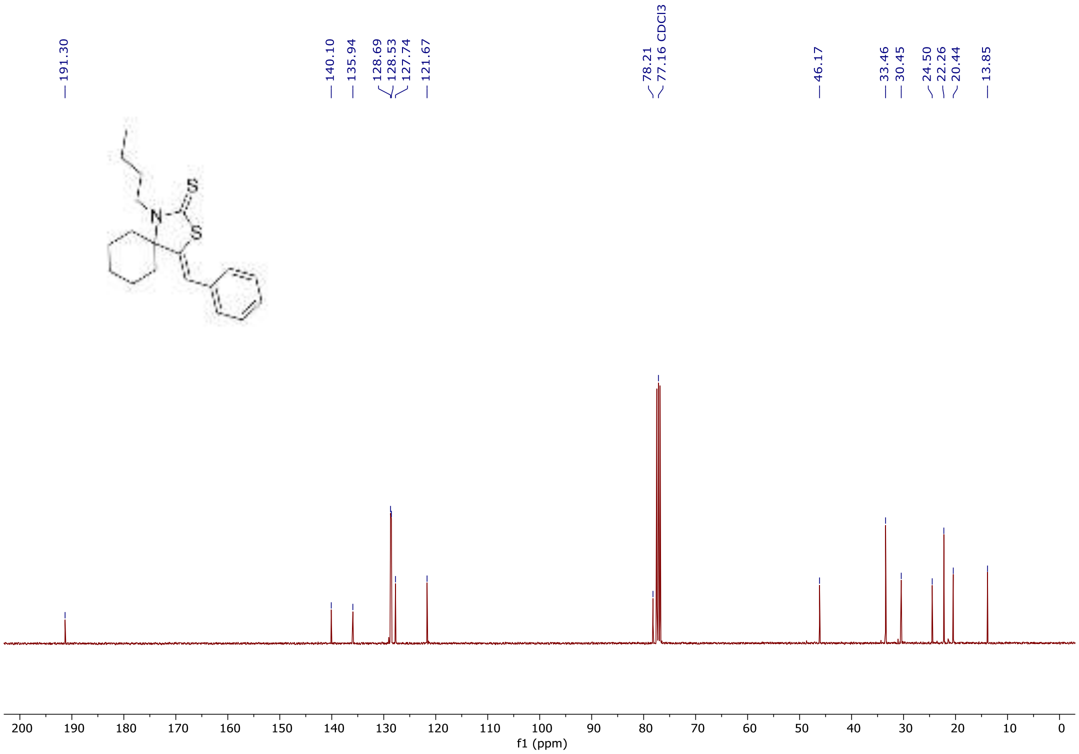

Figure S49: $^{13}C\{1H\}$-NMR (101 MHz, $CDCl_3$) spectrum of compound **2h**.

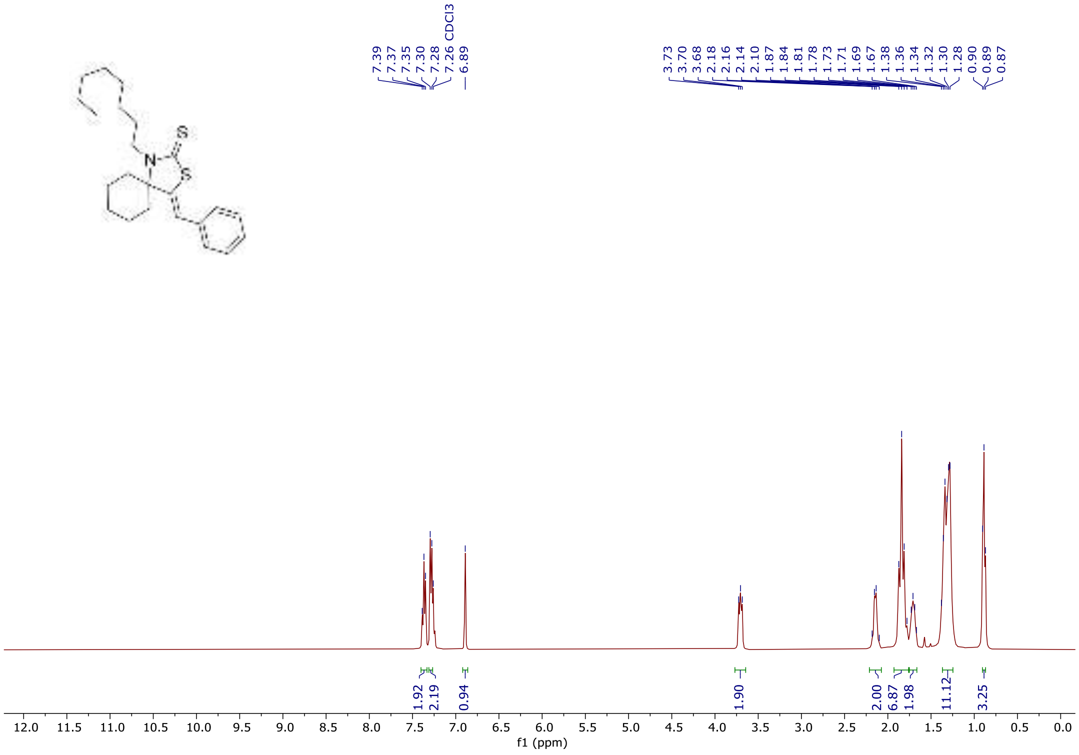

Figure S50: ^{1}H-NMR (400 MHz, $CDCl_3$) spectrum of compound **2i**.

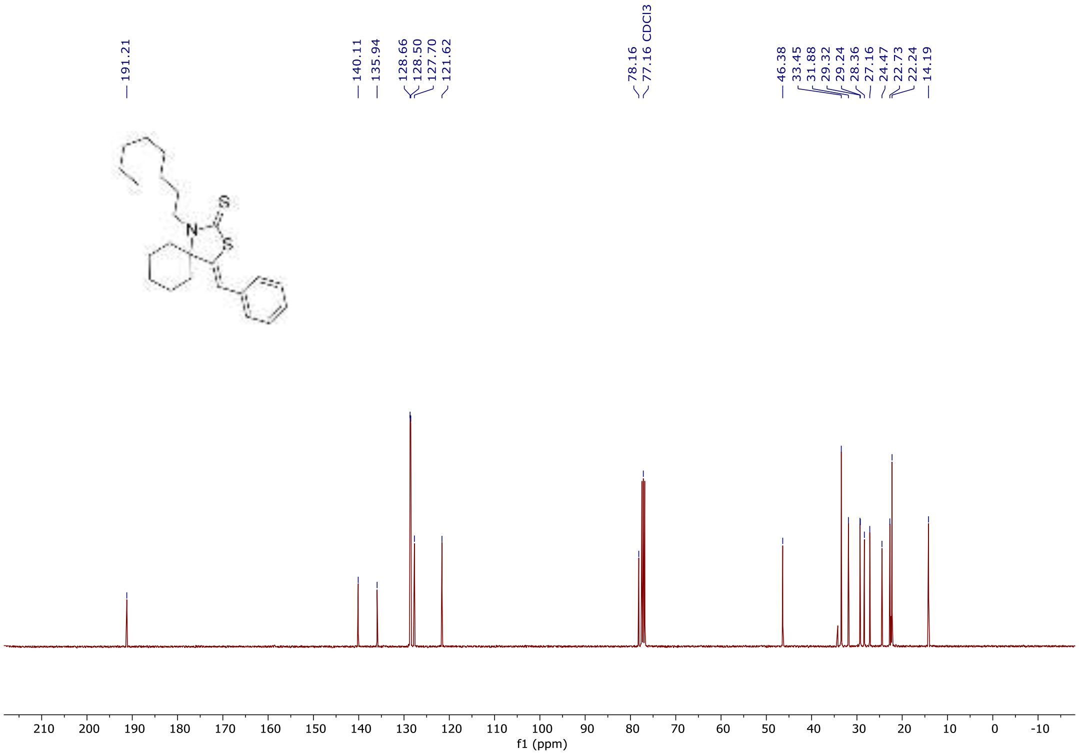

Figure S51: $^{13}C\{1H\}$-NMR (101 MHz, $CDCl_3$) spectrum of compound **2i**.

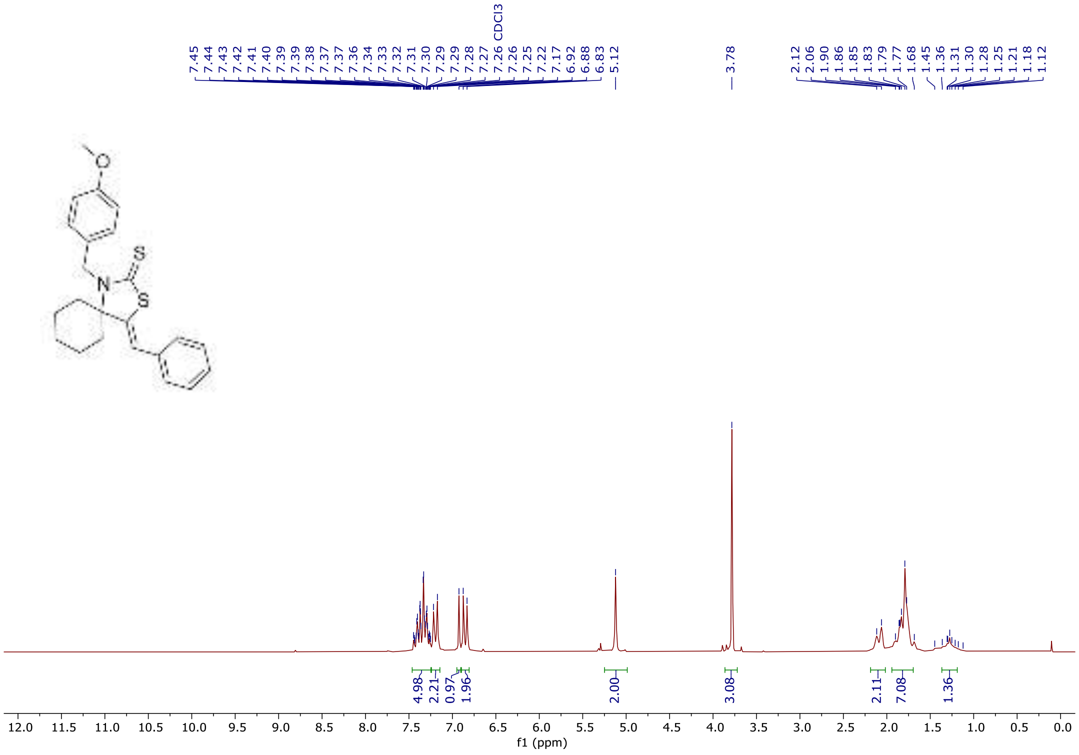

Figure S52: ^{1}H-NMR (200 MHz, $CDCl_3$) spectrum of compound **2j**.

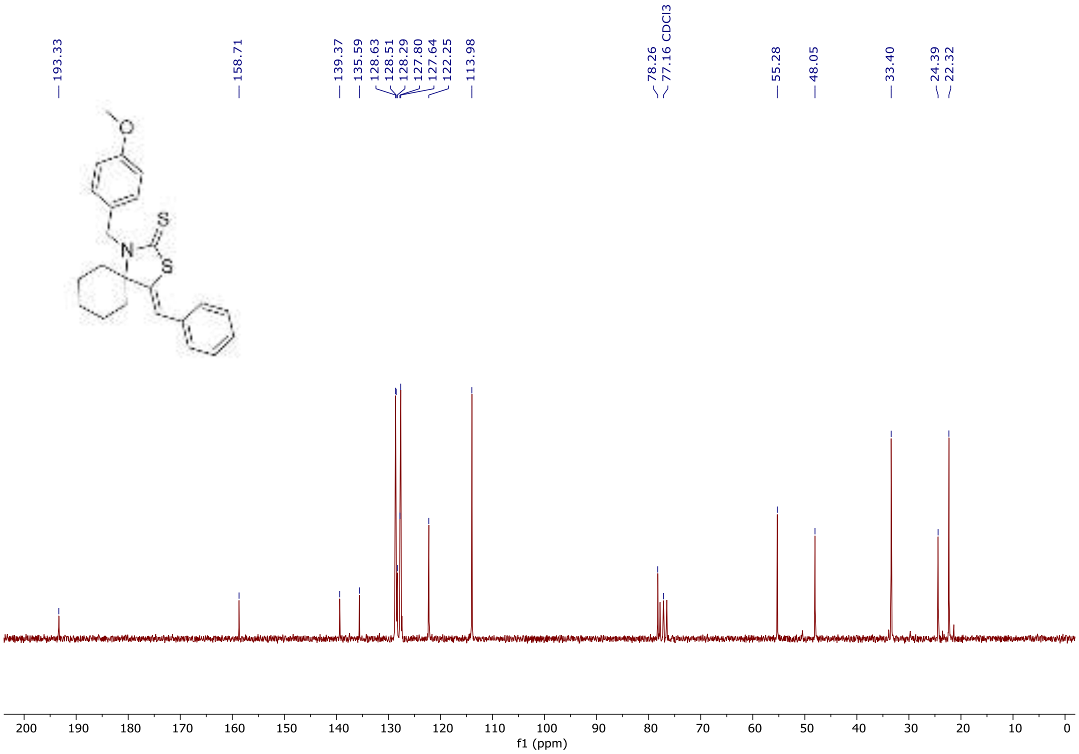

Figure S53: $^{13}C\{1H\}$-NMR (50 MHz, $CDCl_3$) spectrum of compound **2j**.

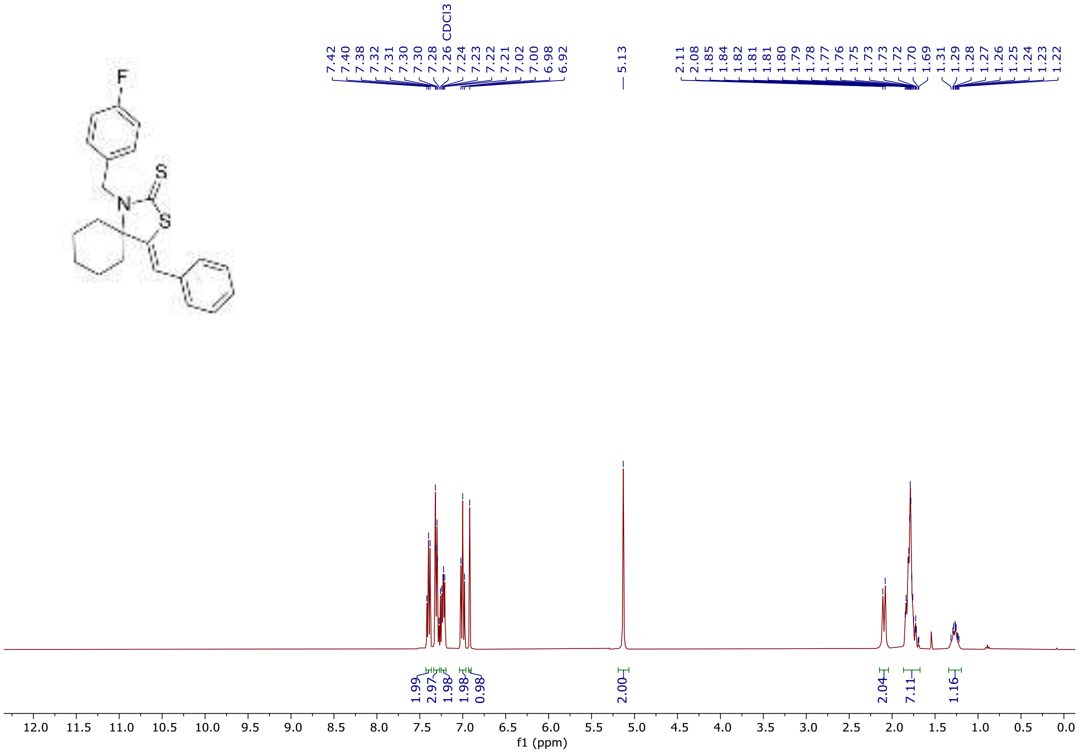

Figure S54: ^{1}H-NMR (400 MHz, $CDCl_3$) spectrum of compound **2k**.

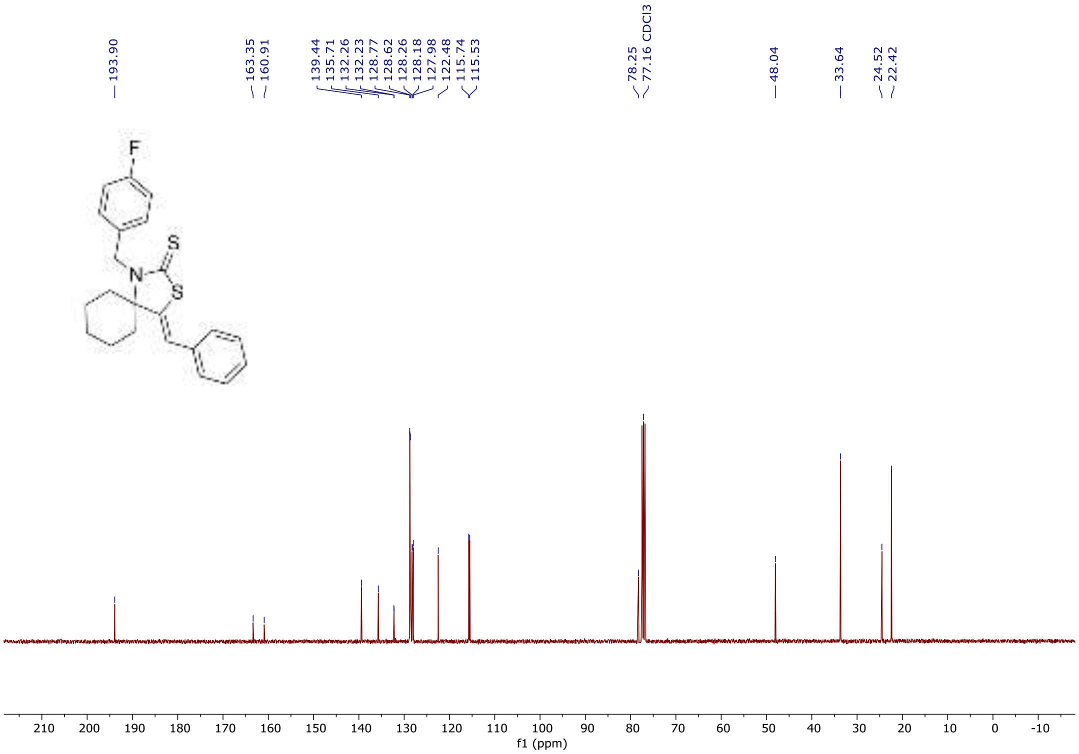

Figure S55: $^{13}C\{1H\}$-NMR (101 MHz, $CDCl_3$) spectrum of compound **2k**.

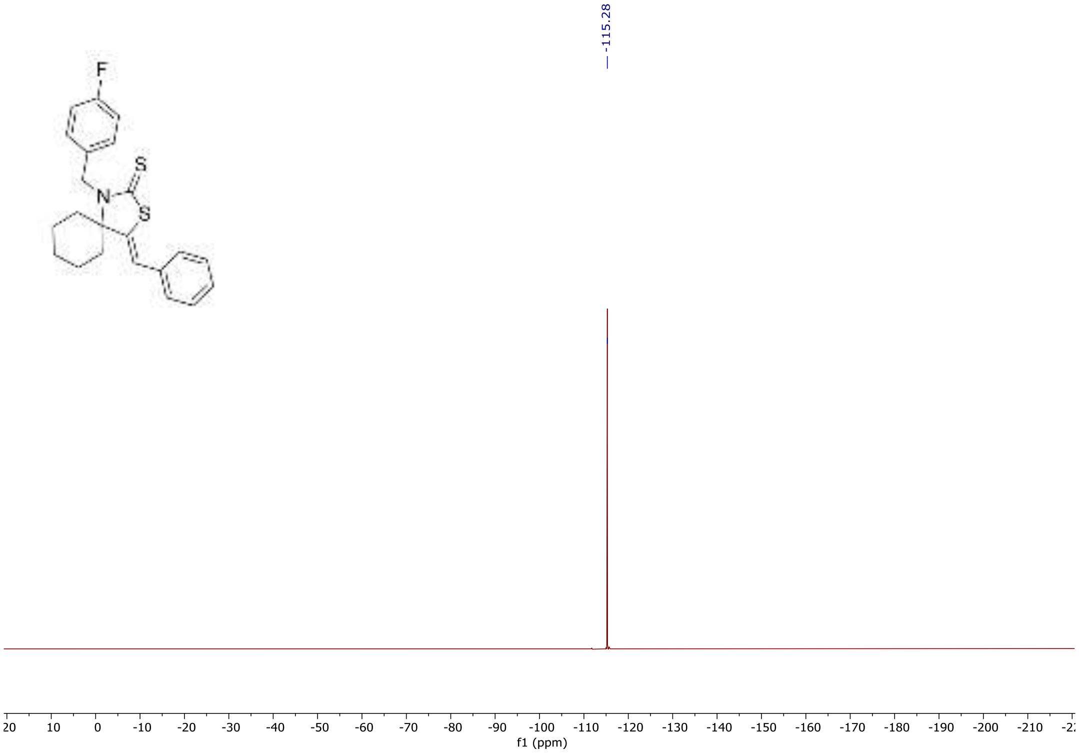

Figure S56: ^{19}F-NMR (376 MHz, $CDCl_3$) spectrum of compound **2k**.

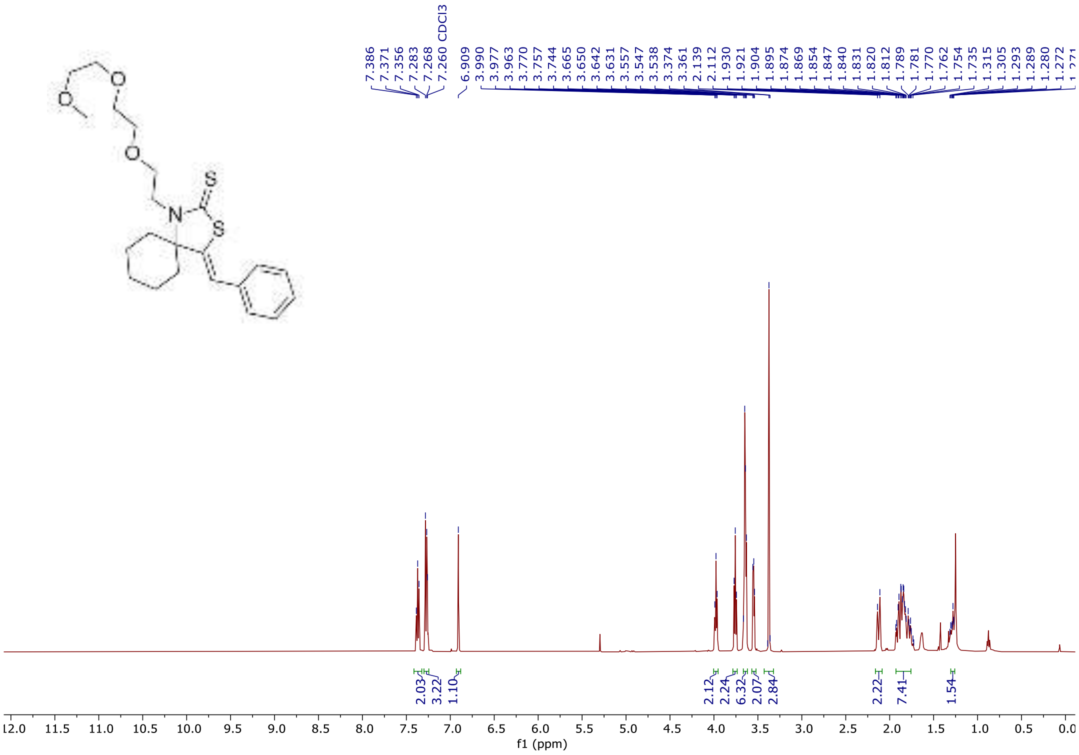

Figure S57: ^{1}H-NMR (400 MHz, $CDCl_3$) spectrum of compound **2l**.

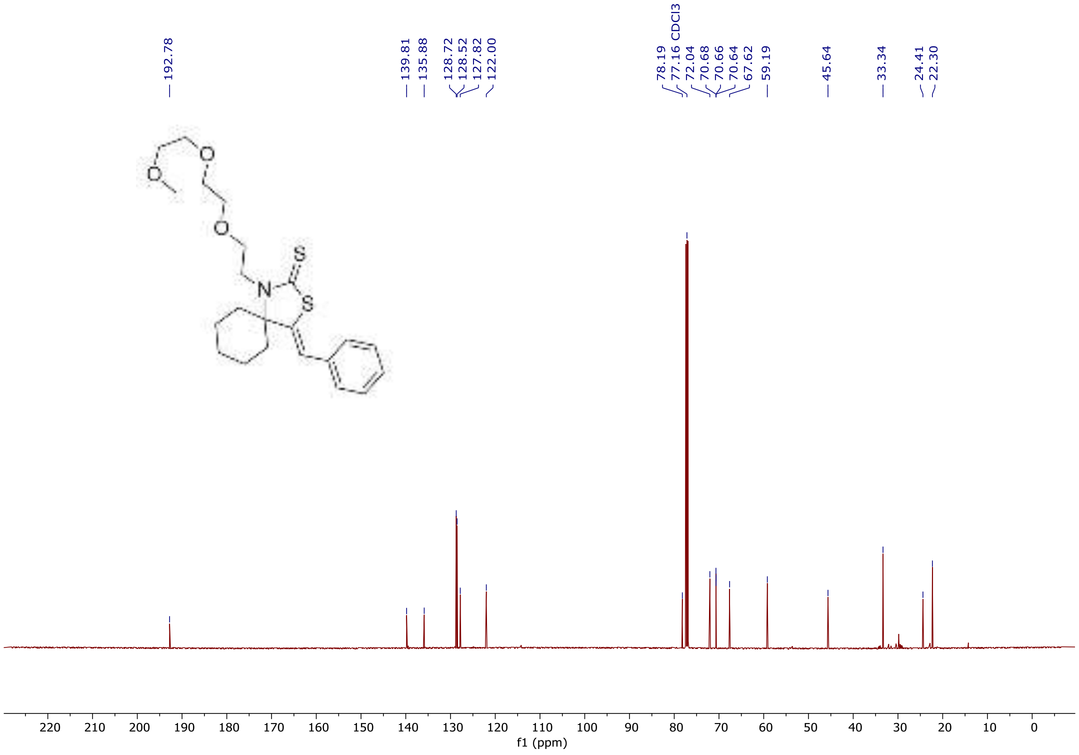

Figure S58: ^{13}C{1H}-NMR (101 MHz, $CDCl_3$) spectrum of compound **2l**.

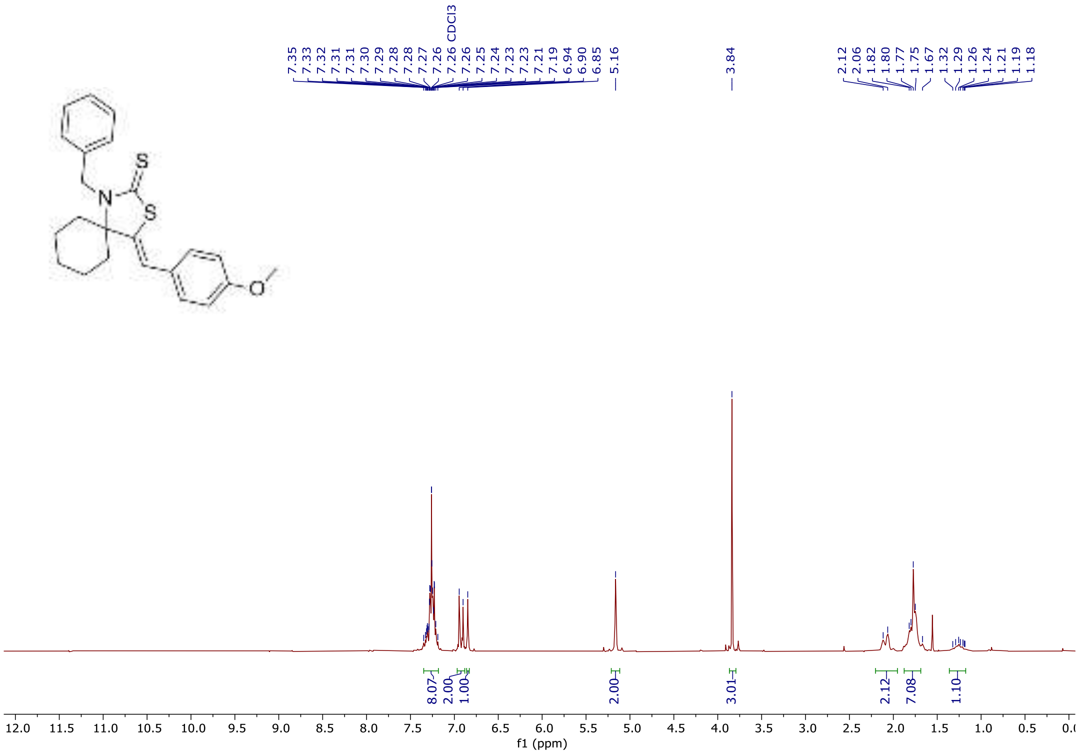

Figure S59: ^{1}H-NMR (200 MHz, $CDCl_3$) spectrum of compound **2n**.

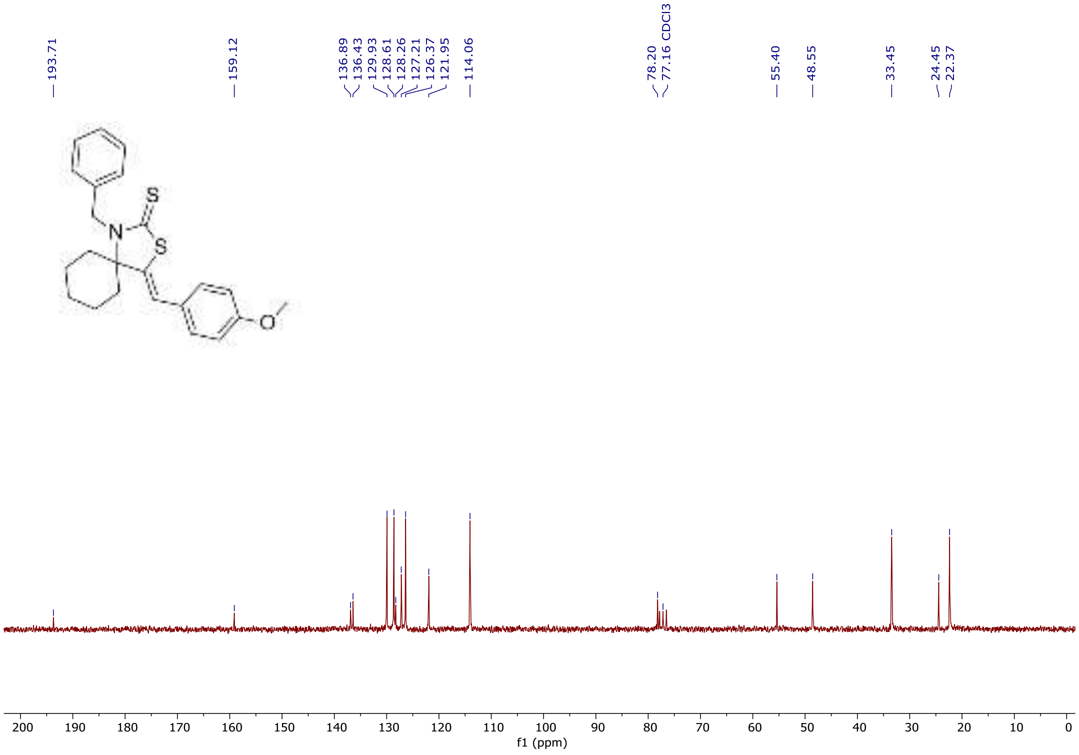

Figure S60: ^{13}C{1H}-NMR (50 MHz, $CDCl_3$) spectrum of compound **2n**.

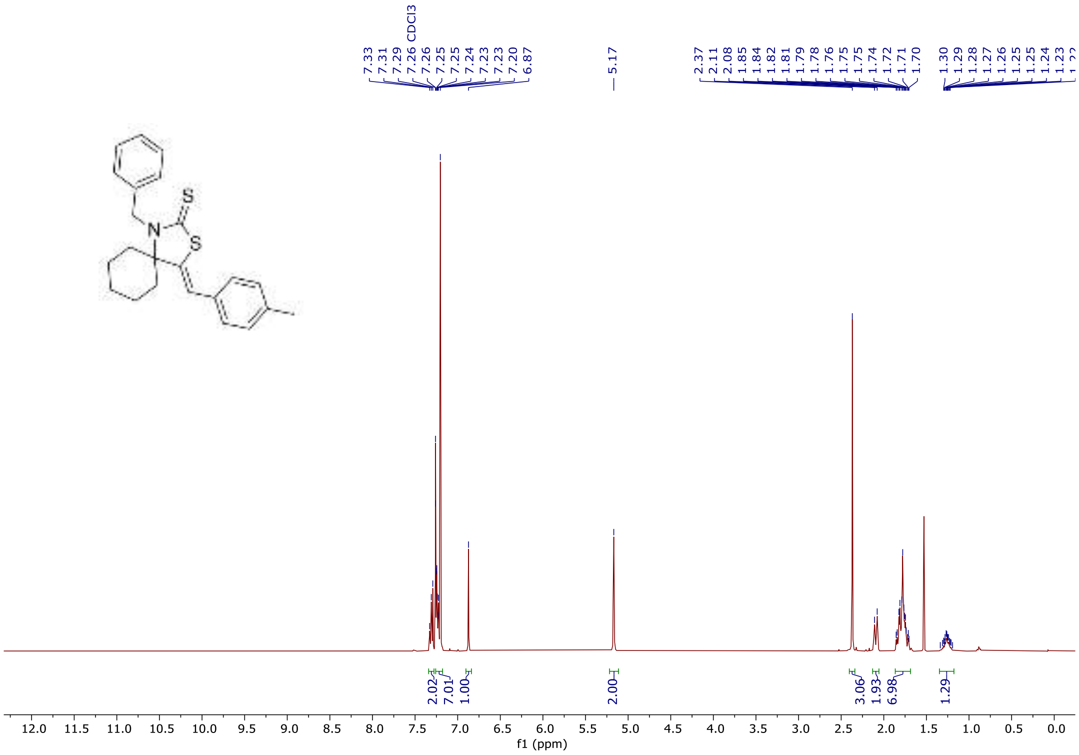

Figure S61: ^{1}H-NMR (400 MHz, $CDCl_3$) spectrum of compound **2o**.

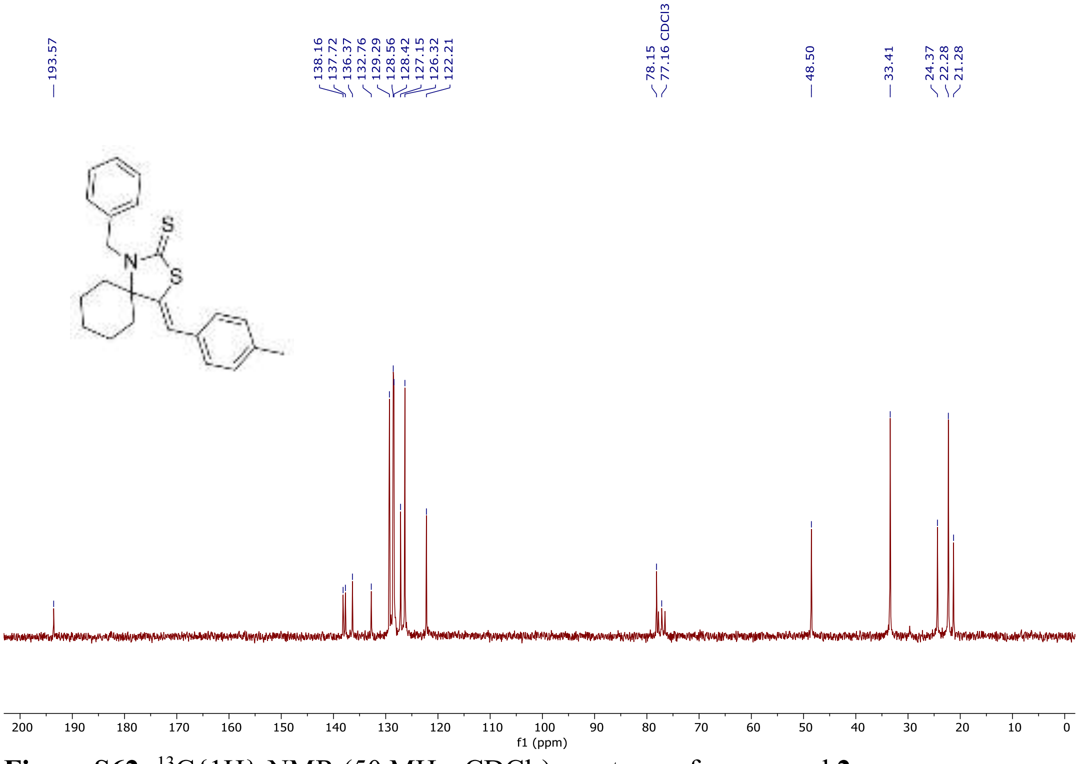

Figure S62: ^{13}C{1H}-NMR (50 MHz, $CDCl_3$) spectrum of compound **2o**.

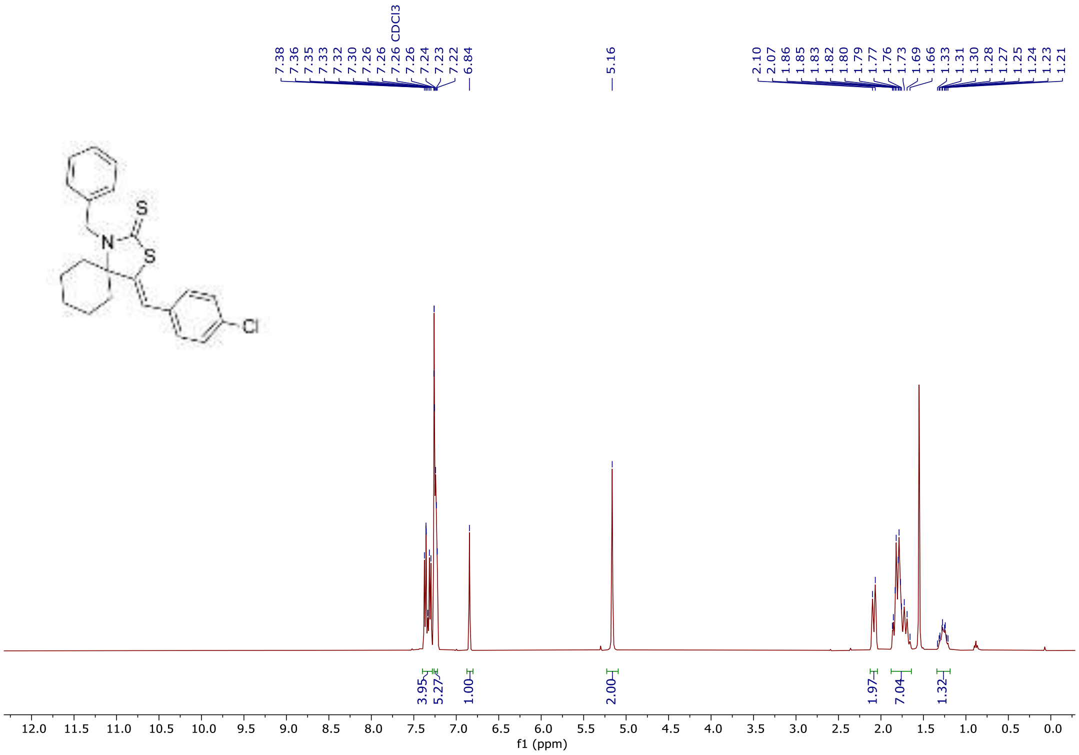

Figure S63: ^{1}H-NMR (400 MHz, $CDCl_3$) spectrum of compound **2p**.

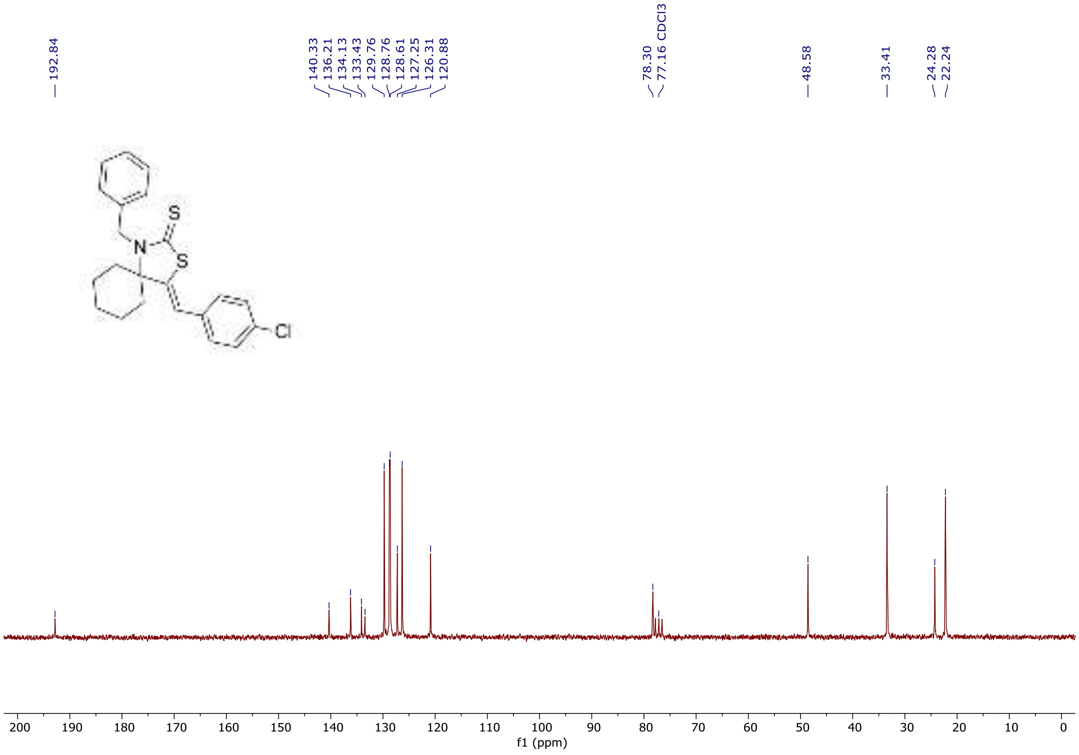

Figure S64: ^{13}C{1H}-NMR (50 MHz, $CDCl_3$) spectrum of compound **2p**.

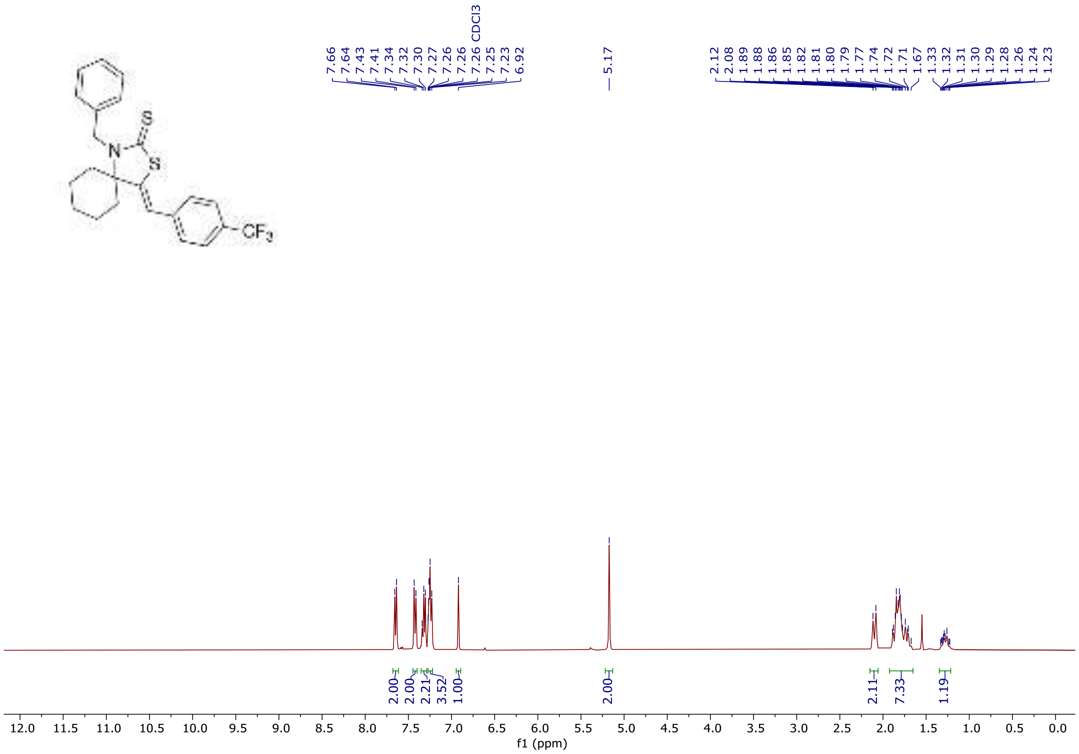

Figure S65: ^{1}H-NMR (400 MHz, $CDCl_3$) spectrum of compound **2q**.

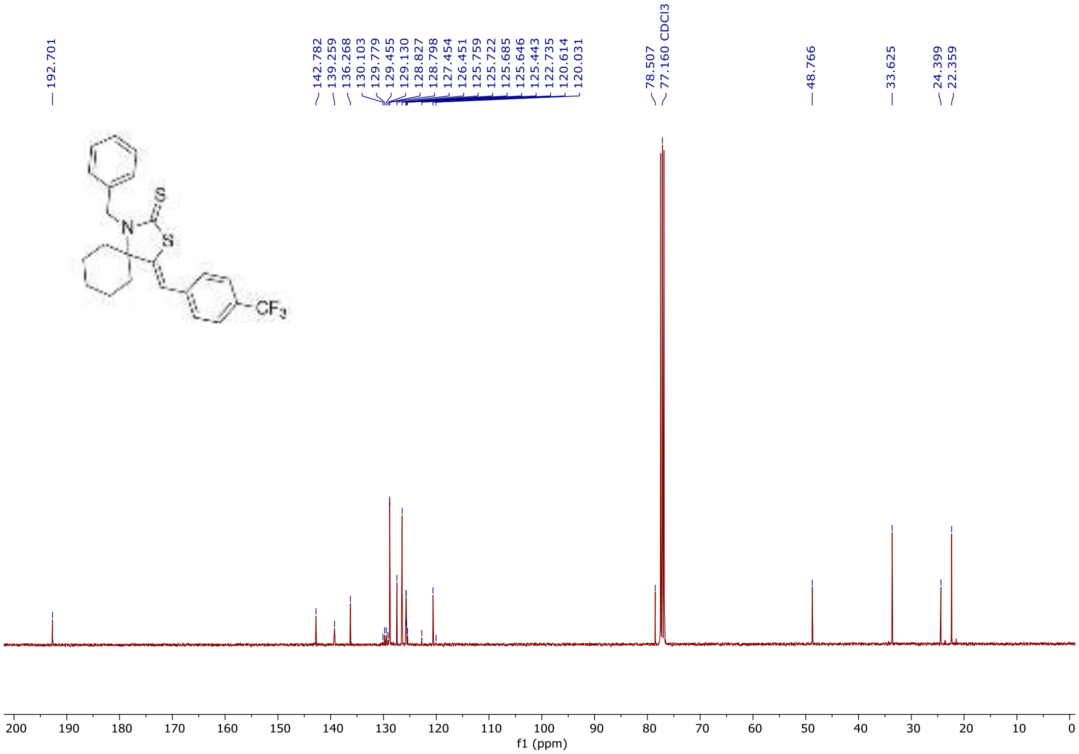

Figure S66: $^{13}C\{1H\}$-NMR (101 MHz, $CDCl_3$) spectrum of compound **2q**.

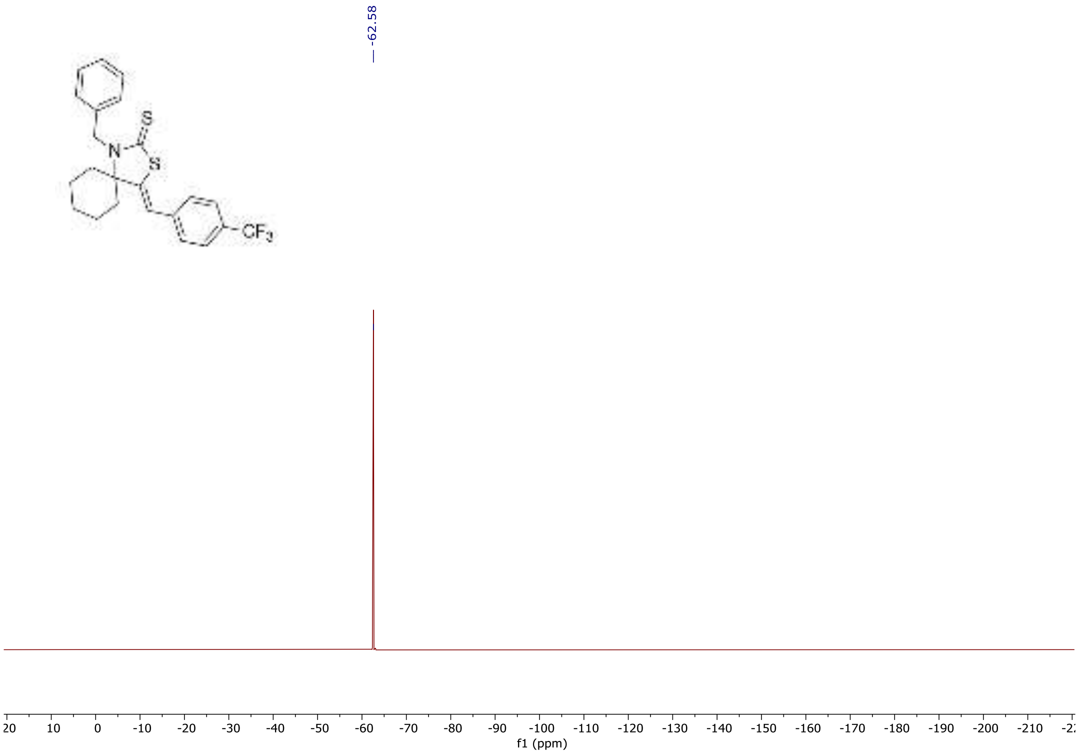

Figure S67: ^{19}F-NMR (376 MHz, $CDCl_3$) spectrum of compound **2q**.

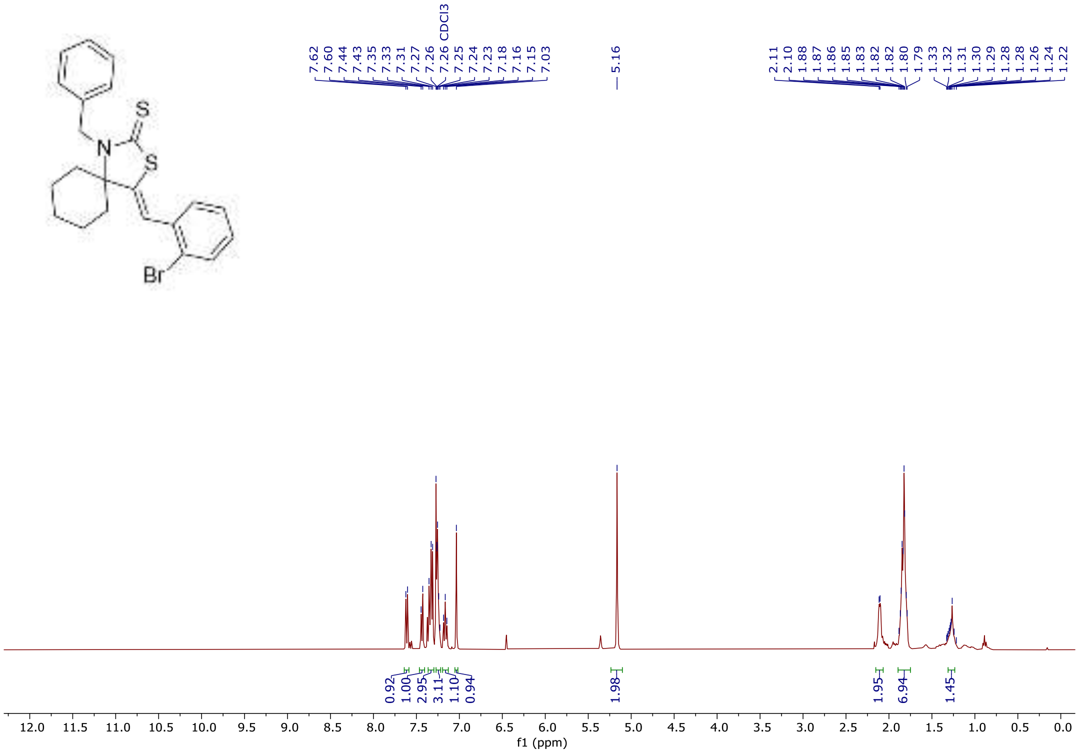

Figure S68: ^{1}H-NMR (400 MHz, $CDCl_3$) spectrum of compound **2r**.

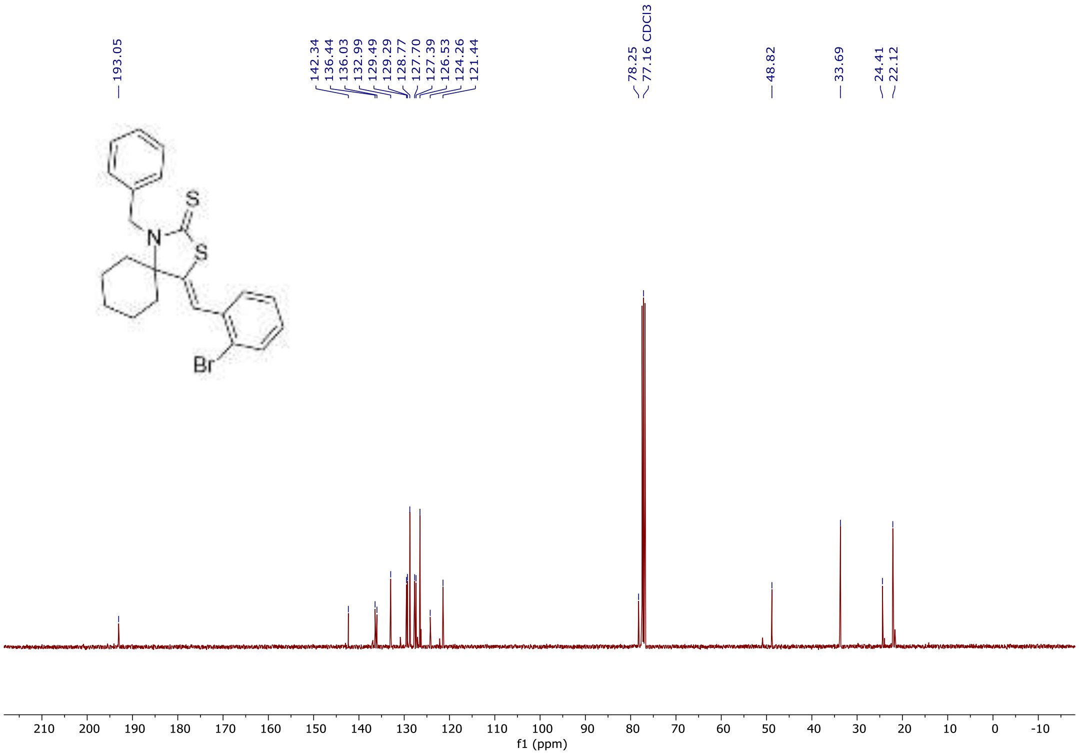

Figure S69: $^{13}C\{1H\}$-NMR (101 MHz, $CDCl_3$) spectrum of compound **2r**.

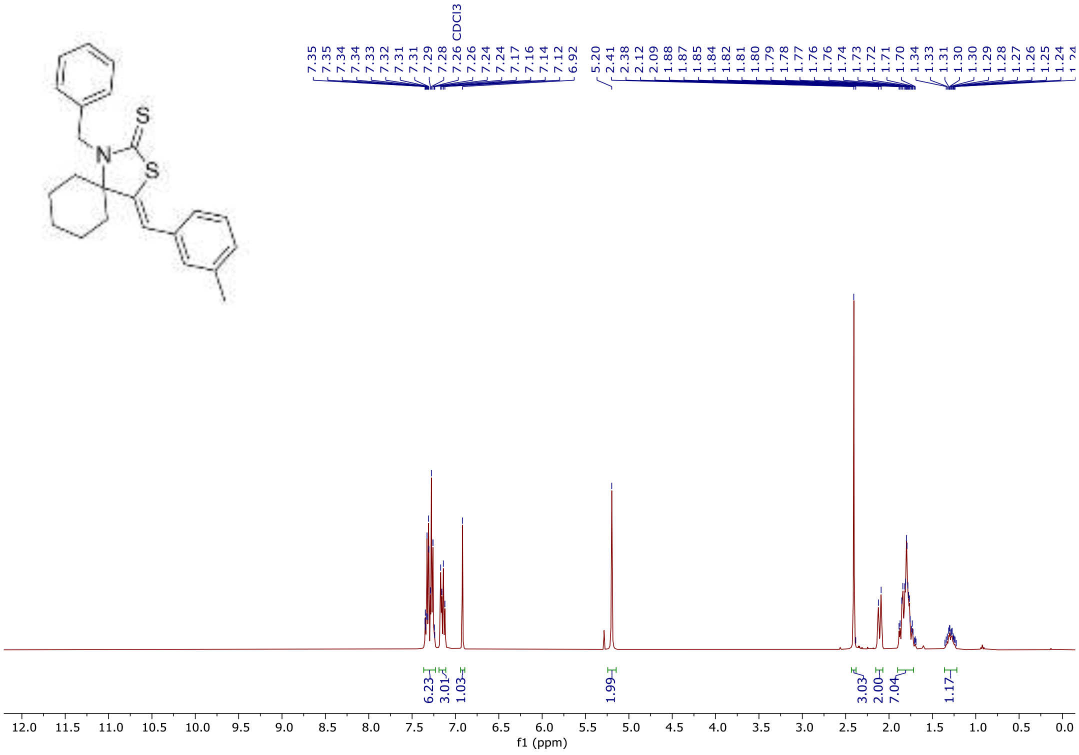

Figure S70: ^{1}H-NMR (400 MHz, $CDCl_3$) spectrum of compound **2s**.

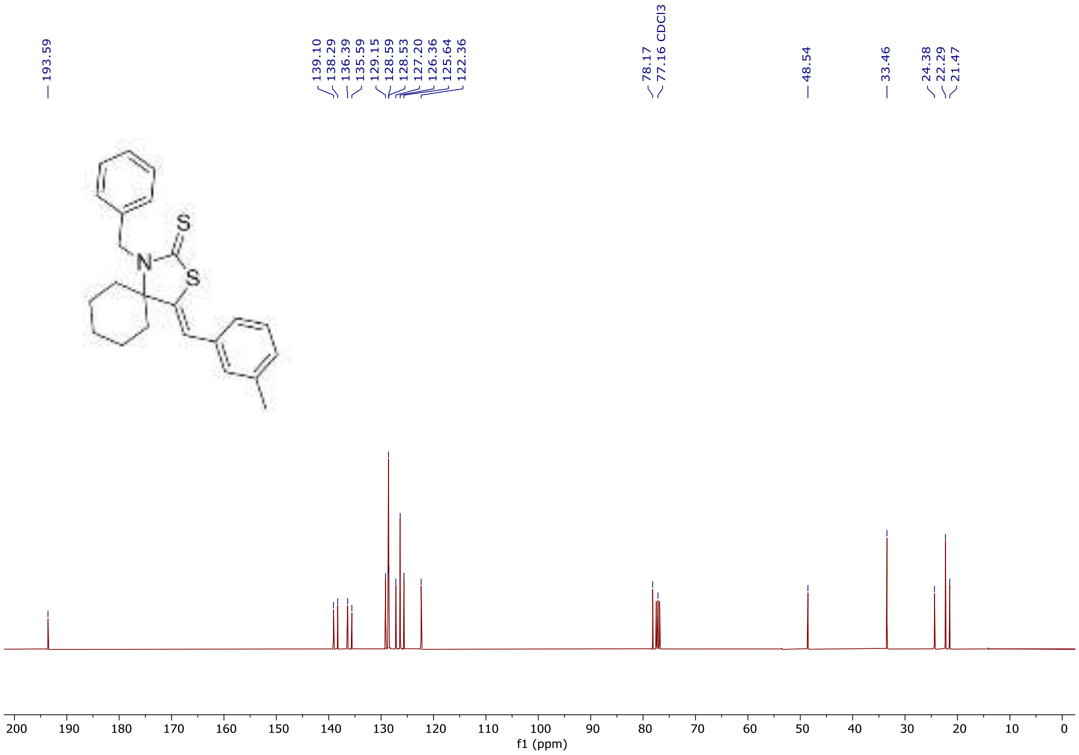

Figure S71: $^{13}C\{1H\}$-NMR (101 MHz, $CDCl_3$) spectrum of compound **2s**.

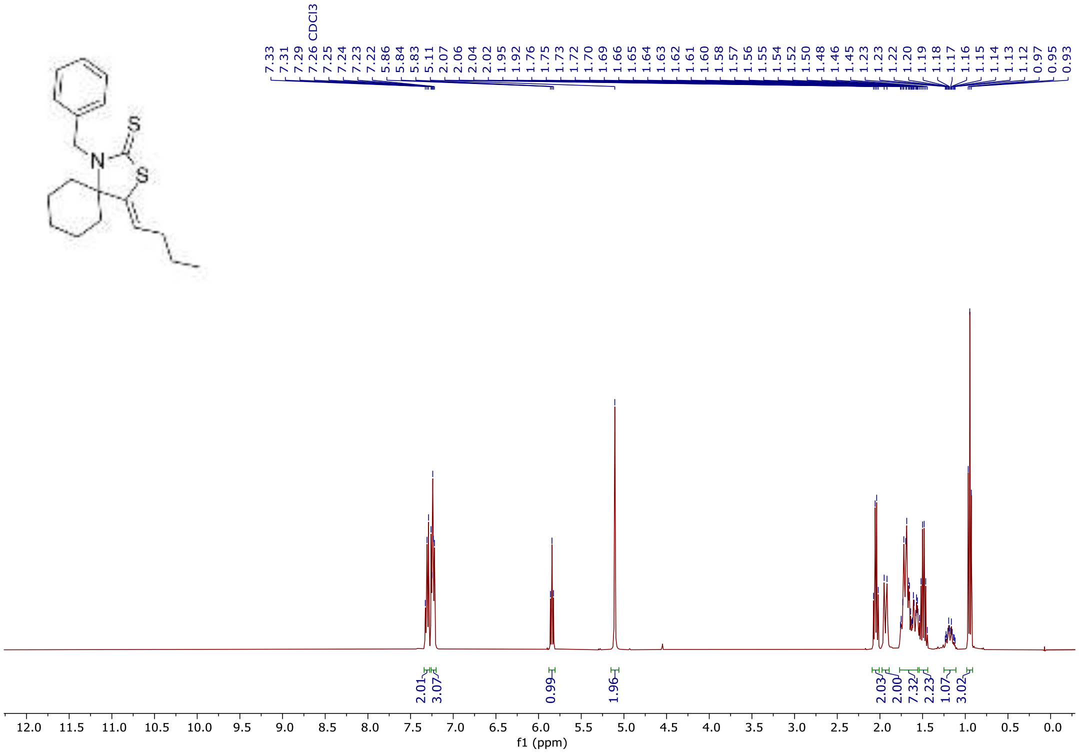

Figure S72: ^{1}H-NMR (400 MHz, $CDCl_3$) spectrum of compound **2t**.

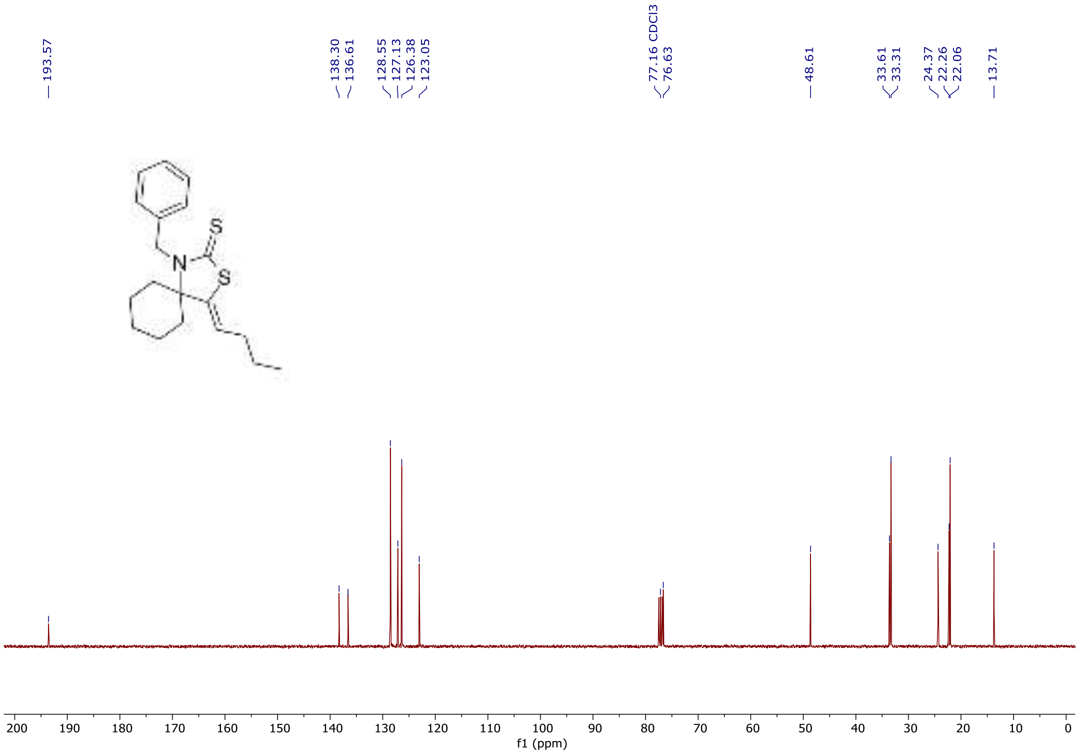

Figure S73: $^{13}C\{1H\}$-NMR (101 MHz, $CDCl_3$) spectrum of compound **2t**.

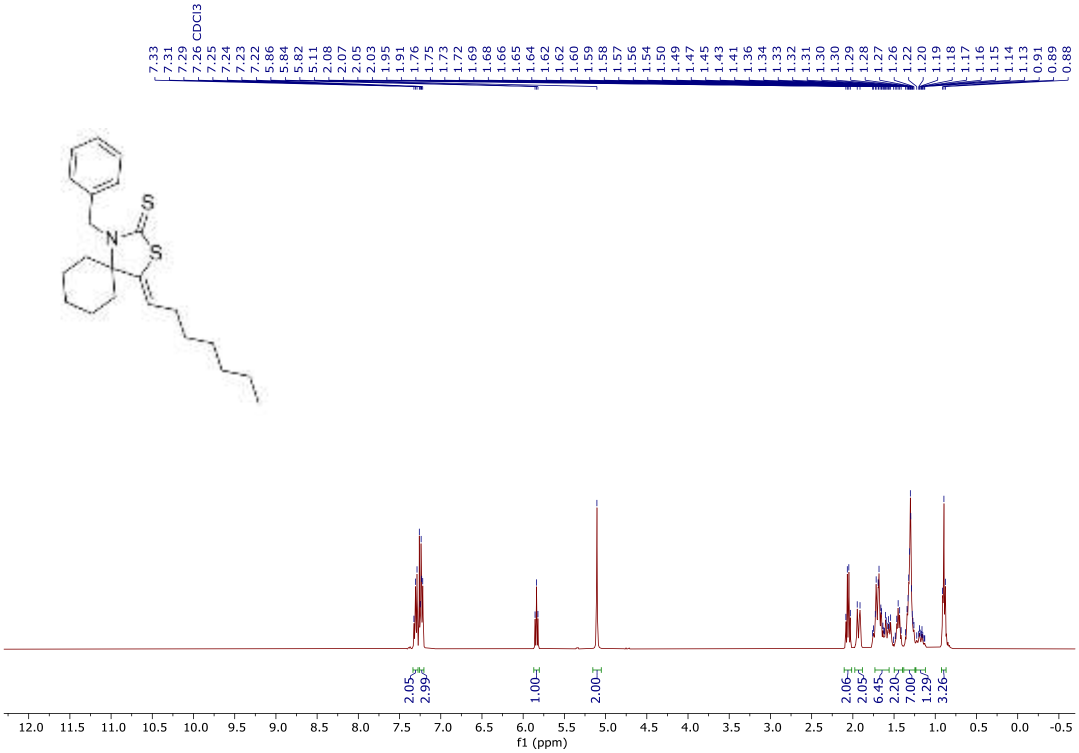

Figure S74: ^{1}H-NMR (400 MHz, $CDCl_3$) spectrum of compound **2u**.

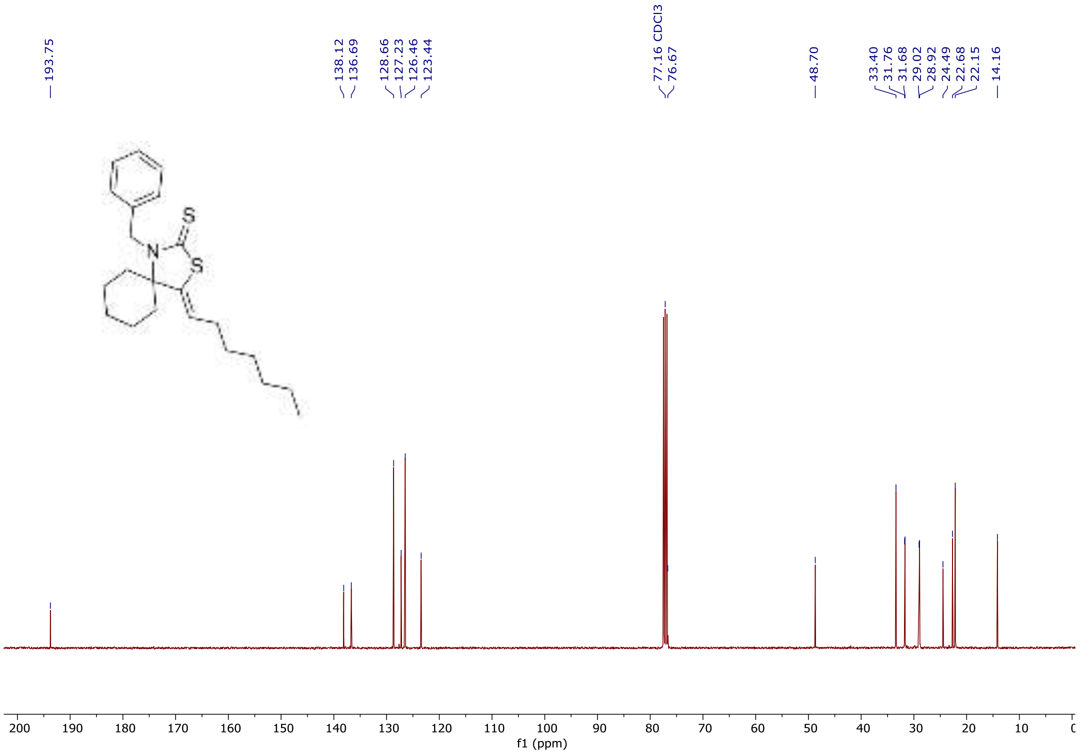

Figure S75: $^{13}C\{1H\}$-NMR (101 MHz, $CDCl_3$) spectrum of compound **2u**.

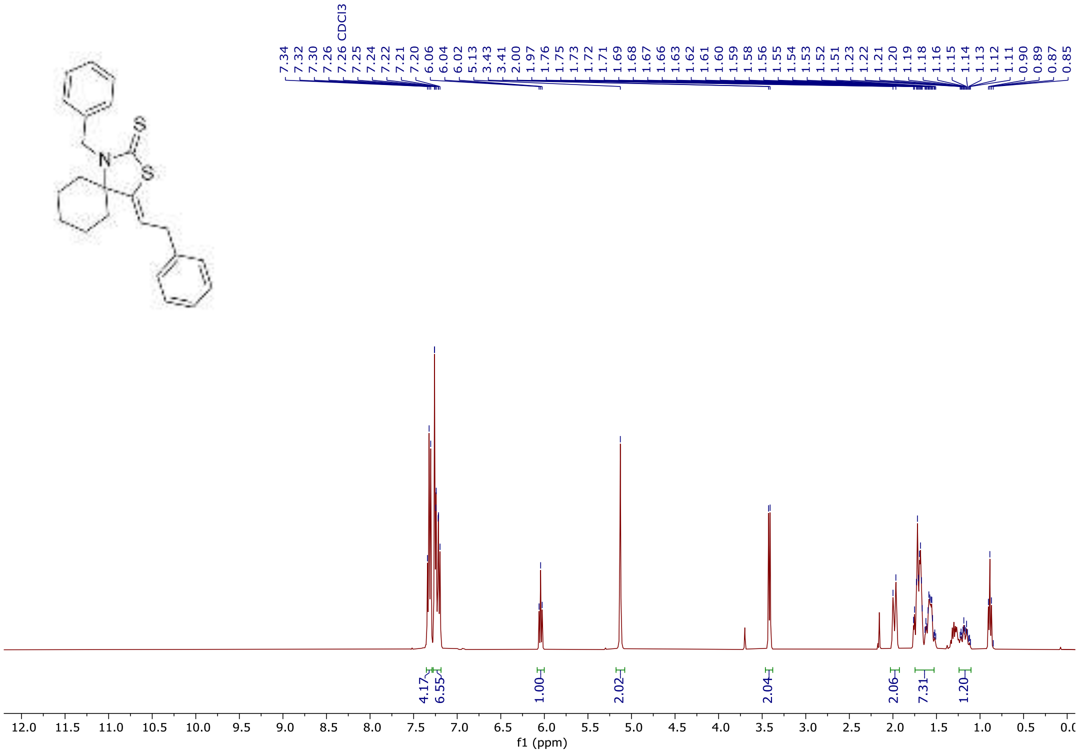

Figure S76: ^{1}H-NMR (400 MHz, $CDCl_3$) spectrum of compound **2v**.

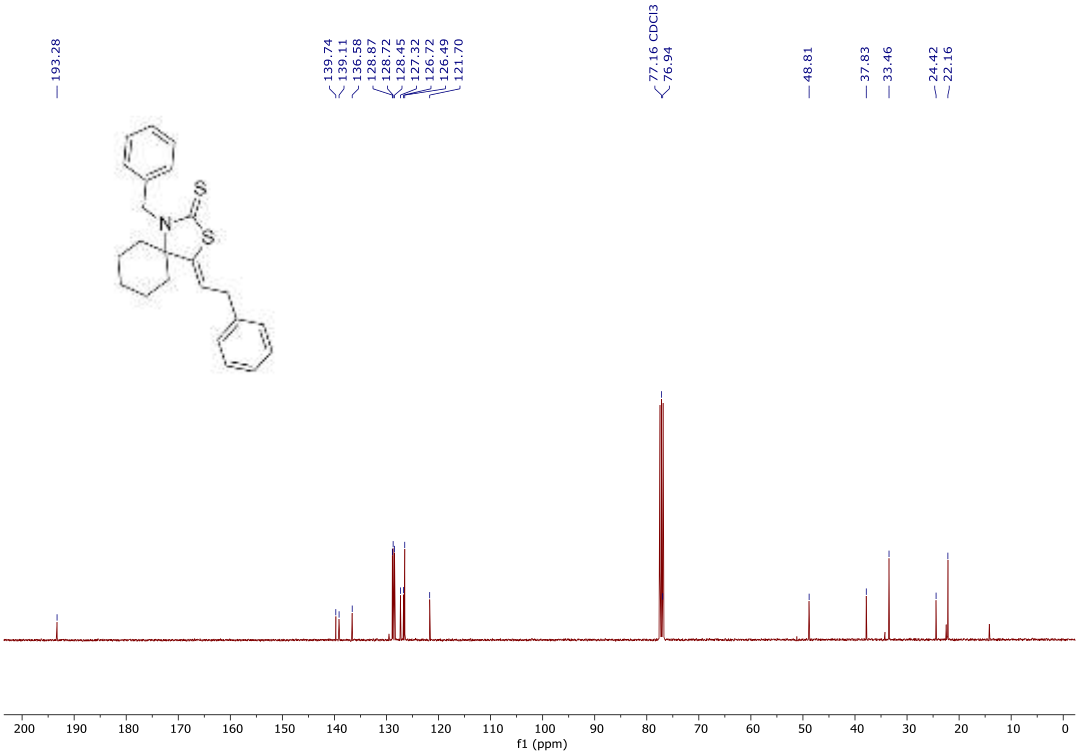

Figure S77: $^{13}C\{1H\}$-NMR (101 MHz, $CDCl_3$) spectrum of compound **2v**.

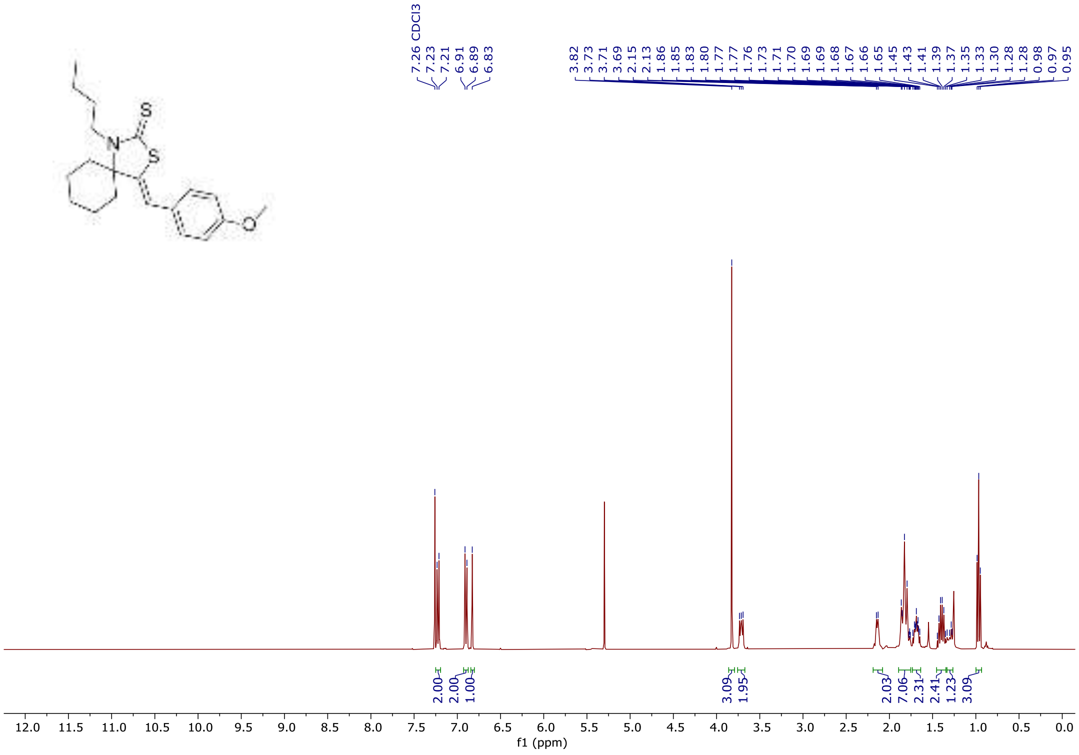

Figure S78: ^{1}H-NMR (400 MHz, $CDCl_3$) spectrum of compound **2w**.

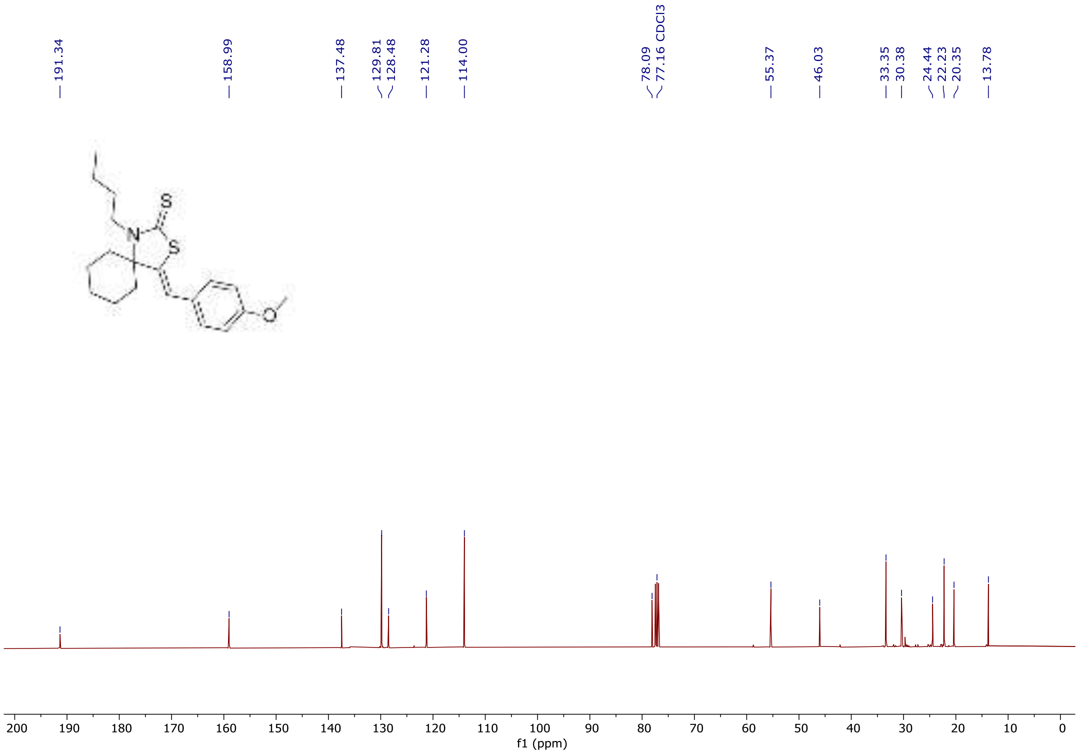

Figure S79: $^{13}C\{1H\}$-NMR (101 MHz, $CDCl_3$) spectrum of compound **2w**.

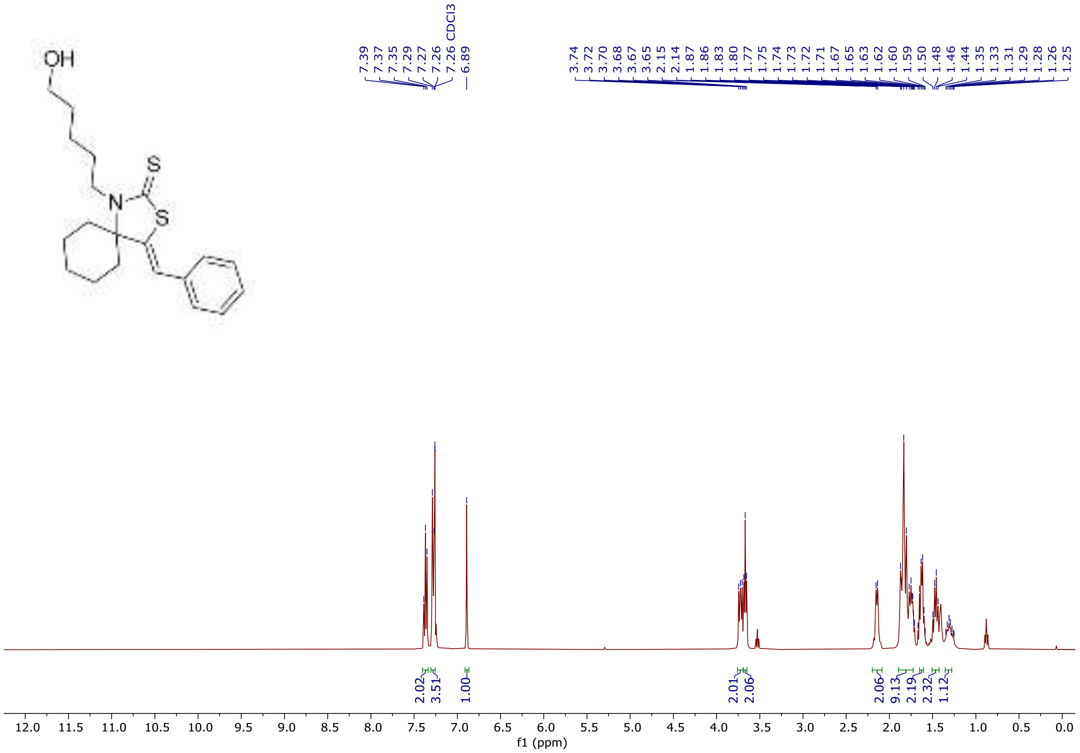

Figure S80: ^{1}H-NMR (400 MHz, $CDCl_3$) spectrum of compound **2x**.

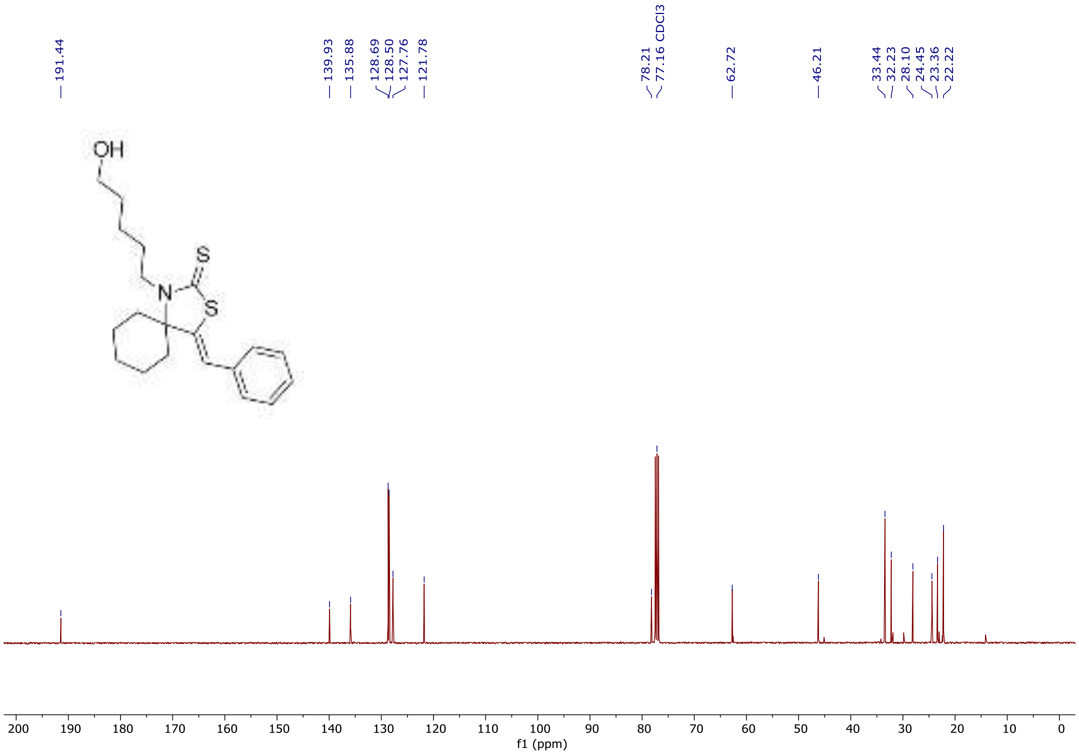

Figure S81: $^{13}C\{1H\}$-NMR (101 MHz, $CDCl_3$) spectrum of compound **2x**.

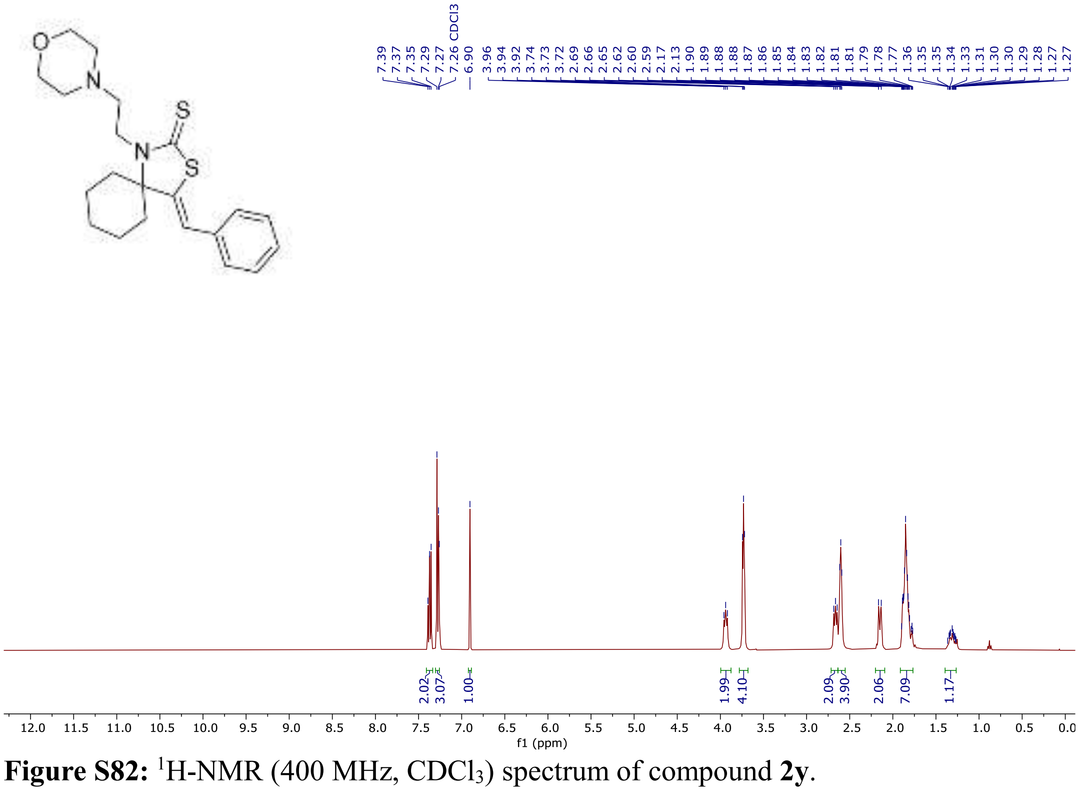

Figure S82: ^{1}H-NMR (400 MHz, $CDCl_3$) spectrum of compound **2y**.

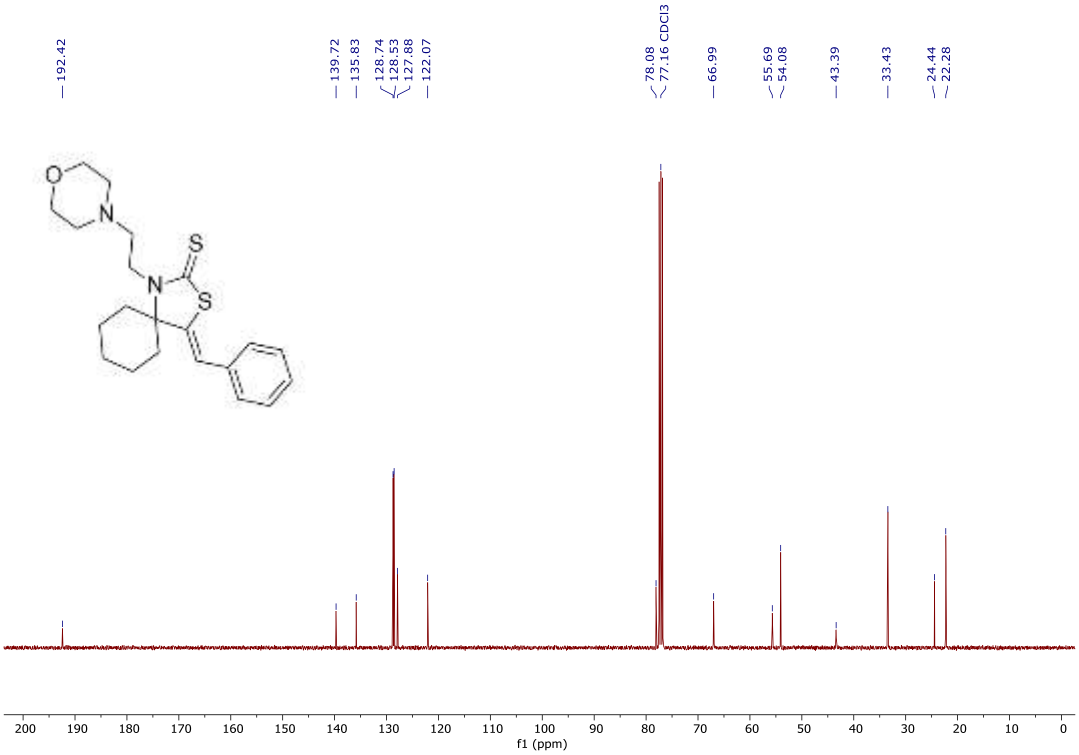

Figure S83: $^{13}C\{1H\}$-NMR (101 MHz, $CDCl_3$) spectrum of compound **2y**.

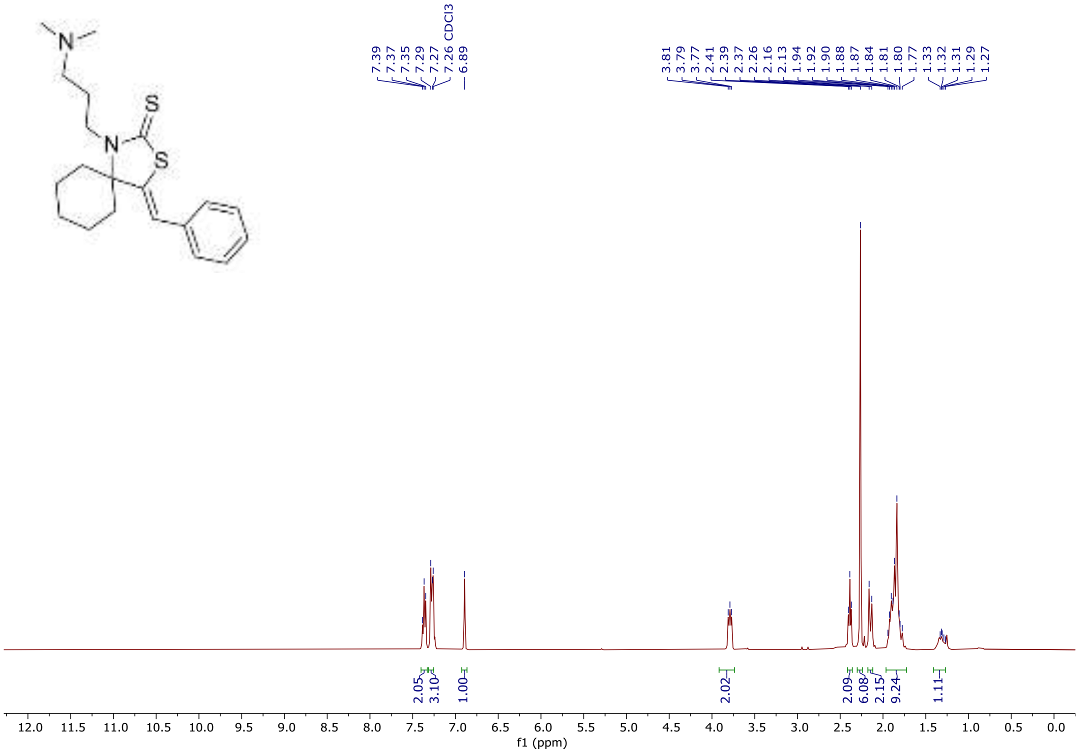

Figure S84: ^{1}H-NMR (400 MHz, $CDCl_3$) spectrum of compound **2z**.

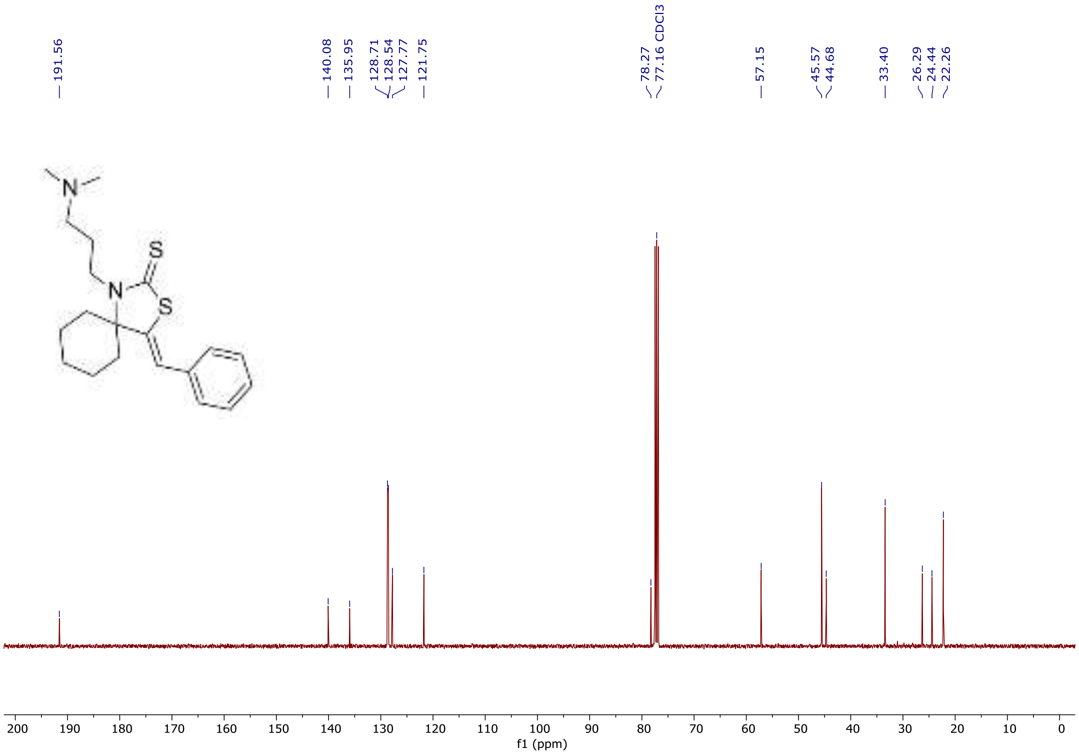

Figure S85: $^{13}C\{1H\}$-NMR (101 MHz, $CDCl_3$) spectrum of compound **2z**.

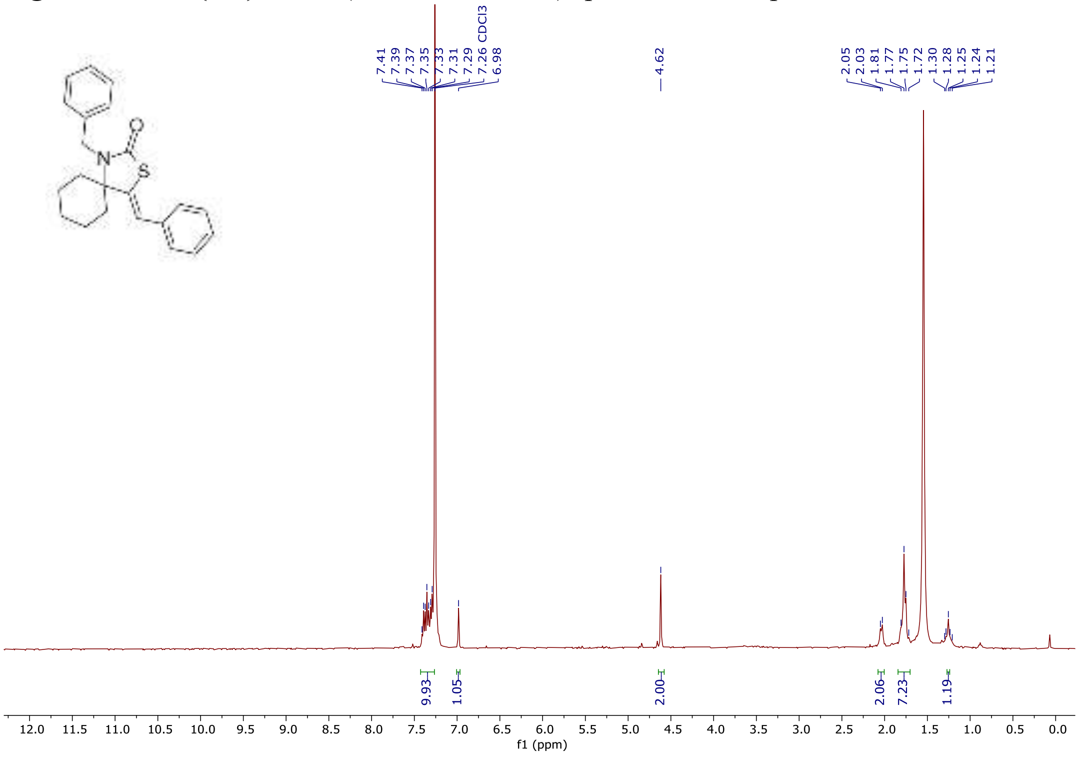

Figure S86: ^{1}H-NMR (200 MHz, $CDCl_3$) spectrum of compound **3**.

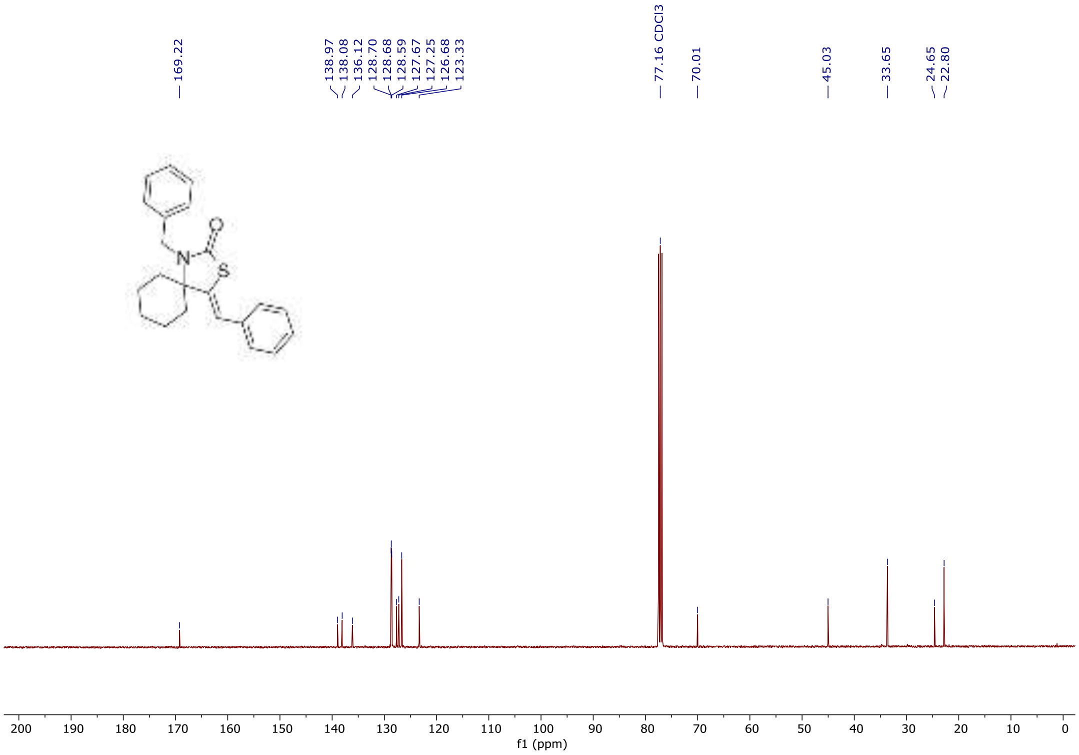

Figure S87: $^{13}C\{1H\}$-NMR (101 MHz, $CDCl_3$) spectrum of compound **3**.

9. Optimized molecular structures with DFT

Cartesian coordinates and energies (in Hartrees) of optimized molecular structures from DFT calculations are provided in the following format:

**Structure name or state number (relevant figure number)**, Stoichiometry<br>Electronic energy | Electronic energy + Zero-point correction | Enthalpy | Gibbs free energy<br>Element | xyz coordinate<br>--

Note that the enthalpy and Gibbs free energies are calculated assuming 1 atm and 25 °C.

State 1 with methyl groups (Figure 4) C7H13N
-329.186582394 -328.999030 -328.987626 -329.034360
C -0.834643 1.277910 1.123805
C -0.861062 0.908545 -1.365621
C -2.598167 -1.030035 0.062596
C 3.587970 -0.403374 0.020870
C -0.490714 0.291637 -0.001924
C 0.961092 0.035379 0.015285
H -2.925998 -0.801256 -0.960806
H -0.279315 2.207550 0.989472
H -1.900775 1.514880 1.114649
H -0.574829 0.840175 2.087688
N -1.155775 -0.998273 0.263214
H -0.261075 1.801066 -1.551232
H -0.674305 0.193588 -2.170572
H -1.915072 1.195264 -1.384617
H -3.083538 -0.326650 0.741900
H -2.958804 -2.026653 0.320978
H -0.709990 -1.714154 -0.299454
C 2.146882 -0.162771 0.010311
H 3.819753 -1.323351 0.560342
H 3.975573 -0.495486 -0.995170
H 4.110652 0.419203 0.512401

State 2 with methyl groups (Figure 4) C8H13NS2
-1163.65564739 -1163.460403 -1163.444088 -1163.507348
C 3.752311 -0.330719 0.022721
C 1.843597 -0.809743 1.591648
C 1.820196 -2.626845 -0.752334
C 0.736882 3.470319 -0.387973
C 2.223656 -0.351063 0.169438
C 1.707109 1.015099 -0.035117
H 1.365403 -3.055863 0.150963
H 4.188847 0.357629 0.748331
H 4.167695 -1.324722 0.203120
H 4.020406 -0.009621 -0.983806
N 1.645383 -1.188294 -0.899133
H 2.214462 -0.095173 2.328616
H 0.757846 -0.881434 1.694280
H 2.283758 -1.785461 1.809266
H 2.883475 -2.874891 -0.750012
H 1.377809 -3.116471 -1.621029
H 0.659812 -0.967161 -0.989816
C 1.273932 2.126576 -0.188385

H 1.117623 3.903996 -1.314248
H -0.353621 3.443402 -0.446574
H 1.017167 4.125017 0.438929
C -3.089859 -0.114962 0.067740
S -4.522740 -0.704337 -0.033682
S -1.653842 0.474765 0.164900

TS1 with methyl groups (Figure 4) C8H13NS2
-1163.63739425 -1163.440926 -1163.426348 -1163.482097
C 0.550469 1.979257 1.313482
C 1.496847 2.243849 -1.002531
C -1.607265 1.850436 -0.621790
C 2.972281 -2.175003 0.416119
C 0.774273 1.289489 -0.037227
C 1.588321 0.078066 0.140187
H -1.281706 2.831981 -0.974682
H 1.525562 2.180106 1.758284
H 0.030407 2.930481 1.182625
H -0.019281 1.342450 1.988001
N -0.530026 0.861298 -0.648764
H 2.461974 2.522461 -0.576708
H 1.676365 1.756598 -1.963065
H 0.915744 3.154840 -1.160897
H -1.979526 1.928276 0.400415
H -2.416235 1.485862 -1.255295
H -0.343066 0.555893 -1.602836
C 2.237602 -0.923773 0.269050
H 2.348689 -3.006327 0.080892
H 3.886224 -2.164613 -0.179692
H 3.239646 -2.345476 1.460079
C -1.261030 -0.836966 -0.034676
S -1.863070 -0.665831 1.444962
S -1.064655 -1.848779 -1.274428

State 3 with methyl groups (Figure 4) C8H13NS2
-1163.64202583 -1163.443707 -1163.429160 -1163.484231
C -0.101425 2.079375 1.264498
C 0.470940 2.462680 -1.159913
C -2.253068 1.065158 -0.407236
C 3.590058 -0.971481 0.304818
C 0.247367 1.408632 -0.065698
C 1.450604 0.585483 0.067676
H -2.285683 2.068612 -0.828195
H 0.772812 2.649839 1.580511
H -0.936983 2.772690 1.150387
H -0.335283 1.337207 2.025225
N -0.892873 0.491955 -0.558764
H 1.301985 3.101057 -0.859328
H 0.735669 1.989019 -2.107672
H -0.410464 3.092464 -1.296085
H -2.503916 1.070976 0.650227
H -2.941013 0.409397 -0.939589
H -0.711230 0.340796 -1.556227
C 2.435567 -0.091481 0.176182
H 3.304121 -1.985859 0.019759
H 4.405658 -0.644428 -0.341567

H 3.945974 -0.990378 1.335880
C -0.866615 -1.002351 -0.037966
S -1.233066 -1.213195 1.553739
S -0.512691 -2.028752 -1.289748

State 4 with methyl groups (Figure 4) C14H25N3S2
-1508.93209726 -1508.546878 -1508.525222 -1508.597799
C 2.768598 -0.337110 2.301380
C 0.654918 1.023878 2.228334
C 0.649031 -1.954778 1.194309
C 3.533682 3.073518 -1.102108
C 1.734544 0.327963 1.388205
C 2.378057 1.318763 0.523186
H 0.053394 -1.711081 2.073535
H 3.295299 0.457058 2.831872
H 2.283362 -0.977798 3.040437
H 3.486683 -0.915711 1.723282
N 1.016811 -0.702713 0.488653
H 1.143294 1.747875 2.881319
H -0.047858 1.554934 1.585659
H 0.110508 0.310585 2.850918
H 1.552077 -2.497961 1.456993
H 0.059823 -2.552136 0.500111
H 0.106121 -0.269886 0.219936
C 2.906936 2.133964 -0.181051
H 3.259895 2.810380 -2.125970
H 3.205692 4.095170 -0.904398
H 4.620426 3.032353 -1.014867
C 1.732552 -1.012737 -0.859377
S 3.069220 -1.979658 -0.765215
S 0.959976 -0.308556 -2.150842
C -3.552482 1.406732 -0.922756
C -2.060358 1.252478 -0.514077
H -3.669133 1.363419 -2.008200
H -3.961414 2.361523 -0.583341
H -1.389520 1.269738 -1.374446
H -1.754456 2.053367 0.165762
C -3.896820 -0.950035 -0.903351
H -4.463897 -1.751910 -0.423409
H -4.139611 -0.965036 -1.968142
C -2.367669 -1.122966 -0.673405
H -2.150435 -2.075720 -0.182637
H -1.801448 -1.091717 -1.606182
C -2.651819 -0.018007 1.430523
H -2.549556 -0.999801 1.903471
H -2.224944 0.726444 2.107729
C -4.141046 0.304271 1.108161
H -4.811302 -0.438865 1.546829
H -4.426836 1.282172 1.503832
N -1.862697 -0.033194 0.187292
N -4.351549 0.324251 -0.340376

TS2 with methyl groups (Figure 4) C14H25N3S2
-1508.92530243 -1508.543724 -1508.522426 -1508.592703
C 3.421447 0.697591 0.561072
C 1.781405 1.174256 2.388632

C 1.238607 -1.448094 1.720468
C 0.282494 4.020931 -1.103773
C 1.929001 0.808866 0.899083
C 1.312115 1.915402 0.148292
H 0.688615 -1.023777 2.559854
H 3.924753 1.609327 0.886604
H 3.860545 -0.160146 1.075720
H 3.570051 0.574454 -0.510898
N 1.200432 -0.499536 0.588197
H 2.204136 2.169291 2.526346
H 0.732695 1.213433 2.691253
H 2.322332 0.478869 3.031126
H 2.258117 -1.694162 2.020397
H 0.764052 -2.375164 1.404735
H -0.133850 -0.258709 0.354574
C 0.853408 2.904257 -0.358084
H 1.025142 4.807879 -1.246750
H -0.043539 3.680729 -2.090099
H -0.573398 4.455500 -0.582917
C 1.642910 -1.112509 -0.718750
S 2.561825 -2.499612 -0.664442
S 1.124968 -0.292598 -2.090978
C -3.357419 0.708900 -0.844077
C -1.857414 0.938646 -0.536248
H -3.503307 0.423837 -1.888192
H -3.932029 1.619848 -0.664842
H -1.231820 1.042141 -1.423070
H -1.693907 1.809267 0.101662
C -3.289590 -1.637175 -0.423152
H -3.578339 -2.404469 0.299109
H -3.700048 -1.923530 -1.392760
C -1.750131 -1.491289 -0.510114
H -1.215530 -2.329636 -0.057505
H -1.387055 -1.383455 -1.531870
C -1.986377 -0.256159 1.563386
H -1.721352 -1.201477 2.041763
H -1.552217 0.560937 2.144124
C -3.521033 -0.108190 1.387552
H -4.050766 -0.806669 2.037967
H -3.848425 0.901333 1.645557
N -1.359623 -0.247332 0.216132
N -3.893677 -0.367829 -0.003028

State 5 with methyl groups (Figure 4) C14H25N3S2
-1508.94207581 -1508.557435 -1508.534904 -1508.610093
C 3.918922 0.511910 -1.282112
C 4.097403 0.799094 1.196163
C 3.192004 -1.806242 1.100584
C 0.475637 3.630968 0.371037
C 3.128796 0.478322 0.034387
C 2.118480 1.555881 0.100314
H 2.983934 -1.635710 2.160364
H 4.428401 1.472959 -1.381750
H 4.668328 -0.284021 -1.263828
H 3.256930 0.362973 -2.131519
N 2.487863 -0.857389 0.239468

H 4.410643 1.837058 1.086461
H 3.601644 0.692612 2.161572
H 4.989364 0.173465 1.171885
H 4.264042 -1.730107 0.922365
H 2.866079 -2.810749 0.851073
H -1.086179 -0.661044 -0.904242
C 1.409084 2.513041 0.262224
H -0.316534 3.545195 -0.378255
H 0.015658 3.666492 1.361479
H 0.985776 4.582232 0.207712
C 1.300854 -1.219176 -0.327391
S 0.421328 -2.531996 0.333573
S 0.715063 -0.370253 -1.715312
C -3.984483 1.046876 -0.681267
C -2.607233 0.679832 -1.287455
H -4.797393 0.630327 -1.279572
H -4.113849 2.129932 -0.651849
H -2.672214 0.270596 -2.296465
H -1.903184 1.513237 -1.304608
C -4.135422 -0.951395 0.612774
H -4.085628 -1.335450 1.633421
H -5.089792 -1.263615 0.185630
C -2.953948 -1.494233 -0.234166
H -2.400985 -2.303422 0.240316
H -3.261918 -1.818752 -1.229349
C -1.647983 0.224568 0.907873
H -1.311442 -0.602402 1.532550
H -0.798377 0.891518 0.742906
C -2.917291 0.928468 1.445612
H -3.072958 0.681556 2.496816
H -2.830751 2.014826 1.368461
N -2.001693 -0.366265 -0.417730
N -4.097711 0.513485 0.677519

TS3 with methyl groups (Figure 4) C14H25N3S2
-1508.92023362 -1508.535388 -1508.514197 -1508.583617
C -2.233879 -1.028611 -2.151738
C -3.834588 -0.869133 -0.220004
C -2.442865 1.752170 -1.501519
C -0.047268 -3.247114 1.153251
C -2.389626 -0.635990 -0.680473
C -1.433931 -1.450389 0.147631
H -1.759869 1.742258 -2.356935
H -2.356528 -2.107635 -2.229060
H -2.979399 -0.535305 -2.778662
H -1.235176 -0.779751 -2.515726
N -2.032562 0.794073 -0.485883
H -4.093085 -1.918794 -0.370753
H -3.937564 -0.627975 0.838302
H -4.526663 -0.250273 -0.797439
H -2.448692 2.747594 -1.066562
H -3.445291 1.499842 -1.851894
H 0.561153 -0.715790 0.332479
C -0.818871 -2.567348 0.077249
H -0.698543 -3.973272 1.648159
H 0.810587 -3.813780 0.780877

H 0.293534 -2.545479 1.938299
C -1.450333 1.175601 0.670228
S -0.889147 2.743103 0.961133
S -1.259122 -0.114535 1.825618
C 2.700717 1.182373 -1.410154
C 1.331265 0.511017 -1.147917
H 2.669976 2.232606 -1.114056
H 2.950771 1.137988 -2.471225
H 0.545258 1.234934 -0.932748
H 1.013751 -0.149539 -1.956229
C 3.535430 0.782576 0.788249
H 4.252928 0.186953 1.356453
H 3.741325 1.834497 0.989106
C 2.079895 0.438446 1.192488
H 2.009466 -0.175875 2.089928
H 1.439766 1.313782 1.322771
C 2.385549 -1.512553 -0.264806
H 2.568418 -2.043241 0.669968
H 1.827120 -2.169295 -0.934620
C 3.680750 -0.924852 -0.878944
H 4.556788 -1.406193 -0.442058
H 3.710886 -1.092007 -1.957245
N 1.497676 -0.357334 0.063874
N 3.753161 0.517477 -0.638045

State 6 with methyl groups (Figure 4) C14H25N3S2
-1508.92177499 -1508.537318 -1508.516420 -1508.585647
C -1.934743 1.086252 2.181841
C -3.757983 0.945292 0.463621
C -2.332486 -1.730560 1.586031
C -0.007736 3.105788 -1.309741
C -2.276777 0.673532 0.754355
C -1.389133 1.393572 -0.247013
H -1.555187 -1.766581 2.356649
H -2.003035 2.171140 2.244110
H -2.617982 0.632532 2.903639
H -0.908425 0.810872 2.434300
N -1.994775 -0.774848 0.544526
H -3.956391 2.008167 0.612507
H -3.999152 0.684291 -0.568074
H -4.402575 0.370413 1.134451
H -2.438453 -2.717760 1.143036
H -3.272773 -1.433814 2.053461
H 0.510825 0.820859 -0.035283
C -0.727910 2.526094 -0.123388
H -0.676249 3.823706 -1.796980
H 0.879701 3.680204 -1.026839
H 0.275173 2.372027 -2.088506
C -1.533358 -1.150912 -0.658322
S -1.071370 -2.708191 -1.084349
S -1.387070 0.212551 -1.754929
C 2.750459 -1.389056 1.181154
C 1.381740 -0.667247 1.152391
H 2.670562 -2.372390 0.713825
H 3.084603 -1.530400 2.210077
H 0.559051 -1.338013 0.902774

H 1.160831 -0.145319 2.084609
C 3.418497 -0.632757 -0.978014
H 4.085347 0.061964 -1.492855
H 3.606343 -1.631632 -1.373704
C 1.933776 -0.247442 -1.195167
H 1.785108 0.482198 -1.990228
H 1.289395 -1.107424 -1.387752
C 2.402668 1.461204 0.498787
H 2.528922 2.111134 -0.367242
H 1.909795 2.034474 1.285738
C 3.722005 0.774217 0.927571
H 4.578024 1.313720 0.519719
H 3.823125 0.761966 2.014629
N 1.459723 0.377967 0.082541
N 3.752813 -0.610826 0.449755

State 7 with methyl groups (Figure 4) C14H25N3S2
-1509.00423766 -1508.619997 -1508.597746 -1508.674007
C -0.594521 -0.977023 -1.262747
C -0.607596 -0.973860 1.263852
C -2.986501 -2.460636 -0.011779
C -0.519829 3.338864 -0.011031
C -1.335783 -0.514930 -0.003981
C -1.465136 1.005973 -0.005426
H -3.572097 -2.730970 -0.892513
H 0.407489 -0.544222 -1.253309
H -0.497975 -2.065596 -1.287833
H -1.124819 -0.645224 -2.157219
N -2.720246 -1.030452 -0.009439
H 0.396878 -0.546436 1.260683
H -1.143008 -0.633496 2.152111
H -0.519431 -2.063027 1.296170
H -3.561871 -2.736235 0.874198
H -2.042253 -2.999983 -0.019103
H 0.564153 1.398099 -0.021061
C -0.430206 1.843137 -0.013660
H -1.553189 3.689363 -0.018744
H -0.031036 3.755756 0.874180
H -0.016126 3.759575 -0.885823
C -3.741562 -0.165613 0.000989
S -5.353881 -0.535672 0.005844
S -3.162356 1.503004 0.010446
C 4.431303 -1.667710 0.043134
C 2.891265 -1.465981 -0.080394
H 4.819830 -2.283721 -0.771424
H 4.693237 -2.158899 0.983796
H 2.514381 -1.844680 -1.035046
H 2.351347 -1.984857 0.717141
C 4.702689 0.348369 -1.203450
H 5.288272 1.267950 -1.275895
H 4.957876 -0.275406 -2.063862
C 3.174585 0.648950 -1.155421
H 2.981118 1.720433 -1.047431
H 2.668791 0.312123 -2.065039
C 3.144376 0.515985 1.229788
H 2.811765 1.553036 1.333344

H 2.733252 -0.047996 2.072432
C 4.697848 0.412476 1.180482
H 5.158563 1.400698 1.103485
H 5.095094 -0.069234 2.077092
N 2.570023 -0.034926 -0.004613
N 5.109361 -0.367817 0.009324

State 8 with methyl groups (Figure 4) C8H13NS2
-1163.72614303 -1163.527686 -1163.513886 -1163.567196
C 0.579808 1.822175 1.409822
C 0.813692 2.108247 -1.094764
C -2.072315 1.654448 -0.134597
C 3.431579 -1.396396 -0.098564
C 0.424962 1.154535 0.037188
C 1.269165 -0.114936 -0.018370
H -2.751316 1.423606 -0.955645
H 1.615438 2.137160 1.549590
H -0.063410 2.702097 1.488636
H 0.318605 1.119054 2.203174
N -0.972100 0.701126 -0.130149
H 1.868571 2.371170 -1.009073
H 0.651884 1.632014 -2.062704
H 0.240311 3.035995 -1.050239
H -1.677638 2.660160 -0.258491
H -2.636104 1.589736 0.799550
H 3.130883 0.795779 -0.123816
C 2.597860 -0.150984 -0.089513
H 4.227085 -1.323257 -0.842961
H 3.908422 -1.554526 0.873535
H 2.834344 -2.280966 -0.327268
C -1.239477 -0.610116 -0.043501
S -2.730008 -1.322208 -0.035742
S 0.249132 -1.553901 0.087026

TS3* with methyl groups (Figure 4) C14H25N3S2
-1508.90968146 -1508.525321 -1508.504620 -1508.573394
C -3.889413 0.724028 0.575007
C -2.692709 1.484857 -1.502755
C -2.744268 -1.353634 -1.691968
C -0.345478 3.585850 0.057864
C -2.570466 0.680038 -0.203132
C -1.430182 1.293992 0.572095
H -2.207726 -1.043307 -2.593796
H -4.164382 1.761392 0.777055
H -4.687794 0.259083 -0.010042
H -3.787188 0.195159 1.521869
N -2.198256 -0.723934 -0.500123
H -2.851307 2.533536 -1.246710
H -1.775881 1.411072 -2.091853
H -3.536001 1.152531 -2.111096
H -3.797674 -1.083563 -1.795035
H -2.655321 -2.431378 -1.587190
H 0.800868 0.772380 0.842670
C -0.629488 2.255089 0.661046
H 0.479942 4.116628 0.540425
H -0.099836 3.489838 -1.008059

H -1.224356 4.236587 0.119661
C -1.414842 -1.395205 0.378542
S -0.744532 -2.916688 0.049442
S -1.098242 -0.506573 1.827659
C 3.363896 -1.359075 0.252853
C 2.196990 -0.807819 1.108397
H 3.051400 -2.269733 -0.261760
H 4.220192 -1.604484 0.882765
H 1.372189 -1.513978 1.206335
H 2.508532 -0.482407 2.102096
C 2.697575 -0.224471 -1.736907
H 2.967511 0.588695 -2.414202
H 2.617078 -1.139756 -2.324576
C 1.354744 0.063404 -1.021178
H 0.808324 0.911473 -1.435748
H 0.700334 -0.810067 -0.994038
C 2.694664 1.496645 0.438658
H 2.303682 2.311138 -0.173062
H 2.765293 1.844825 1.469415
C 4.020246 0.907355 -0.107901
H 4.471328 1.593685 -0.826372
H 4.737847 0.750336 0.699577
N 1.677958 0.405982 0.401221
N 3.777402 -0.379740 -0.759053

State 6* with methyl groups (Figure 4) C14H25N3S2
-1508.99853774 -1508.613792 -1508.592703 -1508.664546
C -2.134188 1.500942 1.719087
C -3.382922 2.039395 -0.444005
C -4.312920 -0.517556 0.925506
C -0.329327 3.374300 -0.625813
C -2.426135 1.079305 0.272443
C -1.119750 0.939003 -0.505787
H -5.019500 -0.928206 0.201956
H -1.669816 2.486525 1.748279
H -3.052501 1.541106 2.309561
H -1.450731 0.784029 2.178861
N -3.028847 -0.273091 0.285165
H -2.945677 3.032724 -0.527627
H -3.589461 1.668645 -1.449364
H -4.326059 2.133512 0.098173
H -4.701562 0.416157 1.325286
H -4.196024 -1.243924 1.732209
H 0.606555 1.557837 -1.429695
C -0.268565 1.894615 -0.880889
H 0.644804 3.724971 -0.273330
H -0.542098 3.915533 -1.553279
H -1.071418 3.670277 0.112285
C -2.340662 -1.298133 -0.234613
S -2.808055 -2.886775 -0.265852
S -0.795479 -0.763739 -0.885929
C 4.062739 -1.673266 0.031843
C 2.688127 -1.436213 -0.659467
H 4.854451 -1.842028 -0.703020
H 4.033343 -2.543510 0.692012
H 2.730715 -1.654519 -1.729762

H 1.905978 -2.064468 -0.225927
C 4.626921 0.640939 -0.076828
H 4.827974 1.525401 0.533825
H 5.513674 0.451501 -0.687057
C 3.359097 0.837041 -0.961031
H 3.007301 1.873415 -0.929434
H 3.559118 0.585659 -2.006432
C 2.063831 0.218766 0.944338
H 1.835994 1.282773 1.063440
H 1.182225 -0.345939 1.261129
C 3.333993 -0.186333 1.748936
H 3.657396 0.615470 2.417781
H 3.149368 -1.073772 2.359975
N 2.280498 -0.036075 -0.485747
N 4.432785 -0.496232 0.827202

State 7* with methyl groups (Figure 4) C8H13NS2
-1163.72104625 -1163.522625 -1163.508667 -1163.562700
C -1.133213 -1.440042 1.337205
C -1.244833 -1.509960 -1.213207
C 1.531455 -2.040283 -0.038397
C -3.658905 0.519853 -0.043154
C -0.694178 -0.785119 0.020874
C -1.100332 0.688165 -0.001129
H 2.194023 -2.076448 -0.904499
H -2.217780 -1.421322 1.437543
H -0.807085 -2.481737 1.380939
H -0.699393 -0.899648 2.180511
N 0.784827 -0.790513 -0.039732
H -2.331885 -1.455616 -1.240338
H -0.854034 -1.044115 -2.119296
H -0.962993 -2.564994 -1.208587
H 0.837825 -2.876782 -0.073626
H 2.146563 -2.109446 0.861452
H -2.386483 2.298780 -0.052262
C -2.326117 1.213292 -0.030266
H -4.313570 0.963592 0.711033
H -4.149998 0.658858 -1.011455
H -3.603961 -0.547844 0.153847
C 1.448498 0.373370 -0.017827
S 3.088752 0.578028 -0.024972
S 0.331294 1.732887 0.029864

State 9 with methyl groups (Figure S2) C6H12N2
-345.267757212 -345.082973 -345.075747 -345.113494
C 0.780001 -0.556629 1.257899
C -0.776429 -0.488102 1.288251
H 1.133499 -1.589982 1.308715
H 1.223276 -0.014165 2.096998
H -1.216838 -1.465320 1.502918
H -1.130836 0.209286 2.052019
C 0.802573 -0.782242 -1.115868
H 1.134336 -0.301218 -2.040040
H 1.281629 -1.763410 -1.063630
C -0.750387 -0.900027 -1.061772
H -1.208268 -0.613618 -2.012159

H -1.064812 -1.923152 -0.838480
C -0.804323 1.340053 -0.243415
H -1.138360 1.645976 -1.238471
H -1.282120 2.002314 0.483246
C 0.748936 1.387332 -0.125140
H 1.204520 1.852155 -1.003251
H 1.064316 1.958777 0.752096
N -1.282395 -0.027269 -0.009260
N 1.282028 0.027275 0.009304

State 10 with methyl groups (Figure S2) C7H12N2S2
-1179.73827661 -1179.545910 -1179.533867 -1179.586795
C -2.551521 -1.115541 -0.642607
C -1.040167 -1.057103 -1.020877
H -2.795697 -2.044716 -0.120937
H -3.189045 -1.056335 -1.528422
H -0.534368 -2.004207 -0.812097
H -0.901871 -0.835538 -2.082728
C -2.128245 -0.142913 1.493786
H -2.350746 0.722147 2.124129
H -2.486427 -1.033783 2.015966
C -0.605663 -0.240971 1.177679
H -0.027019 0.493075 1.746927
H -0.211054 -1.232708 1.417094
C -0.957693 1.294041 -0.613578
H -0.484777 2.058368 0.010123
H -0.698867 1.510551 -1.653749
C -2.501633 1.258503 -0.400251
H -2.838530 2.087825 0.226836
H -3.036677 1.324229 -1.351324
N -0.376774 -0.001618 -0.250709
N -2.887957 0.000887 0.247609
C 2.643735 0.001031 -0.002411
S 2.660776 1.554118 0.002249
S 2.667681 -1.551998 0.002716

TS4 with methyl groups (Figure S2) C7H12N2S2
-1179.72501398 -1179.530962 -1179.520537 -1179.567239
C -2.155152 -0.956246 -0.968296
C -0.622283 -0.776939 -1.153548
H -2.390178 -1.970385 -0.638006
H -2.677075 -0.783413 -1.911262
H -0.089014 -1.727211 -1.143217
H -0.375285 -0.252060 -2.079023
C -2.104548 -0.393345 1.339455
H -2.386406 0.376116 2.061571
H -2.553228 -1.334475 1.662739
C -0.558002 -0.530533 1.248562
H -0.042314 0.002810 2.049779
H -0.231738 -1.570678 1.266369
C -0.644681 1.414402 -0.166321
H -0.338901 1.973535 0.718199
H -0.177277 1.886028 -1.030915
C -2.192137 1.322355 -0.300051
H -2.674442 2.044719 0.361021
H -2.506136 1.544482 -1.322281

N -0.104758 0.045671 -0.038805
N -2.661572 -0.021477 0.037884
C 1.816226 -0.003466 -0.018555
S 2.388433 1.502077 0.004292
S 2.272177 -1.553090 0.003458

State 11 with methyl groups (Figure S2) C7H12N2S2

-1179.72646095 -1179.530855 -1179.520270 -1179.566879
C -2.096710 -0.968335 -0.954915
C -0.578732 -0.760386 -1.177241
H -2.296971 -1.980982 -0.598497
H -2.634566 -0.832945 -1.894681
H -0.025315 -1.695830 -1.201282
H -0.356447 -0.195166 -2.084176
C -2.053571 -0.372436 1.344037
H -2.318646 0.418601 2.048388
H -2.511155 -1.298951 1.694918
C -0.516245 -0.544740 1.256022
H 0.018725 -0.032310 2.056640
H -0.206277 -1.587900 1.247330
C -0.606580 1.428965 -0.149548
H -0.322469 1.966494 0.753359
H -0.122894 1.920890 -0.990431
C -2.143201 1.312928 -0.318735
H -2.642153 2.048616 0.314201
H -2.434304 1.512385 -1.352162
N -0.035595 0.049505 -0.037375
N -2.611718 -0.024849 0.037226
C 1.614385 0.002289 -0.025450
S 2.339118 1.476274 0.005353
S 2.202607 -1.539100 0.004797

State 12 with methyl groups (Figure S2) C14H25N3S2

-1508.93188645 -1508.546996 -1508.524251 -1508.599161
C 2.870533 -0.011655 -2.283092
C 4.255962 -0.854542 -0.357395
C 1.791193 -2.487547 -0.808894
C 2.237955 3.237213 1.340583
C 2.898478 -0.253967 -0.767460
C 2.717182 1.030165 -0.066755
H 2.656669 -3.045313 -0.430203
H 3.656065 0.691258 -2.564487
H 3.036531 -0.943707 -2.827259
H 1.903767 0.403789 -2.575087
N 1.740756 -1.091075 -0.385387
H 5.057220 -0.141278 -0.558784
H 4.259277 -1.085239 0.710204
H 4.461718 -1.768313 -0.919119
H 1.792970 -2.549431 -1.899922
H 0.888130 -2.981264 -0.444777
H 1.629981 -1.047335 0.625320
C 2.525226 2.041272 0.554741
H 2.210575 4.127319 0.709368
H 1.269038 3.122226 1.833457
H 2.996434 3.385975 2.111014
C -2.528585 -0.457535 -2.088362

C -1.552108 -0.994287 -1.015987
H -3.460287 -1.027758 -2.087894
H -2.079698 -0.555430 -3.078487
H -1.825771 -1.984330 -0.663837
H -0.510621 -1.007450 -1.340777
C -3.665449 1.025625 -0.628831
H -3.760347 2.076750 -0.348633
H -4.666064 0.644575 -0.840734
C -3.023634 0.207432 0.519171
H -3.023214 0.741039 1.469547
H -3.491865 -0.767051 0.660970
C -0.954800 1.229076 -0.267362
H -1.132871 1.951028 0.525448
H 0.117166 1.051723 -0.339122
C -1.596646 1.660596 -1.607424
H -1.788846 2.734871 -1.596062
H -0.926100 1.450977 -2.443590
N -1.588047 -0.064827 0.168443
N -2.853069 0.947712 -1.842531
C -0.897202 -0.684584 1.491589
S -0.204008 0.432934 2.491932
S -1.098241 -2.316927 1.673233

TS5 with methyl groups (Figure S2) C14H25N3S2
-1508.92833593 -1508.545517 -1508.522927 -1508.597507
C -3.168655 -0.387769 1.756548
C -4.226647 -0.755892 -0.496498
C -1.843241 -2.581919 0.348038
C -2.178916 3.582052 -0.831033
C -2.935272 -0.347312 0.239116
C -2.603631 1.040302 -0.147962
H -2.797311 -3.084806 0.139074
H -3.927261 0.345689 2.032007
H -3.516214 -1.375891 2.064559
H -2.244310 -0.155991 2.289399
N -1.779138 -1.194509 -0.102367
H -5.043996 -0.080095 -0.237106
H -4.074897 -0.713845 -1.577050
H -4.519560 -1.770471 -0.219061
H -1.649598 -2.634902 1.421409
H -1.050113 -3.137659 -0.152576
H -1.624677 -1.165086 -1.106216
C -2.397688 2.184787 -0.458813
H -1.763301 3.656981 -1.837942
H -3.122034 4.131293 -0.814028
H -1.489766 4.070402 -0.138005
C 0.930476 1.962990 1.396448
C 0.907164 0.419537 1.205023
H 1.211414 2.225658 2.418687
H -0.058775 2.383379 1.198114
H 1.409776 -0.097967 2.023332
H -0.104596 0.023034 1.102783
C 3.173881 1.896473 0.607354
H 3.924886 2.433226 0.023752
H 3.471571 1.943100 1.657471
C 3.055585 0.418440 0.128338

H 3.543402 0.260718 -0.837065
H 3.481165 -0.282668 0.849397
C 1.072885 0.876842 -1.144384
H 1.502176 0.513258 -2.079241
H -0.002925 0.693617 -1.162589
C 1.398760 2.378459 -0.896188
H 2.168910 2.732861 -1.586003
H 0.507470 2.989548 -1.048320
N 1.631108 0.089878 -0.036891
N 1.885663 2.579877 0.469977
C 1.499069 -1.962409 -0.386963
S 1.144266 -2.142443 -1.935947
S 1.824641 -2.643656 1.020074

State 13 with methyl groups (Figure S2) C14H25N3S2
-1508.93731538 -1508.555818 -1508.531749 -1508.612347
C -2.360707 -1.691134 1.505767
C -3.768766 -2.124667 -0.534061
C -0.929458 -2.840802 -0.997914
C -3.434057 2.735887 -0.260297
C -2.597050 -1.304826 0.038585
C -2.936203 0.128083 -0.038230
H -1.624719 -3.400089 -1.637586
H -3.261202 -1.499077 2.091611
H -2.118747 -2.753057 1.589781
H -1.536576 -1.107595 1.917850
N -1.335881 -1.468299 -0.716249
H -4.690345 -1.884083 -0.001401
H -3.913144 -1.897483 -1.592971
H -3.578567 -3.194689 -0.423806
H -0.802903 -3.390066 -0.063598
H 0.045651 -2.815309 -1.489517
H -1.423918 -0.956177 -1.588603
C -3.192195 1.299264 -0.140765
H -3.863626 3.131770 0.662014
H -2.493075 3.261827 -0.451488
H -4.124572 2.948252 -1.078217
C -0.044547 2.392139 1.111700
C 0.740591 1.057974 1.225416
H 0.209537 3.078836 1.924481
H -1.120071 2.211513 1.149188
H 1.153789 0.911870 2.228058
H 0.090899 0.209675 0.992197
C 1.697602 3.493754 -0.071052
H 1.989065 3.875979 -1.053377
H 1.768396 4.320454 0.640689
C 2.594437 2.293889 0.357730
H 3.489260 2.220595 -0.266343
H 2.922854 2.395909 1.395966
C 1.246793 0.969050 -1.093997
H 2.055345 1.072905 -1.824337
H 0.799359 -0.020232 -1.217305
C 0.179073 2.089123 -1.253419
H 0.292131 2.622334 -2.201405
H -0.827704 1.664878 -1.219478
N 1.837620 1.041820 0.247909

N 0.297076 3.059394 -0.153805
C 2.415870 -2.100020 0.181339
S 3.182929 -1.990759 -1.166130
S 1.648501 -2.248343 1.521618

State 14 with methyl groups (Figure S2) C14H25N3S2
-1508.93943913 -1508.557984 -1508.533744 -1508.614029
C 3.808905 1.004627 0.625046
C 2.027451 2.452929 1.650845
C 1.742676 -0.305753 2.597742
C 0.750790 2.088979 -3.010760
C 2.294295 1.240661 0.734838
C 1.761967 1.550683 -0.606181
H 1.251442 0.423057 3.259339
H 4.309089 1.896155 0.241831
H 4.225896 0.773147 1.608023
H 4.000322 0.166663 -0.045631
N 1.642236 -0.002381 1.179688
H 2.457232 3.356460 1.214632
H 0.953714 2.607283 1.779916
H 2.481163 2.296739 2.632376
H 2.789890 -0.384256 2.901208
H 1.282450 -1.281170 2.769031
H 0.661431 -0.009930 0.896514
C 1.314429 1.813055 -1.691374
H 0.691383 1.171292 -3.601470
H -0.256359 2.504552 -2.924690
H 1.367002 2.805331 -3.556991
C 1.108994 -2.459791 -0.717693
S 0.485590 -3.224650 0.485318
S 1.723901 -1.713718 -1.928406
C -2.327171 -0.800826 1.270972
C -3.795946 -0.492902 0.850722
H -2.084571 -1.857806 1.137072
H -2.147887 -0.547939 2.319890
H -4.267168 -1.364568 0.389144
H -4.405280 -0.204311 1.710936
C -1.667187 -0.286218 -0.967391
H -0.905266 0.224816 -1.561218
H -1.544911 -1.361631 -1.124354
C -3.107060 0.179282 -1.333039
H -3.088108 1.026994 -2.023698
H -3.676738 -0.622124 -1.810214
C -3.134340 1.762930 0.448361
H -3.233385 2.599914 -0.247484
H -3.650980 2.037880 1.371686
C -1.639565 1.419100 0.712640
H -0.969192 1.991309 0.065234
H -1.357860 1.626608 1.749741
N -3.822441 0.602338 -0.124247
N -1.400451 -0.008687 0.451731

TS6 with methyl groups (Figure S2) C14H25N3S2
-1508.91968822 -1508.536740 -1508.514138 -1508.589232
C 3.518194 0.976787 1.013855
C 1.471375 1.525267 2.352175

C 1.878782 -1.381787 1.972653
C 0.317747 3.283314 -1.921136
C 1.984793 0.918333 1.027881
C 1.410948 1.733479 -0.057083
H 1.531149 -1.018533 2.941908
H 3.824699 2.023848 1.021406
H 3.926002 0.500341 1.907094
H 3.924986 0.499813 0.125600
N 1.482817 -0.486451 0.879633
H 1.690686 2.593669 2.357585
H 0.390869 1.394654 2.438563
H 1.962758 1.069576 3.212912
H 2.963083 -1.493203 1.988281
H 1.431744 -2.356410 1.790512
H 0.447284 -0.430733 0.828671
C 0.915364 2.470417 -0.866458
H 0.480535 2.815808 -2.894955
H -0.758713 3.392052 -1.766337
H 0.759373 4.281062 -1.941750
C 1.694805 -1.444478 -0.830747
S 0.885523 -2.833681 -0.652815
S 2.546795 -0.596649 -1.891655
C -2.236420 -1.325946 0.935329
C -3.687960 -1.248557 0.379963
H -1.725833 -2.237238 0.616647
H -2.225694 -1.297329 2.029109
H -3.858490 -2.015267 -0.379970
H -4.426631 -1.396614 1.171569
C -1.580561 -0.113949 -1.023096
H -0.887760 0.643303 -1.395586
H -1.251858 -1.078803 -1.413702
C -3.052753 0.188094 -1.415542
H -3.163692 1.204837 -1.802661
H -3.404765 -0.499083 -2.188553
C -3.567931 1.107355 0.718522
H -3.860027 2.075467 0.303898
H -4.155565 0.944981 1.626090
C -2.042358 1.057706 1.019914
H -1.509482 1.908803 0.585907
H -1.857121 1.061993 2.097977
N -3.923132 0.059362 -0.241148
N -1.460937 -0.169756 0.449220

State 2 with methoxyphenyl (Figure 5) C23H25NOS2
-1817.61465418 -1817.183560 -1817.156182 -1817.245177
C 1.103262 3.026495 -1.114101
C 1.090046 2.490916 1.349208
C 1.798411 0.130774 -1.537232
C -3.023644 0.596676 -0.313467
C -0.531180 1.445194 -0.169744
H 0.773863 -0.111622 -1.834100
N 1.743007 0.747026 -0.214476
H 2.222679 0.795150 -2.305972
C -1.674578 1.072308 -0.247150
C 1.506460 -1.540359 1.946353
S -0.031071 -1.544881 1.734867

S 3.046794 -1.538466 2.151483
H 0.937312 2.626488 -2.117506
H 0.951183 1.684947 2.074703
C 2.502711 3.624665 -0.977152
H 3.257521 2.852291 -1.174139
H 2.648047 4.400766 -1.732878
C 2.468011 3.142392 1.503304
H 2.540629 3.590360 2.497823
H 3.259279 2.384548 1.460545
C 2.710921 4.201627 0.425532
H 2.006028 5.028734 0.573642
H 3.716360 4.619085 0.523912
H 0.350629 3.806814 -0.959068
H 0.314097 3.240725 1.531891
C 2.625405 -1.127001 -1.449045
C 4.017388 -1.045494 -1.464295
C 2.025164 -2.371839 -1.268629
C 4.798473 -2.181910 -1.281920
H 4.493127 -0.080451 -1.618534
C 2.801329 -3.512248 -1.092678
H 0.942527 -2.442351 -1.253976
C 4.189803 -3.418500 -1.091225
H 5.879449 -2.103771 -1.291580
H 2.322523 -4.474126 -0.949365
H 4.794469 -4.306173 -0.947701
C 0.862020 1.912561 -0.065228
C -3.736212 0.611567 -1.522841
C -3.657693 0.101670 0.826293
C -5.034195 0.144930 -1.583279
H -3.255710 0.995798 -2.414381
C -4.965812 -0.370983 0.776400
H -3.118406 0.090170 1.766124
C -5.657574 -0.351116 -0.433872
H -5.594405 0.150509 -2.510053
H -5.426245 -0.747292 1.679614
O -6.930880 -0.789064 -0.595737
C -7.604802 -1.293448 0.538564
H -7.086703 -2.164750 0.951261
H -7.703633 -0.526075 1.312705
H -8.592880 -1.590134 0.194467
H 2.680399 0.963965 0.105423

TS1 with methoxyphenyl (Figure 5) C23H25NOS2
-1817.59266248 -1817.160666 -1817.134639 -1817.219877
C 1.134121 2.393916 -1.469377
C 1.002989 2.586554 1.037579
C 1.315703 -0.681464 -0.956375
C -3.170224 0.526250 -0.333585
C -0.637784 1.239591 -0.224082
H 0.419913 -1.180533 -0.579116
N 1.619043 0.410624 -0.007469
H 1.079197 -0.270007 -1.940662
C -1.803426 0.949730 -0.303638
C 1.687274 -0.378510 1.784560
S 0.258504 -1.023582 2.133751
S 3.198260 -0.160081 2.298281

H 0.994864 1.739477 -2.332038
H 0.798258 2.048049 1.964518
C 2.538772 2.997623 -1.444769
H 3.299998 2.208164 -1.392679
H 2.714374 3.524056 -2.386266
C 2.398164 3.217154 1.053550
H 2.457742 3.908341 1.897856
H 3.156993 2.449980 1.235489
C 2.702428 3.944855 -0.255788
H 2.011329 4.787864 -0.375162
H 3.713192 4.359082 -0.234189
H 0.399133 3.198518 -1.571842
H 0.250268 3.376692 0.953093
C 2.478569 -1.634802 -1.055177
C 3.567381 -1.317955 -1.868455
C 2.499468 -2.818834 -0.321502
C 4.665693 -2.166567 -1.942279
H 3.550246 -0.402406 -2.454212
C 3.595762 -3.671139 -0.396127
H 1.659582 -3.063258 0.319986
C 4.680349 -3.345782 -1.203542
H 5.505810 -1.911362 -2.577290
H 3.604707 -4.587061 0.182503
H 5.535136 -4.009373 -1.258181
C 0.772289 1.639868 -0.162128
C -4.089597 1.078631 -1.237997
C -3.605288 -0.466823 0.544514
C -5.400847 0.646859 -1.258384
H -3.760922 1.849945 -1.923946
C -4.923121 -0.910839 0.528434
H -2.897538 -0.894313 1.245045
C -5.826008 -0.352179 -0.376524
H -6.122409 1.063248 -1.950332
H -5.228494 -1.683988 1.219973
O -7.129038 -0.710564 -0.475390
C -7.603378 -1.723395 0.388780
H -7.069488 -2.664356 0.224016
H -7.505694 -1.425340 1.437199
H -8.655199 -1.856583 0.146989
H 2.604045 0.667465 -0.073969

State 3 with methoxyphenyl (Figure 5) C23H25NOS2
-1817.59812376 -1817.163972 -1817.138033 -1817.221928
C 1.148719 2.217801 -1.564418
C 0.963361 2.667518 0.910751
C 1.264911 -0.812075 -0.795546
C -3.173730 0.463128 -0.368762
C -0.647879 1.190710 -0.252104
H 0.386491 -1.249656 -0.324442
N 1.636675 0.374042 0.061182
H 0.989129 -0.437510 -1.779445
C -1.809670 0.892741 -0.350786
C 1.789956 -0.077345 1.565263
S 0.423653 -0.674220 2.275983
S 3.353883 0.091522 2.081008
H 1.042530 1.476109 -2.358048

H 0.773284 2.213266 1.883884
C 2.534438 2.864350 -1.578531
H 3.324994 2.117923 -1.422212
H 2.711484 3.283180 -2.572327
C 2.331482 3.355049 0.870102
H 2.343378 4.136011 1.634130
H 3.115801 2.643869 1.143165
C 2.633158 3.946844 -0.505269
H 1.913547 4.742207 -0.734370
H 3.626758 4.400519 -0.512854
H 0.397034 2.986407 -1.767375
H 0.178347 3.412873 0.748584
C 2.413087 -1.778255 -0.892295
C 3.383783 -1.603561 -1.878794
C 2.533433 -2.834306 0.009485
C 4.467946 -2.469508 -1.961442
H 3.287093 -0.788248 -2.591110
C 3.615749 -3.701774 -0.074203
H 1.786490 -2.960640 0.786244
C 4.583682 -3.519992 -1.056902
H 5.217783 -2.327608 -2.730554
H 3.707331 -4.515957 0.634351
H 5.428122 -4.196165 -1.117624
C 0.751339 1.610580 -0.195372
C -4.035837 0.796487 -1.423922
C -3.661074 -0.316969 0.680561
C -5.345625 0.360801 -1.423323
H -3.665949 1.401834 -2.242610
C -4.977971 -0.763084 0.687209
H -2.994585 -0.576287 1.494858
C -5.825035 -0.422930 -0.368252
H -6.024802 0.610176 -2.228892
H -5.325425 -1.368055 1.513301
O -7.121566 -0.802949 -0.459646
C -7.656380 -1.589511 0.586260
H -7.126064 -2.542648 0.673877
H -7.613507 -1.056712 1.541065
H -8.694397 -1.776161 0.321278
H 2.599293 0.639543 -0.174107

State 2 with phenyl (Figure 5) C22H23NS2
-1703.10167800 -1702.704080 -1702.679178 -1702.762321
C -0.022262 2.991330 -1.246723
C 0.137816 2.514380 1.222385
C 1.112850 0.238311 -1.657617
C -3.622036 -0.145625 -0.240925
C -1.340764 1.177960 -0.211198
H 0.105323 -0.045394 -1.975840
N 1.001860 0.859872 -0.341096
H 1.526327 0.910938 -2.424224
C -2.392528 0.590457 -0.231673
C 1.465868 -1.153184 2.134689
S -0.060330 -1.393526 2.291622
S 2.992838 -0.914820 1.979622
H -0.153741 2.546988 -2.236819
H 0.151878 1.714261 1.966254

C 1.266871 3.809208 -1.171720
H 2.127479 3.164541 -1.391102
H 1.255754 4.584238 -1.942387
C 1.399223 3.377814 1.314912
H 1.433055 3.852357 2.299133
H 2.298063 2.752320 1.256623
C 1.435369 4.439157 0.213240
H 0.618426 5.152321 0.377624
H 2.367778 5.007318 0.264005
H -0.884575 3.645027 -1.076634
H -0.740847 3.135876 1.421570
C 1.975701 -0.992111 -1.532984
C 3.340751 -0.942013 -1.801720
C 1.423350 -2.179175 -1.051100
C 4.148209 -2.055680 -1.579857
H 3.776190 -0.023213 -2.183713
C 2.222111 -3.293823 -0.833700
H 0.360993 -2.211030 -0.829708
C 3.590077 -3.231481 -1.092095
H 5.210525 -2.004270 -1.788379
H 1.783247 -4.209659 -0.454484
H 4.216148 -4.098225 -0.915801
C -0.043562 1.875687 -0.172476
C -4.846874 0.504548 -0.430435
C -3.605226 -1.533728 -0.056145
C -6.030197 -0.222191 -0.434123
H -4.857907 1.578124 -0.573576
C -4.791827 -2.254509 -0.062338
H -2.654601 -2.032824 0.090759
C -6.006648 -1.601731 -0.250371
H -6.974026 0.289217 -0.581316
H -4.768974 -3.328253 0.080992
H 1.904093 1.218332 -0.049404
H -6.931405 -2.166177 -0.253969

TS1 with phenyl (Figure 5) C22H23NS2
-1703.07886766 -1702.680059 -1702.656524 -1702.735738
C 0.177277 2.245484 -1.498440
C -0.043744 2.470863 0.999794
C 0.849312 -0.741609 -0.911986
C -3.831081 -0.200818 -0.365550
C -1.418682 0.852558 -0.257265
H 0.086922 -1.414921 -0.512894
N 0.937314 0.400409 0.024659
H 0.511043 -0.398546 -1.892167
C -2.529660 0.398418 -0.346048
C 1.076999 -0.329584 1.824545
S -0.225548 -1.215240 2.144790
S 2.508620 0.154462 2.388265
H 0.166124 1.558440 -2.346763
H -0.180818 1.926397 1.935797
C 1.468478 3.063537 -1.461678
H 2.342501 2.404189 -1.379740
H 1.578763 3.591871 -2.412043
C 1.234378 3.313886 1.027674
H 1.161357 4.026366 1.853083

H 2.098750 2.680878 1.250149
C 1.456725 4.049229 -0.293525
H 0.650471 4.776190 -0.449078
H 2.392383 4.612385 -0.261086
H -0.673398 2.920307 -1.636681
H -0.909887 3.129700 0.881695
C 2.187764 -1.422422 -1.030086
C 3.145763 -0.906823 -1.904357
C 2.504102 -2.534263 -0.251938
C 4.404842 -1.487541 -1.997089
H 2.898109 -0.047184 -2.521972
C 3.762931 -3.118237 -0.344753
H 1.765966 -2.931559 0.437135
C 4.714455 -2.595770 -1.214435
H 5.140356 -1.080919 -2.681120
H 4.002610 -3.979980 0.266724
H 5.694893 -3.051761 -1.284748
C -0.091374 1.472361 -0.179497
C -4.223117 -1.016972 0.702449
C -4.712124 0.013259 -1.430850
C -5.478542 -1.608719 0.700303
H -3.531610 -1.175977 1.521215
C -5.967461 -0.580251 -1.424355
H -4.404533 0.644691 -2.255572
C -6.352744 -1.391915 -0.361215
H -5.775987 -2.239390 1.529485
H -6.647019 -0.409929 -2.250806
H 1.868384 0.814269 -0.022266
H -7.332723 -1.853966 -0.360020

State 3 with phenyl (Figure 5) C22H23NS2
-1703.08405137 -1702.682864 -1702.659478 -1702.737499
C 0.196725 2.096044 -1.599243
C -0.072806 2.576052 0.862333
C 0.777391 -0.854646 -0.760867
C -3.825180 -0.249971 -0.318765
C -1.427947 0.836338 -0.257470
H 0.004285 -1.433891 -0.258821
N 0.954630 0.395501 0.068345
H 0.413723 -0.554364 -1.741721
C -2.535099 0.372240 -0.337125
C 1.186018 0.006770 1.580422
S -0.062343 -0.783480 2.317172
S 2.707428 0.433346 2.074691
H 0.212421 1.327353 -2.374106
H -0.198372 2.123422 1.846831
C 1.469435 2.943669 -1.624121
H 2.362224 2.331218 -1.437590
H 1.593825 3.355072 -2.628788
C 1.172215 3.466934 0.807355
H 1.053718 4.260766 1.548584
H 2.055027 2.895317 1.105710
C 1.391722 4.059714 -0.583120
H 0.560806 4.727796 -0.839723
H 2.304088 4.659967 -0.598718
H -0.660661 2.736740 -1.825552

H -0.963090 3.184915 0.675164
C 2.082900 -1.590292 -0.875925
C 3.001103 -1.204250 -1.852481
C 2.405839 -2.622120 0.002497
C 4.234982 -1.835508 -1.947670
H 2.746222 -0.404917 -2.543922
C 3.637765 -3.258878 -0.096669
H 1.698350 -2.905979 0.774737
C 4.552643 -2.865432 -1.067833
H 4.943838 -1.528641 -2.707329
H 3.886720 -4.057419 0.591567
H 5.513774 -3.360591 -1.139589
C -0.109915 1.468924 -0.215877
C -4.158902 -1.080339 0.758186
C -4.746790 -0.047522 -1.351130
C -5.400525 -1.699308 0.795648
H -3.433116 -1.228161 1.549287
C -5.988030 -0.668242 -1.303772
H -4.482117 0.595144 -2.182058
C -6.316305 -1.494761 -0.232917
H -5.655373 -2.341267 1.630262
H -6.700793 -0.508096 -2.103835
H 1.862596 0.805159 -0.177878
H -7.285294 -1.978404 -0.199486

State 2 with trifluoromethylphenyl (Figure 5) C23H22F3NS2
-2040.15430221 -2039.751457 -2039.723017 -2039.815330
C 1.689129 2.973056 -1.295482
C 1.796992 2.551395 1.190867
C 2.273319 0.050662 -1.625974
C -2.456379 0.717846 -0.151763
C 0.062584 1.493918 -0.183002
H 1.225646 -0.083675 -1.911671
N 2.295567 0.713000 -0.325341
H 2.762435 0.634797 -2.420235
C -1.091557 1.148358 -0.175353
C 2.266073 -1.307139 2.127885
S 0.717186 -1.225774 2.208807
S 3.815128 -1.389429 2.052277
H 1.462413 2.527954 -2.267681
H 1.674787 1.783069 1.958571
C 3.109480 3.535591 -1.258591
H 3.831794 2.733438 -1.457101
H 3.234516 4.271285 -2.056981
C 3.198714 3.166769 1.237956
H 3.339037 3.653486 2.206505
H 3.965267 2.384813 1.183833
C 3.407727 4.170254 0.101943
H 2.733367 5.022566 0.249183
H 4.427267 4.563199 0.126124
H 0.967252 3.782504 -1.143147
H 1.054115 3.332060 1.381802
C 2.948173 -1.290826 -1.491177
C 4.318563 -1.417233 -1.706111
C 2.225857 -2.403057 -1.060178
C 4.963252 -2.630721 -1.480393

H 4.886601 -0.557195 -2.049522
C 2.862608 -3.618156 -0.840505
H 1.159713 -2.303311 -0.881857
C 4.235721 -3.732417 -1.044763
H 6.030596 -2.716025 -1.648105
H 2.291020 -4.475823 -0.504892
H 4.734190 -4.678397 -0.869352
C 1.473445 1.918792 -0.181388
C -2.763687 -0.648781 -0.132032
C -3.495740 1.655231 -0.141858
C -4.084793 -1.068246 -0.106522
H -1.956896 -1.371481 -0.135678
C -4.816820 1.235655 -0.115643
H -3.256680 2.711146 -0.152640
C -5.107138 -0.124649 -0.096752
H -4.322901 -2.124911 -0.086795
H -5.621356 1.960536 -0.102257
C -6.535831 -0.581884 -0.127864
F -6.965469 -0.781635 -1.386569
F -6.708106 -1.740489 0.524677
F -7.364050 0.315807 0.424534
H 3.252307 0.892537 -0.041623

TS1 with trifluoromethylphenyl (Figure 5) C23H22F3NS2
-2040.13117478 -2039.726626 -2039.699660 -2039.787403
C 1.688124 2.329791 -1.546600
C 1.640734 2.629753 0.955451
C 1.830599 -0.722473 -0.917632
C -2.612574 0.613402 -0.196962
C -0.062374 1.267284 -0.195260
H 0.941336 -1.194101 -0.493001
N 2.181181 0.398113 -0.017513
H 1.567615 -0.340580 -1.906761
C -1.236967 1.009256 -0.228044
C 2.280983 -0.312283 1.787185
S 0.849615 -0.911694 2.209413
S 3.808835 -0.108721 2.257763
H 1.508853 1.643457 -2.376362
H 1.459201 2.132690 1.909936
C 3.102991 2.907459 -1.591534
H 3.851837 2.107235 -1.529293
H 3.256562 3.390185 -2.559823
C 3.045708 3.235549 0.898460
H 3.143451 3.961235 1.709474
H 3.797656 2.463971 1.089820
C 3.319065 3.901112 -0.450103
H 2.638488 4.750399 -0.584335
H 4.336363 4.298203 -0.477961
H 0.964595 3.143435 -1.658847
H 0.898684 3.428995 0.862781
C 2.976222 -1.695325 -1.021011
C 4.039964 -1.421934 -1.881705
C 3.007248 -2.852426 -0.245815
C 5.124565 -2.287195 -1.961840
H 4.014280 -0.527956 -2.499772
C 4.089856 -3.721460 -0.327073

H 2.186953 -3.063469 0.431886
C 5.149956 -3.439273 -1.181640
H 5.945025 -2.066806 -2.634304
H 4.106945 -4.616923 0.282593
H 5.993824 -4.116128 -1.241790
C 1.357236 1.638364 -0.196696
C -2.981051 -0.511784 0.548791
C -3.588297 1.329665 -0.900368
C -4.306427 -0.920949 0.584882
H -2.218126 -1.052787 1.095277
C -4.912062 0.923175 -0.858787
H -3.300007 2.201894 -1.473521
C -5.265963 -0.201206 -0.117522
H -4.594763 -1.790005 1.162827
H -5.672858 1.477092 -1.395963
C -6.697787 -0.652010 -0.130317
F -7.006933 -1.281235 -1.277879
F -6.973413 -1.501237 0.866993
F -7.545957 0.382390 -0.022256
H 3.168854 0.631608 -0.121990

State 3 with trifluoromethylphenyl (Figure 5) C23H22F3NS2
-2040.13603472 -2039.729679 -2039.702733 -2039.789743
C 1.717254 2.164419 -1.627167
C 1.574580 2.689355 0.837207
C 1.803586 -0.841977 -0.773829
C -2.619721 0.541634 -0.276222
C -0.073762 1.205948 -0.253567
H 0.930550 -1.261494 -0.276633
N 2.199626 0.362304 0.046922
H 1.514661 -0.490196 -1.762526
C -1.243821 0.935821 -0.318504
C 2.361113 -0.051362 1.564304
S 0.991116 -0.604433 2.302961
S 3.932297 0.104553 2.057301
H 1.587936 1.401900 -2.397220
H 1.389945 2.267454 1.825856
C 3.112932 2.787358 -1.680044
H 3.893332 2.031940 -1.516016
H 3.280659 3.175790 -2.687547
C 2.954215 3.349225 0.756873
H 2.992228 4.150524 1.498477
H 3.728721 2.630825 1.039063
C 3.246141 3.896825 -0.638608
H 2.538452 4.699152 -0.879954
H 4.247584 4.331312 -0.672829
H 0.975045 2.939443 -1.840623
H 0.800783 3.443615 0.662290
C 2.944030 -1.817234 -0.864641
C 3.905587 -1.663101 -1.863365
C 3.068837 -2.857116 0.054955
C 4.986083 -2.533790 -1.940729
H 3.804427 -0.860473 -2.589474
C 4.147488 -3.729802 -0.023892
H 2.328953 -2.967644 0.840901
C 5.106557 -3.568578 -1.018737

H 5.729193 -2.408000 -2.719012
H 4.243216 -4.531829 0.697831
H 5.948045 -4.248699 -1.075403
C 1.334329 1.602700 -0.234496
C -3.098797 -0.121841 0.859452
C -3.480985 0.798666 -1.348045
C -4.425349 -0.520718 0.921536
H -2.418001 -0.320689 1.678356
C -4.807310 0.400075 -1.283518
H -3.103297 1.307125 -2.226173
C -5.272873 -0.259055 -0.150376
H -4.800018 -1.040121 1.794891
H -5.478272 0.590748 -2.112117
C -6.722524 -0.642027 -0.065292
F -7.232244 -0.942543 -1.268456
F -6.917151 -1.702357 0.729117
F -7.470165 0.358508 0.431427
H 3.164365 0.606245 -0.203340

State 5 with methoxyphenyl (Figure 5) C29H37N3OS2
-2162.89866377 -2162.277600 -2162.244106 -2162.345486
C -2.320287 -2.558093 -1.035008
C -2.229575 -2.177326 1.430376
C -2.931562 0.250117 -1.210370
C 2.343022 -2.041346 -0.197490
C -0.264301 -1.806862 -0.053553
H -2.476277 1.118722 -1.689304
N -2.041010 -0.167599 -0.120809
H -2.989760 -0.534845 -1.956709
H 0.938877 1.720063 1.047104
C 0.913647 -2.003182 -0.217604
C -1.355230 0.816181 0.557089
S -1.492928 2.452195 0.067334
S -0.358593 0.433145 1.919115
C 3.130456 1.957482 -1.520166
C 1.744416 1.664484 -0.894084
H 3.013804 2.490064 -2.465470
H 3.671518 1.030015 -1.717297
H 0.934802 2.241928 -1.338519
H 1.464336 0.608794 -0.912473
C 3.177728 3.954227 -0.207259
H 3.830817 4.624381 0.354200
H 2.860294 4.477546 -1.111142
C 1.949583 3.547316 0.649622
H 2.075938 3.779633 1.708252
H 1.007428 3.976466 0.309968
C 2.972765 1.393568 1.195924
H 2.887133 1.566980 2.269460
H 2.857885 0.325759 1.009563
C 4.266005 1.977228 0.575215
H 4.788036 2.621426 1.285932
H 4.942329 1.167581 0.293259
N 1.814880 2.069913 0.541938
N 3.945662 2.772799 -0.612759
H -1.996786 -2.222075 -2.022256
H -1.875693 -1.531608 2.228329

C -3.830109 -2.810960 -0.969878
H -4.402389 -1.910357 -1.200516
H -4.082947 -3.550422 -1.735682
C -3.747258 -2.338438 1.482170
H -4.028157 -2.688687 2.478874
H -4.227274 -1.365103 1.341806
C -4.243294 -3.309200 0.413706
H -3.807876 -4.300323 0.592828
H -5.329494 -3.419503 0.463950
H -1.817860 -3.517046 -0.873867
H -1.755881 -3.156316 1.570020
C -4.337055 0.632176 -0.787851
C -5.352051 0.588057 -1.744476
C -4.647463 1.061150 0.500423
C -6.653095 0.959397 -1.424885
H -5.121126 0.252743 -2.751966
C -5.950118 1.428023 0.824615
H -3.869094 1.111899 1.252947
C -6.956626 1.379673 -0.134015
H -7.428947 0.913135 -2.180415
H -6.176139 1.759032 1.831635
H -7.969659 1.666552 0.122623
C -1.725344 -1.632983 0.068444
C 3.015517 -2.284574 1.013856
C 3.104164 -1.742667 -1.327867
C 4.392211 -2.200703 1.088620
H 2.431848 -2.513687 1.897577
C 4.494168 -1.655186 -1.262901
H 2.600993 -1.564848 -2.271861
C 5.142079 -1.873448 -0.047649
H 4.917993 -2.378379 2.018863
H 5.050580 -1.418317 -2.160123
O 6.483882 -1.788059 0.131371
C 7.280042 -1.472698 -0.993137
H 7.174769 -2.230100 -1.775824
H 7.019764 -0.489000 -1.398325
H 8.308149 -1.457323 -0.639372

TS3 with methoxyphenyl (Figure 5) C29H37N3OS2
-2162.88367057 -2162.262863 -2162.230301 -2162.326714
C 2.109432 -1.986961 1.621210
C 2.476323 -2.159130 -0.887665
C 3.012483 0.769985 0.969031
C -2.120077 -2.104696 0.214181
C 0.251251 -1.526379 0.065623
H 2.638080 1.780030 1.150703
N 2.006339 0.090006 0.155326
H 3.085389 0.265368 1.930248
H -1.097534 1.879553 -0.785816
C -0.730119 -2.143293 0.598531
C 1.195703 0.801448 -0.647399
S 1.242938 2.497206 -0.812528
S 0.004944 -0.139207 -1.487030
C -3.030155 1.736779 1.942237
C -1.699682 1.569156 1.166411
H -2.847032 2.183191 2.920818

H -3.503934 0.765527 2.091189
H -0.879056 2.162185 1.573055
H -1.379565 0.528026 1.077189
C -3.285799 3.833317 0.830852
H -4.015219 4.530017 0.415022
H -2.879411 4.278940 1.740783
C -2.158531 3.556964 -0.194657
H -2.425384 3.855872 -1.209361
H -1.202838 4.013258 0.063153
C -3.133190 1.384107 -0.805056
H -3.163995 1.616386 -1.869773
H -2.965104 0.313708 -0.683350
C -4.373656 1.888154 -0.025891
H -4.967118 2.577510 -0.630137
H -5.003374 1.038571 0.240024
N -1.943219 2.080528 -0.221414
N -3.962269 2.588289 1.195480
H 1.715909 -1.335351 2.406305
H 2.358928 -1.606578 -1.821903
C 3.582650 -2.345775 1.845393
H 4.217791 -1.459481 1.924675
H 3.662053 -2.873955 2.800043
C 3.948215 -2.476909 -0.619663
H 4.325187 -3.085693 -1.446342
H 4.547362 -1.562222 -0.606356
C 4.118505 -3.211818 0.707210
H 3.567455 -4.159767 0.676689
H 5.170594 -3.453300 0.879717
H 1.520123 -2.904399 1.685622
H 1.933325 -3.103196 -1.007341
C 4.381563 0.869296 0.328870
C 5.519936 0.810371 1.130734
C 4.530945 1.049687 -1.045869
C 6.790028 0.919406 0.572395
H 5.414100 0.667814 2.202242
C 5.798796 1.157535 -1.605488
H 3.653446 1.096043 -1.680388
C 6.932104 1.090802 -0.799800
H 7.665806 0.864409 1.208468
H 5.901895 1.293073 -2.675818
H 7.919197 1.171355 -1.239583
C 1.746968 -1.393149 0.245236
C -2.527701 -2.279117 -1.125727
C -3.125942 -1.842409 1.153851
C -3.855291 -2.156132 -1.499727
H -1.776819 -2.511758 -1.872914
C -4.464172 -1.698442 0.786812
H -2.845063 -1.756041 2.198391
C -4.832816 -1.848860 -0.549381
H -4.165268 -2.292498 -2.529345
H -5.203247 -1.495626 1.552370
O -6.110469 -1.721227 -1.017671
C -7.140694 -1.582559 -0.065102
H -7.047405 -0.645941 0.496644
H -8.073214 -1.569943 -0.625426
H -7.146847 -2.423292 0.636131

State 6 with methoxyphenyl (Figure 5) C29H37N3OS2

-2162.88404636 -2162.262772 -2162.229739 -2162.327369

C 2.054984 -1.878785 1.688389
C 2.510375 -2.153483 -0.799138
C 3.006321 0.852132 0.909501
C -2.082051 -2.111531 0.185923
C 0.241126 -1.479862 0.030390
H 2.639291 1.874273 1.029288
N 1.995207 0.132358 0.137609
H 3.071837 0.402474 1.898725
H -1.085801 1.932676 -0.741604
C -0.699041 -2.162742 0.615127
C 1.177178 0.795856 -0.684989
S 1.197088 2.471242 -0.979042
S -0.032724 -0.218482 -1.418112
C -3.014819 1.694023 1.987296
C -1.687716 1.543163 1.200543
H -2.828180 2.125767 2.971749
H -3.481524 0.717729 2.123039
H -0.862660 2.121262 1.620616
H -1.375104 0.502320 1.084770
C -3.292888 3.811999 0.925134
H -4.028588 4.508736 0.520624
H -2.894710 4.243568 1.845388
C -2.159629 3.569393 -0.102060
H -2.421686 3.896505 -1.109267
H -1.207635 4.023254 0.174157
C -3.122965 1.407929 -0.775326
H -3.159366 1.676534 -1.831362
H -2.947639 0.334875 -0.689206
C -4.364661 1.877788 0.024293
H -4.969879 2.574322 -0.559636
H -4.981334 1.013749 0.272693
N -1.936179 2.094626 -0.171598
N -3.955292 2.552602 1.260425
H 1.646279 -1.184815 2.428932
H 2.429853 -1.631540 -1.755951
C 3.517414 -2.236730 1.973648
H 4.156637 -1.350993 2.032229
H 3.565982 -2.721697 2.952965
C 3.968985 -2.478447 -0.470349
H 4.362950 -3.129675 -1.255788
H 4.583811 -1.574352 -0.476350
C 4.082141 -3.156745 0.892806
H 3.519439 -4.098162 0.883433
H 5.124430 -3.403363 1.112189
H 1.448291 -2.782814 1.781286
H 1.963537 -3.096782 -0.905841
C 4.375233 0.897213 0.264861
C 5.514816 0.842716 1.064965
C 4.522127 1.019815 -1.116419
C 6.784321 0.897585 0.497592
H 5.410380 0.744854 2.141505
C 5.789356 1.072703 -1.684820
H 3.643171 1.063874 -1.749174

C 6.924190 1.008896 -0.880927
H 7.661496 0.846232 1.132004
H 5.891061 1.162014 -2.760124
H 7.911010 1.045596 -1.326860
C 1.739307 -1.352437 0.279662
C -2.480948 -2.297454 -1.156196
C -3.103337 -1.840982 1.109446
C -3.803592 -2.167090 -1.547763
H -1.729177 -2.555385 -1.894875
C -4.436297 -1.690817 0.726452
H -2.835123 -1.759688 2.157965
C -4.790611 -1.844498 -0.613284
H -4.101932 -2.313264 -2.579630
H -5.183825 -1.484328 1.483221
O -6.063297 -1.710057 -1.098561
C -7.105042 -1.586569 -0.157461
H -7.025615 -0.654456 0.414210
H -8.031503 -1.575183 -0.728040
H -7.113443 -2.433167 0.536843

State 5 with phenyl (Figure 5) C28H35N3S2
-2048.38622911 -2047.797017 -2047.766368 -2047.858853
C -2.122694 2.471337 1.067186
C -2.001587 2.123667 -1.409110
C -2.473683 -0.386655 1.184217
C 2.571414 2.392048 0.229921
C -0.006368 1.946396 0.072971
H -1.951045 -1.213246 1.668592
N -1.595962 0.133494 0.130043
H -2.631436 0.376845 1.938604
H 1.305183 -2.162791 -0.602784
C 1.145855 2.252884 0.248727
C -0.806461 -0.759526 -0.559016
S -0.776299 -2.418886 -0.056579
S 0.146790 -0.288290 -1.899011
C 3.822766 -0.908859 1.231932
C 2.337780 -1.114268 0.852675
H 3.943397 -0.912531 2.316465
H 4.190224 0.048632 0.857117
H 1.737406 -1.542515 1.656212
H 1.844817 -0.210331 0.494534
C 4.054882 -3.273886 0.972971
H 4.746608 -4.067251 0.685182
H 3.918766 -3.325819 2.054946
C 2.700538 -3.447171 0.240013
H 2.767931 -4.110056 -0.623866
H 1.892546 -3.795494 0.884110
C 3.230569 -1.669827 -1.354914
H 3.030639 -2.289719 -2.229184
H 2.960609 -0.642067 -1.592916
C 4.672270 -1.807061 -0.804152
H 5.182689 -2.668794 -1.240351
H 5.249361 -0.913099 -1.047102
N 2.303999 -2.104634 -0.269902
N 4.649905 -1.975222 0.651356
H -1.771942 2.144378 2.048444

H -1.599366 1.505839 -2.206620
C -3.648701 2.588937 1.000603
H -4.138267 1.634649 1.204200
H -3.971110 3.284127 1.781384
C -3.527962 2.160678 -1.454362
H -3.838871 2.496402 -2.447171
H -3.929053 1.150950 -1.322281
C -4.099096 3.076412 -0.375068
H -3.741858 4.100795 -0.538971
H -5.190502 3.101932 -0.425823
H -1.708815 3.474967 0.927638
H -1.609933 3.137246 -1.555016
C -3.821027 -0.895564 0.706756
C -4.873962 -0.950415 1.620509
C -4.036584 -1.345534 -0.594085
C -6.120456 -1.440220 1.246796
H -4.717480 -0.598791 2.636671
C -5.284341 -1.831993 -0.971903
H -3.228736 -1.314296 -1.316275
C -6.329478 -1.881858 -0.055659
H -6.927894 -1.471452 1.969168
H -5.436759 -2.177881 -1.987660
H -7.299824 -2.260432 -0.354537
C -1.442416 1.625719 -0.050459
C 3.252351 2.111706 -0.964955
C 3.305842 2.741313 1.369044
C 4.639364 2.177841 -1.011939
H 2.668881 1.845862 -1.840248
C 4.693933 2.807459 1.313835
H 2.779097 2.959645 2.290312
C 5.364625 2.524104 0.127302
H 5.156764 1.968703 -1.941646
H 5.254214 3.080552 2.200372
H 6.446063 2.578399 0.088444

TS3 with phenyl (Figure 5) C28H35N3S2
-2048.37254850 -2047.784435 -2047.754402 -2047.845010
C -1.857699 1.993871 1.560738
C -2.237927 2.063414 -0.949120
C -2.509109 -0.848645 1.011216
C 2.353540 2.430384 0.116825
C 0.028947 1.648701 0.016888
H -2.040792 -1.813456 1.219041
N -1.582365 -0.109980 0.154544
H -2.612114 -0.319748 1.956198
H 1.677774 -1.591452 -0.830480
C 0.972699 2.345338 0.502120
C -0.726683 -0.777649 -0.642864
S -0.640335 -2.475178 -0.752057
S 0.368722 0.225035 -1.539036
C 3.617388 -1.351438 1.884832
C 2.272378 -1.266417 1.119875
H 3.472819 -1.822936 2.858031
H 4.025182 -0.352262 2.045084
H 1.496311 -1.916425 1.526839
H 1.881309 -0.249451 1.040502

C 4.013434 -3.411591 0.745499
H 4.787322 -4.045072 0.309319
H 3.655612 -3.899586 1.654169
C 2.852129 -3.208346 -0.260208
H 3.120879 -3.483773 -1.281021
H 1.933954 -3.728680 0.010979
C 3.669896 -0.968945 -0.865802
H 3.709317 -1.202304 -1.930065
H 3.425013 0.087245 -0.746423
C 4.949243 -1.381969 -0.095276
H 5.590411 -2.017782 -0.709515
H 5.513548 -0.490039 0.177643
N 2.537389 -1.749638 -0.273900
N 4.599804 -2.125040 1.118932
H -1.402534 1.408227 2.364275
H -2.067773 1.503399 -1.870303
C -3.358001 2.219872 1.780042
H -3.904853 1.279330 1.882818
H -3.486136 2.762456 2.721068
C -3.732906 2.247512 -0.685760
H -4.168354 2.791874 -1.528398
H -4.240357 1.279775 -0.642640
C -3.972339 3.000454 0.620322
H -3.513183 3.994932 0.561540
H -5.042517 3.146663 0.788282
H -1.358299 2.965065 1.592861
H -1.783746 3.050693 -1.089419
C -3.874684 -1.096654 0.404533
C -4.995896 -1.124855 1.232066
C -4.035586 -1.330610 -0.960819
C -6.261127 -1.372513 0.708479
H -4.880686 -0.941563 2.296384
C -5.298878 -1.576555 -1.485996
H -3.171376 -1.312757 -1.614576
C -6.415616 -1.596021 -0.655039
H -7.124277 -1.382512 1.363767
H -5.411705 -1.752661 -2.549365
H -7.399664 -1.782200 -1.068862
C -1.446566 1.390823 0.201301
C 2.726117 2.675114 -1.220043
C 3.384245 2.220471 1.053859
C 4.062794 2.676212 -1.601199
H 1.946471 2.856437 -1.951598
C 4.717651 2.208945 0.664728
H 3.113511 2.078690 2.095084
C 5.068584 2.431144 -0.667096
H 4.322516 2.867045 -2.636881
H 5.492152 2.047167 1.408205
H 6.110107 2.440287 -0.966712

State 6 with phenyl (Figure 5) C28H35N3S2
-2048.37359373 -2047.784900 -2047.754559 -2047.845396
C -1.778309 1.855952 1.651567
C -2.312543 2.014009 -0.831689
C -2.515533 -0.967296 0.963772
C 2.267918 2.442957 0.022545

C 0.024863 1.568628 -0.044414
H -2.063329 -1.951021 1.110183
N -1.574320 -0.188927 0.161074
H -2.611841 -0.494171 1.939450
H 1.663923 -1.745424 -0.738180
C 0.902207 2.375339 0.481676
C -0.707028 -0.805144 -0.646571
S -0.572273 -2.482820 -0.884692
S 0.395289 0.292550 -1.429335
C 3.650446 -1.212951 1.906017
C 2.287369 -1.236425 1.168423
H 3.546707 -1.627650 2.909757
H 4.012681 -0.188390 1.991931
H 1.551567 -1.893443 1.635385
H 1.852041 -0.242356 1.039220
C 4.119141 -3.322746 0.895502
H 4.912089 -3.950933 0.486793
H 3.797332 -3.763498 1.841078
C 2.935562 -3.235602 -0.099956
H 3.203677 -3.558686 -1.106854
H 2.048761 -3.784564 0.217638
C 3.630573 -1.002494 -0.864145
H 3.657576 -1.296854 -1.913671
H 3.346725 0.049256 -0.798817
C 4.942138 -1.315932 -0.101307
H 5.593814 -1.966007 -0.689111
H 5.474895 -0.386123 0.096883
N 2.547212 -1.796461 -0.198552
N 4.652067 -1.989384 1.168505
H -1.297433 1.218821 2.400106
H -2.208850 1.470476 -1.774271
C -3.259934 2.095819 1.961559
H -3.816926 1.159567 2.058415
H -3.329662 2.602312 2.928537
C -3.785729 2.224282 -0.476081
H -4.251666 2.815210 -1.269772
H -4.320472 1.271405 -0.441121
C -3.925424 2.931521 0.869875
H -3.446202 3.916889 0.819608
H -4.979730 3.095576 1.107857
H -1.248767 2.810257 1.707255
H -1.851419 2.996635 -0.980076
C -3.880460 -1.142380 0.331877
C -5.016804 -1.145880 1.138412
C -4.024287 -1.321461 -1.043601
C -6.281829 -1.311364 0.582365
H -4.914345 -1.004860 2.210246
C -5.287186 -1.484432 -1.600820
H -3.147089 -1.323652 -1.680388
C -6.419765 -1.476545 -0.791031
H -7.157528 -1.302230 1.220781
H -5.387595 -1.615871 -2.671885
H -7.403487 -1.597433 -1.229200
C -1.450122 1.317754 0.250597
C 2.610123 2.677949 -1.326264
C 3.332658 2.250821 0.929035

C 3.935672 2.675249 -1.745830
H 1.815207 2.865758 -2.040497
C 4.654335 2.236641 0.503870
H 3.091709 2.133649 1.980966
C 4.969908 2.439993 -0.840447
H 4.165163 2.858425 -2.790264
H 5.449345 2.089673 1.229264
H 6.002393 2.449416 -1.169619

State 5 with trifluoromethylphenyl (Figure 5) C29H34F3N3S2
-2385.43834971 -2384.845574 -2384.811076 -2384.916195
C 2.661267 -2.594076 1.087565
C 2.521111 -2.233925 -1.382018
C 3.381742 0.197587 1.194701
C -1.992994 -1.887322 0.349706
C 0.610518 -1.783548 0.152478
H 2.977339 1.089643 1.675923
N 2.430984 -0.205498 0.151857
H 3.442763 -0.576396 1.952520
H -0.553501 1.766369 -0.981832
C -0.566849 -1.934295 0.364958
C 1.741652 0.787733 -0.509268
S 1.947630 2.423704 -0.054709
S 0.674099 0.405326 -1.818134
C -2.694455 2.338241 1.575802
C -1.333491 1.916043 0.969671
H -2.539437 2.893590 2.502224
H -3.308504 1.464547 1.804544
H -0.487231 2.474860 1.367070
H -1.116574 0.849935 1.068183
C -2.562808 4.258700 0.162338
H -3.156240 4.975198 -0.407773
H -2.164727 4.776146 1.037359
C -1.411981 3.689866 -0.707998
H -1.569524 3.851888 -1.775505
H -0.424101 4.062572 -0.440106
C -2.616453 1.593470 -1.077424
H -2.526372 1.651578 -2.163014
H -2.604392 0.542021 -0.786270
C -3.839283 2.358632 -0.514425
H -4.272132 3.018124 -1.269565
H -4.613455 1.656018 -0.202248
N -1.393223 2.216252 -0.491559
N -3.437085 3.178988 0.631000
H 2.378281 -2.232446 2.078195
H 2.171711 -1.580602 -2.176087
C 4.157917 -2.905169 0.985061
H 4.769821 -2.025578 1.194348
H 4.400830 -3.647823 1.750818
C 4.030042 -2.452135 -1.469512
H 4.270998 -2.823358 -2.469054
H 4.549890 -1.496413 -1.353186
C 4.516653 -3.430200 -0.403536
H 4.042598 -4.406904 -0.562331
H 5.596476 -3.579891 -0.479862
H 2.119504 -3.535947 0.953707

H 2.008858 -3.195878 -1.503196
C 4.779908 0.522187 0.705579
C 5.836019 0.444749 1.614246
C 5.044915 0.934613 -0.598326
C 7.133075 0.768608 1.232329
H 5.640858 0.121812 2.633284
C 6.343086 1.253750 -0.984780
H 4.234909 1.008824 -1.314690
C 7.390635 1.173538 -0.073294
H 7.941047 0.698242 1.951346
H 6.533539 1.572512 -2.003028
H 8.399886 1.424448 -0.377729
C 2.072259 -1.660741 -0.012558
C -2.676009 -2.185339 -0.840326
C -2.723260 -1.460124 1.466680
C -4.051194 -2.032662 -0.915993
H -2.106813 -2.510338 -1.702306
C -4.100484 -1.301134 1.388328
H -2.199764 -1.247334 2.391420
C -4.759737 -1.581767 0.195863
H -4.577170 -2.259824 -1.835928
H -4.664468 -0.972459 2.253385
C -6.236923 -1.350882 0.079813
F -6.833816 -2.273957 -0.684771
F -6.847092 -1.355916 1.271230
F -6.505376 -0.155259 -0.486256

TS3 with trifluoromethylphenyl (Figure 5) C29H34F3N3S2
-2385.43000355 -2384.836730 -2384.803081 -2384.903091
C 2.502600 -1.986338 1.687434
C 2.682948 -2.252319 -0.833133
C 3.434931 0.711921 0.912074
C -1.842475 -1.970671 0.518188
C 0.568812 -1.519831 0.268387
H 3.104272 1.738590 1.083921
N 2.369507 0.049288 0.160003
H 3.533316 0.231320 1.883043
H -0.752718 1.892799 -0.831256
C -0.446063 -2.026224 0.810811
C 1.562291 0.776446 -0.644893
S 1.703748 2.462279 -0.846628
S 0.307291 -0.107195 -1.447722
C -2.445628 2.114502 2.044033
C -1.201778 1.797329 1.179047
H -2.151509 2.632986 2.957852
H -2.960275 1.194447 2.324761
H -0.313902 2.363439 1.463400
H -0.950610 0.734738 1.149901
C -2.688648 4.121307 0.773593
H -3.416776 4.814556 0.350064
H -2.191803 4.625509 1.604622
C -1.656952 3.696136 -0.303149
H -1.980508 3.932752 -1.317695
H -0.660002 4.107902 -0.148919
C -2.804156 1.549613 -0.655061
H -2.917441 1.717378 -1.726281

H -2.677413 0.480452 -0.479408
C -3.944096 2.175558 0.188860
H -4.562840 2.838101 -0.419331
H -4.587992 1.389709 0.582170
N -1.530820 2.210607 -0.223996
N -3.391132 2.952299 1.301358
H 2.181612 -1.303397 2.478463
H 2.517720 -1.729108 -1.775952
C 3.975461 -2.386758 1.822953
H 4.639281 -1.518956 1.824863
H 4.103508 -2.883446 2.789010
C 4.159545 -2.608132 -0.652649
H 4.462761 -3.249416 -1.484719
H 4.782098 -1.710827 -0.704491
C 4.400009 -3.308361 0.682085
H 3.817960 -4.237230 0.722102
H 5.453183 -3.580364 0.788190
H 1.897522 -2.887185 1.815819
H 2.103116 -3.180914 -0.879386
C 4.779897 0.750852 0.216304
C 5.943936 0.707971 0.981983
C 4.886194 0.863489 -1.169522
C 7.196176 0.767212 0.378532
H 5.871795 0.618789 2.062211
C 6.136559 0.920160 -1.774522
H 3.989030 0.898299 -1.776691
C 7.295061 0.870710 -1.004314
H 8.091620 0.726542 0.987744
H 6.205761 1.003247 -2.852929
H 8.267916 0.912592 -1.479572
C 2.066542 -1.415425 0.317606
C -2.313004 -2.280029 -0.774126
C -2.778358 -1.533204 1.476295
C -3.646486 -2.102205 -1.107414
H -1.607479 -2.641751 -1.512572
C -4.110373 -1.349061 1.141387
H -2.436011 -1.343695 2.487510
C -4.544749 -1.620961 -0.155959
H -3.992930 -2.327902 -2.109351
H -4.820721 -1.007235 1.886734
C -5.954531 -1.319360 -0.545092
F -6.396511 -2.098595 -1.540239
F -6.810180 -1.448107 0.479889
F -6.085868 -0.040561 -0.981230

State 6 with trifluoromethylphenyl (Figure 5) C29H34F3N3S2
-2385.43325822 -2384.839363 -2384.805510 -2384.905046
C 2.485246 -1.802920 1.788199
C 2.790827 -2.181930 -0.711649
C 3.448372 0.874667 0.834007
C -1.707625 -2.049529 0.501722
C 0.586428 -1.431491 0.212757
H 3.115192 1.911139 0.925618
N 2.369696 0.147781 0.166843
H 3.565058 0.470536 1.838261
H -0.771875 2.022255 -0.696408

C -0.321657 -2.115372 0.855866
C 1.508823 0.795671 -0.620925
S 1.537143 2.446985 -1.009405
S 0.226863 -0.229051 -1.207949
C -2.611957 2.011387 2.100831
C -1.314109 1.789605 1.284666
H -2.387699 2.511930 3.043899
H -3.083221 1.053885 2.324976
H -0.472492 2.391887 1.630644
H -1.010125 0.741130 1.237999
C -2.907507 4.046396 0.892271
H -3.651302 4.718859 0.462613
H -2.472294 4.541269 1.762556
C -1.813920 3.714289 -0.153644
H -2.111817 3.965075 -1.172598
H -0.849309 4.178916 0.052460
C -2.818780 1.520203 -0.630777
H -2.893081 1.717517 -1.700271
H -2.647806 0.453414 -0.479166
C -4.026858 2.059765 0.177673
H -4.650440 2.711533 -0.436977
H -4.645692 1.228800 0.513787
N -1.604128 2.235165 -0.117919
N -3.567568 2.822624 1.342669
H 2.141291 -1.066795 2.521043
H 2.670566 -1.688706 -1.679816
C 3.953154 -2.188522 1.999378
H 4.613830 -1.316945 1.978586
H 4.051168 -2.631787 2.994399
C 4.255949 -2.538673 -0.453396
H 4.585162 -3.230156 -1.234117
H 4.895298 -1.655270 -0.528217
C 4.427155 -3.167839 0.927178
H 3.838035 -4.090962 0.987303
H 5.472603 -3.437953 1.097269
H 1.863445 -2.686069 1.957626
H 2.214775 -3.112738 -0.756364
C 4.772781 0.846548 0.101877
C 5.958483 0.790866 0.831485
C 4.834155 0.900667 -1.290230
C 7.190259 0.777600 0.184550
H 5.919853 0.746131 1.915731
C 6.063638 0.883639 -1.938061
H 3.918224 0.946325 -1.868082
C 7.244839 0.818922 -1.204035
H 8.104119 0.726239 0.764709
H 6.099470 0.919137 -3.020517
H 8.201419 0.800081 -1.712457
C 2.099708 -1.326655 0.380633
C -2.180013 -2.391249 -0.787938
C -2.666318 -1.594936 1.437115
C -3.503366 -2.196242 -1.143799
H -1.478847 -2.792620 -1.511268
C -3.990692 -1.400242 1.081889
H -2.339201 -1.405345 2.453872
C -4.412520 -1.677330 -0.219080

H -3.837156 -2.433828 -2.148285
H -4.705990 -1.042077 1.814981
C -5.795007 -1.344357 -0.661476
F -6.336520 -2.283658 -1.450530
F -6.636737 -1.148026 0.365614
F -5.823205 -0.193687 -1.388395

State 5 with propyl and DABCO (Figure 6) C16H29N3S2
-1587.54984685 -1587.107110 -1587.082417 -1587.162262
C 3.675832 1.407889 -1.336089
C 3.966161 0.873670 1.097199
C 3.548721 -1.342271 -1.341001
C -0.201568 3.302114 0.493615
C 3.007874 0.764478 -0.098836
C 1.791734 1.576544 0.119441
H 3.304523 -1.075547 -2.372041
H 3.791681 2.472348 -1.133656
H 4.662386 0.989753 -1.533458
H 3.047258 1.295034 -2.220474
N 2.680033 -0.666020 -0.378274
H 4.246084 1.917512 1.253927
H 3.494401 0.486687 1.997758
H 4.869372 0.295505 0.884358
H 3.424877 -2.413384 -1.226858
H 4.589220 -1.081400 -1.136869
H -0.620529 -1.203814 0.905060
C 0.913882 2.393056 0.229216
H 0.179154 4.321644 0.614300
H -0.654540 3.030705 1.455886
C 1.730077 -1.379125 0.296613
S 1.183084 -2.875386 -0.331492
S 1.092509 -0.770452 1.786045
C -2.762484 -0.330823 -1.590850
C -1.369613 -0.678358 -1.004452
H -2.924361 -0.867567 -2.526850
H -2.851020 0.736619 -1.803539
H -0.884344 -1.515545 -1.503771
H -0.669189 0.160482 -0.992724
C -3.684523 -2.110189 -0.290492
H -4.541460 -2.404040 0.317865
H -3.712049 -2.697002 -1.210159
C -2.356410 -2.357029 0.474742
H -2.509667 -2.587747 1.530054
H -1.728312 -3.133456 0.039059
C -2.279301 -0.012512 1.160461
H -2.284106 -0.296458 2.213742
H -1.666542 0.884890 1.056010
C -3.698934 0.126601 0.553299
H -4.460731 -0.192694 1.267335
H -3.902562 1.166943 0.290309
N -1.575751 -1.092707 0.415404
N -3.826241 -0.696237 -0.650844
C -1.275697 3.292795 -0.601472
C -2.470747 4.166414 -0.233203
H -2.932855 3.823580 0.697285
H -1.604526 2.262354 -0.767113

H -0.830491 3.627227 -1.542206
H -2.164455 5.204869 -0.084610
H -3.233017 4.151119 -1.014608

TS3 with propyl and DABCO (Figure 6) C16H29N3S2
-1587.52733803 -1587.084880 -1587.061105 -1587.136767
C -1.962103 1.315082 2.314304
C -3.236865 2.277928 0.372350
C -3.416521 -0.852338 1.144666
C 1.344522 2.706222 -0.491389
C -2.137005 1.294067 0.792717
C -0.827865 1.663265 0.153899
H -2.899418 -1.334341 1.980862
H -1.561883 2.287536 2.596293
H -2.911541 1.155057 2.828859
H -1.246842 0.554007 2.633148
N -2.494435 -0.072592 0.331813
H -2.970662 3.280427 0.711782
H -3.339036 2.287329 -0.713128
H -4.194355 1.997345 0.819540
H -3.871453 -1.618777 0.523879
H -4.192032 -0.196441 1.543984
H 0.496067 0.008552 -0.125940
C 0.229624 2.320187 0.416207
H 0.968492 3.489896 -1.161893
H 1.595369 1.865428 -1.168505
C -2.069136 -0.498305 -0.878849
S -2.330737 -2.067344 -1.454998
S -1.157963 0.683931 -1.770916
C 1.263705 -2.983069 1.181999
C 0.408260 -1.699904 1.054435
H 0.785481 -3.809338 0.652679
H 1.368562 -3.269040 2.229632
H -0.588222 -1.900699 0.662050
H 0.333497 -1.140673 1.988518
C 2.461971 -2.618066 -0.848002
H 3.440351 -2.344273 -1.248247
H 2.179313 -3.577379 -1.283135
C 1.399714 -1.544565 -1.191024
H 1.740730 -0.810305 -1.920799
H 0.448500 -1.956052 -1.535472
C 2.381917 -0.305583 0.688923
H 2.909303 0.252881 -0.086081
H 2.105875 0.387257 1.485740
C 3.169876 -1.551513 1.169117
H 4.215166 -1.475753 0.866491
H 3.142446 -1.633554 2.257353
N 1.111334 -0.802300 0.079362
N 2.592817 -2.772462 0.604135
C 2.613306 3.224541 0.188612
C 3.720832 3.536284 -0.813946
H 4.009889 2.640974 -1.374121
H 2.967354 2.494845 0.924408
H 2.354120 4.119231 0.760966
H 3.386243 4.279855 -1.542272
H 4.614481 3.926703 -0.323246

State 6 with propyl and DABCO (Figure 6) C16H29N3S2
-1587.52948059 -1587.086794 -1587.062687 -1587.139008
C -1.859018 1.057272 2.371423
C -3.296180 2.126046 0.612821
C -3.375676 -1.065929 1.094725
C 1.215533 2.635950 -0.474306
C -2.144579 1.147762 0.876484
C -0.899698 1.564644 0.110743
H -2.819983 -1.578995 1.886335
H -1.445965 2.011849 2.693589
H -2.765737 0.837307 2.940008
H -1.108770 0.291432 2.579644
N -2.504574 -0.190623 0.328747
H -3.019259 3.106514 1.004350
H -3.483262 2.216761 -0.458393
H -4.214661 1.796903 1.106649
H -3.811586 -1.805384 0.427782
H -4.166918 -0.471420 1.554709
H 0.535949 0.137127 0.189715
C 0.154108 2.242438 0.518662
H 0.788197 3.368423 -1.174466
H 1.507870 1.784344 -1.123061
C -2.087415 -0.489074 -0.910737
S -2.302012 -1.960706 -1.691182
S -1.189010 0.838858 -1.629087
C 1.342534 -3.039502 0.956908
C 0.474431 -1.761318 1.047507
H 0.878711 -3.766973 0.288280
H 1.444962 -3.500334 1.940515
H -0.525481 -1.903632 0.636135
H 0.401953 -1.369108 2.062958
C 2.542274 -2.325630 -0.974213
H 3.511352 -1.955251 -1.314521
H 2.293552 -3.206523 -1.567268
C 1.442836 -1.247510 -1.141424
H 1.747414 -0.409972 -1.767326
H 0.503056 -1.645287 -1.528543
C 2.433112 -0.296813 0.891367
H 2.951844 0.358619 0.189930
H 2.166863 0.290697 1.771789
C 3.223457 -1.592995 1.195289
H 4.274856 -1.462459 0.935401
H 3.172058 -1.843462 2.256831
N 1.157123 -0.708127 0.228766
N 2.671997 -2.716031 0.433369
C 2.474995 3.246709 0.143556
C 3.560249 3.533353 -0.890742
H 3.878656 2.613683 -1.393829
H 2.859446 2.578943 0.922148
H 2.189348 4.164966 0.663730
H 3.190518 4.212228 -1.664042
H 4.443765 3.989621 -0.439319

State 5 with propyl and DBU (Figure 6) C19H33N3S2
-1704.30094979 -1703.794287 -1703.766427 -1703.851118

C -3.087846 -0.235211 2.229821
C -4.260982 -1.063661 0.172748
C -1.714616 -2.549548 1.636697
C -1.895417 3.210782 -0.629051
C -2.919306 -0.561306 0.726977
C -2.533267 0.735074 0.125965
H -1.186243 -2.137452 2.502936
H -3.785312 0.597668 2.316723
H -3.491147 -1.078936 2.789526
H -2.135475 0.069793 2.669357
N -1.843903 -1.579186 0.551609
H -5.040045 -0.320763 0.357921
H -4.180060 -1.247327 -0.896017
H -4.533611 -1.991302 0.682957
H -1.158796 -3.407448 1.274270
H -2.706319 -2.879251 1.956642
C -2.274829 1.873318 -0.172951
H -2.725843 3.902862 -0.454653
H -1.732165 3.188336 -1.713069
C -1.166880 -1.776753 -0.635787
S 0.284744 -2.711630 -0.597222
S -1.744209 -1.084775 -2.080811
C -0.636176 3.742134 0.071190
C -0.280439 5.150103 -0.396001
H -0.103455 5.169616 -1.474436
H 0.197071 3.060967 -0.129646
H -0.801241 3.730065 1.152680
H -1.091846 5.849605 -0.179544
H 0.620128 5.518591 0.098520
C 0.837756 0.334317 1.152617
C 1.631945 1.214701 2.132273
C 2.682384 0.448487 2.934783
C 1.529566 0.101264 -0.158061
C 3.790330 -0.168468 2.084785
C 3.282243 -1.118207 1.001580
H 0.625306 -0.649954 1.585490
H 2.096932 2.045878 1.590207
H 2.178477 -0.350848 3.490873
H 4.388894 0.616317 1.609641
H -0.126645 0.791753 0.934027
H 0.914690 1.660405 2.826175
H 3.127858 1.114960 3.678239
H 4.463764 -0.733668 2.735321
H 4.106527 -1.731919 0.638221
H 2.520777 -1.809130 1.380513
N 2.743792 -0.435398 -0.184642
N 0.935171 0.540893 -1.255114
C 3.372072 -0.796098 -1.462266
H 3.096425 -1.833292 -1.685823
H 4.453089 -0.735407 -1.322981
C 2.913785 0.129496 -2.575953
H 3.315934 1.134142 -2.418144
H 3.289784 -0.240890 -3.530324
C 1.396780 0.161407 -2.589847
H 1.006265 0.891228 -3.298606
H 0.970887 -0.819415 -2.823047

H -0.071219 0.692161 -1.175518

TS3 with propyl and DBU (Figure 6) C19H33N3S2

-1704.28297817 -1703.777958 -1703.750855 -1703.833202

C -2.133654 -2.095490 2.019571
C -3.441817 -2.608358 -0.051317
C -0.336494 -3.466893 0.238175
C -2.883728 2.074473 0.398265
C -2.246589 -1.830691 0.517462
C -2.387719 -0.351893 0.260860
H 0.192875 -3.325281 1.186220
H -3.002165 -1.652700 2.504524
H -2.109982 -3.167199 2.230792
H -1.246748 -1.624769 2.445799
N -1.001481 -2.247438 -0.186377
H -4.349930 -2.291082 0.464682
H -3.555361 -2.411551 -1.117214
H -3.310431 -3.683158 0.102880
H 0.372153 -3.767906 -0.528771
H -1.080440 -4.255057 0.377629
C -2.709648 0.691928 0.934077
H -3.959228 2.251924 0.276930
H -2.456771 2.170734 -0.621190
C -0.625847 -1.544931 -1.274312
S 0.839278 -1.786388 -2.096552
S -1.765932 -0.321565 -1.735648
C -2.324138 3.183677 1.295199
C -2.585500 4.577594 0.729917
H -2.142956 4.694435 -0.264553
H -1.245605 3.038105 1.439511
H -2.772602 3.083976 2.287215
H -3.659155 4.756366 0.627558
H -2.175774 5.359293 1.373277
C 1.217253 -0.360758 1.326698
C 2.177434 -0.144808 2.507641
C 3.554967 -0.773768 2.301733
C 1.413088 0.614085 0.205521
C 4.342788 -0.196766 1.127106
C 3.624284 -0.314241 -0.216431
H 1.331869 -1.366856 0.909300
H 2.271996 0.926919 2.712642
H 3.424873 -1.850727 2.141775
H 4.582457 0.856554 1.308628
H 0.181028 -0.263050 1.653265
H 1.715725 -0.590093 3.391883
H 4.141680 -0.662274 3.217321
H 5.293969 -0.729950 1.044219
H 4.335930 -0.171217 -1.029323
H 3.180163 -1.305001 -0.360054
N 2.579934 0.701918 -0.419403
N 0.403081 1.409214 -0.083920
C 2.709200 1.556262 -1.606172
H 2.426974 0.959449 -2.481680
H 3.758447 1.841858 -1.692242
C 1.826469 2.786493 -1.473621
H 2.191145 3.415858 -0.657307

H 1.867468 3.368189 -2.394879
C 0.398197 2.349538 -1.199791
H -0.236092 3.191786 -0.918280
H -0.044819 1.848218 -2.064875
H -0.496252 1.162892 0.333928

State 6 with propyl and DBU (Figure 6) C19H33N3S2
-1704.28312655 -1703.778195 -1703.750396 -1703.834877
C -2.068553 -2.125536 2.012324
C -3.363010 -2.692428 -0.050241
C -0.240953 -3.483936 0.218149
C -2.909460 1.983380 0.372717
C -2.187672 -1.879160 0.510706
C -2.350639 -0.403294 0.209246
H 0.266851 -3.338784 1.177129
H -2.939210 -1.681692 2.492512
H -2.035144 -3.195055 2.235537
H -1.186907 -1.641669 2.434185
N -0.934919 -2.278294 -0.196163
H -4.276899 -2.386708 0.462420
H -3.483221 -2.511231 -1.118726
H -3.211668 -3.763012 0.115613
H 0.487737 -3.751918 -0.542818
H -0.964000 -4.294871 0.334513
C -2.712435 0.610319 0.944542
H -3.986828 2.119376 0.214135
H -2.455887 2.100382 -0.634394
C -0.582522 -1.560012 -1.274269
S 0.851029 -1.758233 -2.151072
S -1.757803 -0.331403 -1.664334
C -2.428554 3.121676 1.279108
C -2.729049 4.502049 0.699761
H -2.265724 4.632709 -0.283735
H -1.350084 3.023964 1.462818
H -2.904495 3.004821 2.256226
H -3.805930 4.637013 0.567318
H -2.368891 5.303045 1.349324
C 1.229513 -0.342312 1.334510
C 2.205434 -0.130250 2.503333
C 3.586749 -0.739184 2.266587
C 1.395515 0.652504 0.226571
C 4.347616 -0.138065 1.086007
C 3.608492 -0.248147 -0.247149
H 1.352979 -1.341122 0.902503
H 2.289476 0.939812 2.721046
H 3.467260 -1.815695 2.095517
H 4.577169 0.915929 1.276538
H 0.197061 -0.264038 1.677603
H 1.762830 -0.592674 3.388492
H 4.187857 -0.631851 3.173279
H 5.303960 -0.657834 0.980568
H 4.304417 -0.087010 -1.070221
H 3.174505 -1.242501 -0.395660
N 2.548625 0.757586 -0.420412
N 0.374390 1.444568 -0.029384
C 2.654393 1.639661 -1.588713

H 2.365493 1.060908 -2.474019
H 3.700026 1.935727 -1.683009
C 1.764377 2.859916 -1.415380
H 2.137992 3.474800 -0.592140
H 1.785231 3.461523 -2.324457
C 0.344087 2.406933 -1.125781
H -0.292020 3.238363 -0.818466
H -0.108669 1.920088 -1.994151
H -0.513528 1.191675 0.410565

State 5 with benzyl and DABCO (Figure S6) C20H29N3S2
-1739.95573008 -1739.486544 -1739.460560 -1739.541962
C 3.110559 -1.949935 1.983359
C 3.073843 -2.802428 -0.374440
C 4.256112 0.102513 0.576955
C -1.392347 -2.620930 1.376411
C 2.546058 -1.704365 0.563765
C 1.081275 -1.841771 0.748539
H 4.200917 0.394771 1.627710
H 2.707052 -2.895175 2.345220
H 4.197751 -2.019667 1.983540
H 2.796986 -1.159896 2.668066
N 2.958545 -0.356229 0.075682
H 2.821228 -3.786049 0.026927
H 2.640248 -2.692316 -1.365910
H 4.161900 -2.721113 -0.442726
H 4.567689 0.961016 -0.006130
H 4.999720 -0.691853 0.467312
H -0.288172 0.334184 -0.264780
C -0.022285 -2.177305 1.102650
H -1.706075 -2.291202 2.372141
H -1.397201 -3.716614 1.408705
C 2.368985 0.289033 -0.989117
S 2.842418 1.886953 -1.379913
S 1.120578 -0.492814 -1.881727
C -0.122065 3.152558 1.548950
C 0.310373 1.713318 1.180665
H 0.574604 3.873029 1.116411
H -0.120199 3.288282 2.631746
H 1.306973 1.682740 0.744118
H 0.253472 1.014230 2.016909
C -1.410661 3.480523 -0.433258
H -2.429575 3.607296 -0.804940
H -0.830917 4.351123 -0.742599
C -0.766938 2.190016 -0.991415
H -1.362564 1.700104 -1.762171
H 0.246208 2.329524 -1.374743
C -1.995568 1.030468 0.788025
H -2.693784 0.721138 0.008657
H -1.901956 0.221328 1.511307
C -2.376797 2.382388 1.449565
H -3.395513 2.660556 1.175228
H -2.333549 2.304327 2.537828
N -0.660107 1.217406 0.149137
N -1.462494 3.443330 1.031845
C -2.402481 -2.142968 0.346037

C -3.730811 -1.931419 0.715932
C -2.012869 -1.886981 -0.967727
C -4.654070 -1.450336 -0.207018
H -4.042873 -2.130917 1.736872
C -2.935235 -1.400310 -1.891484
H -0.979046 -2.041616 -1.258703
C -4.255846 -1.175289 -1.513449
H -5.682344 -1.286161 0.093917
H -2.613432 -1.195433 -2.906423
H -4.972486 -0.794091 -2.231569

TS3 with benzyl and DABCO (Figure S6) C20H29N3S2
-1739.93843718 -1739.470066 -1739.444868 -1739.523427
C 2.114542 -1.421239 2.421424
C 3.306170 -2.659735 0.590712
C 3.800232 0.458132 1.067450
C -1.173473 -2.934684 -0.416579
C 2.296530 -1.544862 0.904919
C 0.970941 -1.875362 0.290333
H 3.375153 0.957989 1.943046
H 1.667367 -2.345554 2.784000
H 3.069298 -1.264089 2.926921
H 1.432464 -0.605405 2.671992
N 2.775827 -0.263920 0.326059
H 2.940912 -3.601270 1.004246
H 3.420956 -2.769689 -0.487940
H 4.279025 -2.434355 1.035032
H 4.246677 1.200729 0.412691
H 4.573504 -0.235177 1.408522
H 0.092280 0.176840 0.108453
C -0.123544 -2.460589 0.533064
H -1.425288 -3.976258 -0.192574
H -0.778294 -2.907465 -1.443528
C 2.473988 0.034765 -0.969523
S 3.008270 1.448807 -1.736161
S 1.438006 -1.097978 -1.762824
C -0.083751 3.296321 1.360810
C 0.665138 1.991696 0.994029
H 0.545484 4.158527 1.136065
H -0.327967 3.325507 2.424789
H 1.508994 2.160901 0.322989
H 1.004163 1.435064 1.867141
C -1.042442 3.189727 -0.818734
H -1.926012 3.445076 -1.405610
H -0.237574 3.868237 -1.108423
C -0.630943 1.722311 -1.079751
H -1.438943 1.121086 -1.496440
H 0.257167 1.624593 -1.705221
C -1.561668 0.981435 1.072338
H -2.170240 0.203880 0.621117
H -1.255055 0.653336 2.066880
C -2.255536 2.364108 1.065614
H -3.120148 2.348116 0.398679
H -2.609014 2.615962 2.066707
N -0.310146 1.118642 0.259438
N -1.332382 3.403195 0.603458

C -2.433194 -2.103227 -0.357742
C -3.257817 -2.123994 0.771289
C -2.773877 -1.265576 -1.419559
C -4.387149 -1.316293 0.840200
H -2.991888 -2.768216 1.602796
C -3.906071 -0.452968 -1.354673
H -2.139668 -1.248955 -2.300952
C -4.713068 -0.472044 -0.221887
H -5.018313 -1.344547 1.721298
H -4.161290 0.186164 -2.193180
H -5.597303 0.152774 -0.170833

State 6 with benzyl and DABCO (Figure S6) C20H29N3S2
-1739.94414186 -1739.476312 -1739.450684 -1739.531004
C 1.816789 -1.399496 2.342677
C 3.202750 -2.774679 0.766044
C 3.923827 0.319636 1.293051
C -1.137182 -2.338409 -0.872255
C 2.265869 -1.566816 0.894901
C 1.072213 -1.715787 -0.037721
H 3.412908 0.956978 2.022502
H 1.186918 -2.248656 2.602243
H 2.670270 -1.353088 3.023133
H 1.212178 -0.497220 2.466124
N 2.971664 -0.344977 0.419930
H 2.671127 -3.669029 1.095840
H 3.507300 -2.910878 -0.272909
H 4.096219 -2.649483 1.384033
H 4.590249 0.932657 0.691187
H 4.501124 -0.432633 1.833198
H -0.063260 0.005907 0.426238
C -0.146731 -2.134969 0.249497
H -1.212779 -3.422239 -1.029462
H -0.816591 -1.918681 -1.843039
C 2.775185 0.027062 -0.855665
S 3.382069 1.413611 -1.578048
S 1.709926 -1.084425 -1.697165
C 0.082757 3.369455 0.755890
C 0.578792 1.929971 1.040704
H 0.674762 3.820431 -0.042930
H 0.190198 3.989402 1.647339
H 1.556447 1.740033 0.598914
H 0.600511 1.697965 2.106841
C -1.419387 2.737060 -0.985078
H -2.475471 2.560821 -1.202590
H -1.028650 3.427744 -1.733358
C -0.621028 1.413477 -1.020025
H -1.147068 0.601890 -1.519912
H 0.369061 1.523992 -1.466118
C -1.708775 1.057024 1.156814
H -2.439615 0.502318 0.571102
H -1.563491 0.540428 2.106442
C -2.086004 2.549249 1.298065
H -3.153104 2.678091 1.110581
H -1.874249 2.921461 2.302741
N -0.409543 0.998614 0.408032

N -1.322405 3.355554 0.342426
C -2.520667 -1.811651 -0.553107
C -3.106251 -2.053111 0.694457
C -3.225375 -1.036257 -1.474935
C -4.345312 -1.512054 1.015074
H -2.549668 -2.641711 1.414857
C -4.462870 -0.478869 -1.153581
H -2.795810 -0.861333 -2.457597
C -5.023752 -0.708451 0.098204
H -4.784325 -1.708754 1.987068
H -4.988742 0.126818 -1.883186
H -5.985464 -0.278854 0.353354

State 5 with benzyl and DBU (Figure S6) C23H33N3S2
-1856.71295646 -1856.182450 -1856.152844 -1856.241874
C -4.084975 -0.709355 1.627654
C -4.564328 0.151742 -0.676456
C -3.021478 -2.678146 0.031790
C -1.232967 2.970672 1.691296
C -3.444909 -0.233984 0.301963
C -2.634667 0.934495 0.704243
H -2.777743 -2.972613 1.056280
H -4.600611 0.141470 2.072613
H -4.811910 -1.505500 1.469141
H -3.319737 -1.048305 2.328018
N -2.583802 -1.314706 -0.264935
H -5.216221 0.897889 -0.216803
H -4.142442 0.553209 -1.594800
H -5.158827 -0.736179 -0.908283
H -2.525531 -3.360150 -0.649450
H -4.102401 -2.757561 -0.111279
C -2.063578 1.856303 1.227071
H -0.935944 2.813812 2.732330
H -1.823850 3.892521 1.671939
C -1.605844 -1.092030 -1.208403
S -0.513650 -2.374066 -1.583595
S -1.478735 0.426674 -1.981576
C 0.005226 3.147631 0.828476
C 1.279997 3.126658 1.389850
C -0.126184 3.331912 -0.550034
C 2.409876 3.302928 0.593584
H 1.391684 2.975478 2.459252
C 0.999228 3.518428 -1.344620
H -1.114245 3.301283 -0.996819
C 2.270898 3.506329 -0.774274
H 3.395620 3.285162 1.044164
H 0.880652 3.661958 -2.412806
H 3.147891 3.652139 -1.394822
C 0.626255 -0.511514 1.200077
C 1.362856 -0.375537 2.540791
C 1.754329 -1.718256 3.157263
C 1.536379 -0.686719 0.022151
C 2.735308 -2.534576 2.316857
C 2.248818 -2.837788 0.899666
H -0.064506 -1.363040 1.211763
H 2.245619 0.260676 2.411019

H 0.842218 -2.308259 3.306221
H 3.699900 -2.019613 2.251142
H 0.014707 0.368656 1.006030
H 0.696811 0.150149 3.230658
H 2.185790 -1.552891 4.148392
H 2.917703 -3.489598 2.818031
H 2.846888 -3.644713 0.475718
H 1.206493 -3.176482 0.884324
N 2.389910 -1.707489 -0.029388
N 1.512084 0.248655 -0.902149
C 3.124650 -1.982302 -1.270397
H 2.509318 -2.653014 -1.881632
H 4.049265 -2.495077 -0.999111
C 3.422292 -0.697224 -2.027892
H 4.148086 -0.097175 -1.471732
H 3.855972 -0.942513 -2.998085
C 2.133180 0.084725 -2.208928
H 2.314030 1.079335 -2.617768
H 1.418813 -0.444558 -2.848989
H 0.695155 0.860213 -0.887001

TS3 with benzyl and DBU (Figure S6) C23H33N3S2
-1856.69549588 -1856.165314 -1856.136727 -1856.222308
C -3.131216 -0.044953 2.142001
C -4.610572 0.273009 0.130395
C -2.873769 -2.401376 0.516149
C -1.138695 3.452047 0.078330
C -3.165729 0.104923 0.619048
C -2.331613 1.293264 0.221910
H -2.237934 -2.638212 1.377583
H -3.458599 0.896153 2.580857
H -3.788272 -0.849070 2.481281
H -2.113037 -0.234861 2.489522
N -2.566019 -1.090860 -0.030270
H -5.046850 1.152920 0.606267
H -4.627479 0.413106 -0.951158
H -5.214131 -0.602084 0.387629
H -2.720139 -3.153451 -0.253007
H -3.914653 -2.420602 0.843816
C -1.927147 2.377096 0.759567
H -1.408773 4.441149 0.465334
H -1.349999 3.467894 -1.002452
C -1.857301 -0.927564 -1.164444
S -1.040481 -2.200588 -1.937272
S -1.829682 0.702000 -1.753981
C 0.354079 3.262213 0.260627
C 0.887859 2.833123 1.482091
C 1.235938 3.498901 -0.797043
C 2.256373 2.639963 1.634082
H 0.205710 2.642246 2.304225
C 2.608488 3.307395 -0.646809
H 0.838037 3.832089 -1.750826
C 3.124204 2.869727 0.567753
H 2.649276 2.313230 2.591009
H 3.274244 3.502044 -1.480955
H 4.190944 2.719304 0.687748

C 0.510317 -0.710346 1.241398
C 1.396080 -0.841554 2.489964
C 1.681547 -2.290416 2.884876
C 1.267579 -0.824217 -0.046165
C 2.455381 -3.089851 1.837095
C 1.776818 -3.148795 0.468591
H -0.260023 -1.489619 1.232505
H 2.331413 -0.295050 2.328923
H 0.726144 -2.793720 3.076897
H 3.461320 -2.675684 1.709149
H -0.019241 0.244924 1.234786
H 0.880809 -0.341766 3.314232
H 2.236422 -2.306552 3.826655
H 2.576086 -4.116101 2.195311
H 2.212065 -3.953668 -0.123955
H 0.706700 -3.367293 0.551761
N 1.952124 -1.927902 -0.330257
N 1.264611 0.213623 -0.849862
C 2.589083 -2.094540 -1.642318
H 1.880907 -2.619012 -2.293766
H 3.472165 -2.720732 -1.500461
C 2.974038 -0.750899 -2.241087
H 3.791693 -0.305436 -1.667463
H 3.316999 -0.898037 -3.265649
C 1.769295 0.174368 -2.214544
H 2.033019 1.192500 -2.498406
H 0.964742 -0.188637 -2.862519
H 0.671811 0.996306 -0.590889

State 6 with benzyl and DBU (Figure S6) C23H33N3S2
-1856.69589867 -1856.165505 -1856.136401 -1856.223135
C -3.085497 -0.035464 2.131693
C -4.599257 0.249873 0.144262
C -2.853279 -2.420646 0.497367
C -1.157983 3.419553 0.026365
C -3.146045 0.097058 0.611995
C -2.309934 1.273780 0.150263
H -2.223561 -2.648929 1.365388
H -3.391872 0.916810 2.561664
H -3.745203 -0.829220 2.491649
H -2.063589 -0.229243 2.466000
N -2.554323 -1.105755 -0.039800
H -5.025051 1.138000 0.614212
H -4.637293 0.375696 -0.939205
H -5.201665 -0.619708 0.423225
H -2.684177 -3.168759 -0.273007
H -3.896643 -2.450202 0.815902
C -1.950661 2.365634 0.760480
H -1.439605 4.419563 0.376470
H -1.347577 3.410604 -1.059998
C -1.848411 -0.925041 -1.164301
S -1.052603 -2.156999 -2.008737
S -1.806146 0.743620 -1.668409
C 0.333742 3.254596 0.234914
C 0.854079 2.846043 1.470399
C 1.232739 3.491696 -0.809529

C 2.222230 2.669681 1.646059
H 0.158510 2.658343 2.281698
C 2.605186 3.320053 -0.634485
H 0.847182 3.812405 -1.772764
C 3.105846 2.899346 0.592472
H 2.603069 2.357526 2.612784
H 3.282744 3.516905 -1.458699
H 4.172221 2.763706 0.732152
C 0.511208 -0.701011 1.244477
C 1.391152 -0.842635 2.495850
C 1.656002 -2.295485 2.890974
C 1.273287 -0.828660 -0.038596
C 2.413583 -3.110255 1.842624
C 1.728835 -3.164800 0.476627
H -0.268132 -1.470949 1.235663
H 2.333266 -0.306822 2.337877
H 0.693370 -2.783432 3.086886
H 3.425080 -2.711830 1.708685
H -0.008496 0.259958 1.234151
H 0.878911 -0.336606 3.318071
H 2.213346 -2.319309 3.831077
H 2.520556 -4.136689 2.204581
H 2.146613 -3.980253 -0.113958
H 0.655809 -3.364605 0.567104
N 1.923159 -1.950910 -0.328022
N 1.305619 0.213667 -0.835965
C 2.570803 -2.126415 -1.633614
H 1.856069 -2.625557 -2.297488
H 3.433555 -2.778936 -1.486276
C 3.002477 -0.789727 -2.216312
H 3.826080 -0.374240 -1.629122
H 3.352932 -0.937935 -3.238129
C 1.826357 0.172169 -2.194328
H 2.124406 1.184302 -2.464978
H 1.020523 -0.161138 -2.856638
H 0.748664 1.019544 -0.568063
----

10. References

[1] C. J. Pierce, C. H. Larsen, *Green Chem.* **2012**, *14*, 2672–2676.
[2] A. Ranjan, R. Yerande, P. B. Wakchaure, S. G. Yerande, D. H. Dethe, *Org. Lett.* **2014**, *16*, 5788–5791.
[3] A. D. Gammack Yamagata, S. Datta, K. E. Jackson, L. Stegbauer, R. S. Paton, D. J. Dixon, *Angew. Chem. Int. Ed.* **2015**, *54*, 4899–4903.
[4] H. Wang, J. Ying, M. Lai, X. Qi, J.-B. Peng, X.-F. Wu, *Adv. Synth. Catal.* **2018**, *360*, 1693–1703.
[5] S. P. Neofotistos, N. V. Tzouras, M. Pauze, E. Gómez-Bengoa, G. C. Vougioukalakis, *Adv. Synth. Catal.* **2020**, *362*, 3872–3885.
[6] J. Ying, Z. Le, X.-F. Wu, *Org. Lett.* **2020**, *22*, 194–198.
[7] F. Schlimpen, C. Plaçais, E. Starck, V. Bénéteau, P. Pale, S. Chassaing, *J. Org. Chem.* **2021**, *86*, 16593–16613.
[8] C. J. Pierce, M. Nguyen, C. H. Larsen, *Angew. Chem. Int. Ed.* **2012**, *51*, 12289–12292.
